# Associations between COVID‐19 therapies and outcomes in rural and urban America: A multisite, temporal analysis from the Alpha to Omicron SARS‐CoV‐2 variants

**DOI:** 10.1111/jrh.12857

**Published:** 2024-07-02

**Authors:** A. Jerrod Anzalone, William H. Beasley, Kimberly Murray, William B. Hillegass, Makayla Schissel, Michael T. Vest, Scott A. Chapman, Ronald Horswell, Lucio Miele, J. Zachary Porterfield, H. Timothy Bunnell, Bradley S. Price, Sharon Patrick, Clifford J. Rosen, Susan L. Santangelo, James C. McClay, Sally L. Hodder, the National COVID Cohort Collaborative (N3C) Consortium

**Affiliations:** ^1^ University of Nebraska Medical Center Omaha Nebraska USA; ^2^ University of Oklahoma Norman Oklahoma USA; ^3^ Maine Health Institute for Research Portland Maine USA; ^4^ University of Mississippi Medical Center Jackson Mississippi USA; ^5^ Christiana Care Health System Newark Delaware USA; ^6^ University of Minnesota College of Pharmacy Minneapolis Minnesota USA; ^7^ Louisiana State University Health Sciences Center New Orleans Louisiana USA; ^8^ University of Kentucky Lexington Kentucky USA; ^9^ Nemours Children's Health Wilmington Delaware USA; ^10^ West Virginia University Morgantown West Virginia USA; ^11^ Tufts University School of Medicine Boston Massachusetts USA; ^12^ University of Missouri School of Medicine Columbia Missouri USA

**Keywords:** COVID‐19 Therapies, Mortality, National COVID Cohort Collaborative (N3C), SARS‐CoV‐2, Urban‐Rural Health

## Abstract

**Purpose:**

To investigate the enduring disparities in adverse COVID‐19 events between urban and rural communities in the United States, focusing on the effects of SARS‐CoV‐2 vaccination and therapeutic advances on patient outcomes.

**Methods:**

Using National COVID Cohort Collaborative (N3C) data from 2021 to 2023, this retrospective cohort study examined COVID‐19 hospitalization, inpatient death, and other adverse events. Populations were categorized into urban, urban‐adjacent rural (UAR), and nonurban‐adjacent rural (NAR). Adjustments included demographics, variant‐dominant waves, comorbidities, region, and SARS‐CoV‐2 treatment and vaccination. Statistical methods included Kaplan‐Meier survival estimates, multivariable logistic, and Cox regression.

**Findings:**

The study included 3,018,646 patients, with rural residents constituting 506,204. These rural dwellers were older, had more comorbidities, and were less vaccinated than their urban counterparts. Adjusted analyses revealed higher hospitalization odds in UAR and NAR (aOR 1.07 [1.05–1.08] and 1.06 [1.03–1.08]), greater inpatient death hazard (aHR 1.30 [1.26–1.35] UAR and 1.37 [1.30–1.45] NAR), and greater risk of other adverse events compared to urban dwellers. Delta increased, while Omicron decreased, inpatient adverse events relative to pre‐Delta, with rural disparities persisting throughout. Treatment effectiveness and vaccination were similarly protective across all cohorts, but dexamethasone post‐ventilation was effective only in urban areas. Nirmatrelvir/ritonavir and molnupiravir better protected rural residents against hospitalization.

**Conclusions:**

Despite advancements in treatment and vaccinations, disparities in adverse COVID‐19 outcomes persist between urban and rural communities. The effectiveness of some therapeutic agents appears to vary based on rurality, suggesting a nuanced relationship between treatment and geographic location while highlighting the need for targeted rural health care strategies.

## INTRODUCTION

The first confirmed case of COVID‐19 in the United States was reported on January 20, 2020.^1^ Subsequently, the SARS‐CoV‐2 virus spread rapidly throughout the country, resulting in more than 100 million cases and 1 million deaths in the United States.^2^ The pandemic first surged in urban areas. Still, new variants like Delta and Omicron and uneven vaccination distribution later caused a disproportionate impact on rural regions with lower vaccination and health care access.^3^, ^4^ Early in the pandemic, urban areas were hit hardest, with higher rates of infection, hospitalization, and death than rural areas.^5^ This was likely due to the greater population density in urban areas and the ease of virus transmission in crowded public spaces.^6^ However, rural areas experienced significant impacts as the pandemic progressed with higher hospitalizations and mortality rates.^7^ On December 12, 2020, the cumulative death rate in rural areas first exceeded urban areas.^8^ From January 20, 2020, through January 20, 2023, the cumulative death rate in rural counties was 37% higher than in urban counties, 411 versus 301 deaths per 100,000 persons, respectively.^8^

Delta was the most prominent early SARS‐CoV‐2 variant.^1^ Its impact was amplified in rural areas of the United States, particularly in the South and Midwest, due to lower vaccination rates and limited health care resources, and this pattern continued into the Omicron‐dominant period.^9^ Rural dwellers were more reluctant to be vaccinated,^10^ contributing to a higher risk of community transmission.^11^ Additionally, the pandemic disproportionately affected communities of color, with higher rates of hospitalization and death observed among Hispanic and Black individuals.^12^, ^13^ While many factors contributed to these differences, vaccination rates emerged as a critical determinant in the latest stages of the pandemic. The highly transmissible Omicron variant disproportionately impacted medically underserved communities^14^, ^15^ and those with low vaccination rates.^9^ Through March 15, 2023, primary vaccination series completion rates were lower in rural versus urban counties (55.2% rural, 66.5% urban), as were rates for updated bivalent booster doses (11.5% rural, 18.0% urban).^16^

Rural areas often offer fewer health care resources, including hospitals, medical professionals, and specialized medical equipment,^6^ thereby increasing barriers for rural patients attempting to access necessary care, including intensive care and specialized treatments.^17^ Urban and rural differences in availability^18^ and prescribing rates^19^ of COVID‐19 therapies, such as nirmatrelvir/ritonavir and molnupiravir, have been described and may contribute to disparate outcomes.^20^, ^21^ However, no nationwide studies have examined the impact of delayed access to COVID‐19 therapies on patient outcomes or the persistent gap in case fatality experienced by rural compared with urban communities.

To better understand the differential impact of COVID‐19 over time in rural America, we assessed treatments and adverse acute COVID‐19 events using the National COVID Cohort Collaborative (N3C), a centralized data enclave containing electronic health record (EHR) information on SARS‐CoV‐2‐infected persons from across the United States, representing the single largest limited data set gathered in US history.^22^ Our study aimed to (1) estimate differences in hospitalization and adverse inpatient events (AIEs) throughout the COVID‐19 pandemic among rural and urban dwellers and (2) quantify differences in treatment rates and their impact on mediating adverse events, if any, based on rurality. Compared to urban residents, we hypothesized that rural residents may present with more severe COVID‐19 due to disparities in treatment access, even after adjusting for vaccination rates.

## METHODS

This retrospective cohort study received Institutional Review Board (IRB) approval from each investigator's home institution. It was reviewed by the N3C Data Access Committee (RP‐B3442B), which operates under the authority of the National Institutes of Health IRB, with Johns Hopkins University School of Medicine serving as the central IRB. No informed consent was obtained because the study used a limited data set. This study followed the Enhancing the Quality and Transparency of Health Research (EQUATOR) reporting guidelines: Reporting of Studies Conducted Using Observational Routinely Collected Health Data (RECORD).^23^ The Strobe Statement and Checklist is available in Methods S1. Data extraction and analyses were performed using PySpark, SQL, and R within the N3C Data Enclave per N3C privacy and download review policies.^24^

### National COVID Cohort Collaborative (N3C)

This study utilized data from the N3C, a centralized repository of EHR data on COVID‐19 patients and controls submitted by 83 medical centers nationwide (as of December 27, 2023). Each study site provides demographic, medication, laboratory, diagnoses, and vital status data harmonized into the Observational Medical Outcomes Partnership (OMOP) Common Data Model. Information is available for COVID‐19 encounters and a patient look‐back data period to January 1, 2018, to provide information on pre‐existing conditions and encounters. The N3C design, data collection, sampling approach, and data harmonization methods have been described previously.^24^, ^25^ An overview of N3C's ingestion and harmonization process, sampling approaches, overall structure, and data access process are provided in Methods S2.

### Data extraction and curation

Data were extracted in April 2023 (N3C release 120), in the OMOP Common Data Model version 5.3.1.^26^ Patients were included if they had a positive PCR or Antigen lab test or a definitive COVID‐19 diagnosis (U07.7) between January 1, 2021, and December 31, 2022, which facilitates at least 3 months of follow‐up through the data extraction period to account for reporting variance across N3C sites through March 31, 2023.

Clinical concept sets were co‐developed with an informatician and a clinical expert reviewing each. These sets include standardized terminologies (eg, LOINC, SNOMED CT, and RxNorm). Methods S3 details the concept set definitions and the logic for timing and computable phenotypes used in this study. Employing a methodology described in prior research,^7^ sites were excluded from the study if they exhibited missing data in a significant domain (such as conditions or medication exposure, which encapsulates vaccination reporting), defined as being more than 1 standard deviation from the mean across all N3C sites, proportion of clinical domains relevant to this investigation (medications, measurements, death, and conditions), did not provide 5‐digit ZIP Codes, or were contributing only pediatric data (eg, pediatric hospitals and medical centers). We excluded patients missing age or sex values.

### Covariates

Persons were classified based on rurality, identified by mapping 5‐digit ZIP Codes to the 2010 Rural‐Urban Continuum Codes (RUCA).^27^ We grouped RUCA codes into a binary urban‐rural distinction (RUCA codes 1‐3 are urban and 4‐10 are rural). Further, we subclassified their degree of rurality into urban‐adjacent rural (UAR) and nonurban‐adjacent rural (NAR), using a previously described methodology based on commuting flows to urban clusters.^7^, ^11^ We utilized demographics (age, sex, and race/ethnicity) and diagnoses of comorbid conditions from January 2018 (the earliest pre‐pandemic lookback date) until a diagnosis of SARS‐CoV‐2 infection. Comorbid conditions included obesity, components of the Quan‐Charlson Comorbidity Index (CCI),^28^ history of tobacco use, substance abuse disorder, US Census region, vaccination status before SARS‐CoV‐2 infection (indicating whether the observed infection was a primary or booster breakthrough infection, defined as having completed a primary vaccination series with any additional doses before infection), and COVID‐19 epoch, delineated according to variant‐dominant periods based on Centers for Disease Control and Prevention (CDC) reporting.^29^ These epochs included pre‐Delta dominance (January 1, 2021‐June 14, 2021), Delta dominant (June 15, 2021‐December 21, 2021), and Omicron dominant (after December 22, 2021).

We selected medications using a priori selection from the CDC recommendations for COVID‐19 treatment over time, including nirmatrelvir/ritonavir, molnupiravir, spike protein monoclonal antibody therapies (mABs), remdesivir, anticoagulants (apixaban, betrixaban, dabigatran, enoxaparin, rivaroxaban, or warfarin administered during an inpatient stay), tocilizumab, and dexamethasone.^30^ Medications were assessed based on delivery timing between COVID‐19 diagnosis and each adverse outcome of interest (eg, between COVID‐19 diagnosis and date of death). Associations between nirmatrelvir/ritonavir, molnupiravir, and mABs were evaluated for COVID‐19 hospitalization and all medications for COVID‐19 AIEs. If a patient was prescribed anticoagulants within a year of their COVID‐19 diagnosis and continued the regimen after diagnosis, they were not categorized in the group who received anticoagulants as a COVID‐19 therapy. Medications were stratified based on when they were widely used or given Food and Drug Administration (FDA) emergency use authorization (described in Methods S3).

### Outcomes

**FIGURE 1:**
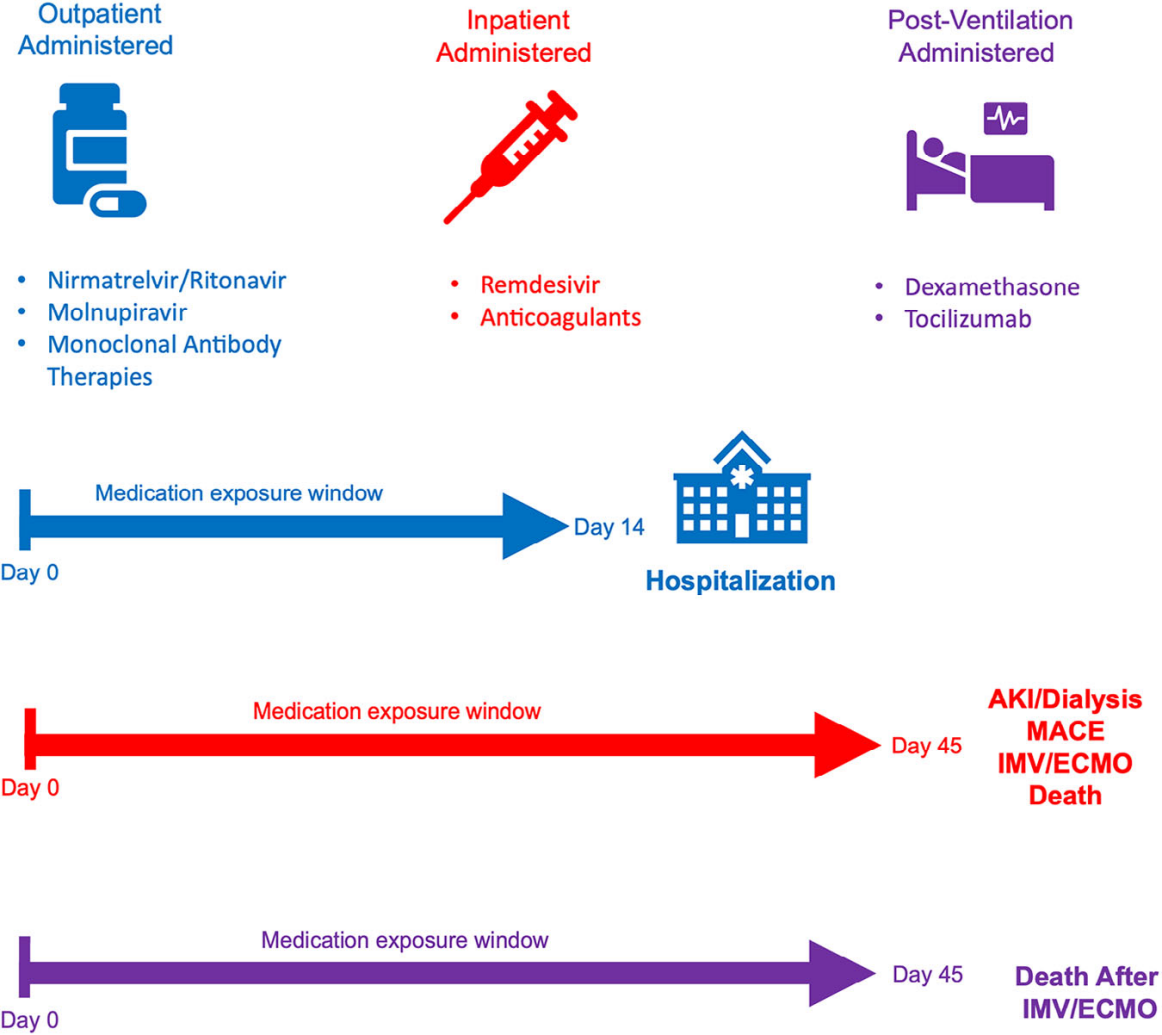
Medication exposure types and timing relevant to adverse events. COVID‐19 therapy timing relative to COVID‐19 diagnosis date (Day 0) and adverse post‐acute‐COVID‐19 event. Methods S3 contains concept set definitions and logic for timing, concept definitions (eg, RxNorm codes) for medications, and availability of medications based on FDA emergency use authorization.

The primary outcomes of interest included COVID‐19‐associated hospitalization (defined as occurring between 3 days before and 14 days after the initial acute COVID‐19 diagnosis documented) and AIEs, including inpatient major adverse cardiovascular event (MACE, defined as acute myocardial infarction, angina, stent occlusion/thrombosis, stroke, transient ischemic attack, congestive heart failure), acute kidney injury with or without dialysis (AKI/dialysis), invasive mechanical ventilation or extracorporeal membrane oxygenation (IMV/ECMO), or death in the 45 days after acute COVID diagnosis. We secondarily assessed the impact of dexamethasone and tocilizumab on death among patients receiving IMV/ECMO. Figure 1 provides temporal logic for COVID‐19 therapies relative to COVID‐19 diagnosis and adverse events, discharge, or censoring.

### Statistical analyses

We examined the proportion of patients who received COVID‐19 therapies by rurality within 14 days following the earliest documented SARS‐CoV‐2 infection. Baseline characteristics were reported for all patients in this study by rurality and hospitalization status. Trends for differences in targeted therapies were assessed throughout the study period in the overall cohort and stratified by rurality. Significant differences in event rates were determined using Chi‐square tests by rurality.

Multivariable logistic regression was used for the binary outcome of post‐COVID‐19 hospitalization, adjusting for the covariates listed above. This method was chosen for its ability to model the relationship between multiple independent variables and a binary outcome, providing insights into the factors contributing to hospitalization.

Multivariable Cox proportional hazards models among those hospitalized within 14 days after COVID‐19 diagnosis were performed to examine the association of rurality with each outcome, including time to inpatient death. This approach allows us to account for the time dimension of the data, offering a dynamic view of how rurality influences patient trajectories over time. Kaplan‐Meier (KM) survival analysis was utilized to assess the time to inpatient death by rurality and the COVID‐19 epoch. KM curves were compared with a log‐rank test. We secondarily assessed relative risk in stratified analyses by variant epoch and rurality. This sequential use of Cox models and KM analysis provides a comprehensive picture of survival probabilities, aiding in understanding how rurality impacts patient outcomes during different pandemic phases.

### Propensity score matching

To ensure the robustness of our findings and mitigate potential biases due to unrecognized and unaccounted‐for differences in site reporting and baseline risk at COVID‐19 diagnosis, we performed a propensity score‐matched (PSM) analysis. By using logistic regression with 1:1 matching for binary rurality (ie, Urban: UAR or NAR) with exact matching on sex, race/ethnicity, COVID‐19 epoch, and data‐contributing site and nearest neighbor PSM on composite CCI and age, we aimed to create comparable groups for analysis. This method enhances the validity of our conclusions by closely matching urban and rural patients on key characteristics, thereby reducing confounding and allowing for a more accurate comparison of outcomes across rurality.

### Sensitivity analyses

Several sensitivity analyses (SAs) were performed by repeating our primary and secondary analyses with the following modifications:

I. Evaluating model performance with a binary rural indicator (SA‐1).
II. To mitigate potential referral‐in bias and delays in care‐seeking behavior among rural dwellers, potentially resulting in greater severity at admission, we evaluated model performance after excluding patients who died in the first 3 days after COVID‐19 hospitalization (SA‐2) and adjusting for prior visit history (SA‐3).
III. To assess potential confounding between epochs and medication availability, we assessed drugs in combination and individually only when those therapies were available or widely used (SA‐4).

Statistical analyses were performed in R^31^; additional packages are detailed in Methods S2. Two‐sided *P*‐values < .05 were considered statistically significant.

## FINDINGS

### Demographic and clinical characteristics

**FIGURE 2:**
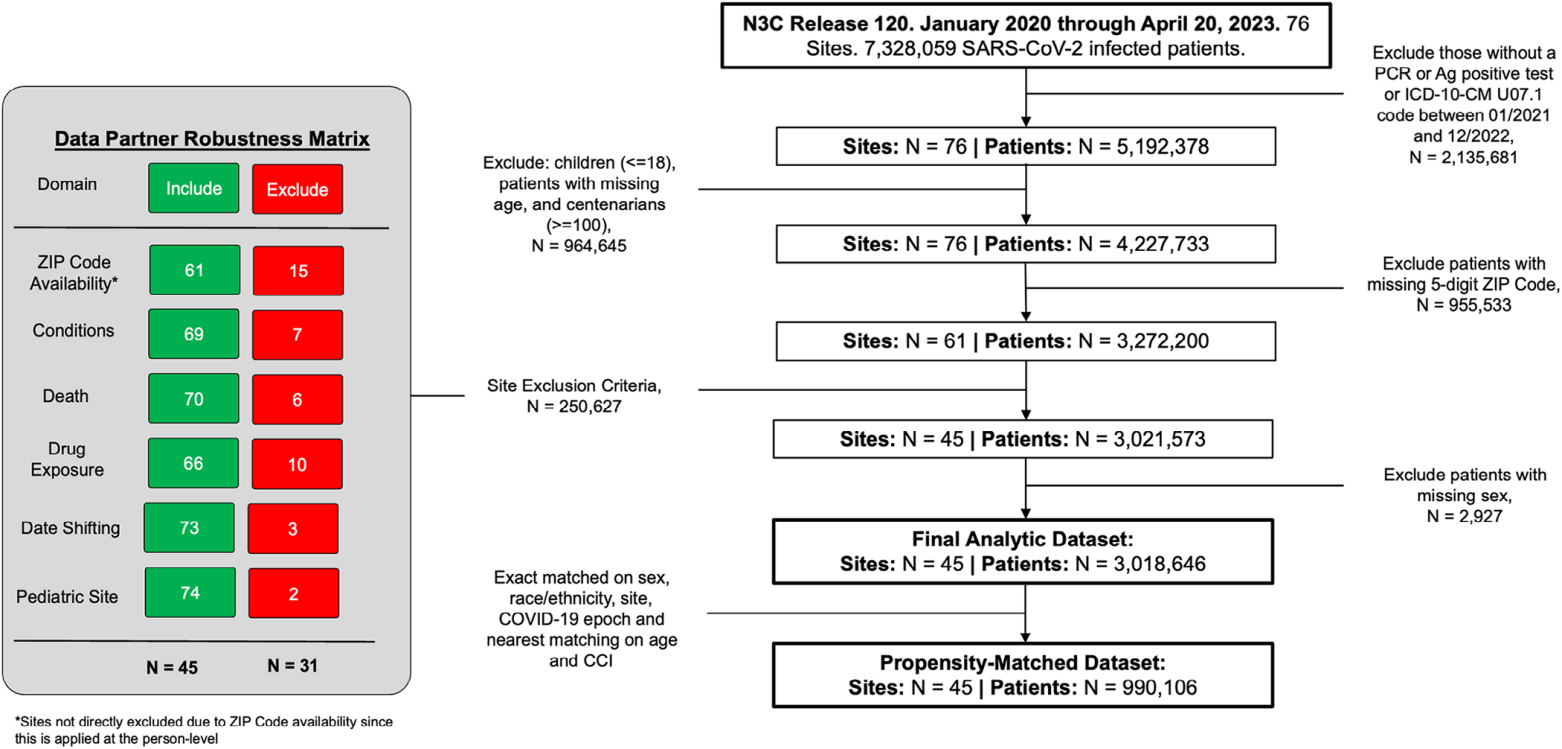
Analytic sample selection flow diagram. Figure 2 documents the data selection requirements, including steps for inclusion and exclusion of data partners based on the availability of 5‐digit ZIP Codes and reporting at the site level based on condition data availability, death reporting, drug exposure availability, date shifting, and contributions from pediatric sites (since we are interested in adults for this study, we also excluded patients with missing age or sex).

After applying inclusion and exclusion criteria, our sample included 3,018,646 SARS‐CoV‐2‐infected patients from 45 sites (Figure 2), of whom 2,512,442 were urban, 408,580 UAR, and 97,624 NAR dwellers. Hospitalization within 14 days of COVID‐19 diagnosis occurred among 299,513 persons (12% of the sample). Patient distribution by rurality was similar to US population distribution (Figure S1), with caseload and case fatality aligning with national reporting disparities through the study period (Figure S2).^32^

Patient demographics (Table 1) in the study demonstrated rural dwellers had less racial/ethnic diversity (90% UAR and 85% NAR non‐Hispanic White vs 64% urban). Medical visit history before COVID‐19 diagnosis was similar among rural and urban dwellers during the pre‐COVID‐19 observation period for both number of visits (median [IQR] visits 12 [3‐37] UAR and 12 [2‐41] vs 13 [3‐40] urban) and amount of observation time (median [IQR] days 883 [128‐1,306] UAR and 883 [102‐1,313] NAR vs 921 [126‐1,330] urban). Rural dwellers had a higher prevalence of tobacco usage (5.0% UAR and 5.2% NAR vs 4.4% urban) but lower documented substance use disorders (2.4% UAR and 2.3% NAR vs 2.7% urban). Rural dwellers had lower documented completion of at least a primary vaccination series (77% UAR and 82% NAR vs 75% urban) before SARS‐CoV‐2 infection throughout the observation period. Given that this study includes SARS‐CoV‐2‐infected patients over time, and vaccination rates have concurrently increased over time, patients with documented vaccination events prior to infection, as a result of this definition, had a breakthrough SARS‐CoV‐2 infection after vaccination, so the presented vaccination information must be interpreted in that context.

**TABLE 1:** Baseline characteristics of patients with SARS‐CoV‐2 infection by rurality, January 2021‐December 2022.

	SARS‐CoV‐2 infected				Hospitalized within 14 days of SARS‐CoV‐2 infection			
Characteristic^a^	Overall, N = 3,018,646	Urban, N = 2,512,442	Urban‐adjacent rural, N = 408,580	Nonurban‐adjacent rural, N = 97,624	Overall, N = 299,513	Urban, N = 248,770	Urban‐adjacent rural, N = 40,330	Nonurban‐adjacent rural, N = 10,413
Sex								
Female	1,723,967 (57%)	1,442,625 (57%)	227,890 (56%)	53,452 (55%)	152,354 (51%)	127,425 (51%)	19,966 (50%)	4,963 (48%)
Male	1,294,679 (43%)	1,069,817 (43%)	180,690 (44%)	44,172 (45%)	147,159 (49%)	121,345 (49%)	20,364 (50%)	5,450 (52%)
Age at COVID‐19 diagnosis, Median (IQR)	47 (33‐63)	47 (33‐62)	48 (33‐63)	51 (35‐65)	63 (47‐75)	62 (46‐75)	64 (50‐75)	65 (52‐75)
Race/ethnicity								
White non‐Hispanic	2,050,535 (68%)	1,614,892 (64%)	347,889 (85%)	87,754 (90%)	188,126 (63%)	144,542 (58%)	34,319 (85%)	9,265 (89%)
Black or African American non‐Hispanic	393,047 (13%)	370,104 (15%)	20,085 (4.9%)	2,858 (2.9%)	55,922 (19%)	52,434 (21%)	2,940 (7.3%)	548 (5.3%)
Hispanic or Latino any race	254,459 (8.4%)	234,102 (9.3%)	17,838 (4.4%)	2,519 (2.6%)	32,094 (11%)	30,024 (12%)	1,765 (4.4%)	305 (2.9%)
Other	139,640 (4.6%)	125,022 (5.0%)	12,585 (3.1%)	2,033 (2.1%)	10,073 (3.4%)	9,482 (3.8%)	518 (1.3%)	73 (0.7%)
Missing/unknown	180,965 (6.0%)	168,322 (6.7%)	10,183 (2.5%)	2,460 (2.5%)	13,298 (4.4%)	12,288 (4.9%)	788 (2.0%)	222 (2.1%)
Vaccination status prior to SARS‐CoV‐2 infection								
No documented COVID‐19 vaccination	2,278,507 (75%)	1,885,695 (75%)	313,139 (77%)	79,673 (82%)	250,484 (84%)	208,518 (84%)	33,041 (82%)	8,925 (86%)
Primary vaccination series	434,199 (14%)	361,156 (14%)	61,159 (15%)	11,884 (12%)	29,348 (9.8%)	23,989 (9.6%)	4,439 (11%)	920 (8.8%)
Primary+ vaccination series	305,940 (10%)	265,591 (11%)	34,282 (8.4%)	6,067 (6.2%)	19,681 (6.6%)	16,263 (6.5%)	2,850 (7.1%)	568 (5.5%)
Variant‐dominant epoch								
Pre‐Delta (January 1, 2021‐June 14, 2021)	528,579 (18%)	448,120 (18%)	64,890 (16%)	15,569 (16%)	72,260 (24%)	63,128 (25%)	7,337 (18%)	1,795 (17%)
Delta (June 15, 2021‐December 21, 2021)	778,430 (26%)	607,961 (24%)	136,429 (33%)	34,040 (35%)	87,063 (29%)	68,550 (28%)	14,695 (36%)	3,818 (37%)
Omicron (≥ December 22, 2021)	1,711,637 (57%)	1,456,361 (58%)	207,261 (51%)	48,015 (49%)	140,190 (47%)	117,092 (47%)	18,298 (45%)	4,800 (46%)
Comorbidities before SARS‐CoV‐2 infection								
Quan‐Charlson Comorbidity Index, Median (IQR)	0 (0‐1)	0 (0‐1)	0 (0‐1)	0 (0‐1)	2 (0‐5)	2 (0‐5)	2 (0‐4)	2 (0‐4)
Myocardial infarction	72,109 (2.4%)	59,726 (2.4%)	9,705 (2.4%)	2,678 (2.7%)	21,947 (7.3%)	18,373 (7.4%)	2,780 (6.9%)	794 (7.6%)
Congestive heart failure	142,091 (4.7%)	119,486 (4.8%)	17,764 (4.3%)	4,841 (5.0%)	46,362 (15%)	39,327 (16%)	5,530 (14%)	1,505 (14%)
Peripheral vascular disease	89,310 (3.0%)	75,800 (3.0%)	10,638 (2.6%)	2,872 (2.9%)	25,215 (8.4%)	21,433 (8.6%)	2,986 (7.4%)	796 (7.6%)
Cerebrovascular disease	97,097 (3.2%)	83,008 (3.3%)	11,051 (2.7%)	3,038 (3.1%)	24,936 (8.3%)	21,356 (8.6%)	2,803 (7.0%)	777 (7.5%)
Dementia	42,418 (1.4%)	36,288 (1.4%)	4,980 (1.2%)	1,150 (1.2%)	13,710 (4.6%)	11,994 (4.8%)	1,398 (3.5%)	318 (3.1%)
Chronic pulmonary disease	366,852 (12%)	312,562 (12%)	42,892 (10%)	11,398 (12%)	65,005 (22%)	54,476 (22%)	8,377 (21%)	2,152 (21%)
Rheumatologic disease	139,785 (4.6%)	119,118 (4.7%)	16,265 (4.0%)	4,402 (4.5%)	24,618 (8.2%)	20,690 (8.3%)	3,081 (7.6%)	847 (8.1%)
Peptic ulcer disease	31,875 (1.1%)	27,290 (1.1%)	3,555 (0.9%)	1,030 (1.1%)	6,819 (2.3%)	5,838 (2.3%)	763 (1.9%)	218 (2.1%)
Mild liver disease	112,409 (3.7%)	96,862 (3.9%)	12,221 (3.0%)	3,326 (3.4%)	17,062 (5.7%)	14,685 (5.9%)	1,849 (4.6%)	528 (5.1%)
Moderate to severe liver disease	20,477 (0.7%)	17,334 (0.7%)	2,513 (0.6%)	630 (0.6%)	6,755 (2.3%)	5,713 (2.3%)	844 (2.1%)	198 (1.9%)
Diabetes without chronic complications	167,616 (5.6%)	139,424 (5.5%)	22,829 (5.6%)	5,363 (5.5%)	26,535 (8.9%)	21,978 (8.8%)	3,662 (9.1%)	895 (8.6%)
Diabetes with chronic complications	177,234 (5.9%)	149,573 (6.0%)	21,992 (5.4%)	5,669 (5.8%)	44,891 (15%)	37,827 (15%)	5,616 (14%)	1,448 (14%)
Hemiplegia or paraplegia	20,927 (0.7%)	17,789 (0.7%)	2,493 (0.6%)	645 (0.7%)	7,662 (2.6%)	6,629 (2.7%)	794 (2.0%)	239 (2.3%)
Renal disease	242,623 (8.0%)	205,144 (8.2%)	29,906 (7.3%)	7,573 (7.8%)	68,026 (23%)	57,786 (23%)	8,115 (20%)	2,125 (20%)
Any malignancy except neoplasm of skin	180,441 (6.0%)	154,949 (6.2%)	19,811 (4.8%)	5,681 (5.8%)	30,402 (10%)	25,557 (10%)	3,803 (9.4%)	1,042 (10%)
Metastatic solid tumor	27,375 (0.9%)	23,364 (0.9%)	3,137 (0.8%)	874 (0.9%)	7,333 (2.4%)	6,133 (2.5%)	945 (2.3%)	255 (2.4%)
HIV	11,436 (0.4%)	10,634 (0.4%)	678 (0.2%)	124 (0.1%)	1,915 (0.6%)	1,796 (0.7%)	<120^c^	<20^c^
Hypertension	780,064 (26%)	659,478 (26%)	96,091 (24%)	24,495 (25%)	130,002 (43%)	109,516 (44%)	16,320 (40%)	4,166 (40%)
Obesity	822,095 (27%)	701,480 (28%)	95,020 (23%)	25,595 (26%)	100,406 (34%)	85,066 (34%)	12,035 (30%)	3,305 (32%)
Former or current tobacco user	135,531 (4.5%)	110,051 (4.4%)	20,380 (5.0%)	5,100 (5.2%)	27,322 (9.1%)	22,378 (9.0%)	3,915 (9.7%)	1,029 (9.9%)
History of substance abuse disorder	80,649 (2.7%)	68,640 (2.7%)	9,731 (2.4%)	2,278 (2.3%)	20,767 (6.9%)	18,064 (7.3%)	2,159 (5.4%)	544 (5.2%)
US Census region								
Midwest	1,422,648 (47%)	1,090,752 (43%)	272,832 (67%)	59,064 (61%)	115,990 (39%)	89,536 (36%)	21,442 (53%)	5,012 (48%)
Northeast	370,570 (12%)	330,642 (13%)	23,948 (5.9%)	15,980 (16%)	51,533 (17%)	48,258 (19%)	2,012 (5.0%)	1,263 (12%)
South	802,396 (27%)	683,970 (27%)	98,546 (24%)	19,880 (20%)	94,976 (32%)	76,201 (31%)	15,172 (38%)	3,603 (35%)
West	423,032 (14%)	407,078 (16%)	13,254 (3.2%)	2,700 (2.8%)	37,014 (12%)	34,775 (14%)	1,704 (4.2%)	535 (5.1%)
COVID‐19 hospitalization (−3/+14 days within diagnosis)	299,513 (9.9%)	248,770 (9.9%)	40,330 (9.9%)	10,413 (11%)	N/A	N/A	N/A	N/A
Adverse inpatient events within 45 days								
AKI/dialysis	N/A	N/A	N/A	N/A	65,311 (22%)	55,092 (22%)	8,025 (20%)	2,194 (21%)
MACE	N/A	N/A	N/A	N/A	16,158 (5.4%)	13,458 (5.4%)	2,115 (5.2%)	585 (5.6%)
ECMO/invasive mechanical ventilation	N/A	N/A	N/A	N/A	22,718 (7.6%)	18,060 (7.3%)	3,570 (8.9%)	1,088 (10%)
Inpatient death	N/A	N/A	N/A	N/A	28,181 (9.4%)	22,253 (8.9%)	4,635 (11%)	1,293 (12%)
Number of visits before COVID‐19, Median (IQR)	13 (3‐40)	13 (3‐40)	12 (3‐37)	12 (2‐41)	15 (2‐51)	15 (2‐52)	14 (2‐47)	13 (1‐47)
Observation period before COVID‐19, Median (IQR)	915 (126‐1,326)	921 (126‐1,330)	883 (128‐1,306)	883 (102‐1,313)	865 (18‐1,300)	866 (19‐1,299)	872 (19‐1,306)	802 (0‐1,296)
Number of visits after COVID‐19, Median (IQR)	5 (1‐15)	5 (1‐15)	5 (1‐15)	5 (1‐16)	6 (1‐21)	6 (1‐21)	6 (1‐20)	6 (1‐20)
Observation period after COVID‐19, Median (IQR)	151 (0‐356)	148 (0‐351)	168 (0‐375)	166 (0‐377)	145 (0‐384)	145 (0‐384)	150 (0‐386)	144 (0‐386)

^a^ Median (IQR); n (%).

^b^ Computable definitions for all exposures are available in Methods S3.

^c^ Small cell counts representing <20 patients and any adjacent cells that allow back‐calculation of small cell counts are obfuscated per N3C policies.

### Adverse events and COVID‐19 therapies over time

**FIGURE 3:**
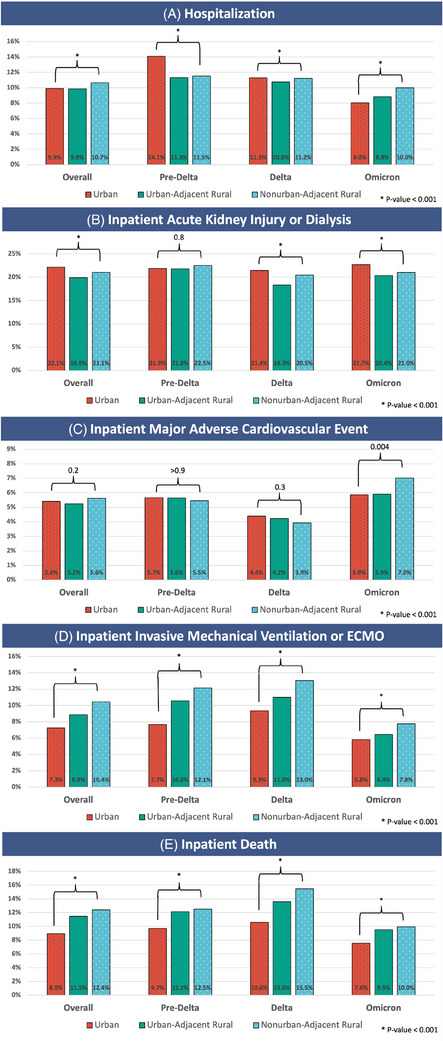
Adverse events by rurality over time. Figure 3 documents rates of adverse events across the overall observation period and during the pre‐Delta, Delta, and Omicron variant‐dominant epochs for urban, urban‐adjacent rural, and nonurban‐adjacent rural dwellers. The statistics presented are Pearson's Chi‐squared test.

Figure 3 reports the rate of adverse events by rurality overall and during individual epochs. Inpatient death and inpatient IMV or ECMO increased significantly along a gradient risk by rurality from urban to UAR to NAR. This trend was not seen in hospitalization or inpatient AKI/dialysis. Inpatient MACE events did not show a rurality trend except during Omicron, where higher MACE adverse events were noted among NAR dwellers.

Figure 4 shows the distribution of COVID‐19 therapy use in the overall cohort and stratified by rurality over time. Across all epochs (Table S1), rural dwellers had statistically higher rates of spike mAB treatment. In comparison, urban dwellers were more often prescribed nirmatrelvir/ritonavir, molnupiravir, anticoagulants, dexamethasone, and remdesivir but not tocilizumab (*P* = .069). These trends held in those hospitalized.

FIGURE 4COVID‐19 therapy usage over time in study cohort. (A) All COVID‐19 therapies per 1,000 person visit days, January 2021‐December 2022. (B) Individual COVID‐19 therapies per 1,000 person visit days, January 2021‐December 2022. (C) COVID‐19 therapies per 1,000 person visit days by rurality, January 2021‐December 2022. COVID‐19 therapy delivery rates among the study cohort across the observation period (January 2021‐March 2023) among those with a SARS‐CoV‐2 infection between January 2021 and December 2022. Figure 4A includes all COVID‐19 therapies for all patients. Figure 4B includes individual COVID‐19 therapies by binary rural‐dwelling status. Figure 4C includes individual COVID‐19 therapies over the observation period among all patients. The date on the X axis reflects dates medications are documented in N3C, while COVID‐19 epochs (pre‐Delta, Delta, and Omicron) designations in Figure 4A reflect dates of patient SARS‐CoV‐2 infection, while 4B and 4C reflect treatment exposures per 1,000 person visit days. The availability of medications was not universal across all observation periods. Full details about medication availability are available in Table S1.

**Figure:**
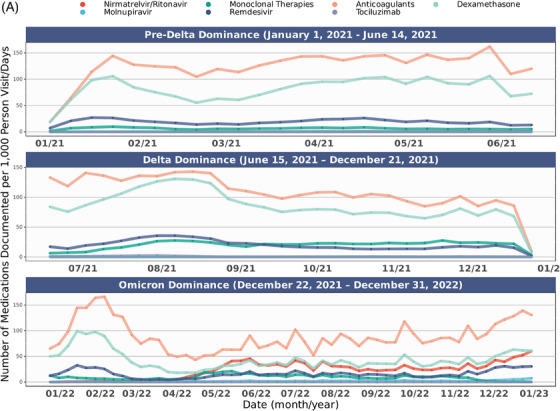

**Figure:**
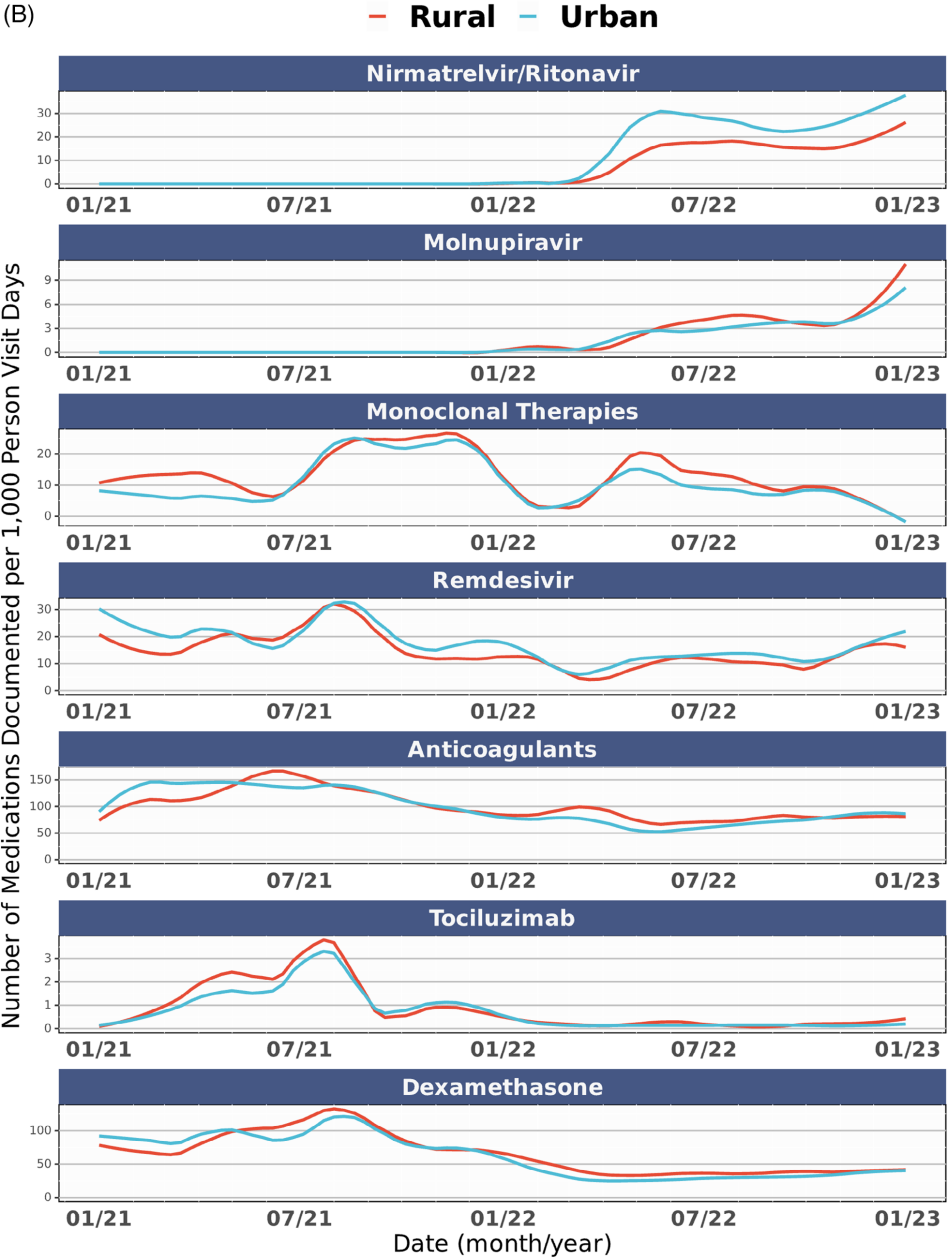

**Figure:**
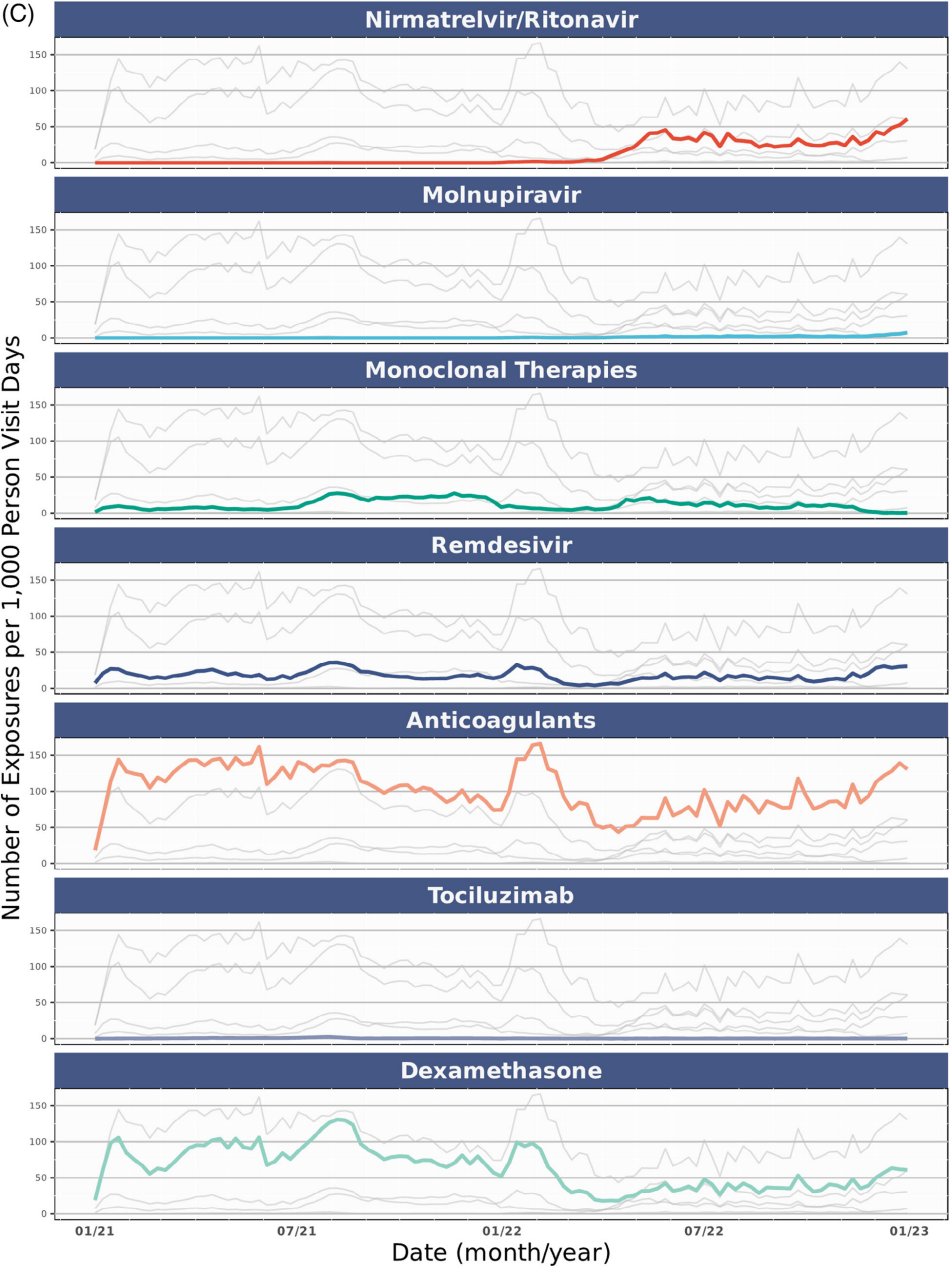

### KM survival estimates

KM survival estimates demonstrate significantly higher 45‐day inpatient mortality among UAR and NAR rural patients compared to their urban counterparts (log‐rank *P* <.001) across all variant epochs (Figure 5A). A gradient risk was observed in survival probability based on the degree of rurality (91% urban, 88% UAR, and 87% NAR). Significant survival differences were observed across epochs (Figure 5B), with the highest mortality observed during Delta, followed by pre‐Delta and Omicron (*P* <.001). In each COVID‐19 epoch, urban dwellers had a higher probability of survival (Figure 5C).

FIGURE 5Kaplan‐Meier 45‐day survival estimates by rurality and COVID‐19 epoch. (A) Rurality across all variant epochs. (B) COVID‐19 epochs. (C) Rurality stratified by COVID‐19 epochs. Kaplan‐Meier survival 45‐day survival curves based on (A) rurality, (B) COVID‐19 epoch, and (C) rurality stratified by COVID‐19 epoch.

**Figure:**
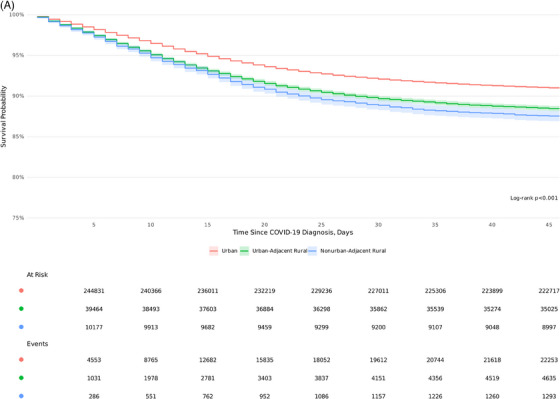

**Figure:**
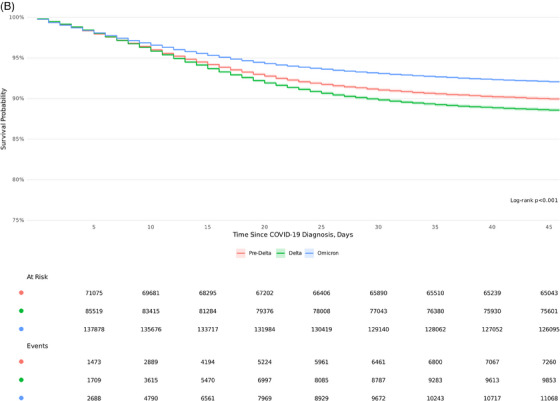

**Figure:**
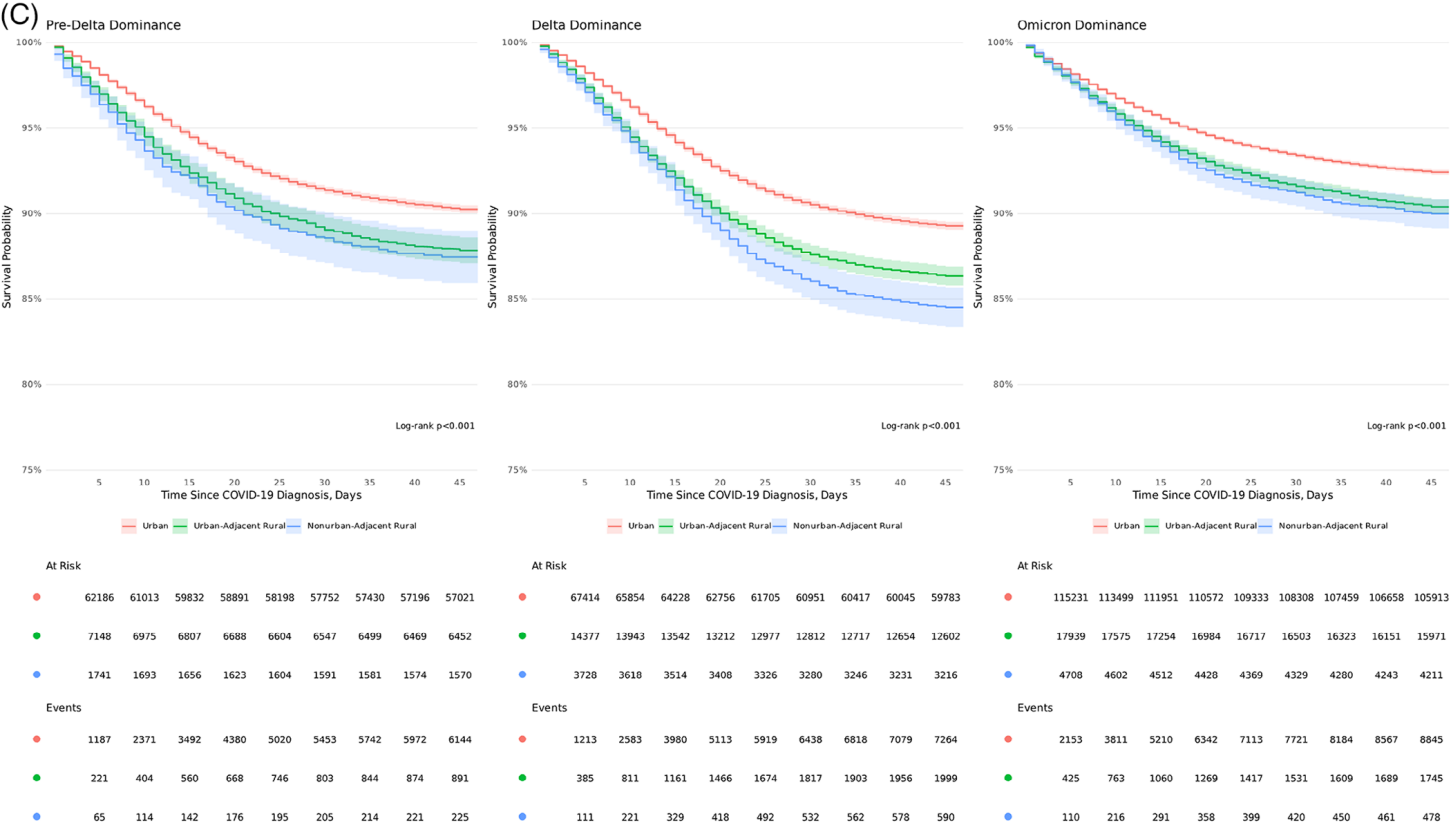

### Multivariable‐adjusted odds and hazard ratios for adverse acute COVID‐19 events

**FIGURE 6:**
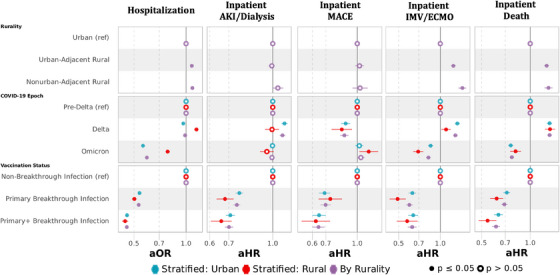
Multivariable logistic regression and Cox proportional hazards regression for adverse acute COVID‐19 events by rurality. Multivariable logistic regression for hospitalization and multivariable Cox proportional hazards for inpatient acute kidney injury (AKI) or dialysis, major adverse cardiovascular event (MACE), invasive mechanical ventilation (IMV) or extracorporeal membrane oxygenation (ECMO), and death. All models adjusted for sex, age, race/ethnicity, myocardial infarction, congestive heart failure, peripheral vascular disease, cerebrovascular disease, dementia, chronic pulmonary disease, rheumatologic disease, peptic ulcer disease, liver disease, diabetes mellitus, hemiplegia or paraplegia, renal disease, cancer, HIV, obesity, hypertension, smoking status, substance abuse disorder, variant period, vaccination status (completion of primary vaccination series or primary vaccination series + additional dose(s) before SARS‐CoV‐2 infection), and US Census region. Figure 6 contains 3 models for each outcome: models in purple include rural and urban dwellers, while models in blue and red are stratified into urban and rural dwellers, respectively. Full model specifications are available in Table S2.

Adjusting for age, sex, race/ethnicity, comorbid conditions, Census region, vaccination status, tobacco usage, and substance abuse disorder (Figure 6; models are specified in Table S2), rural dwellers had higher odds of hospitalization during the entire observation period (adjusted odds ratio [aOR] 1.06 [95% confidence interval 1.05‐1.08] UAR and 1.06 [1.04‐1.08] NAR relative urban). The disparity in hospitalizations among rural compared with urban populations worsened across variant epochs, from pre‐Delta (aOR 0.90 [0.88‐0.93] UAR and 0.90 [0.85‐0.94] NAR relative to urban) to Delta (1.04 [1.02‐1.06] UAR and 1.03 [1.00‐1.07] NAR) to Omicron (aOR 1.15 [1.13‐1.17] UAR and 1.16 [1.13‐1.20] NAR relative to urban). Among those hospitalized, rural dwellers had higher risks across all epochs for all outcomes: AKI/dialysis (adjusted hazard ratio [aHR] 1.04 [1.02‐1.07] UAR and 1.09 [1.04‐1.14]), MACE (aHR 1.08 [1.03‐1.13] UAR and 1.08 [1.00‐1.18] NAR), ECMO/IMV (aHR 1.33 [1.28‐1.38] UAR and 1.56 [1.47‐1.66]), and death (aHR 1.28 [1.24‐1.32] UAR and 1.35 [1.28‐1.43] NAR).

While the rates of adverse events decreased over time in all cohorts, the relative risk of adverse events across the COVID‐19 epochs did not change. In pre‐Delta, UAR had a 31% (aHR 1.31 [1.22‐1.41]) and NAR had a 35% (aHR 1.35 [1.18‐1.54]) higher risk of inpatient death than urban, which narrowed in the Delta to 29% (aHR 1.29 [1.22‐1.36]) for UAR and increased to 42% (aHR 1.42 [1.31‐1.55]) for NAR and narrowed slightly for the Omicron to 25% (aHR 1.25 [1.18‐1.31]) for UAR and 28% (aHR 1.28 [1.17‐1.40]) for NAR. Similar trends occurred in other adverse events across epochs (Table S2). Adjusted models (Table S3) demonstrate that underlying risk factors attenuated some, but not most, of the rurality effect.

Documented vaccination before SARS‐CoV‐2 infection status dramatically reduced the odds of hospitalization, with completion of a primary vaccination series (aOR 0.56 [0.55‐0.56]) having less protection than completion of a booster series (0.47 [0.47‐0.48]) relative to those without documented vaccination before infection. Similar trends were observed among those hospitalized, with a lower risk of death in those completing a primary (aHR 0.70 [0.67‐0.73]) and booster series (aHR 0.621 [0.58‐0.65]) relative to those without documented vaccination. In models stratified by rurality (Figure 6 and Table S4), vaccination status was similarly protective in rural and urban dwellers.

In models adjusted for medication exposure between COVID‐19 diagnosis and (1) AIEs, (2) discharge, or (3) censor or end of follow‐up (Figure 7, Tables S5 and S6), odds of hospitalization were similar as models not accounting for nirmatrelvir/ritonavir, molnupiravir, and mABs (aOR 1.07 [1.05‐1.08] UAR and 1.06 [1.03‐1.08] NAR). After adjusting for outpatient and inpatient therapies, the risk of AIEs was comparable to models without medications for IMV/ECMO and death.

FIGURE 7Multivariable regression models for hospitalization and adverse inpatient events with targeted COVID‐19 therapies across all variant epochs. (A) Multivariable logistic regression for hospitalization and Cox proportional hazards regression for inpatient AKI/dialysis or MACE with targeted COVID‐19 therapies. (B) Cox proportional hazards regression for inpatient IMV/ECMO, death, or death after mechanical ventilation with targeted COVID‐19 therapies. Multivariable logistic regression for hospitalization and multivariable Cox proportional hazards for inpatient acute kidney injury (AKI) or dialysis, major adverse cardiovascular event (MACE), invasive mechanical ventilation (IMV) or extracorporeal membrane oxygenation (ECMO), and death. All models adjusted for sex, age, race/ethnicity, myocardial infarction, congestive heart failure, peripheral vascular disease, cerebrovascular disease, dementia, chronic pulmonary disease, rheumatologic disease, peptic ulcer disease, liver disease, diabetes mellitus, hemiplegia or paraplegia, renal disease, cancer, HIV, obesity, hypertension, smoking status, substance abuse disorder, US Census region, vaccination status (completion of primary vaccination series or primary vaccination series + additional dose(s) before SARS‐CoV‐2 infection), nirmatrelvir/ritonavir, molnupiravir, monoclonal therapies, remdesivir, anticoagulants, tocilizumab, and dexamethasone. Figure 6 contains 3 models for each outcome: models in purple include rural and urban dwellers, while models in blue and red are stratified into urban and rural dwellers, respectively. Full model specifications are available in Tables S5 and S6.

**Figure:**
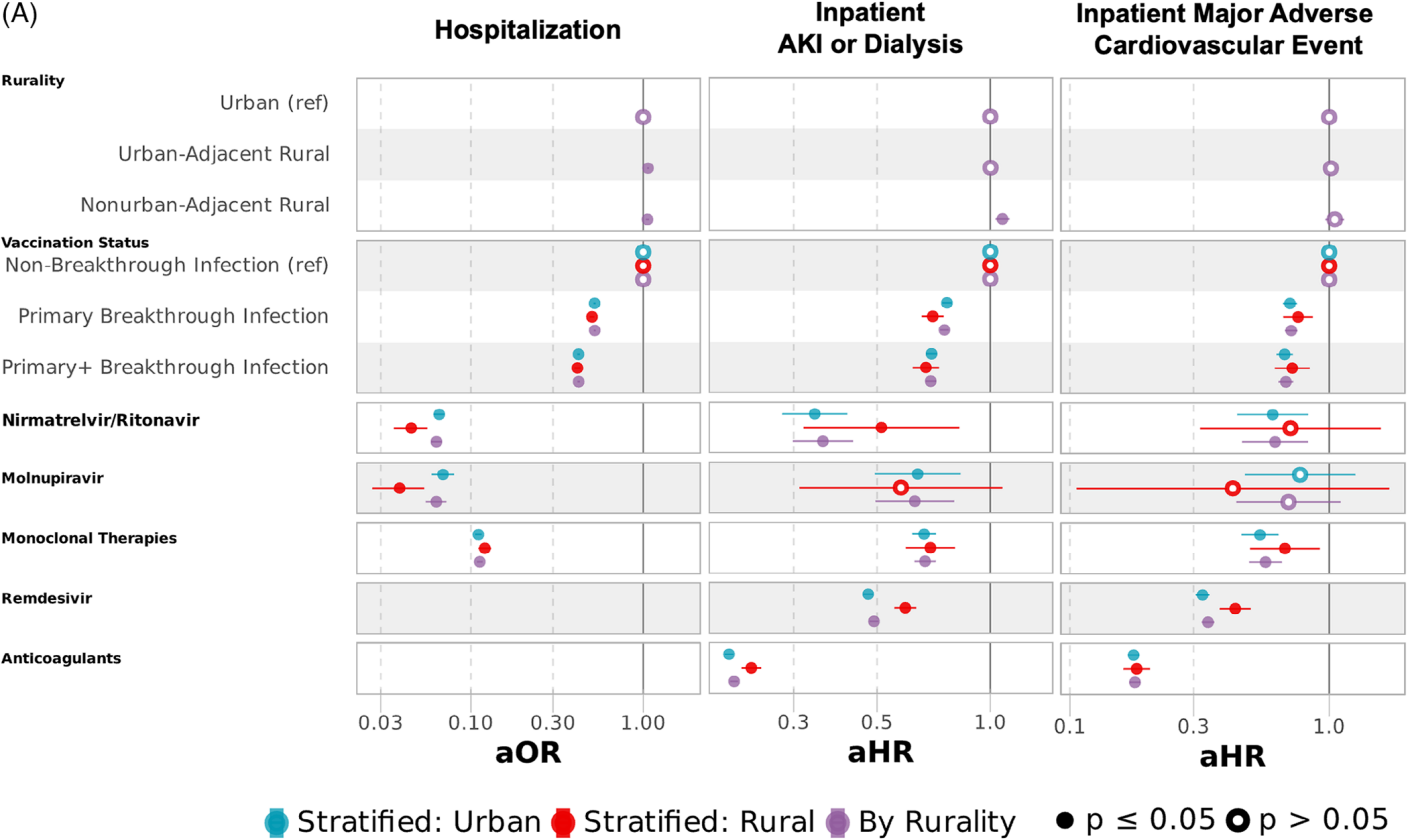

**Figure:**
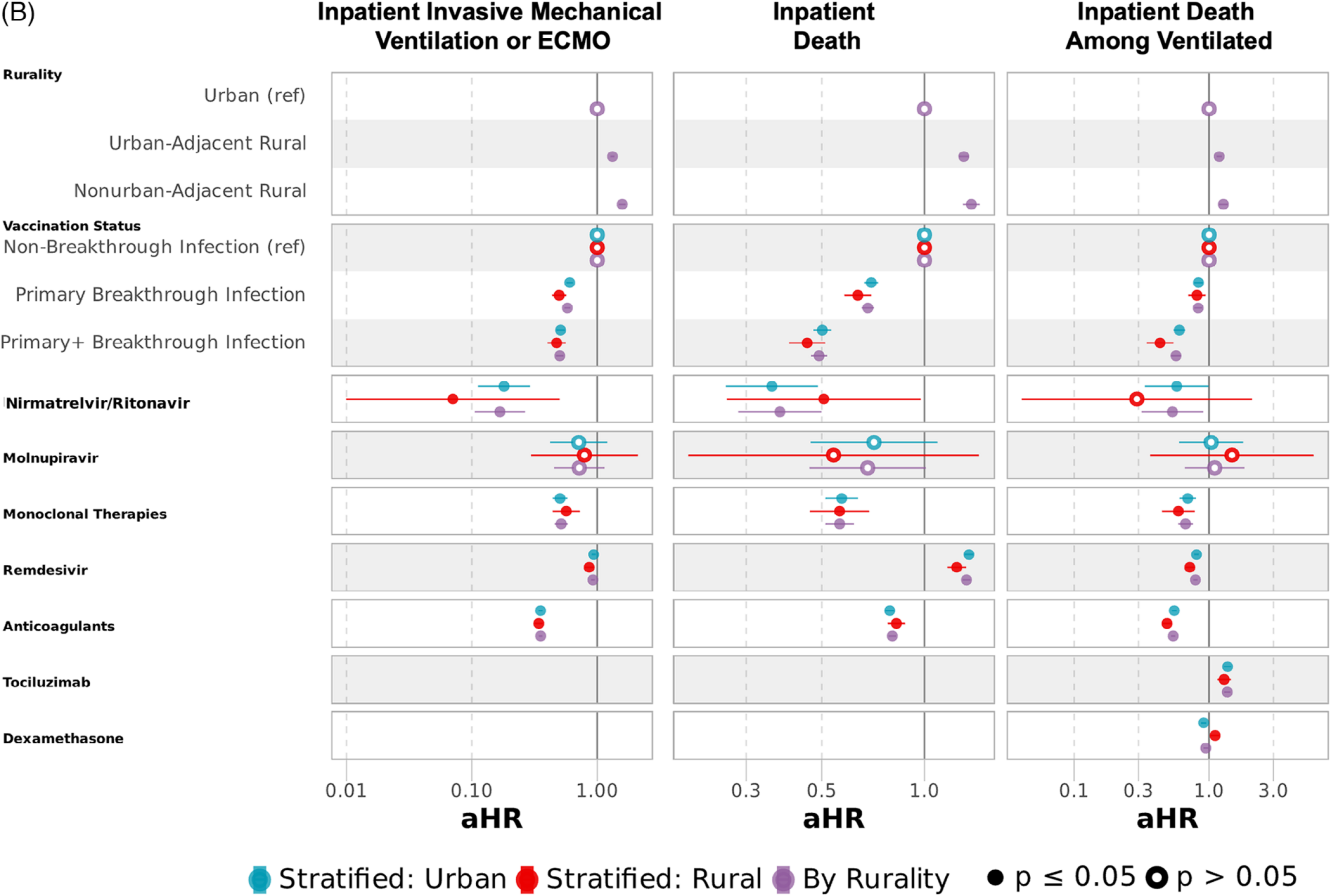

Medical therapies reduced the risk of AKI/dialysis and MACE for UAR but not NAR compared to urban dwellers when compared to models without medication adjustment (AKI/dialysis: aHR 1.00 [0.98‐1.03] UAR and 1.08 [1.03‐1.13] NAR); MACE: aHR 1.02 [0.97‐1.06] UAR and 1.05 [0.97‐1.14] NAR). Risk among rural relative to urban dwellers was higher and consistent with models not accounting for medication for IMV/ECMO (aHR 1.32 [1.27‐1.37] UAR and 1.58 [1.49‐1.68] NAR) and death (aHR 1.30 [1.26‐1.35] UAR and 1.37 [1.30‐1.45] NAR). Among those who received mechanical ventilation, we adjusted for all medications, including tocilizumab and dexamethasone. Among rural compared with urban dwellers, the adjusted risk of inpatient death among those with IMV/ECMO, with additional adjustments for tocilizumab and dexamethasone, was 19% higher (aHR 1.19 [1.14‐1.24]) in UAR and 28% higher (aHR 1.28 [1.18‐1.38]) in NAR.

Across all epochs in combined models, those with documented nirmatrelvir/ritonavir (aOR 0.06 [0.06‐0.07]), molnupiravir (aOR 0.06 [0.05‐0.07]), and mABs (aOR 0.11 [0.11‐0.12]) in outpatient settings experienced significantly lower adjusted odds of hospitalization. Nirmatrelvir/ritonavir, monoclonal therapies, and anticoagulants lowered the adjusted risk of all inpatient adverse events, notably death (aHR 0.38 [0.28‐0.50] nirmatrelvir/ritonavir, 0.56 [0.51‐0.62] mABs, and 0.69 [0.67‐0.71] anticoagulants). Molnupiravir lowered the risk of AKI/dialysis but had no association with reduced risk of MACE, IMV/ECMO, or death. Remdesivir had lower risk of AKI/dialysis, MACE, and IMV/ECMO, but not death. Among those with IMV/ECMO, remdesivir and dexamethasone had a lower risk of inpatient death (aHR 0.79 [0.76‐0.82] remdesivir and 0.95 [0.91‐0.98] dexamethasone), while tocilizumab had a higher risk of death (aHR 1.36 [1.29‐1.44]). Similar adjusted odds and risk were observed in models stratified by rurality for most observed medication, except for dexamethasone administered after IMV/ECMO, which was protective in urban (aHR 0.91 [0.87‐0.95]) but not rural (aHR 1.11 [1.02‐1.21]) dwellers.

### Propensity‐score matched results

After PSM with exceptional covariate balance (absolute standardized mean differences <0.1; covariate balance plot available in Figure S3), a total of 990,106 patients were included, of whom 495,053 (50%) were urban, 402,118 (41%) were UAR, and 92,935 (9.4%) were NAR dwellers. Baseline characteristics of the PSM cohort are shown in Table S7. The primary and secondary analyses were repeated in this cohort. KM survival estimates demonstrate significantly higher mortality 45 days after hospitalization among UAR and NAR rural patients compared to their urban counterparts (log‐rank *P* <.001) across all variant epochs (Figure S4A), within each COVID‐19 epoch (Figure S4B), and stratified by rurality in each COVID‐19 epoch (Figure S4C). Adjusted odds of hospitalization were higher after PSM (aOR 1.10 [1.08‐1.12] UAR and 1.15 [1.12‐1.18] NAR relative urban), while AIEs were comparable (Table S8). Similar findings were observed in models stratified by rurality (Table S9), with COVID‐19 therapies included (Table S10), and with COVID‐19 therapies after rural stratification (Table S11).

### Sensitivity analyses

SAs evaluated the robustness of models under altered conditions. Using a binary rather than a multilevel rurality indicator (SA‐1), model estimates were similar throughout analyses (Table S12). To account for potential referral‐in bias leading to more severe disease progression at admission (SA‐2 and SA‐3), we restricted analyses to patients who did not die in the first 3 days after COVID‐19 diagnosis (Table S13) and with an additional covariate for pre‐COVID‐19 visit history (Table S14), neither of which changed relative associations between rurality and adverse events. When evaluating drugs individually during dates when they were widely in use or under FDA EUA (SA‐4), the individual benefit of several medications was attenuated compared to combined models (Tables S15 and S16). Still, it was similar between urban and rural dwellers.

## DISCUSSION

This study leverages real‐world data (RWD) from a nationally sampled US cohort to examine disparities in adverse COVID‐19 events between urban and rural populations. These disparities persist even after accounting for treatment and vaccination rate variations. We find that rural dwellers face higher odds of hospitalization and higher risk of inpatient death, and other AIEs, and these disparities have widened notably after the availability of COVID‐19 vaccines and the emergence of the Delta and Omicron variants. To our knowledge, this is the most extensive study that uses RWD to assess how COVID‐19 treatments and vaccinations affect patient outcomes in rural and urban contexts within the United States.

After adjusting for age, sex, race/ethnicity, Census region, and underlying comorbidities, our findings indicate a considerably elevated risk of post‐hospitalization death among rural residents, at rates 43% higher in UAR and 48% higher in NAR than their urban counterparts. Furthermore, the data reveal that rural patients are more likely to require AKI/dialysis and IMV/ECMO following hospitalization for COVID‐19. Such interventions precipitate increased long‐term health challenges like post‐traumatic stress disorder, depression, anxiety, and physical deconditioning.^33^, ^34^ The need for extended post‐treatment rehabilitation among survivors could exacerbate existing pressures on already‐stressed health care resources in rural settings.^35^

The FDA has authorized several COVID‐19 treatments, including antiviral medications (eg, antiviral drugs remdesivir,^36^ molnupiravir,^37^ and nirmatrelvir/ritonavir^38^) and mABs spike (eg, REGEN‐COV^39^ and bamlanivimab).^40^ Corticosteroids, primarily dexamethasone, received recommendations for treating COVID‐19 from the American Medical Association and World Health Organization in Fall 2020.^41^ Treatment recommendations have evolved and varied depending on disease severity. Nirmatrelvir/ritonavir, molnupiravir, and mABs targeting the SARS‐CoV‐2 spike protein are generally advised for outpatients. At the same time, remdesivir and anticoagulants are reserved for inpatient care, and tocilizumab and dexamethasone are advised for hypoxic patients.^42^, ^43^, ^44^, ^45^ However, the literature remains scant on how these therapeutic advancements have directly benefited rural communities.

Geographic disparities in oral antiviral availability and prescribing are notable nationwide. In high social vulnerability zip codes, prescribing rates are only half compared to medium or low social vulnerability areas.^46^ Through June 2022, 98% of US residents had access to oral antiviral treatments in their counties.^47^ However, this availability is not uniformly distributed; 28% of rural counties lacked such a facility. The number of treatment courses was generally lower in rural counties, exacerbated by the already longer travel distance to health care facilities in rural settings.^48^ Our findings showed a significant disparity in nirmatrelvir/ritonavir prescribing and administration in rural areas but not for molnupiravir, which had similar rates among rural and urban populations. Both antiviral agents were associated with greater protection against hospitalization in rural compared with urban areas. Given the increased hurdles that rural dwellers must overcome to obtain these medications, those rural dwellers who do obtain them may have increased health‐seeking behaviors.

Despite health care infrastructure disparities that may limit treatment availability, our study shows that except for nirmatrelvir/ritonavir, prescribing and administration rates of other COVID‐19 medications were generally similar for rural and urban populations. Our data further indicate that therapeutic agents provided similar protection among urban and rural populations for key clinical adverse outcomes, including acute COVID‐19 hospitalization, inpatient MACE, IMV/ECMO, and death. Intriguingly, although dexamethasone has proven effective in treating acute hypoxemic respiratory failure stemming from COVID‐19,^45^ its benefits appeared to be less pronounced among rural dwellers (aHR 1.11 [1.02‐1.21]) as compared to urban dwellers (aHR 0.91 [0.87‐0.95]).

Our study reveals differences in pre‐infection vaccination rates between rural and urban populations, with 23% of UAR and 18% of NAR having a breakthrough SARS‐CoV‐2 infection versus 25% of urban dwellers. Despite these disparities, when adjusting for rurality in our models and in those models stratified by rurality, vaccines conferred similar levels of protection against hospitalization and adverse events in both demographic groups. However, greater protection was observed in individuals who had completed their primary vaccination series and received at least 1 booster dose. Through March 15, 2023, rural dwellers have lower rates of primary vaccination series (55.2% rural, 66.5% urban) and updated bivalent booster doses (11.5% rural, 18.0% urban),^16^ with rates lower in more rural areas, areas with populations having lower educational attainment, and counties dependent on farming and mining.^49^

Our study reveals a critical insight: despite similar benefits observed with COVID‐19 treatments and vaccination among rural and urban populations, there remains a pronounced disparity in adverse events. This suggests that the observed rural penalty is not a function of treatment efficacy or vaccination status but likely stems from deeper, systemic factors that disproportionately affect rural communities. This increased vulnerability can be attributed to a confluence of factors: higher rates of underlying health conditions,^50^ reduced access to—and willingness to utilize—preventive measures like vaccines,^51^, ^52^, ^53^ lower levels of community resilience,^54^ and overarching socioeconomic inequalities in health care.^6^ This study found that even after accounting for differences in therapeutic interventions, SARS‐CoV‐2 vaccination, demographic differences, and comorbid burden, rurality is still associated with higher odds of hospitalization and risk for adverse inpatient events. The compelling question is why the disparities persist.

Ensuring equitable access to specialty care is crucial, yet persistent disparities exist between rural and urban settings in the United States. To bridge this gap, a comprehensive and multifaceted approach is essential. Rural disparities in health continue to expand, with studies pointing to structural,^55^ environmental, socioeconomic, cultural, behavioral, and health care access inequities. Addressing these disparities will necessitate a multidisciplinary approach that extends beyond medical treatment to include societal and systemic interventions. Providers and public health officials should consider the wider social and structural context in which their patients live, as these significantly contribute to the risk and outcomes of COVID‐19.

### Limitations

This study has several limitations. N3C contains RWD from sites across the United States, with most contributors coming from tertiary care centers. As a result, more rural than urban dwellers may have presented to N3C‐contributing institutions with more severe COVID‐19 illness. However, we observed similar visits and observation histories across rural and urban dwellers, suggesting that most of our cohort, regardless of rurality, receives regular care at the site reporting to N3C. As reported previously,^7^, ^11^ vaccination capture in EHR data is notoriously underreported, as is data reporting across contributing sites based on the maturity of source clinical research data management. We have systematically selected a subset of participating organizations to mitigate potential site reporting bias and adjusted for site throughout our analysis. Still, there is potential misclassification of comorbid conditions and vaccinations across our cohort. However, no evidence exists that the sites systematically differ in reporting vaccination status between urban and rural patients. The vaccination data presented in this study represent breakthrough SARS‐CoV‐2 infections rather than vaccination rates among our cohorts. Further, our goal was not to determine the absolute number of vaccinated individuals but to look at trends and associations that we feel are accurately reflected in the data.

While we have made every effort to consider baseline differences between rural and urban dwellers, N3C reporting sites do not routinely provide information on admitting or referral status, so prior care‐seeking behavior or treatments administered are unavailable in this analysis. Finally, this study compares the relative effectiveness of recommended COVID‐19 therapies between urban and rural dwellers. Our study design and findings are not optimized for identifying optimal therapies but for establishing the relative benefits based on rurality.

## CONCLUSIONS

This retrospective cohort study using RWD highlights persistent COVID‐19 disparities between rural and urban areas. Although targeted therapies and vaccinations are equally effective in both settings, rural regions face higher hospitalization and mortality rates as the pandemic persists into its fourth year. These findings emphasize the urgent need to strengthen rural health care infrastructure and expand public health campaigns. Immediate efforts should focus on enhancing vaccination drives and increasing access to comprehensive health care services, including post‐acute care rehabilitation, to mitigate ongoing disparities. The critical unresolved issue remains: Why do rural communities continue to experience higher rates of hospitalization, mortality, and adverse events related to acute COVID‐19? Further research is required to address this question.

## REVIEW BOARD APPROVALS AND CONSENT TO PARTICIPATE

National Institute of Health's (NIH) National COVID Cohort Collaborative (N3C) Data Utilization Request Approval committee approved the data utilization request of this project (RP‐504BA5), which is approved under the authority of the National Institutes of Health Institutional Review Board and with Johns Hopkins University School of Medicine serving as a central institutional review board. The study protocol was obtained from the University of Mississippi Medical Center (IRB2020V0280, 3/31/2021), Johns Hopkins University (IRB00249128, 9/18/2020), Christiana Health (IRB604959, 5/07/2021), West Virginia University (IRB2012192778, 12/17/2020), University of Nebraska Medical Center (IRB050‐21‐EP, 2/9/2021), Nemour's Children's Health (IRB1700991, 2/17/2022), and Maine Medical Center (IRB1697848‐2, 3/5/2021). The N3C data transfer to NCATS is performed under a Johns Hopkins University Reliance Protocol # IRB00249128 or individual site agreements with NIH. The N3C Data Enclave is managed under the authority of the NIH; information can be found at https://ncats.nih.gov/n3c/resources. No informed consent was obtained because the study used a limited data set.

## N3C ATTRIBUTION

The analyses described in this publication were conducted with data or tools accessed through the NCATS N3C Data Enclave covid.cd2h.org/enclave and supported by NCATS U24 TR002306. This research was possible because of the patients whose information is included within the data from participating organizations (covid.cd2h.org/dtas) and the organizations and scientists (covid.cd2h.org/duas) who have contributed to the ongoing development of this community resource.

## INDIVIDUAL ACKNOWLEDGMENTS FOR CORE CONTRIBUTORS

We gratefully acknowledge the following core contributors to N3C:

Adam B. Wilcox, Adam M. Lee, Alexis Graves, Alfred (Jerrod) Anzalone, Amin Manna, Amit Saha, Amy Olex, Andrea Zhou, Andrew E. Williams, Andrew Southerland, Andrew T. Girvin, Anita Walden, Anjali A. Sharathkumar, Benjamin Amor, Benjamin Bates, Brian Hendricks, Brijesh Patel, Caleb Alexander, Carolyn Bramante, Cavin Ward‐Caviness, Charisse Madlock‐Brown, Christine Suver, Christopher Chute, Christopher Dillon, Chunlei Wu, Clare Schmitt, Cliff Takemoto, Dan Housman, Davera Gabriel, David A. Eichmann, Diego Mazzotti, Don Brown, Eilis Boudreau, Elaine Hill, Elizabeth Zampino, Emily Carlson Marti, Emily R. Pfaff, Evan French, Farrukh M Koraishy, Federico Mariona, Fred Prior, George Sokos, Greg Martin, Harold Lehmann, Heidi Spratt, Hemalkumar Mehta, Hongfang Liu, Hythem Sidky, J.W. Awori Hayanga, Jami Pincavitch, Jaylyn Clark, Jeremy Richard Harper, Jessica Islam, Jin Ge, Joel Gagnier, Joel H. Saltz, Joel Saltz, Johanna Loomba, John Buse, Jomol Mathew, Joni L. Rutter, Julie A. McMurry, Justin Guinney, Justin Starren, Karen Crowley, Katie Rebecca Bradwell, Kellie M. Walters, Ken Wilkins, Kenneth R. Gersing, Kenrick Dwain Cato, Kimberly Murray, Kristin Kostka, Lavance Northington, Lee Allan Pyles, Leonie Misquitta, Lesley Cottrell, Lili Portilla, Mariam Deacy, Mark M. Bissell, Marshall Clark, Mary Emmett, Mary Morrison Saltz, Matvey B. Palchuk, Melissa A. Haendel, Meredith Adams, Meredith Temple‐O'Connor, Michael G. Kurilla, Michele Morris, Nabeel Qureshi, Nasia Safdar, Nicole Garbarini, Noha Sharafeldin, Ofer Sadan, Patricia A. Francis, Penny Wung Burgoon, Peter Robinson, Philip R.O. Payne, Rafael Fuentes, Randeep Jawa, Rebecca Erwin‐Cohen, Rena Patel, Richard A. Moffitt, Richard L. Zhu, Rishi Kamaleswaran, Robert Hurley, Robert T. Miller, Saiju Pyarajan, Sam G. Michael, Samuel Bozzette, Sandeep Mallipattu, Satyanarayana Vedula, Scott Chapman, Shawn T. O'Neil, Soko Setoguchi, Stephanie S. Hong, Steve Johnson, Tellen D. Bennett, Tiffany Callahan, Umit Topaloglu, Usman Sheikh, Valery Gordon, Vignesh Subbian, Warren A. Kibbe, Wenndy Hernandez, Will Beasley, Will Cooper, William Hillegass, Xiaohan Tanner Zhang. Details of contributions are available at covid.cd2h.org/core‐contributors.

## DATA PARTNERS WITH RELEASED DATA

The following institutions whose data are released or pending:

Available: Advocate Health Care Network—UL1TR002389: The Institute for Translational Medicine (ITM) • Boston University Medical Campus—UL1TR001430: Boston University Clinical and Translational Science Institute • Brown University—U54GM115677: Advance Clinical Translational Research (Advance‐CTR) • Carilion Clinic—UL1TR003015: iTHRIV Integrated Translational health Research Institute of Virginia • Charleston Area Medical Center—U54GM104942: West Virginia Clinical and Translational Science Institute (WVCTSI) • Children's Hospital Colorado—UL1TR002535: Colorado Clinical and Translational Sciences Institute • Columbia University Irving Medical Center—UL1TR001873: Irving Institute for Clinical and Translational Research • Duke University—UL1TR002553: Duke Clinical and Translational Science Institute • George Washington Children's Research Institute—UL1TR001876: Clinical and Translational Science Institute at Children's National (CTSA‐CN) • George Washington University—UL1TR001876: Clinical and Translational Science Institute at Children's National (CTSA‐CN) • Indiana University School of Medicine—UL1TR002529: Indiana Clinical and Translational Science Institute • Johns Hopkins University—UL1TR003098: Johns Hopkins Institute for Clinical and Translational Research • Loyola Medicine—Loyola University Medical Center • Loyola University Medical Center—UL1TR002389: The Institute for Translational Medicine (ITM) • Maine Medical Center—U54GM115516: Northern New England Clinical & Translational Research (NNE‐CTR) Network • Massachusetts General Brigham—UL1TR002541: Harvard Catalyst • Mayo Clinic Rochester—UL1TR002377: Mayo Clinic Center for Clinical and Translational Science (CCaTS) • Medical University of South Carolina—UL1TR001450: South Carolina Clinical & Translational Research Institute (SCTR) • Montefiore Medical Center—UL1TR002556: Institute for Clinical and Translational Research at Einstein and Montefiore • Nemours—U54GM104941: Delaware CTR ACCEL Program • NorthShore University HealthSystem—UL1TR002389: The Institute for Translational Medicine (ITM) • Northwestern University at Chicago—UL1TR001422: Northwestern University Clinical and Translational Science Institute (NUCATS) • OCHIN—INV‐018455: Bill and Melinda Gates Foundation grant to Sage Bionetworks • Oregon Health & Science University—UL1TR002369: Oregon Clinical and Translational Research Institute • Penn State Health Milton S. Hershey Medical Center—UL1TR002014: Penn State Clinical and Translational Science Institute • Rush University Medical Center—UL1TR002389: The Institute for Translational Medicine (ITM) • Rutgers, The State University of New Jersey—UL1TR003017: New Jersey Alliance for Clinical and Translational Science • Stony Brook University—U24TR002306 • The Ohio State University—UL1TR002733: Center for Clinical and Translational Science • The State University of New York at Buffalo—UL1TR001412: Clinical and Translational Science Institute • The University of Chicago—UL1TR002389: The Institute for Translational Medicine (ITM) • The University of Iowa—UL1TR002537: Institute for Clinical and Translational Science • The University of Miami Leonard M. Miller School of Medicine—UL1TR002736: University of Miami Clinical and Translational Science Institute • The University of Michigan at Ann Arbor—UL1TR002240: Michigan Institute for Clinical and Health Research • The University of Texas Health Science Center at Houston—UL1TR003167: Center for Clinical and Translational Sciences (CCTS) • The University of Texas Medical Branch at Galveston—UL1TR001439: The Institute for Translational Sciences • The University of Utah—UL1TR002538: Uhealth Center for Clinical and Translational Science • Tufts Medical Center—UL1TR002544: Tufts Clinical and Translational Science Institute • Tulane University—UL1TR003096: Center for Clinical and Translational Science • University Medical Center New Orleans—U54GM104940: Louisiana Clinical and Translational Science (LA CaTS) Center • University of Alabama at Birmingham—UL1TR003096: Center for Clinical and Translational Science • University of Arkansas for Medical Sciences—UL1TR003107: UAMS Translational Research Institute • University of Cincinnati—UL1TR001425: Center for Clinical and Translational Science and Training • University of Colorado Denver, Anschutz Medical Campus—UL1TR002535: Colorado Clinical and Translational Sciences Institute • University of Illinois at Chicago—UL1TR002003: UIC Center for Clinical and Translational Science • University of Kansas Medical Center—UL1TR002366: Frontiers: University of Kansas Clinical and Translational Science Institute • University of Kentucky—UL1TR001998: UK Center for Clinical and Translational Science • University of Massachusetts Medical School Worcester—UL1TR001453: The UMass Center for Clinical and Translational Science (UMCCTS) • University of Minnesota—UL1TR002494: Clinical and Translational Science Institute • University of Mississippi Medical Center—U54GM115428: Mississippi Center for Clinical and Translational Research (CCTR) • University of Nebraska Medical Center—U54GM115458: Great Plains IDeA‐Clinical & Translational Research • University of North Carolina at Chapel Hill—UL1TR002489: North Carolina Translational and Clinical Science Institute • University of Oklahoma Health Sciences Center—U54GM104938: Oklahoma Clinical and Translational Science Institute (OCTSI) • University of Rochester—UL1TR002001: UR Clinical & Translational Science Institute • University of Southern California—UL1TR001855: The Southern California Clinical and Translational Science Institute (SC CTSI) • University of Vermont—U54GM115516: Northern New England Clinical & Translational Research (NNE‐CTR) Network • University of Virginia—UL1TR003015: iTHRIV Integrated Translational health Research Institute of Virginia • University of Washington—UL1TR002319: Institute of Translational Health Sciences • University of Wisconsin‐Madison—UL1TR002373: UW Institute for Clinical and Translational Research • Vanderbilt University Medical Center—UL1TR002243: Vanderbilt Institute for Clinical and Translational Research • Virginia Commonwealth University—UL1TR002649: C. Kenneth and Dianne Wright Center for Clinical and Translational Research • Wake Forest University Health Sciences—UL1TR001420: Wake Forest Clinical and Translational Science Institute • Washington University in St. Louis—UL1TR002345: Institute of Clinical and Translational Sciences • Weill Medical College of Cornell University—UL1TR002384: Weill Cornell Medicine Clinical and Translational Science Center • West Virginia University—U54GM104942: West Virginia Clinical and Translational Science Institute (WVCTSI)

Submitted: Icahn School of Medicine at Mount Sinai—UL1TR001433: ConduITS Institute for Translational Sciences • The University of Texas Health Science Center at Tyler—UL1TR003167: Center for Clinical and Translational Sciences (CCTS) • University of California, Davis—UL1TR001860: UCDavis Health Clinical and Translational Science Center • University of California, Irvine—UL1TR001414: The UC Irvine Institute for Clinical and Translational Science (ICTS) • University of California, Los Angeles—UL1TR001881: UCLA Clinical Translational Science Institute • University of California, San Diego—UL1TR001442: Altman Clinical and Translational Research Institute • University of California, San Francisco—UL1TR001872: UCSF Clinical and Translational Science Institute

Pending: Arkansas Children's Hospital—UL1TR003107: UAMS Translational Research Institute • Baylor College of Medicine—None (Voluntary) • Children's Hospital of Philadelphia—UL1TR001878: Institute for Translational Medicine and Therapeutics • Cincinnati Children's Hospital Medical Center—UL1TR001425: Center for Clinical and Translational Science and Training • Emory University—UL1TR002378: Georgia Clinical and Translational Science Alliance • HonorHealth—None (Voluntary) • Loyola University Chicago—UL1TR002389: The Institute for Translational Medicine (ITM) • Medical College of Wisconsin—UL1TR001436: Clinical and Translational Science Institute of Southeast Wisconsin • MedStar Health Research Institute—UL1TR001409: The Georgetown‐Howard Universities Center for Clinical and Translational Science (GHUCCTS) • MetroHealth—None (Voluntary) • Montana State University—U54GM115371: American Indian/Alaska Native CTR • NYU Langone Medical Center—UL1TR001445: Langone Health's Clinical and Translational Science Institute • Ochsner Medical Center—U54GM104940: Louisiana Clinical and Translational Science (LA CaTS) Center • Regenstrief Institute—UL1TR002529: Indiana Clinical and Translational Science Institute • Sanford Research—None (Voluntary) • Stanford University—UL1TR003142: Spectrum: The Stanford Center for Clinical and Translational Research and Education • The Rockefeller University—UL1TR001866: Center for Clinical and Translational Science • The Scripps Research Institute—UL1TR002550: Scripps Research Translational Institute • University of Florida—UL1TR001427: UF Clinical and Translational Science Institute • University of New Mexico Health Sciences Center—UL1TR001449: University of New Mexico Clinical and Translational Science Center • University of Texas Health Science Center at San Antonio—UL1TR002645: Institute for Integration of Medicine and Science • Yale New Haven Hospital—UL1TR001863: Yale Center for Clinical Investigation.

## CONFLICT OF INTEREST STATEMENT

The authors report no conflict of interest.

## Supporting information

Supporting Information

## Data Availability

The N3C Enclave is available for public research use. More than 4,000 researchers have access to data in N3C, representing more than 300 US research institutions. Institutions must have a signed Data Use Agreement executed with the US National Center for Advancing Translational Sciences (NCATS) to access data. Investigators must complete mandatory training and submit a Data Use Request (DUR) to N3C. To request N3C data access, follow the instructions at https://covid.cd2h.org/onboarding. This project utilized data from N3C release 120, which can be replicated within the Enclave by qualified N3C users. All concepts and definitions are provided in Methods S3. All code used for analyses can be made available upon request.
