# Supplementary material for: Associations between COVID-19 therapies and outcomes in rural and urban America: A multisite, temporal analysis from the Alpha to Omicron SARS-CoV-2 variants
Source: J Rural Health. Author manuscript; Available in PMC 2025 Jan 1. (PMC11635344; doi:10.1111/jrh.12857)
Supplement: Supplemental Material [file NIHMS2015457-supplement-Supplemental_Material.docx]

**Supplementary Online Content**

*Temporal Trends in Rural-Urban COVID-19 Therapies and Outcomes from the Alpha to Omicron XBB Variants: A Multi-site Observational Cohort Study from the National COVID Cohort Collaborative (N3C)*

**Table of Contents**

1. ***Supplemental Methods***

**eMethods S-1.** STROBE Statement and Checklist

**eMethods S-2.** National COVID Cohort Collaborative (N3C) Structure and Data Collection

**eMethods S-3.** Concept Sets and Key Definitions

1. ***Supplemental Figures***

**eFigure S-1.** Patient Distribution of COVID-19 Positive Cohort

**eFigure S-2.** Population Caseload and Case Fatality by Rural-Dwelling Status in the United States, January 2020 – December 2022

**eFigure S-3.** Covariate Balance Plot After Propensity Score Matching

**eFigure S-4.** Kaplan Meier 45-Day Survival Estimates by Rurality and COVID-19 Epoch after Propensity-Score Matching

1. ***Supplemental Tables***

**eTable S-1.** COVID-19 Therapeutic Usage Over Time in 45 Days After Acute COVID-19

**eTable S-2.** Multivariable Regression for and Number of Adverse Acute COVID-19 Events by Rurality Across All Time Periods and within COVID-19 Epochs

**eTable S-3.** Univariable Regression for Adverse Acute COVID-19 Events by Rurality

**eTable S-4.** Multivariable Regression for Adverse Acute COVID-19 Events with COVID-19 Therapies Across All Time Periods

**eTable S-6.** Multivariable Regression for Adverse Acute COVID-19 Events with COVID-19 Therapies Stratified by Rurality Across All Time Periods

**eTable S-7.** Baseline Characteristics of Patients with SARS-CoV-2 Infection by Rural-Dwelling Status after Propensity-Score Matching

**eTable S-8.** Multivariable Regression for Adverse Acute COVID-19 Events by Rurality Across All Time Periods and within COVID-19 Epochs after Propensity-Score Matching

**eTable S-9.** Multivariable Regression for Adverse Acute COVID-19 Events Stratified by Rurality after Propensity-Score Matching

**eTable S-10.** Multivariable Regression for Adverse Acute COVID-19 Events with COVID-19 Therapies Combined Across All Time Periods after Propensity-Score Matching

**eTable S-11.** Multivariable Regression for Adverse Acute COVID-19 Events with COVID-19 Therapies Stratified by Rurality Across All Time Periods after Propensity-Score Matching

**eTable S-12.** Sensitivity Analysis 1: Multivariable Regression of Adverse Acute COVID-19 Events by Rural Binary Across All Time Periods

**eTable S-13.** Sensitivity Analysis 2: Multivariable Regression of Adverse Acute COVID-19 Events by Rurality Across All Time Periods among Patients who Survived the Initial 3 days After Acute COVID-19

**eTable S-14.** Sensitivity Analysis 3: Multivariable Regression of Adverse Acute COVID-19 Events by Rurality Across All Time Periods, Including Adjustments for Visit History Prior to COVID-19

**eTable 15.** Sensitivity Analysis 4: Multivariable Regression for Adverse Acute COVID-19 Events with COVID-19 Therapies Individually During the Time Periods they were Widely in Use by Rurality

**S-1 Methods. STROBE Statement and Checklist^1^**

|  | Item No. | Recommendation | |  |
| --- | --- | --- | --- | --- |
| **Title and abstract** | 1 | (*a*) Indicate the study’s design with a commonly used term in the title or the abstract | | 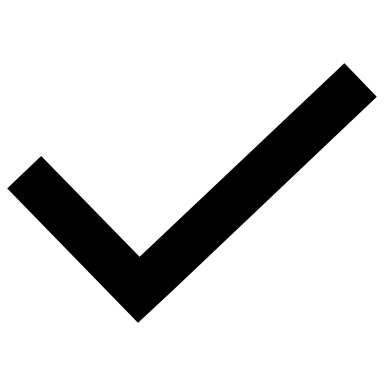 |
|  |  | (*b*) Provide in the abstract an informative and balanced summary of what was done and what was found | | 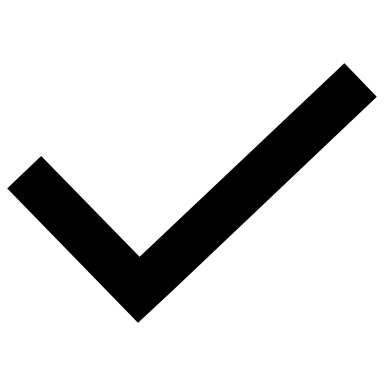 |
| Background/rationale | 2 | Explain the scientific background and rationale for the investigation being reported | | 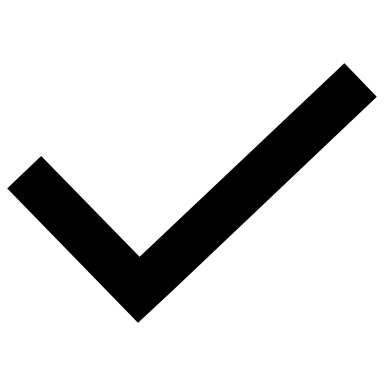 |
| Objectives | 3 | State specific objectives, including any prespecified hypotheses | | 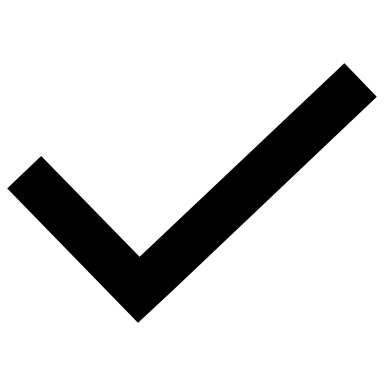 |
| Study design | 4 | Present key elements of study design early in the paper | | 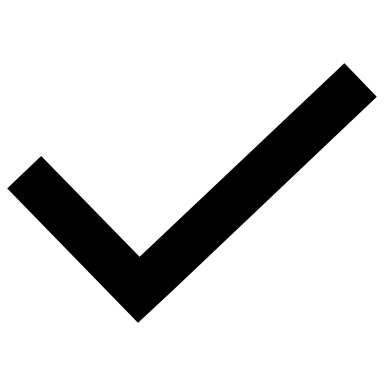 |
| Setting | 5 | Describe the setting, locations, and relevant dates, including periods of recruitment, exposure, follow-up, and data collection | | 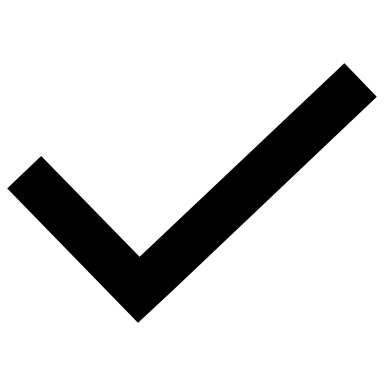 |
| Participants | 6 | (*a*) *Cohort study*—Give the eligibility criteria, and the sources and methods of selection of participants. Describe methods of follow-up | | 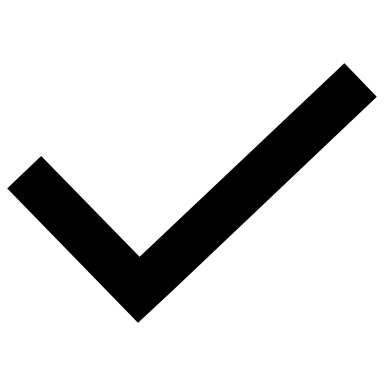 |
|  |  | (*b*) *Cohort study*—For matched studies, give matching criteria and number of exposed and unexposed | | 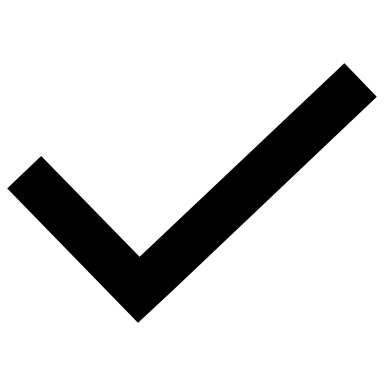 |
| Variables | 7 | Clearly define all outcomes, exposures, predictors, potential confounders, and effect modifiers. Give diagnostic criteria, if applicable | | 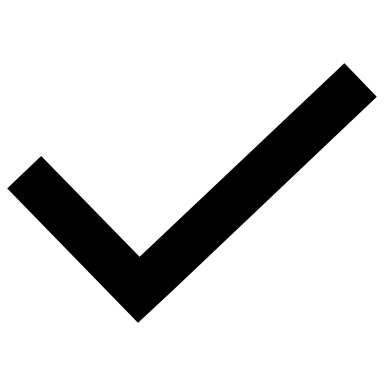 |
| Data sources/ measurement | 8* | For each variable of interest, give sources of data and details of methods of assessment (measurement). Describe comparability of assessment methods if there is more than one group | | 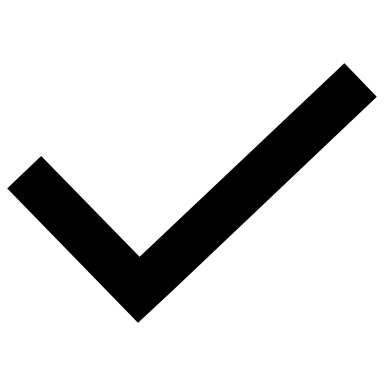 |
| Bias | 9 | Describe any efforts to address potential sources of bias | | 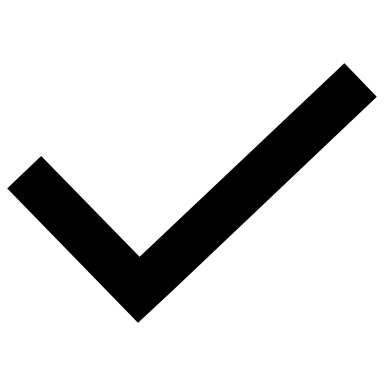 |
| Study size | 10 | Explain how the study size was arrived at | | 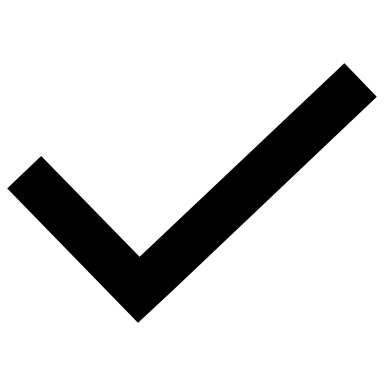 |
| Quantitative variables | 11 | Explain how quantitative variables were handled in the analyses. If applicable, describe which groupings were chosen and why | | 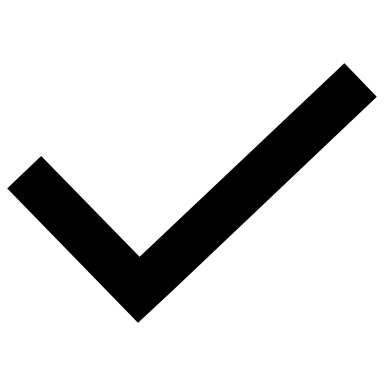 |
| Statistical methods | 12 | (*a*) Describe all statistical methods, including those used to control for confounding | | 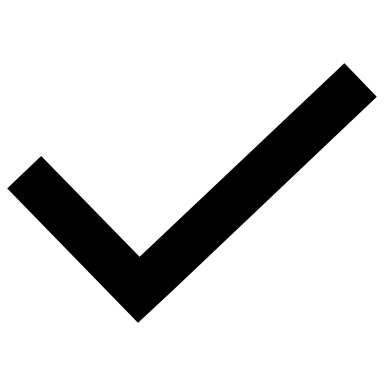 |
|  |  | (*b*) Describe any methods used to examine subgroups and interactions | | 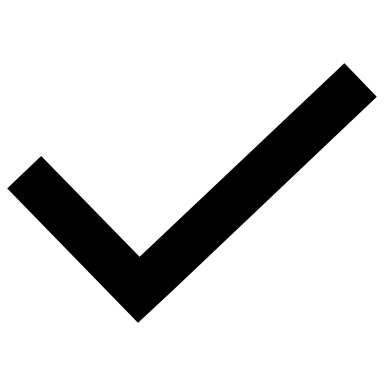 |
|  |  | (*c*) Explain how missing data were addressed | | 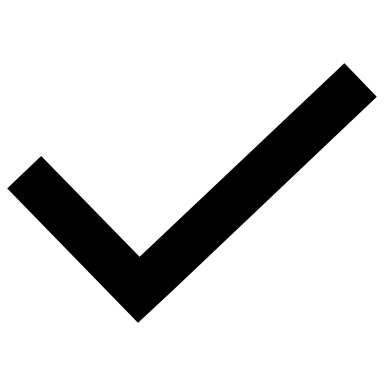 |
|  |  | (*d*) *Cohort study*—If applicable, explain how loss to follow-up was addressed | | 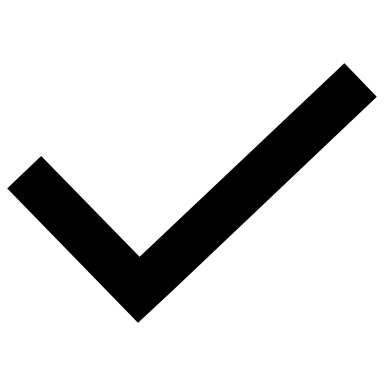 |
|  |  | (*e*) Describe any sensitivity analyses | | 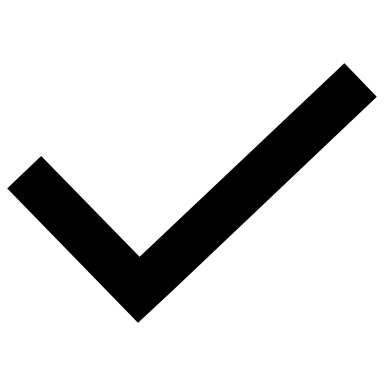 |
| Participants | 13* | (a) Report numbers of individuals at each stage of study—eg numbers potentially eligible, examined for eligibility, confirmed eligible, included in the study, completing follow-up, and analyzed | | N/A |
|  |  | (b) Give reasons for non-participation at each stage | | N/A |
|  |  | (c) Consider use of a flow diagram | | 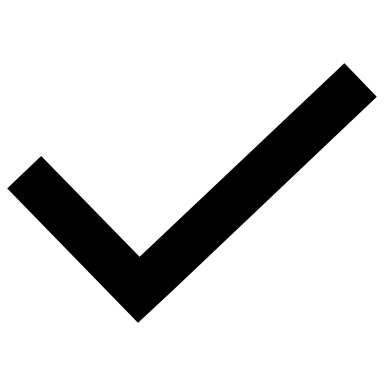 |
| Descriptive data | 14* | (a) Give characteristics of study participants (eg demographic, clinical, social) and information on exposures and potential confounders | | 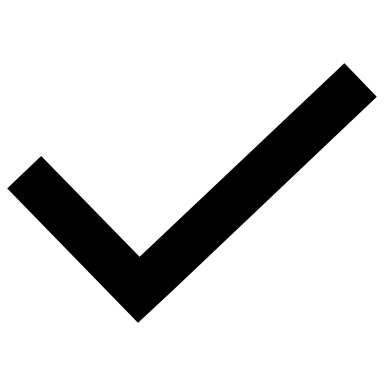 |
|  |  | (b) Indicate number of participants with missing data for each variable of interest | | N/A |
|  |  | (c) *Cohort study*—Summarize follow-up time (eg, average and total amount) | | 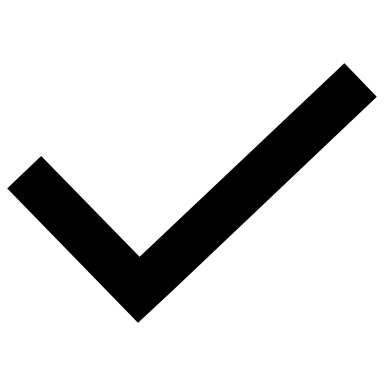 |
| Outcome data | 15* | *Cohort study*—Report numbers of outcome events or summary measures over time | | 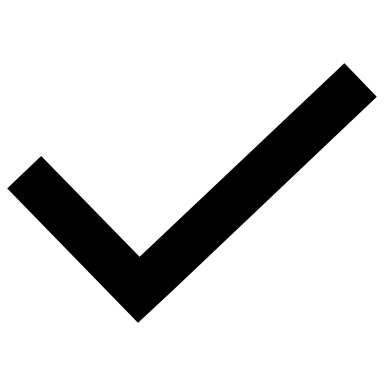 |
| Main results | 16 | (*a*) Give unadjusted estimates and, if applicable, confounder-adjusted estimates and their precision (e.g., 95% confidence interval). Make clear which confounders were adjusted for and why they were included | | 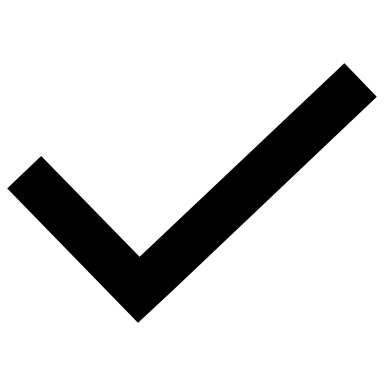 |
|  |  | (*b*) Report category boundaries when continuous variables were categorized | | N/A |
|  |  | (*c*) If relevant, consider translating estimates of relative risk into absolute risk for a meaningful time period | | 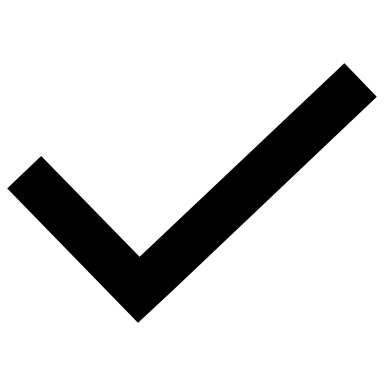 |
| Other analyses | 17 | Report other analyses done—eg analyses of subgroups and interactions, and sensitivity analyses | | 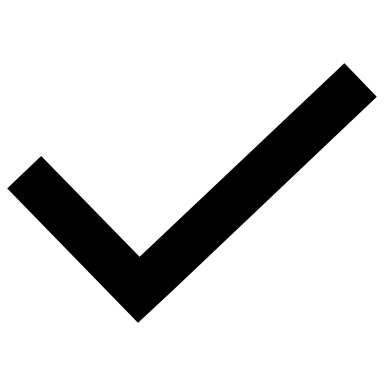 |
| Key results | 18 | Summarize key results with reference to study objectives | | 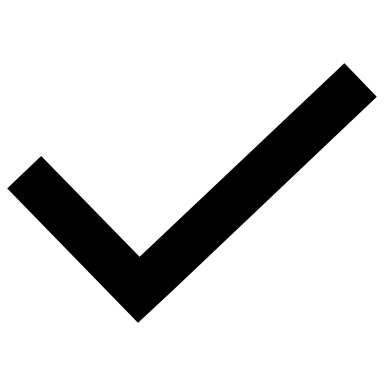 |
| Limitations | 19 | Discuss limitations of the study, taking into account sources of potential bias or imprecision. Discuss both direction and magnitude of any potential bias | | 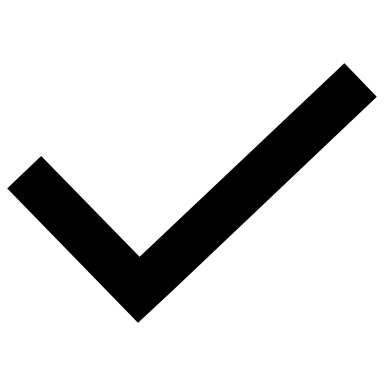 |
| Interpretation | 20 | Give a cautious overall interpretation of results considering objectives, limitations, multiplicity of analyses, results from similar studies, and other relevant evidence | | 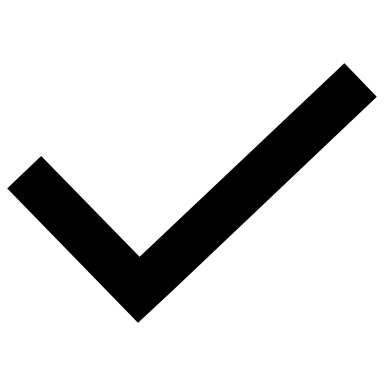 |
| Generalizability | 21 | Discuss the generalizability (external validity) of the study results | | 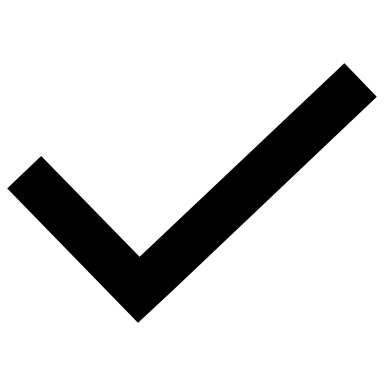 |
| Other information |  | |  |  |
| Funding | 22 | Give the source of funding and the role of the funders for the present study and, if applicable, for the original study on which the present article is based | | 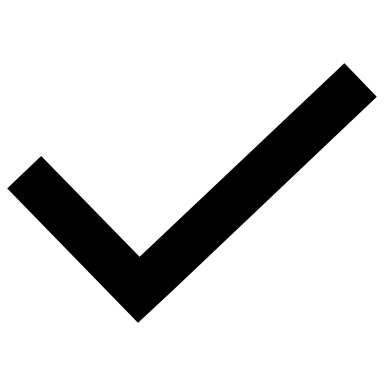 |

**S-2 Methods. National COVID Cohort Collaborative (N3C) Structure and Data Collection**

This supplementary methods section describes the data collection strategy and overall structure of the N3C Data Enclave. Its contents are adapted from previously published work developed by the Immunosuppressed/Compromised Domain Team and others in the N3C community: <https://covid.cd2h.org/compromised>.

**Data Access**

Researchers request access to a HIPAA Limited Data Set (LDS) or a HIPAA Safe Harbor (de-identified) version of the N3C data through a project-specific Data Use Request. The de-identified data set is generated from the LDS data by shifting all dates up to 180 days (except the birth year) and reducing zip codes to 3 digits.  N3C represents the largest centralized enclave of LDS captured in the United States for clinical research, relying on standardized robustness checks to ensure high-fidelity data. Once approved, access to N3C data is achieved through a secure Enclave, a virtual, secure, cloud-based environment that meets federal data security standards. Technical measures have been put in place such that no patient-level data may be downloaded or removed from the environment, and NCATS must approve all summarized data (including images) before publication or distribution.

**N3C Phenotype**

The N3C Phenotype is a broad set of criteria to identify patients important for COVID-19 research and for inclusion in the N3C database. This includes all lab-confirmed, suspected, and possible cases of COVID-19, and a demographically matched (on age group, sex, race, and ethnicity) control group who tested negative for COVID-19 at a ratio of 1:2 (cases: controls).  Briefly, COVID-19 cases are defined as any patient with an encounter after 1/1/2020 with either 1) one or more positive COVID-19 lab tests, 2) one or more “strong positive” diagnosis codes, or 3) two or more “weak positive” diagnosis codes during the same encounter on or before 5/1/2020.  Control cases must 1) not qualify as a case, 2) match a case demographically, 3) have one or more non-positive COVID-19 lab tests after 1/1/2020, and 4) have at least 10 days between the minimum and maximum encounter date to eliminate person’s that were only seen for testing. ^2^ The majority of COVID-19 positivity is from PCR or Antigen tests (78%) or physician diagnosis (19%) with the remaining coming from antibody-only testing (3%). Additional details, including specific labs and diagnostic codes, can be found in the N3C Phenotype GitHub repository (<https://github.com/National-COVID-Cohort-Collaborative/Phenotype_Data_Acquisition/wiki/Latest-Phenotype>).

**Data Characteristics**

N3C data is both retrospective and prospective.  Retrospective records dating back to 1/1/2018 are included for the historical medical context for all individuals in N3C.  In addition, once a patient is added to N3C (case or control), they remain in the cohort with new records being added until they qualify as a COVID-19 case.  If a control qualifies as a case, a new demographically matched control is selected and historical records are retrieved back to 1/1/2018 for inclusion in N3C. Patient records include various clinical data modeled by the OMOP CDM, including demographics, encounters, diagnoses, procedures, medications, vitals, labs, vaccinations, and other clinically relevant facts.  Additional details on the OMOP CDM can be found on the OHDSI (Observational Health Data Sciences and Informatics) website [<https://www.ohdsi.org/data-standardization/the-common-data-model/>] or OMOP GitHub repository [<https://ohdsi.github.io/CommonDataModel/>].

**Experimental Design and Data Analysis**

Designing experiments and performing data analysis must be done entirely within the N3C Enclave.  Computational phenotypes to identify cohorts can only be designed using concepts defined by OMOP and available in the N3C Enclave as researchers do not have access to the original EMR record.  As data is ingested from dozens of data partners using different CDMs some data that would normally be available in the original EHR, and used in clinically validated computational phenotypes, is either not present (e.g., O2 saturation) or sparsely populated (e.g., BMI).  Thus, N3C computational phenotypes and data analyses must take this into account during the experimental design phase and recognize that these may be possible limitations to the analysis.^3^

All statistical analyses were performed in R v4.1.3.^2^ Survival analysis was performed using the ‘survival’^3^ package. PSM utilized the ‘MatchIt’^4^ package and ‘Cobalt’^5^ packages. Base ‘stats’ package was used to perform Pearson’s Chi-squared, analysis of variance (ANOVA), and Wilcoxon rank sum tests, with table formatting aided by the ‘gtsummary’^6^ package. Data visualization was performed using the ‘ggplot2,’^7^ ‘ggstats’,^8^ and ‘usmap’^9^ packages.

**N3C Cohort Definition Process**

Defining a cohort in N3C first requires a computational phenotype be developed that utilizes Concepts and Concept Sets from the OMOP Standard Vocabulary.  During harmonization, source vocabularies like ICD-10 and RxNorm are mapped to the OMOP Standard Vocabulary.^10^  A computational phenotype is developed by identifying those diagnoses, labs, medications, and other criteria using source vocabularies.  Then the ATLAS tool (<http://atlas-demo.ohdsi.org/>) can map those to OMOP Concepts.  Once all relevant OMOP Concepts are collected, they can be grouped into Concept Sets, which is just a list of one or more related Concepts.  For example, Supplementary Method Table 1 provides an example where the concept of wheelchair dependence is first defined using source vocabularies, then is mapped to OMOP Concepts followed by the creation of a custom Concept Set that is imported into the N3C Concept Set table.  Once imported, Concept IDs and Concept Sets can be used to identify patients matching specific criteria to create a cohort.

**Supplementary Methods Table 1. Example of how a source vocabulary term is mapped to a OMOP Concept Set**

| **Source Vocabulary** | **Source ID** | **OMOP Concept ID** | **OMOP Domain** | **N3CConcept Set Name** | **N3C Concept Set ID** |
| --- | --- | --- | --- | --- | --- |
| CPT4 | 97542 | 2314299 | Procedure | [N3C][ISC] Wheelchair Dependence (v1) | 1869047 |
| ICD10-CM | Z99.3 | 45547382 | Condition |  |  |
| This specific concept set has 246 source vocabulary terms and can be accessed on ATLAS at <http://atlas-demo.ohdsi.org/#/conceptset/1869047/expression>. | | | | | |

**N3C Data Partners**

As of April 20, 2023 (N3C Release 120), N3C harmonizes EHR data from a data partner network that includes 76 institutions from across the United States. The majority of N3C data partners represent tertiary care centers (Supplementary Methods Table 2).  Additional sites have signed Data Transfer Agreements with their data pending for availability (<https://covid.cd2h.org/dashboard/>).

**Supplementary Methods Table 2. N3C Data Partners with Data Availability**

| **Site** | **Data Status** | **Data Model** | **URL** |
| --- | --- | --- | --- |
| Advocate Aurora Research Institute | available | OMOP | http://aurorahealthcare.org |
| Advocate Health Care Network | pending | pending | http://advocatehealth.com |
| Arkansas Children's Hospital | pending | pending | http://archildrens.org |
| Baylor College of Medicine | pending | pending | http://bcm.edu |
| Boston University Medical Campus | available | TRINETX | http://bu.edu |
| Brown University | available | OMOP | http://brown.edu |
| Carilion Clinic | available | TRINETX | http://carilionclinic.org |
| Charleston Area Medical Center | available | TRINETX | http://camc.org |
| Children's Hospital Colorado | available | OMOP | http://childrenscolorado.org |
| Children's Hospital of Philadelphia | pending | pending | http://chop.edu |
| Children's National Hospital | available | TRINETX | http://childrensnational.org |
| Cincinnati Children's Hospital Medical Center | pending | pending | http://cincinnatichildrens.org |
| Columbia University Irving Medical Center | available | OMOP | http://health.columbia.edu |
| Duke University | available | PCORNET | http://duke.edu |
| Emory University | pending | pending | http://emory.edu |
| George Washington University | available | OMOP | http://gwu.edu |
| HonorHealth | pending | pending | http://honorhealth.com |
| Icahn School of Medicine at Mount Sinai | available | OMOP | http://mssm.edu |
| Indiana University School of Medicine | available | OMOP | http://regenstrief.org |
| Johns Hopkins University | available | PCORNET | http://jhu.edu |
| Loyola Medicine | available | PCORNET | http://loyolamedicine.org |
| Loyola University Chicago | pending | pending | http://luc.edu |
| Maine Medical Center | available | OMOP | http://mmcri.org |
| Massachusetts General Brigham | available | ACT | http://massgeneralbrigham.org |
| Mayo Clinic Rochester | submitted | ACT | http://mayo.edu |
| Medical College of Wisconsin | available | TRINETX | http://mcw.edu |
| Medical College of Wisconsin | available | PCORnet | http://mcw.edu |
| Medical University of South Carolina | available | OMOP | http://musc.edu |
| Medical University of South Carolina | available | ACT | http://musc.edu |
| MedStar Health Research Institute | pending | pending | http://medstarhealth.org |
| MetroHealth | pending | pending | http://metrohealth.org |
| Montana State University | pending | pending | http://montana.edu |
| Montefiore Medical Center | available | OMOP | http://montefiore.org |
| Nemours | available | OMOP | http://nemours.org |
| New York University Grossman School of Medicine | pending | pending | http://med.nyu.edu |
| NorthShore University Health System | available | PCORnet | http://northshore.org |
| Northwestern University at Chicago | available | PCORNET | http://northwestern.edu |
| OCHIN | available | PCORNET | http://ochin.org |
| Ochsner Health System | available | PCORnet | http://ochsner.org/locations/ochsner-medical-center |
| Oregon Health & Science University | available | OMOP | http://ohsu.edu |
| Penn State | available | TRINETX | http://psu.edu |
| Rush University Medical Center | available | PCORNET | http://rush.edu |
| Rutgers, The State University of New Jersey | available | OMOP | http://rutgers.edu |
| Sanford Research | pending | pending | http://sanfordresearch.org |
| Stanford University | pending | pending | http://stanford.edu |
| Stony Brook University | available | TRINETX | http://stonybrook.edu |
| The Ohio State University | available | PCORNET | http://osu.edu |
| The Rockefeller University | pending | pending | http://rockefeller.edu |
| The Scripps Research Institute | pending | pending | http://scripps.edu |
| The State University of New York at Buffalo | submitted | OMOP | http://buffalo.edu |
| The University of Chicago | available | PCORNET | http://uchicago.edu |
| The University of Iowa | available | PCORNET | http://uiowa.edu |
| The University of Michigan at Ann Arbor | available | PCORNET | http://umich.edu |
| The University of Texas Health Science Center at Houston | available | ACT | http://uth.edu |
| The University of Texas Health Science Center at Rio Grande Valley | available | PCORnet | http://uth.edu |
| The University of Texas Health Science Center at Tyler | submitted | PCORnet | http://uth.edu |
| The University of Texas Medical Branch at Galveston | available | TRINETX | http://utmb.edu |
| The University of Utah | available | PCORNET | http://utah.edu |
| Tufts Medical Center | available | OMOP | http://tuftsmedicalcenter.org |
| Tulane (University Medical Center New Orleans) | available | PCORnet | http://lphi.org |
| University Medical Center New Orleans | available | PCORNET | http://umcno.org |
| University of Alabama at Birmingham | available | TRINETX | http://uab.edu |
| University of Arkansas for Medical Sciences | available | TRINETX | http://uams.edu |
| University of California at Davis | available | OMOP | http://ucdavis.edu |
| University of California at Irvine | available | OMOP | http://uci.edu |
| University of California at Los Angeles | available | OMOP | http://ucla.edu |
| University of California at San Diego | available | OMOP | http://ucsd.edu |
| University of California at San Fransisco | available | OMOP | http://ucsf.edu |
| University of California, Davis | submitted | OMOP | http://ucdavis.edu |
| University of California, Irvine | submitted | OMOP | http://uci.edu |
| University of California, Los Angeles | submitted | OMOP | http://ucla.edu |
| University of California, San Diego | submitted | OMOP | http://ucsd.edu |
| University of California, San Francisco | submitted | OMOP | http://ucsf.edu |
| University of Cincinnati | available | TRINETX | http://uc.edu |
| University of Colorado, Anschutz Medical Campus | available | OMOP | http://cuanschutz.edu |
| University of Florida | pending | pending | http://ufl.edu |
| University of Illinois at Chicago | available | ACT | http://uic.edu |
| University of Kansas Medical Center | available | PCORnet | http://kumc.edu |
| University of Kentucky | available | ACT | http://uky.edu |
| University of Massachusetts Medical School Worcester | submitted | OMOP | http://umassmed.edu |
| University of Massachusetts Medical School Worcester | submitted | TRINETX | http://umassmed.edu |
| University of Miami | available | PCORNET | http://miami.edu |
| University of Minnesota | available | ACT | http://umn.edu |
| University of Mississippi Medical Center | available | OMOP | http://umc.edu |
| University of Nebraska Medical Center | available | PCORNET | http://unmc.edu |
| University of New Mexico Health Sciences Center | pending | pending | http://hsc.unm.edu |
| University of North Carolina at Chapel Hill | available | PCORNET | http://unc.edu |
| University of Oklahoma Health Sciences Center | available | OMOP | http://ouhsc.edu |
| University of Puerto Rico | pending | pending | http://upr.edu |
| University of Rochester | available | OMOP | http://rochester.edu |
| University of Southern California | available | TRINETX | http://usc.edu |
| University of Texas Health Science Center at San Antonio | submitted | PCORnet | http://uthscsa.edu |
| University of Vermont | available | TRINETX | http://uvm.edu |
| University of Virginia | submitted | OMOP | http://virginia.edu |
| University of Washington | available | OMOP | http://washington.edu |
| University of Wisconsin?Madison | available | TRINETX | http://wisc.edu |
| Vanderbilt University Medical Center | available | OMOP | http://vumc.org |
| Virginia Commonwealth University | available | ACT | http://vcu.edu |
| Wake Forest University Health Sciences | available | PCORNET | http://wakehealth.edu |
| Washington University in St. Louis | available | OMOP | http://wustl.edu |
| Weill Medical College of Cornell University | available | OMOP | http://weill.cornell.edu |
| West Virginia University | available | TRINETX | http://wvu.edu |
| Yale New Haven Hospital | pending | pending | http://ynhh.org |

**Definitions (Computable Phenotypes)**

Coding algorithms used to identify cohorts in the N3C repository utilize OMOP Concept Identifiers (Concept IDs) that are associated with a patient’s submitted health records, which includes reported conditions, drugs, procedures, lab results and more. Concept IDs map a variety of medical vocabularies reported from each submitting institution, such as ICD10, CPT, RxNorm, SNOMED, and more, to a single unique identifier. Groups of related Concept IDs form a Concept Set, which can be used to query the N3C repository for patients meeting certain criteria. In this work we developed a series of OMOP Concept Sets along with inclusion/exclusion logic to define our cohorts.

All codesets used to define COVID-19 positivity, covariates, and outcomes of interest are provided in **S-3 Methods**. All Concept IDs and Concept Sets listed in the supplementary information for this work can be searched for specific source vocabulary terms, such as ICD10 and RxNorm, using the ATLAS demo browser (<http://atlas-demo.ohdsi.org/>). had data released for analysis and the N3C Enclave, and (3) had data that passed initial quality checks. Definitions include systematic utilization of both source mappings to OMOP standards done during the data ingestion process and mapping to source concepts for certain concepts that less clearly structured when transmitted to N3C. This process is described in detail in *The Guide to N3C*, chapter 8 “Introducing Enclave Analysis Tools” (https://national-covid-cohort-collaborative.github.io/guide-to-n3c-v1/).

Medication exposures were identified based on exposure between the earliest COVID-19 diagnosis and each adverse event. **Supplemental Methods Figure 1** demonstrates typical medication types in this study, which includes primarily outpatient-administered medications (nirmatrelvir/ritonavir, molnupiravir, and monoclonal antibody therapies), primarily inpatient-administered medications (Remdesivir, anticoagulants), and primarily post-IMV- or post-ECMO-administered medications (Dexamethasone, Tocilizumab). All medications are assigned an indicator if they occur between COVID-19 diagnosis and relevant adverse event for each category. Patients on background anticoagulants (1 year before SARS-CoV-2 infection) are not considered as having received anticoagulant therapy as part of their COVID-19 therapeutic regimen.

**Supplementary Methods Figure 1. Medication Exposure Types**


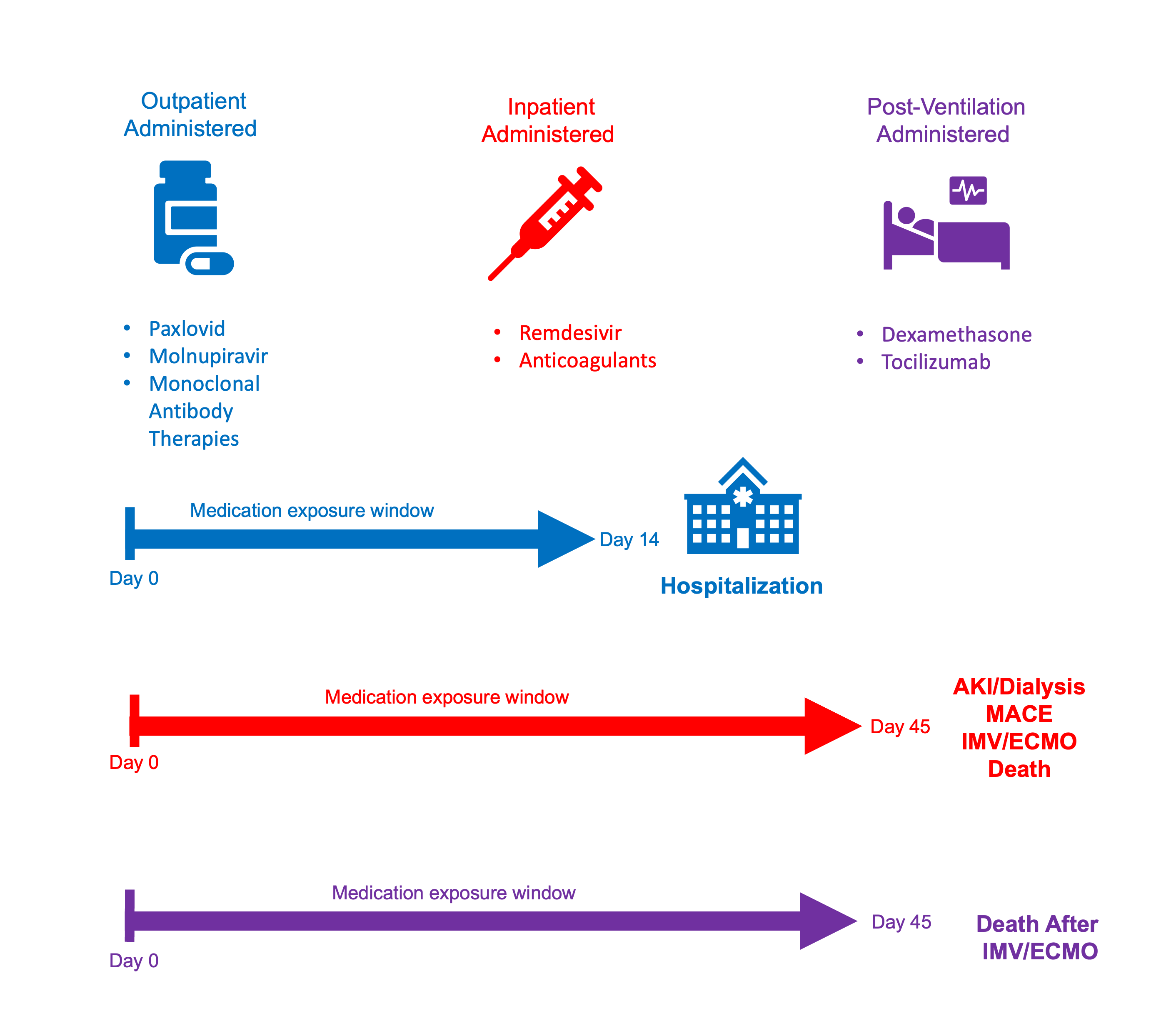


**Supplemental Methods Figure 2** documents potential medication exposure timing for three hypothetical patients. Patient A had Remdesivir and Dexamethasone administered on days 4 and 9, respectively, after their COVID-19 diagnosis. Patient A would thus have an indicator for remdesivir administered before invasive mechanical ventilation, which then occurred on Day 6. They were then administered dexamethasone on day 9, which would result in an indicator for models assessing hazard for inpatient death after ventilation. They were discharged from the hospital at Day 19, which was within their 45-day observation window after acute COVID-19 diagnosis. Patient B had no documented medication administration events and died at day 4 after their COVID-19 diagnosis. Patient C had nirmatrelvir/ritonavir administered in an outpatient setting and was followed for 14 days after COVID-19 diagnosis for a COVID-19-associated hospitalization. Inpatient adverse events are followed for up to 45 days after acute COVID-19 diagnosis.

**Supplementary Methods Figure 2. Examples of Potential Medication Timing Relative to Adverse Event or End of Follow-up Period**

**
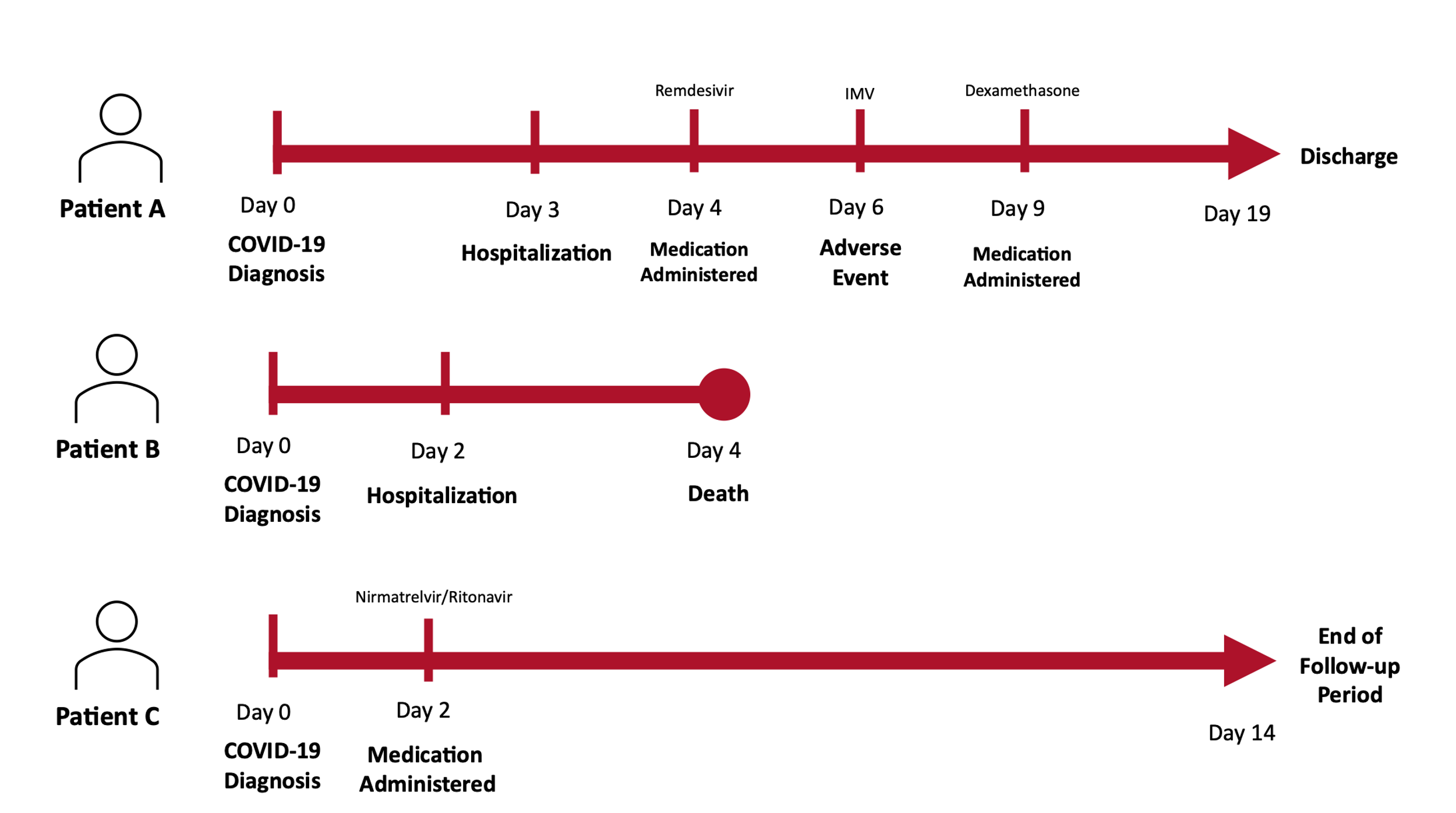
**

**S-3 Methods. Concept Sets and Key Definitions**

This supplemental section describes key concept definitions and exposures as used in this manuscript.

**Defining Rural in N3C**

There is no single definition of rural across Federal agencies, but they primarily rely on population density and geographic unit. There are three primary definitions from the U.S. Census Bureau, Office of Management and Budget, and Federal Office of Rural Health Policy. They are defined below:

U.S. Census Bureau definition of urban areas^7^

- Urbanized Areas (UAs) of 50,000 or more people
- Urban Clusters (UCs) of at least 2,500 and less than 50,000 people

Office of Management and Budget^7^

- A Metro area contains a core urban area of 50,000 or more population
- A Micro area contains an urban core of at least 10,000 (but less than 50,000) population

Federal Office of Rural Health Policy^7^

- Accepts all non-Metro counties as rural and uses an additional method of determining rurality called the Rural-Urban Commuting Area (RUCA) codes^8^
- RUCA Codes 1-3 are urban and 4-10 are rural (as well as 132 large area census tracts with RUCA codes 2 or 3)

The most granular and widely available unit of residential geography available in N3C is the 5-digit ZIP Code. As a result, this analysis relies on the Federal Office of Rural Health Policy's definition of rurality, utilizing RUCA Codes to indicate rurality. Patients were included if they had a 5-digit ZIP Code mapped to RUCA Codes through an external dataset maintained by the USDA Economic Research Services: <https://www.ers.usda.gov/data-products/rural-urban-commuting-area-codes/>. This dataset is available in N3C here: [[EXTDATASET-59] RUCA Rural-Urban Commuting Area Codes 1](https://unite.nih.gov/workspace/compass/view/ri.compass.main.folder.ade189b0-39fe-45b8-943c-fb4cf38fb996). Patients were mapped to three categories based on this ZIP Code crosswalk as follows based on primary RUCA Code designation as used in other published studies:^11,12^

Urban:

1. Metropolitan area core: primary flow within an urbanized area (UA)
2. Metropolitan area high commuting: primary flow 30% or more to a UA
3. Metropolitan area low commuting: primary flow 10% to 30% to a UA

Urban-Adjacent Rural:

1. Micropolitan area core: primary flow within an Urban Cluster of 10,000 to 49,999 (large UC)
2. Micropolitan high commuting: primary flow 30% or more to a large UC
3. Small town core: primary flow within an Urban Cluster of 2,500 to 9,999 (small UC)
4. Small town high commuting: primary flow 30% or more to a small UC

Nonurban-Adjacent Rural:

1. Micropolitan low commuting: primary flow 10% to 30% to a large UC
2. Small town low commuting: primary flow 10% to 30% to a small UC
3. Rural areas: primary flow to a tract outside a UA or UC

**Concept Sets in Use**

All concept sets used in this work are available below in **Supplemental Methods Table 3**. This table provides the concept set name (which can be found within the N3C Concept Set Browser), the Codeset ID (a unique id for each concept set in N3C, tied to both name and version), clinical domains used in the concept set (aligning to the OMOP CDM domains^13^), and a brief description of the concept set.

**Supplementary Methods Table 3: Concept Sets Used**

| **Concept Set Name** | **Codeset ID** | **Domain(s)** | **Author** |
| --- | --- | --- | --- |
| N3C Covid diagnosis | 35486128 | Condition | [REDACTED] |
| ATLAS SARS-CoV-2 rt-PCR and Ag | 651620200 | Measurement | [REDACTED] |
| ResultPos | 400691529 | Measurement Value | [REDACTED] |
| Hospitalization | N/A | Visit | [REDACTED] |
| [N3C] [ISC] MACE | 765147687 | Condition | [REDACTED] |
| [N3C] [ISC] MACE Hospitalization Required | 720679479 | Procedure, Condition | [REDACTED] |
| [ICU/MODS]IMV | 469361388 | Procedure, Observation, Condition | [REDACTED] |
| Kostka - ECMO | 415149730 | Procedure, Observation | [REDACTED] |
| CEREBROVASCULAR DISEASE | 718894835 | Condition | [REDACTED] |
| CHRONIC LUNG DISEASE | 525604750 | Condition | [REDACTED] |
| CONGESTIVE HEART FAILURE | 754910420 | Condition | [REDACTED] |
| CORONARY ARTERY DISEASE | 139102200 | Condition | [REDACTED] |
| DEMENTIA | 719082190 | Condition | [REDACTED] |
| DIABETES COMPLICATED | 18918743 | Condition | [REDACTED] |
| DIABETES UNCOMPLICATED | 248468138 | Condition | [REDACTED] |
| HEART FAILURE | 882775108 | Condition | [REDACTED] |
| HEMIPLEGIA or PARAPLEGIA | 157435065 | Condition | [REDACTED] |
| HIV INFECTION | 865749288 | Condition | [REDACTED] |
| HYPERTENSION | 834391873 | Condition | [REDACTED] |
| KIDNEY DISEASE | 579110282 | Condition | [REDACTED] |
| MALIGNANT CANCER | 585389357 | Condition | [REDACTED] |
| METASTATIC SOLID TUMOR CANCERS | 916369077 | Condition | [REDACTED] |
| MILD LIVER DISEASE | 590841465 | Condition | [REDACTED] |
| MODERATE OR SEVERE LIVER DISEASE | 437957818 | Condition | [REDACTED] |
| MYOCARDIAL INFARCTION | 933777427 | Condition | [REDACTED] |
| N3C CORTICOSTEROIDS FOR SYSTEMIC USE | 425332925 | Drug | [REDACTED] |
| OBESITY | 581513221 | Condition, Observation | [REDACTED] |
| PEPTIC ULCER | 413781282 | Condition | [REDACTED] |
| PERIPHERAL VASCULAR DISEASE | 817711041 | Condition | [REDACTED] |
| RHEUMATOLOGIC DISEASE | 903209001 | Condition | [REDACTED] |
| SUBSTANCE ABUSE | 606624084 | Condition | [REDACTED] |
| TOBACCO SMOKER | 628969102 | Observation | [REDACTED] |
| [N3C] [ISC] AKI | 843458909 | Condition | [REDACTED] |
| [AKI] Dialysis | 777835196 | Procedure, Observation, Measurement | [REDACTED] |
| Dexamethasone | 815004753 | Drug | [REDACTED] |
| Ivermectin | 980395214 | Drug | [REDACTED] |
| paxlovid | 798981734 | Drug | [REDACTED] |
| Remdesivir | 719693192 | Drug | [REDACTED] |
| Chloroquine | 818210864 | Drug | [REDACTED] |
| N3C Hydroxychloroquine | 807281242 | Drug | [REDACTED] |
| [Pitt N3C] Apixaban | 259221776 | Drug | [REDACTED] |
| [Pitt N3C] Rivaroxaban | 544420473 | Drug | [REDACTED] |
| [Pitt N3C] Dabigatran | 23600781 | Drug | [REDACTED] |
| [CVDT] Enoxaparin | 858278110 | Drug | [REDACTED] |
| [CVDT] Warfarin | 441951686 | Drug | [REDACTED] |
| [Pitt N3C] Betrixaban | 568693141 | Drug | [REDACTED] |
| [PASC] nirmatrelvir | 399252964 | Drug | [REDACTED] |
| [PASC] ritonavir | 329050933 | Drug | [REDACTED] |
| Lopinavir & Ritonavir | 165611849 | Drug | [REDACTED] |
| tenofovir alafenamide | 387127421 | Drug | [REDACTED] |
| Tenofovir disoproxil fumarate | 366325963 | Drug | [REDACTED] |
| interferonbeta-1a | 359012050 | Drug | [REDACTED] |
| interferonbeta-1b | 531467540 | Drug | [REDACTED] |
| [RHDT] Tixagevimab | 882538817 | Drug | [REDACTED] |
| [RHDT] Cilgavimab | 445489305 | Drug | [REDACTED] |
| [RHDT] Tocilizumab | 152723863 | Drug | [REDACTED] |
| [RHDT] Bamlanivimab | 279107457 | Drug | [REDACTED] |
| [RHDT] Etesevimab | 690302347 | Drug | [REDACTED] |
| [RHDT] Casirivimab | 443231483 | Drug | [REDACTED] |
| [RHDT] Imdevimab | 907711827 | Drug | [REDACTED] |
| [RHDT] Sotrovimab | 417597790 | Drug | [REDACTED] |

**eFigure S-1. Patient Distribution of COVID-19 Positive Cohort**

**A. Percentage of SARS-CoV-2 Infected Persons Represented in N3C by County in Study Cohort**

**
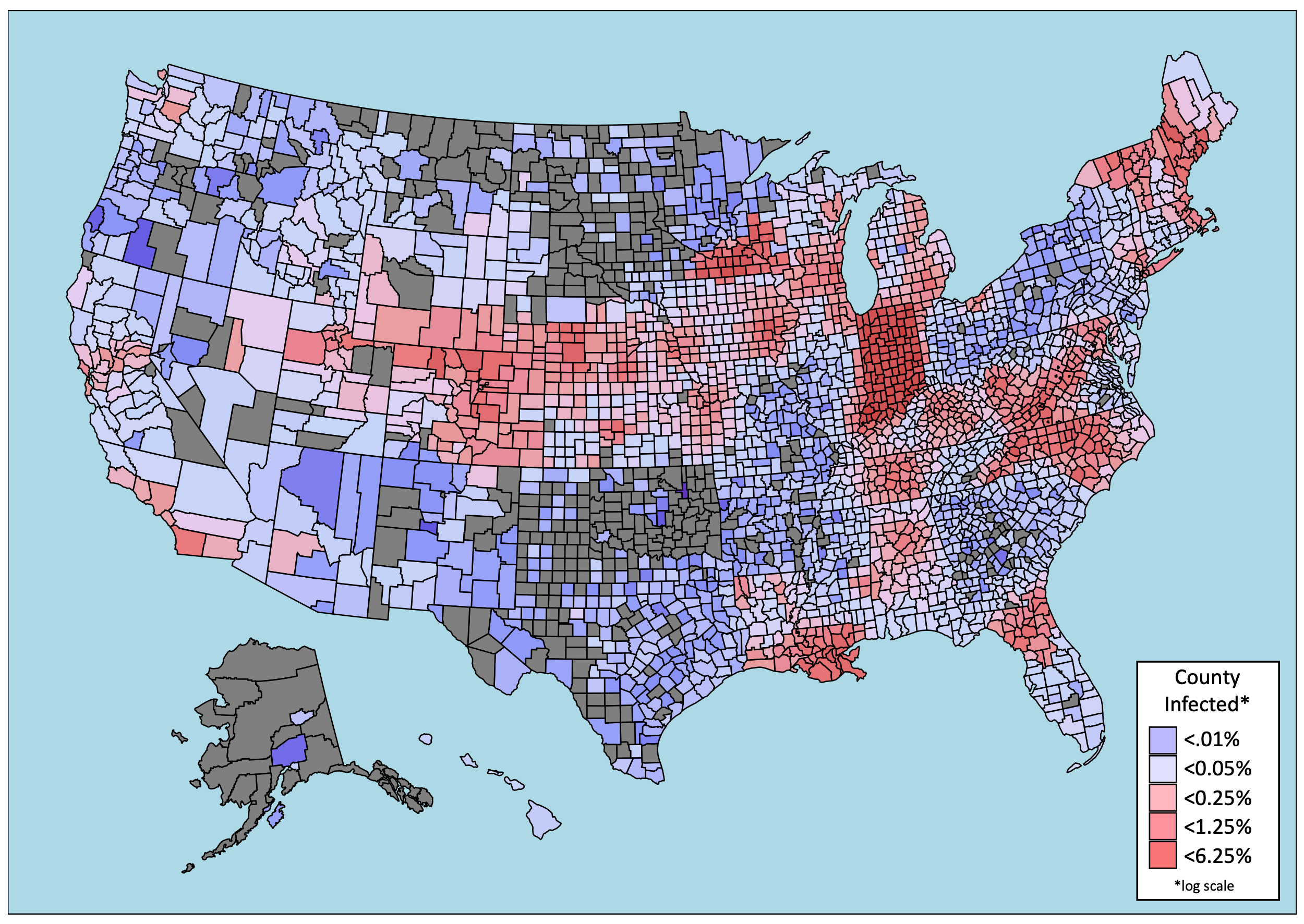
**

Figure Caption: Percentage presented is in log scale and represents percentage of overall population per US county (US Census 2013) in the entire study cohort. Counties with counts fewer than 20 persons not mapped (presented in grey) on map to comply with N3C privacy policies.

**B. Counts of SARS-CoV-2 Infected Persons Represented in N3C by County in Study Cohort**

**
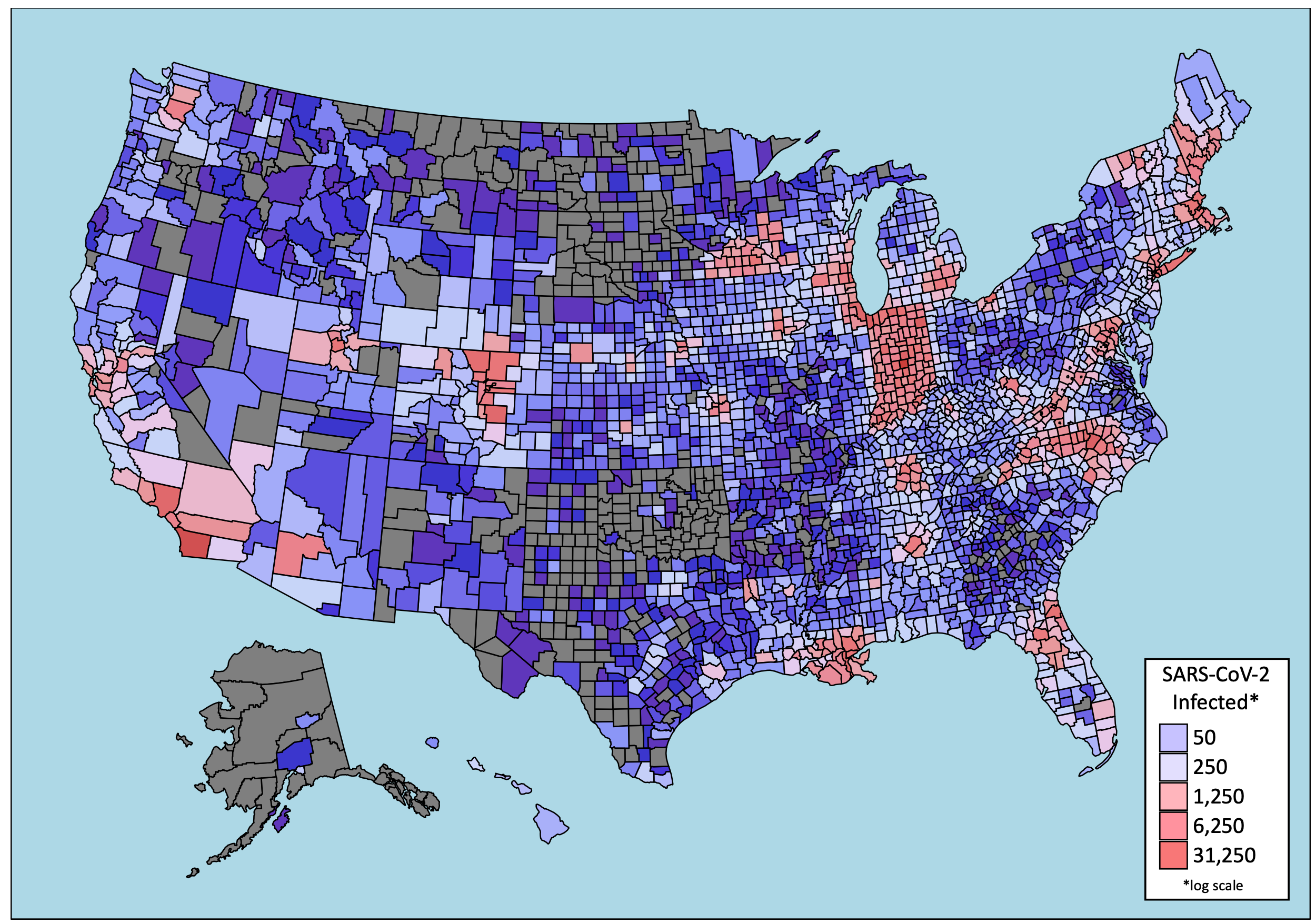
**

Figure Caption: Percentage presented is in log scale and represents actual patients in the entire study cohort. Counties with counts fewer than 20 persons not mapped (presented in grey) on map to comply with N3C privacy policies.

**eFigure S-2. Population Caseload and Case Fatality by Rural-Dwelling Status in the United States, January 2020 – December 2022**

**
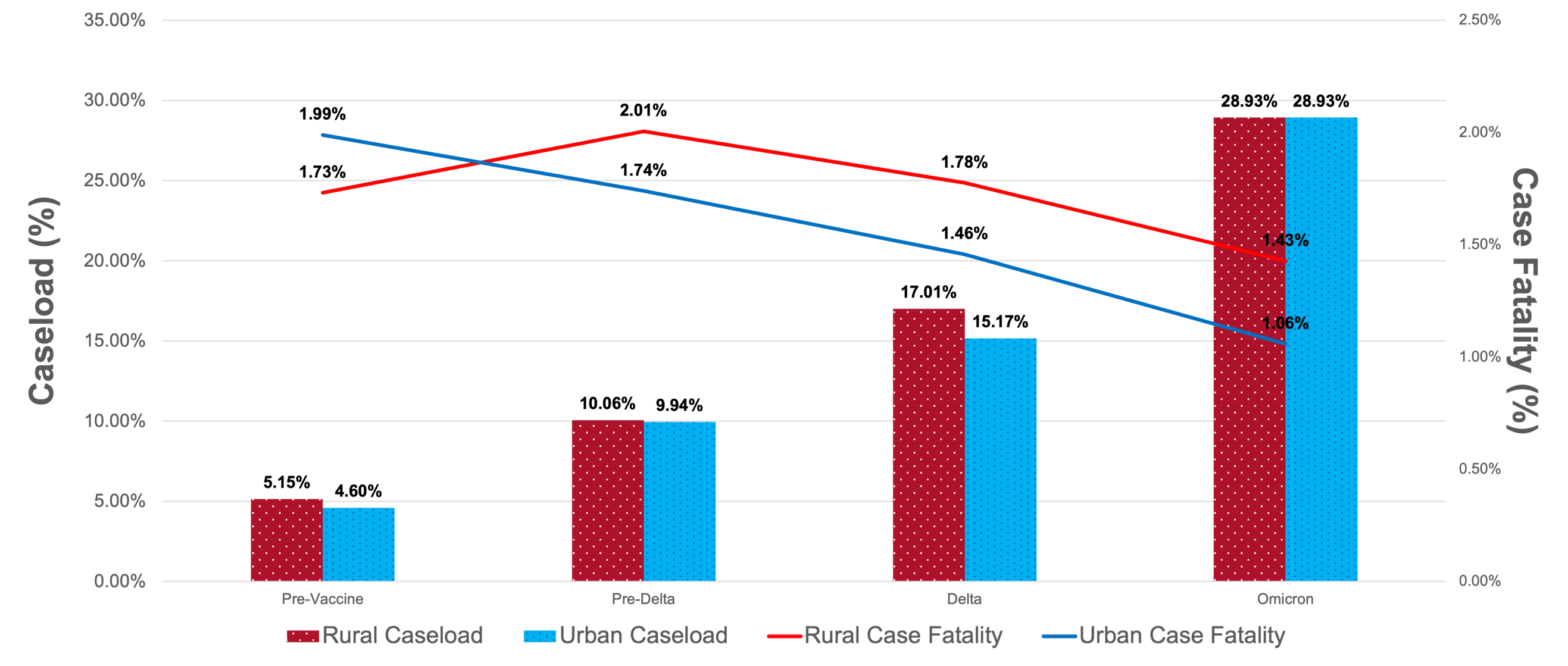
**

Data gathered from the Johns Hopkins University dashboard,^14^ mapped to rurality by county FIPS codes and Rural Urban Continuum Codes.^15^ Populations derived from the 2020 US Census.^16^

**eFigure S-3. Covariate Balance Plot After Propensity Score Matching**

**
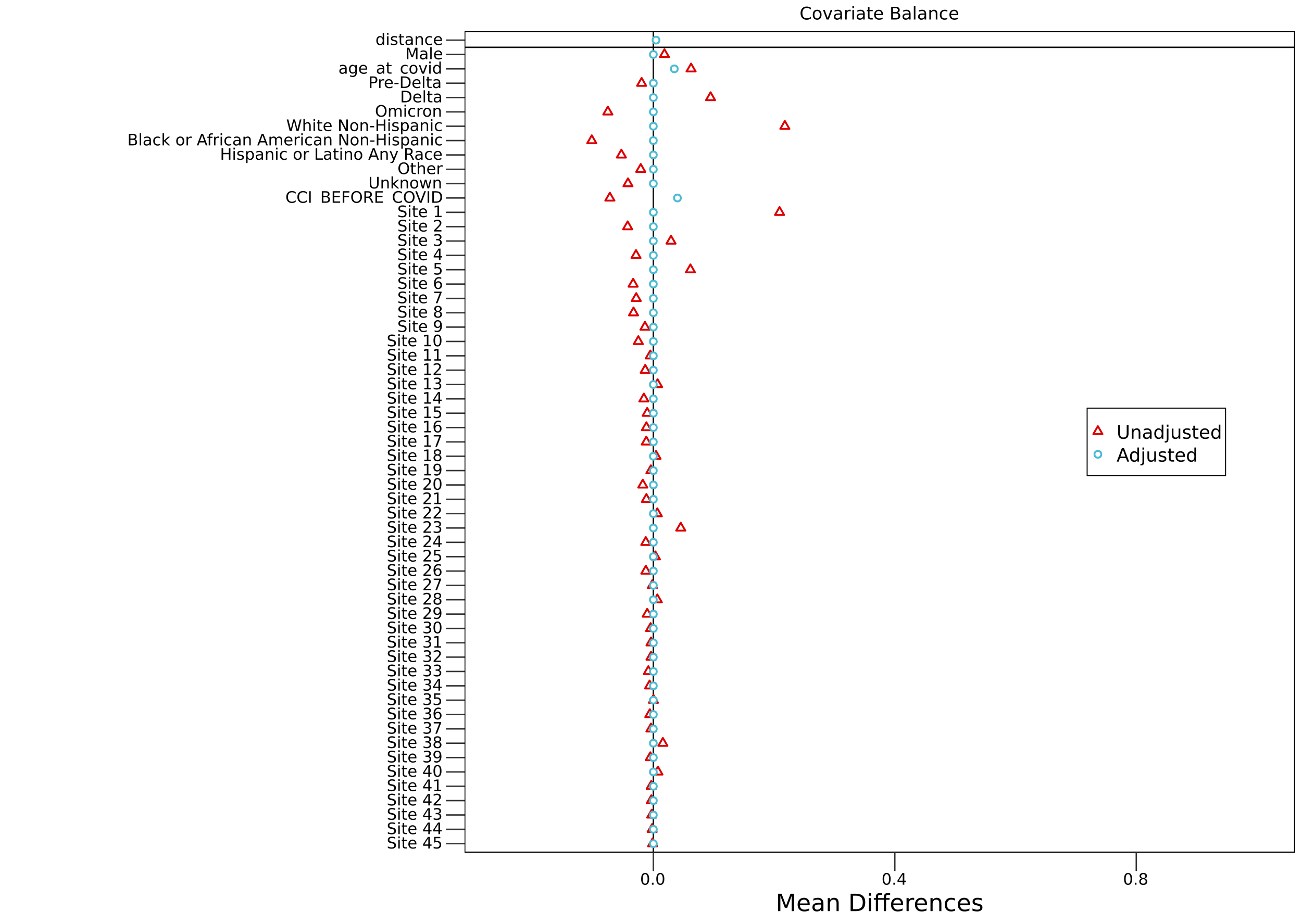
**

**eFigure S-4. Kaplan Meier 45-Day Survival Estimates by Rurality and COVID-19 Epoch after Propensity-Score Matching**

A. Rurality Across All Time Periods


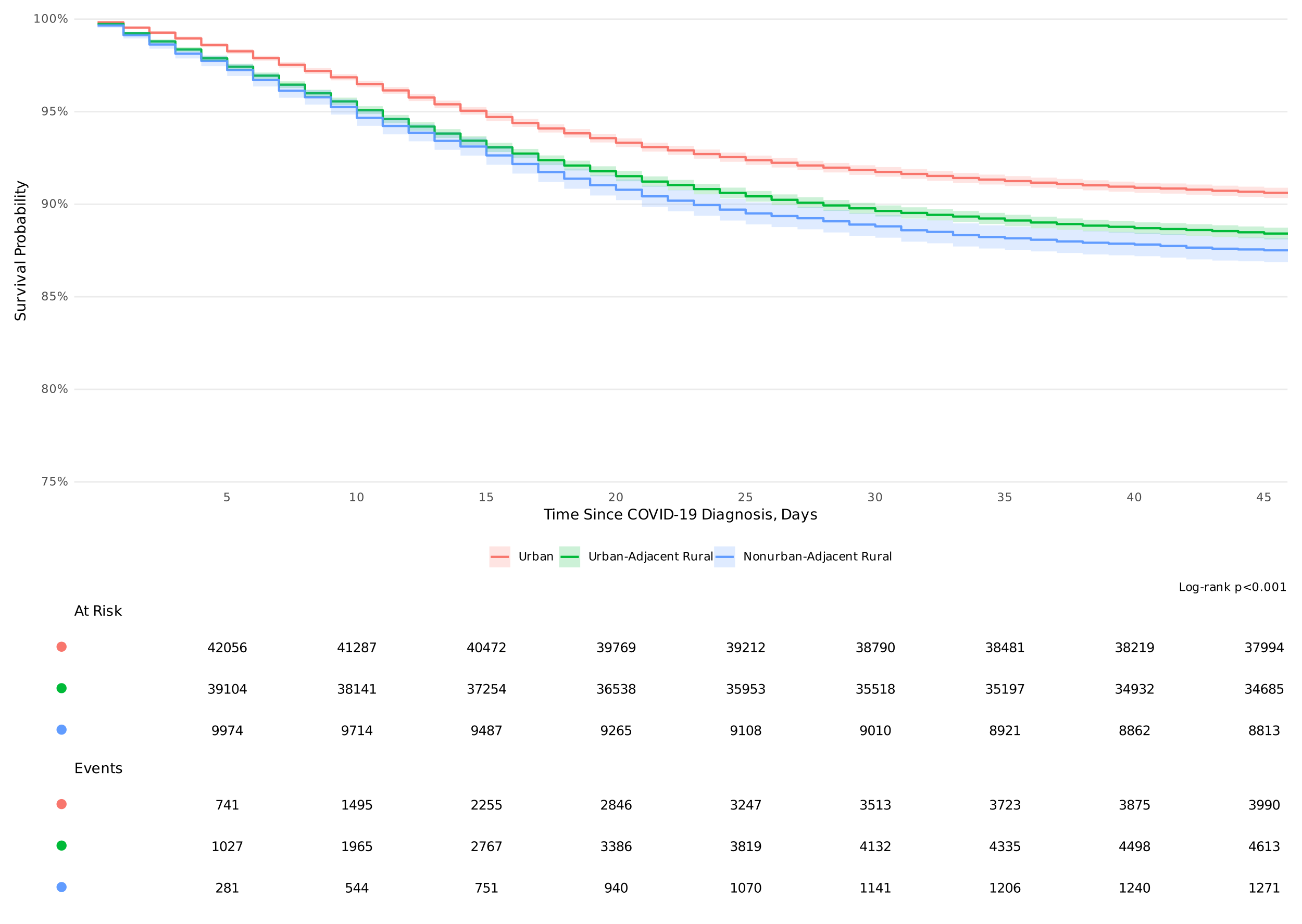


B. COVID-19 Epochs


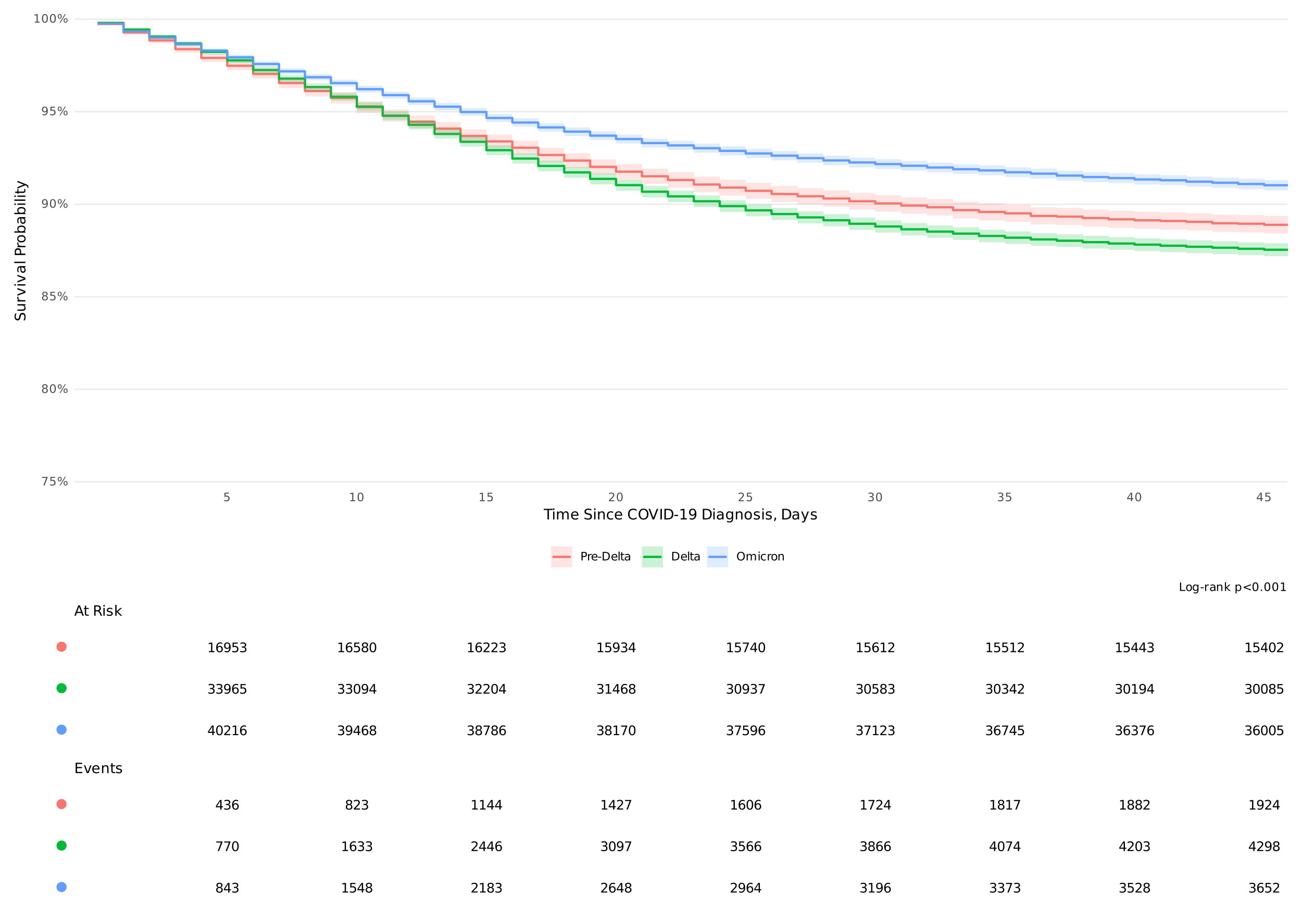


C. Rurality Stratified by COVID-19 Epochs


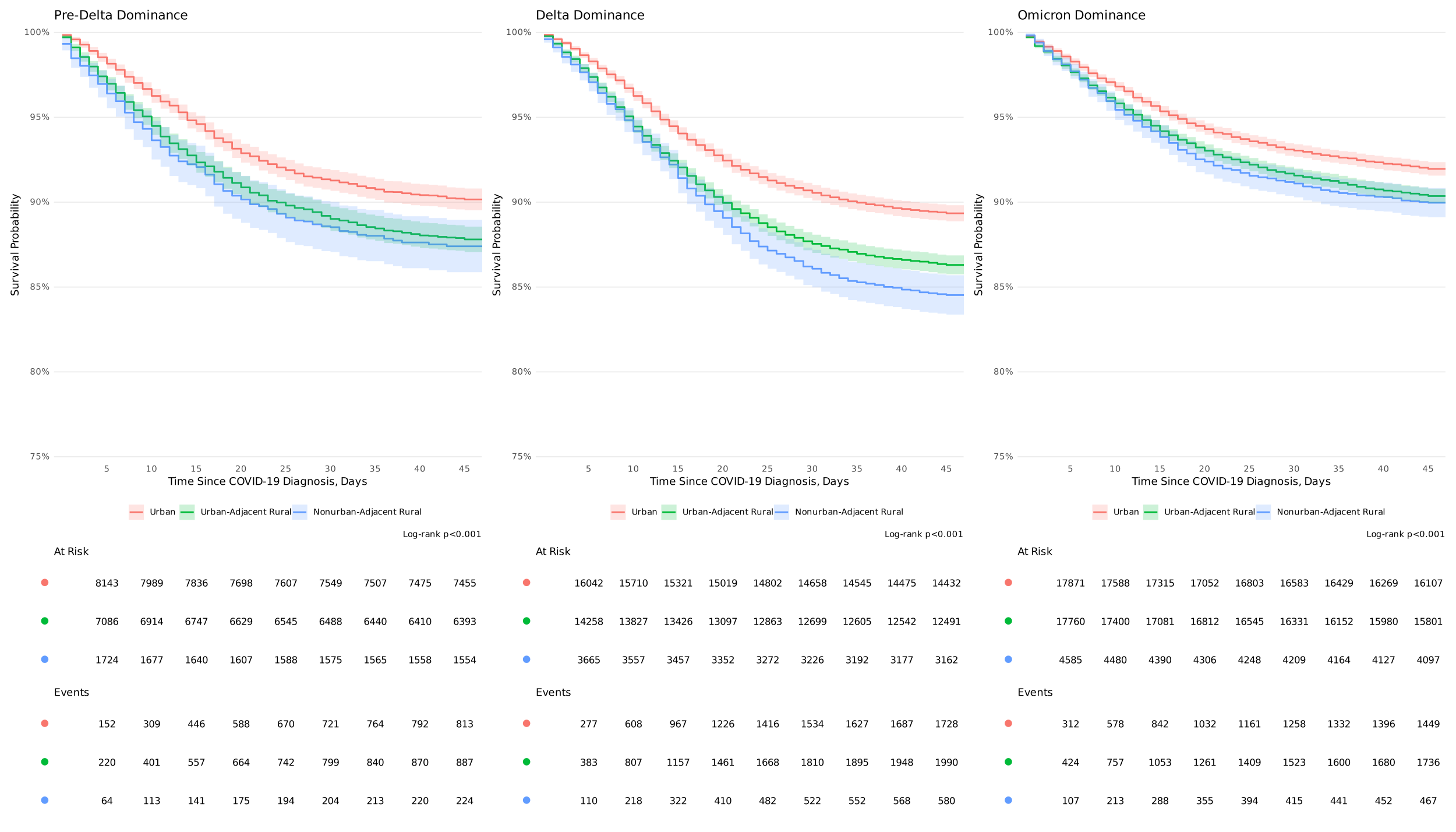


Figure Caption: Kaplan Meier Survival 45-day survival curves based on A) Rurality, B) COVID-19 Epoch, and C) Rurality Stratified by COVID-19 Epoch.

**eTable S-1. COVID-19 Therapeutic Usage Over Time in 45 Days After Acute COVID-19**

| **Therapeutic** | **Urban** | **Rural** | ***p* value*** | **Date of EUA or Widespread Use**** | **Date EUA Revoked**** |
| --- | --- | --- | --- | --- | --- |
| Dexamethasone | 167,902 (6.7%) | 32,294 (6.4%) | <0.001 | June 16, 2020^17^ | N/A |
| Remdesivir | 74,634 (3.0%) | 13,009 (2.6%) | <0.001 | May 1, 2020^18^ | N/A |
| Anticoagulants | 142,736 (5.7%) | 25,863 (5.1%) | <0.001 | N/A | N/A |
| Nirmatrelvir/Ritonavir | 88,683 (3.5%) | 9,120 (1.8%) | <0.001 | December 22, 2021 | N/A |
| Molnupiravir | 12,706 (0.5%) | 2,744 (0.5%) | <0.001 | December 23, 2021 | N/A |
| Monoclonal Antibody Therapies*** | 104,943 (4.2%) | 22,687 (4.5%) | <0.001 | November 9, 2020 | January 26, 2023 |
| Tocilizumab^3^ | 5,559 (0.2%) | 1,187 (0.2%) | 0.069 | June 24, 2021 | N/A |
| Bamlanivimab^4^ | 15,402 (0.6%) | 3,966 (0.8%) | <0.001 | November 9, 2020 | April 16, 2021 |
| Etesevimab | 7,590 (0.3%) | 1,396 (0.3%) | 0.002 | February 9, 2021 | January 24, 2022 |
| Bebtelovimab | 9,692 (0.4%) | 1,466 (0.3%) | <0.001 | February 11, 2022 | November 30, 2022 |
| Casirivimab^5^ | 53,801 (2.1%) | 12,392 (2.4%) | <0.001 | November 21, 2020 | January 24, 2022 |
| Imdevimab^5^ | 46,555 (1.9%) | 8,253 (1.6%) | <0.001 | November 21, 2020 | January 24, 2022 |
| Sotrovimab | 10,684 (0.4%) | 2,409 (0.5%) | <0.001 | May 26, 2021 | April 5, 2022 |
| Tixagevimab^6^ | 1,152 (<0.1%) | 282 (<0.1%) | 0.003 | December 8, 2021 | January 26, 2023 |
| Cilgavimab^6^ | 319 (<0.1%) | 78 (<0.1%) | 0.12 | December 8, 2021 | January 26, 2023 |
| * Pearson's Chi-squared test  ** Date the U.S. Food and Drug Administration issued an emergency use authorization (EUA) or clinical guidelines were released recommending use  *** Overall monoclonal antibody counts do not include tocilizumab in aggregate as it was primarily used for moderate to severe COVID-19 while the other monoclonal antibody therapies were used for mild COVID-19. **On April 16, 2021, the FDA revoked the individual approval of bamlanivimab and only authorized its use in combination with** etesevimab. Casirivimab and imdevimab are administered together as REGEN-COV®. Tixagevimab was co-packaged with cilgavimab as EVUSHELD^TM^, which had an EUA issued December 8, 2021, which was revoked on January 26, 2023. | | | | | |

**eTable S-2. Multivariable Regression for and Number of Adverse Acute COVID-19 Events** **by Rurality Across All Time Periods and within COVID-19 Epochs**

| **Characteristic** | **All Time Periods, January 2021 – December 2022** | | | | | | | | | |
| --- | --- | --- | --- | --- | --- | --- | --- | --- | --- | --- |
|  | **Hospitalization** | | **AKI/Dialysis** | | **MACE** | | **ECMO/IMV** | | **Death** | |
|  | **N Events / N (%)** | **Adjusted Odds Ratio (95% CI)** | **N Events / N (%)** | **Adjusted Hazard Ratio (95% CI)** | **N Events / N (%)** | **Adjusted Hazard Ratio (95% CI)** | **N Events / N (%)** | **Adjusted Hazard Ratio (95% CI)** | **N Events / N (%)** | **Adjusted Hazard Ratio (95% CI)** |
| Sex |  |  |  |  |  |  |  |  |  |  |
| Female | 152,354 / 1,723,967 (8.8%) | Reference | 27,568 / 152,354 (18%) | Reference | 6,653 / 152,354 (4.4%) | Reference | 9,168 / 152,354 (6.0%) | Reference | 12,074 / 152,354 (7.9%) | Reference |
| Male | 147,159 / 1,294,679 (11%) | 1.21 (1.20-1.22) | 37,743 / 147,159 (26%) | 1.45 (1.43-1.47) | 9,505 / 147,159 (6.5%) | 1.41 (1.36-1.45) | 13,550 / 147,159 (9.2%) | 1.50 (1.46-1.54) | 16,107 / 147,159 (11%) | 1.35 (1.32-1.38) |
| Age at COVID-19 Diagnosis, Median (IQR) | 299,513 / 3,018,646 (9.9%) | 1.04 (1.04-1.04) | 65,311 / 299,513 (22%) | 1.02 (1.02-1.02) | 16,158 / 299,513 (5.4%) | 1.03 (1.03-1.03) | 22,718 / 299,513 (7.6%) | 1.00 (1.00-1.00) | 28,181 / 299,513 (9.4%) | 1.04 (1.04-1.04) |
| Race/Ethnicity |  |  |  |  |  |  |  |  |  |  |
| White Non-Hispanic | 188,126 / 2,050,535 (9.2%) | Reference | 37,228 / 188,126 (20%) | Reference | 9,942 / 188,126 (5.3%) | Reference | 13,630 / 188,126 (7.2%) | Reference | 19,007 / 188,126 (10%) | Reference |
| Black or African American Non-Hispanic | 55,922 / 393,047 (14%) | 1.79 (1.77-1.81) | 16,755 / 55,922 (30%) | 1.64 (1.61-1.68) | 3,329 / 55,922 (6.0%) | 1.25 (1.20-1.30) | 4,142 / 55,922 (7.4%) | 1.05 (1.01-1.09) | 4,164 / 55,922 (7.4%) | 0.96 (0.93-0.99) |
| Hispanic or Latino Any Race | 32,094 / 254,459 (13%) | 1.86 (1.84-1.89) | 6,251 / 32,094 (19%) | 1.10 (1.06-1.13) | 1,377 / 32,094 (4.3%) | 0.96 (0.91-1.02) | 2,718 / 32,094 (8.5%) | 1.13 (1.08-1.18) | 2,648 / 32,094 (8.3%) | 1.13 (1.08-1.18) |
| Other | 10,073 / 139,640 (7.2%) | 1.03 (1.01-1.05) | 2,307 / 10,073 (23%) | 1.25 (1.19-1.30) | 658 / 10,073 (6.5%) | 1.29 (1.19-1.40) | 925 / 10,073 (9.2%) | 1.31 (1.22-1.40) | 1,037 / 10,073 (10%) | 1.20 (1.13-1.28) |
| Missing/Unknown | 13,298 / 180,965 (7.3%) | 0.96 (0.95-0.98) | 2,770 / 13,298 (21%) | 1.21 (1.17-1.26) | 852 / 13,298 (6.4%) | 1.31 (1.22-1.41) | 1,303 / 13,298 (9.8%) | 1.37 (1.29-1.46) | 1,325 / 13,298 (10%) | 1.31 (1.24-1.39) |
| Myocardial infarction (MI) |  |  |  |  |  |  |  |  |  |  |
| No History of MI | 277,566 / 2,946,537 (9.4%) | Reference | 57,039 / 277,566 (21%) | Reference | 12,454 / 277,566 (4.5%) | Reference | 20,849 / 277,566 (7.5%) | Reference | 25,119 / 277,566 (9.0%) | Reference |
| History of MI | 21,947 / 72,109 (30%) | 1.18 (1.16-1.20) | 8,272 / 21,947 (38%) | 1.01 (0.99-1.04) | 3,704 / 21,947 (17%) | 2.71 (2.59-2.83) | 1,869 / 21,947 (8.5%) | 1.04 (0.98-1.09) | 3,062 / 21,947 (14%) | 1.05 (1.01-1.10) |
| Congestive heart failure (CHF) |  |  |  |  |  |  |  |  |  |  |
| No History of CHF | 253,151 / 2,876,555 (8.8%) | Reference | 48,143 / 253,151 (19%) | Reference | 11,563 / 253,151 (4.6%) | Reference | 19,055 / 253,151 (7.5%) | Reference | 21,823 / 253,151 (8.6%) | Reference |
| History of CHF | 46,362 / 142,091 (33%) | 1.70 (1.67-1.72) | 17,168 / 46,362 (37%) | 1.06 (1.04-1.09) | 4,595 / 46,362 (9.9%) | 1.22 (1.17-1.28) | 3,663 / 46,362 (7.9%) | 1.05 (1.01-1.10) | 6,358 / 46,362 (14%) | 1.14 (1.11-1.19) |
| Peripheral vascular disease (PVD) |  |  |  |  |  |  |  |  |  |  |
| No History of PVD | 274,298 / 2,929,336 (9.4%) | Reference | 55,968 / 274,298 (20%) | Reference | 13,690 / 274,298 (5.0%) | Reference | 20,784 / 274,298 (7.6%) | Reference | 24,773 / 274,298 (9.0%) | Reference |
| History of PVD | 25,215 / 89,310 (28%) | 1.09 (1.07-1.11) | 9,343 / 25,215 (37%) | 0.98 (0.95-1.00) | 2,468 / 25,215 (9.8%) | 1.05 (1.00-1.10) | 1,934 / 25,215 (7.7%) | 0.96 (0.91-1.02) | 3,408 / 25,215 (14%) | 1.03 (0.99-1.07) |
| Cerebrovascular disease (CVD) |  |  |  |  |  |  |  |  |  |  |
| No History of CVD | 274,577 / 2,921,549 (9.4%) | Reference | 57,072 / 274,577 (21%) | Reference | 13,690 / 274,577 (5.0%) | Reference | 20,942 / 274,577 (7.6%) | Reference | 24,902 / 274,577 (9.1%) | Reference |
| History of CVD | 24,936 / 97,097 (26%) | 0.98 (0.96-1.00) | 8,239 / 24,936 (33%) | 0.96 (0.93-0.99) | 2,468 / 24,936 (9.9%) | 1.16 (1.10-1.22) | 1,776 / 24,936 (7.1%) | 0.98 (0.92-1.03) | 3,279 / 24,936 (13%) | 0.95 (0.91-0.99) |
| Dementia |  |  |  |  |  |  |  |  |  |  |
| No History of dementia | 285,803 / 2,976,228 (9.6%) | Reference | 61,153 / 285,803 (21%) | Reference | 15,175 / 285,803 (5.3%) | Reference | 22,109 / 285,803 (7.7%) | Reference | 25,805 / 285,803 (9.0%) | Reference |
| History of dementia | 13,710 / 42,418 (32%) | 1.49 (1.45-1.52) | 4,158 / 13,710 (30%) | 0.84 (0.81-0.86) | 983 / 13,710 (7.2%) | 0.69 (0.65-0.74) | 609 / 13,710 (4.4%) | 0.59 (0.54-0.64) | 2,376 / 13,710 (17%) | 1.19 (1.14-1.24) |
| Chronic pulmonary disease (CPD) |  |  |  |  |  |  |  |  |  |  |
| No History of CPD | 234,508 / 2,651,794 (8.8%) | Reference | 46,709 / 234,508 (20%) | Reference | 11,694 / 234,508 (5.0%) | Reference | 17,776 / 234,508 (7.6%) | Reference | 20,775 / 234,508 (8.9%) | Reference |
| History of CPD | 65,005 / 366,852 (18%) | 1.19 (1.17-1.20) | 18,602 / 65,005 (29%) | 0.92 (0.90-0.94) | 4,464 / 65,005 (6.9%) | 0.89 (0.85-0.92) | 4,942 / 65,005 (7.6%) | 1.06 (1.02-1.10) | 7,406 / 65,005 (11%) | 1.07 (1.04-1.10) |
| Rheumatologic disease (RD) |  |  |  |  |  |  |  |  |  |  |
| No History of RD | 274,895 / 2,878,861 (9.5%) | Reference | 56,668 / 274,895 (21%) | Reference | 14,318 / 274,895 (5.2%) | Reference | 20,714 / 274,895 (7.5%) | Reference | 25,229 / 274,895 (9.2%) | Reference |
| History of RD | 24,618 / 139,785 (18%) | 0.95 (0.94-0.97) | 8,643 / 24,618 (35%) | 1.11 (1.08-1.14) | 1,840 / 24,618 (7.5%) | 1.00 (0.95-1.05) | 2,004 / 24,618 (8.1%) | 1.08 (1.03-1.14) | 2,952 / 24,618 (12%) | 1.02 (0.98-1.06) |
| Peptic ulcer disease (PUD) |  |  |  |  |  |  |  |  |  |  |
| No History of PUD | 292,694 / 2,986,771 (9.8%) | Reference | 62,879 / 292,694 (21%) | Reference | 15,673 / 292,694 (5.4%) | Reference | 22,154 / 292,694 (7.6%) | Reference | 27,383 / 292,694 (9.4%) | Reference |
| History of PUD | 6,819 / 31,875 (21%) | 1.08 (1.05-1.11) | 2,432 / 6,819 (36%) | 1.07 (1.03-1.12) | 485 / 6,819 (7.1%) | 0.93 (0.85-1.02) | 564 / 6,819 (8.3%) | 1.05 (0.96-1.14) | 798 / 6,819 (12%) | 0.97 (0.90-1.04) |
| Liver Disease |  |  |  |  |  |  |  |  |  |  |
| No History of Liver Disease | 275,696 / 2,885,760 (9.6%) | Reference | 57,790 / 275,696 (21%) | Reference | 14,814 / 275,696 (5.4%) | Reference | 20,511 / 275,696 (7.4%) | Reference | 25,622 / 275,696 (9.3%) | Reference |
| Mild Liver Disease | 17,062 / 112,409 (15%) | 0.99 (0.97-1.01) | 4,796 / 17,062 (28%) | 1.02 (0.99-1.06) | 958 / 17,062 (5.6%) | 0.92 (0.86-0.98) | 1,432 / 17,062 (8.4%) | 1.07 (1.01-1.13) | 1,529 / 17,062 (9.0%) | 1.02 (0.97-1.08) |
| Moderate to Severe Liver Disease | 6,755 / 20,477 (33%) | 1.70 (1.64-1.76) | 2,725 / 6,755 (40%) | 1.36 (1.31-1.42) | 386 / 6,755 (5.7%) | 0.87 (0.79-0.97) | 775 / 6,755 (11%) | 1.41 (1.30-1.52) | 1,030 / 6,755 (15%) | 1.71 (1.61-1.83) |
| Diabetes Mellitus |  |  |  |  |  |  |  |  |  |  |
| No History of Diabetes | 228,087 / 2,673,796 (8.5%) | Reference | 41,397 / 228,087 (18%) | Reference | 10,850 / 228,087 (4.8%) | Reference | 16,944 / 228,087 (7.4%) | Reference | 20,061 / 228,087 (8.8%) | Reference |
| Diabetes without Chronic Complications | 26,535 / 167,616 (16%) | 1.38 (1.36-1.40) | 6,034 / 26,535 (23%) | 1.06 (1.03-1.09) | 1,481 / 26,535 (5.6%) | 1.03 (0.97-1.09) | 1,915 / 26,535 (7.2%) | 1.04 (0.99-1.09) | 2,528 / 26,535 (9.5%) | 1.03 (0.98-1.07) |
| Diabetes with Chronic Complications | 44,891 / 177,234 (25%) | 1.37 (1.35-1.39) | 17,880 / 44,891 (40%) | 1.25 (1.22-1.28) | 3,827 / 44,891 (8.5%) | 1.14 (1.09-1.19) | 3,859 / 44,891 (8.6%) | 1.20 (1.15-1.25) | 5,592 / 44,891 (12%) | 1.09 (1.05-1.13) |
| Hemiplegia or paraplegia |  |  |  |  |  |  |  |  |  |  |
| No History of Hemiplegia or paraplegia | 291,851 / 2,997,719 (9.7%) | Reference | 62,855 / 291,851 (22%) | Reference | 15,525 / 291,851 (5.3%) | Reference | 22,143 / 291,851 (7.6%) | Reference | 27,264 / 291,851 (9.3%) | Reference |
| History of Hemiplegia or paraplegia | 7,662 / 20,927 (37%) | 1.91 (1.85-1.98) | 2,456 / 7,662 (32%) | 0.94 (0.90-0.98) | 633 / 7,662 (8.3%) | 0.91 (0.83-0.99) | 575 / 7,662 (7.5%) | 1.00 (0.91-1.09) | 917 / 7,662 (12%) | 1.10 (1.02-1.18) |
| Renal Disease |  |  |  |  |  |  |  |  |  |  |
| No History of Renal Disease | 231,487 / 2,776,023 (8.3%) | Reference | 35,991 / 231,487 (16%) | Reference | 10,669 / 231,487 (4.6%) | Reference | 17,360 / 231,487 (7.5%) | Reference | 19,264 / 231,487 (8.3%) | Reference |
| History of Disease Renal | 68,026 / 242,623 (28%) | 1.80 (1.78-1.82) | 29,320 / 68,026 (43%) | 2.37 (2.32-2.42) | 5,489 / 68,026 (8.1%) | 1.04 (1.0-1.08) | 5,358 / 68,026 (7.9%) | 1.05 (1.01-1.09) | 8,917 / 68,026 (13%) | 1.21 (1.17-1.25) |
| Cancer |  |  |  |  |  |  |  |  |  |  |
| No History of Malignant Cancer | 261,778 / 2,810,830 (9.3%) | Reference | 54,838 / 261,778 (21%) | Reference | 13,922 / 261,778 (5.3%) | Reference | 20,212 / 261,778 (7.7%) | Reference | 22,834 / 261,778 (8.7%) | Reference |
| Any Malignancy Except Neoplasm of skin | 30,402 / 180,441 (17%) | 0.98 (0.96-0.99) | 8,519 / 30,402 (28%) | 0.93 (0.90-0.95) | 1,861 / 30,402 (6.1%) | 0.80 (0.76-0.85) | 2,016 / 30,402 (6.6%) | 0.87 (0.83-0.92) | 3,921 / 30,402 (13%) | 1.11 (1.07-1.15) |
| Metastatic Solid Tumor | 7,333 / 27,375 (27%) | 1.88 (1.82-1.94) | 1,954 / 7,333 (27%) | 0.93 (0.89-0.98) | 375 / 7,333 (5.1%) | 0.75 (0.68-0.83) | 490 / 7,333 (6.7%) | 0.87 (0.80-0.95) | 1,426 / 7,333 (19%) | 2.11 (2.00-2.23) |
| HIV |  |  |  |  |  |  |  |  |  |  |
| No History of HIV | 297,598 / 3,007,210 (9.9%) | Reference | 64,743 / 297,598 (22%) | Reference | 16,055 / 297,598 (5.4%) | Reference | 22,571 / 297,598 (7.6%) | Reference | 28,043 / 297,598 (9.4%) | Reference |
| History of HIV | 1,915 / 11,436 (17%) | 0.99 (0.93-1.04) | 568 / 1,915 (30%) | 0.95 (0.87-1.03) | 103 / 1,915 (5.4%) | 0.93 (0.76-1.13) | 147 / 1,915 (7.7%) | 0.87 (0.74-1.03) | 138 / 1,915 (7.2%) | 1.00 (0.85-1.19) |
| Obesity |  |  |  |  |  |  |  |  |  |  |
| No History of Obesity | 199,107 / 2,196,551 (9.1%) | Reference | 38,746 / 199,107 (19%) | Reference | 10,425 / 199,107 (5.2%) | Reference | 14,634 / 199,107 (7.3%) | Reference | 19,024 / 199,107 (9.6%) | Reference |
| History of Obesity | 100,406 / 822,095 (12%) | 0.94 (0.93-0.95) | 26,565 / 100,406 (26%) | 1.05 (1.03-1.07) | 5,733 / 100,406 (5.7%) | 0.94 (0.90-0.97) | 8,084 / 100,406 (8.1%) | 1.07 (1.04-1.10) | 9,157 / 100,406 (9.1%) | 0.99 (0.96-1.01) |
| Hypertension |  |  |  |  |  |  |  |  |  |  |
| No History of Hypertension | 169,511 / 2,238,582 (7.6%) | Reference | 25,741 / 169,511 (15%) | Reference | 6,867 / 169,511 (4.1%) | Reference | 13,196 / 169,511 (7.8%) | Reference | 13,642 / 169,511 (8.0%) | Reference |
| History of Hypertension | 130,002 / 780,064 (17%) | 0.84 (0.83-0.85) | 39,570 / 130,002 (30%) | 1.13 (1.11-1.16) | 9,291 / 130,002 (7.1%) | 1.06 (1.01-1.11) | 9,522 / 130,002 (7.3%) | 0.88 (0.85-0.91) | 14,539 / 130,002 (11%) | 0.85 (0.82-0.87) |
| Former or Current Tobacco User |  |  |  |  |  |  |  |  |  |  |
| No History of Tobacco Use | 272,191 / 2,883,115 (9.4%) | Reference | 57,989 / 272,191 (21%) | Reference | 14,232 / 272,191 (5.2%) | Reference | 20,339 / 272,191 (7.5%) | Reference | 25,920 / 272,191 (9.5%) | Reference |
| History of Tobacco Use | 27,322 / 135,531 (20%) | 1.59 (1.57-1.62) | 7,322 / 27,322 (27%) | 1.10 (1.07-1.13) | 1,926 / 27,322 (7.0%) | 1.23 (1.17-1.30) | 2,379 / 27,322 (8.7%) | 0.98 (0.94-1.03) | 2,261 / 27,322 (8.3%) | 0.99 (0.94-1.03) |
| History of Substance Abuse Disorder |  |  |  |  |  |  |  |  |  |  |
| No History of Substance Abuse | 278,746 / 2,937,997 (9.5%) | Reference | 60,118 / 278,746 (22%) | Reference | 15,078 / 278,746 (5.4%) | Reference | 20,730 / 278,746 (7.4%) | Reference | 26,704 / 278,746 (9.6%) | Reference |
| History of Substance Abuse | 20,767 / 80,649 (26%) | 2.69 (2.64-2.75) | 5,193 / 20,767 (25%) | 1.01 (0.98-1.04) | 1,080 / 20,767 (5.2%) | 0.97 (0.91-1.04) | 1,988 / 20,767 (9.6%) | 1.16 (1.10-1.22) | 1,477 / 20,767 (7.1%) | 0.99 (0.93-1.05) |
| Variant Period |  |  |  |  |  |  |  |  |  |  |
| Pre-Delta (January 1, 2021 - June 14, 2021) | 72,260 / 528,579 (14%) | Reference | 15,824 / 72,260 (22%) | Reference | 4,090 / 72,260 (5.7%) | Reference | 5,839 / 72,260 (8.1%) | Reference | 7,260 / 72,260 (10%) | Reference |
| Delta (June 15, 2021 - December 21, 2021) | 87,063 / 778,430 (11%) | 0.97 (0.96-0.98) | 18,176 / 87,063 (21%) | 1.09 (1.06-1.11) | 3,781 / 87,063 (4.3%) | 0.89 (0.85-0.93) | 8,518 / 87,063 (9.8%) | 1.24 (1.20-1.29) | 9,853 / 87,063 (11%) | 1.28 (1.24-1.32) |
| Omicron (≥ December 22 ,2021) | 140,190 / 1,711,637 (8.2%) | 0.58 (0.57-0.58) | 31,311 / 140,190 (22%) | 0.95 (0.93-0.97) | 8,287 / 140,190 (5.9%) | 1.01 (0.97-1.05) | 8,361 / 140,190 (6.0%) | 0.75 (0.72-0.78) | 11,068 / 140,190 (7.9%) | 0.74 (0.72-0.76) |
| Vaccination Status Prior to SARS-CoV-2 Infection |  |  |  |  |  |  |  |  |  |  |
| No Documented COVID-19 Vaccination | 250,484 / 2,278,507 (11%) | Reference | 54,791 / 250,484 (22%) | Reference | 13,767 / 250,484 (5.5%) | Reference | 20,444 / 250,484 (8.2%) | Reference | 24,186 / 250,484 (9.7%) | Reference |
| Primary Vaccination Series | 29,348 / 434,199 (6.8%) | 0.56 (0.55-0.56) | 6,301 / 29,348 (21%) | 0.80 (0.78-0.82) | 1,398 / 29,348 (4.8%) | 0.76 (0.72-0.81) | 1,430 / 29,348 (4.9%) | 0.65 (0.61-0.68) | 2,546 / 29,348 (8.7%) | 0.70 (0.67-0.73) |
| Primary+ Vaccination Series | 19,681 / 305,940 (6.4%) | 0.47 (0.47-0.48) | 4,219 / 19,681 (21%) | 0.75 (0.73-0.78) | 993 / 19,681 (5.0%) | 0.70 (0.65-0.75) | 844 / 19,681 (4.3%) | 0.65 (0.61-0.70) | 1,449 / 19,681 (7.4%) | 0.61 (0.58-0.65) |
| Census Region |  |  |  |  |  |  |  |  |  |  |
| Midwest | 115,990 / 1,422,648 (8.2%) | Reference | 19,826 / 115,990 (17%) | Reference | 4,341 / 115,990 (3.7%) | Reference | 5,690 / 115,990 (4.9%) | Reference | 10,390 / 115,990 (9.0%) | Reference |
| Northeast | 51,533 / 370,570 (14%) | 1.26 (1.25-1.28) | 11,749 / 51,533 (23%) | 1.37 (1.34-1.41) | 3,542 / 51,533 (6.9%) | 1.63 (1.56-1.71) | 3,009 / 51,533 (5.8%) | 1.22 (1.16-1.28) | 4,611 / 51,533 (8.9%) | 0.92 (0.89-0.96) |
| South | 94,976 / 802,396 (12%) | 1.23 (1.22-1.24) | 24,402 / 94,976 (26%) | 1.53 (1.50-1.56) | 6,260 / 94,976 (6.6%) | 1.70 (1.64-1.77) | 9,607 / 94,976 (10%) | 2.17 (2.10-2.25) | 9,329 / 94,976 (9.8%) | 1.19 (1.16-1.23) |
| West | 37,014 / 423,032 (8.7%) | 0.91 (0.90-0.93) | 9,334 / 37,014 (25%) | 1.63 (1.58-1.67) | 2,015 / 37,014 (5.4%) | 1.42 (1.34-1.50) | 4,412 / 37,014 (12%) | 2.57 (2.46-2.68) | 3,851 / 37,014 (10%) | 1.16 (1.11-1.20) |
| Rural-Dwelling Status |  |  |  |  |  |  |  |  |  |  |
| Urban | 248,770 / 2,512,442 (9.9%) | Reference | 55,092 / 248,770 (22%) | Reference | 13,458 / 248,770 (5.4%) | Reference | 18,060 / 248,770 (7.3%) | Reference | 22,253 / 248,770 (8.9%) | Reference |
| Urban-Adjacent Rural | 40,330 / 408,580 (9.9%) | 1.06 (1.05-1.08) | 8,025 / 40,330 (20%) | 1.04 (1.02-1.07) | 2,115 / 40,330 (5.2%) | 1.08 (1.03-1.13) | 3,570 / 40,330 (8.9%) | 1.33 (1.28-1.38) | 4,635 / 40,330 (11%) | 1.28 (1.24-1.32) |
| Nonurban-Adjacent Rural | 10,413 / 97,624 (11%) | 1.06 (1.04-1.08) | 2,194 / 10,413 (21%) | 1.09 (1.04-1.14) | 585 / 10,413 (5.6%) | 1.08 (1.00-1.18) | 1,088 / 10,413 (10%) | 1.56 (1.47-1.66) | 1,293 / 10,413 (12%) | 1.35 (1.28-1.43) |
| **Characteristic** | **Pre-Delta Dominance, January 1, 2021 – June 14, 2021** | | | | | | | | | |
|  | **Hospitalization** | | **AKI/Dialysis** | | **MACE** | | **ECMO/IMV** | | **Death** | |
|  | **N Events / N (%)** | **Adjusted Odds Ratio (95% CI)** | **N Events / N (%)** | **Adjusted Hazard Ratio (95% CI)** | **N Events / N (%)** | **Adjusted Hazard Ratio (95% CI)** | **N Events / N (%)** | **Adjusted Hazard Ratio (95% CI)** | **N Events / N (%)** | **Adjusted Hazard Ratio (95% CI)** |
| Sex |  |  |  |  |  |  |  |  |  |  |
| Female | 35,800 / 288,700 (12%) | Reference | 6,501 / 35,800 (18%) | Reference | 1,666 / 35,800 (4.7%) | Reference | 2,535 / 288,700 (0.88%) | Reference | 3,061 / 35,800 (8.6%) | Reference |
| Male | 36,452 / 239,833 (15%) | 1.27 (1.25-1.29) | 9,321 / 36,452 (26%) | 1.47 (1.43-1.52) | 2,423 / 36,452 (6.6%) | 1.38 (1.30-1.47) | 3,809 / 239,833 (1.6%) | 1.75 (1.66-1.84) | 4,199 / 36,452 (12%) | 1.39 (1.33-1.46) |
| Age at COVID-19 Diagnosis, Median (IQR) | 72,252 / 528,533 (14%) | 1.04 (1.04-1.04) | 15,822 / 72,252 (22%) | 1.02 (1.02-1.02) | 4,089 / 72,252 (5.7%) | 1.03 (1.03-1.03) | 6,344 / 528,533 (1.2%) | 1.04 (1.04-1.04) | 7,260 / 72,252 (10%) | 1.05 (1.04-1.05) |
| Race/Ethnicity |  |  |  |  |  |  |  |  |  |  |
| White Non-Hispanic | 38,832 / 335,743 (12%) | Reference | 7,564 / 38,832 (19%) | Reference | 2,205 / 38,832 (5.7%) | Reference | 3,249 / 335,743 (0.97%) | Reference | 4,028 / 38,832 (10%) | Reference |
| Black or African American Non-Hispanic | 15,002 / 73,294 (20%) | 2.07 (2.02-2.12) | 4,485 / 15,002 (30%) | 1.72 (1.66-1.79) | 872 / 15,002 (5.8%) | 1.15 (1.06-1.25) | 1,255 / 73,294 (1.7%) | 1.69 (1.57-1.81) | 1,267 / 15,002 (8.4%) | 1.06 (0.99-1.13) |
| Hispanic or Latino Any Race | 10,808 / 57,628 (19%) | 2.10 (2.05-2.16) | 2,080 / 10,808 (19%) | 1.08 (1.03-1.14) | 483 / 10,808 (4.5%) | 0.83 (0.75-0.92) | 1,019 / 57,628 (1.8%) | 1.95 (1.81-2.10) | 1,047 / 10,808 (9.7%) | 1.26 (1.17-1.36) |
| Other | 3,486 / 24,407 (14%) | 1.47 (1.41-1.53) | 786 / 3,486 (23%) | 1.21 (1.12-1.30) | 255 / 3,486 (7.3%) | 1.17 (1.02-1.34) | 368 / 24,407 (1.5%) | 1.67 (1.50-1.87) | 416 / 3,486 (12%) | 1.28 (1.15-1.42) |
| Missing/Unknown | 4,124 / 37,461 (11%) | 1.04 (1.00-1.08) | 907 / 4,124 (22%) | 1.25 (1.16-1.34) | 274 / 4,124 (6.6%) | 1.07 (0.94-1.22) | 453 / 37,461 (1.2%) | 1.27 (1.15-1.41) | 502 / 4,124 (12%) | 1.49 (1.35-1.64) |
| Myocardial infarction (MI) |  |  |  |  |  |  |  |  |  |  |
| No History of MI | 67,977 / 517,667 (13%) | Reference | 14,106 / 67,977 (21%) | Reference | 3,240 / 67,977 (4.8%) | Reference | 5,884 / 517,667 (1.1%) | Reference | 6,598 / 67,977 (9.7%) | Reference |
| History of MI | 4,275 / 10,866 (39%) | 1.16 (1.11-1.21) | 1,716 / 4,275 (40%) | 1.03 (0.97-1.09) | 849 / 4,275 (20%) | 2.78 (2.53-3.04) | 460 / 10,866 (4.2%) | 1.11 (0.99-1.23) | 662 / 4,275 (15%) | 0.97 (0.89-1.07) |
| Congestive heart failure (CHF) |  |  |  |  |  |  |  |  |  |  |
| No History of CHF | 63,021 / 506,370 (12%) | Reference | 12,262 / 63,021 (19%) | Reference | 3,044 / 63,021 (4.8%) | Reference | 5,428 / 506,370 (1.1%) | Reference | 5,789 / 63,021 (9.2%) | Reference |
| History of CHF | 9,231 / 22,163 (42%) | 1.52 (1.47-1.58) | 3,560 / 9,231 (39%) | 1.05 (1.01-1.10) | 1,045 / 9,231 (11%) | 1.23 (1.13-1.35) | 916 / 22,163 (4.1%) | 1.31 (1.20-1.43) | 1,471 / 9,231 (16%) | 1.17 (1.09-1.26) |
| Peripheral vascular disease (PVD) |  |  |  |  |  |  |  |  |  |  |
| No History of PVD | 67,246 / 515,161 (13%) | Reference | 13,870 / 67,246 (21%) | Reference | 3,541 / 67,246 (5.3%) | Reference | 5,857 / 515,161 (1.1%) | Reference | 6,468 / 67,246 (9.6%) | Reference |
| History of PVD | 5,006 / 13,372 (37%) | 1.03 (0.99-1.08) | 1,952 / 5,006 (39%) | 0.98 (0.93-1.03) | 548 / 5,006 (11%) | 1.04 (0.94-1.15) | 487 / 13,372 (3.6%) | 0.95 (0.85-1.05) | 792 / 5,006 (16%) | 1.06 (0.97-1.15) |
| Cerebrovascular disease (CVD) |  |  |  |  |  |  |  |  |  |  |
| No History of CVD | 67,627 / 515,147 (13%) | Reference | 14,182 / 67,627 (21%) | Reference | 3,563 / 67,627 (5.3%) | Reference | 5,896 / 515,147 (1.1%) | Reference | 6,519 / 67,627 (9.6%) | Reference |
| History of CVD | 4,625 / 13,386 (35%) | 0.92 (0.88-0.97) | 1,640 / 4,625 (35%) | 0.96 (0.90-1.02) | 526 / 4,625 (11%) | 1.15 (1.04-1.28) | 448 / 13,386 (3.3%) | 0.97 (0.86-1.08) | 741 / 4,625 (16%) | 0.97 (0.89-1.06) |
| Dementia |  |  |  |  |  |  |  |  |  |  |
| No History of dementia | 69,416 / 521,925 (13%) | Reference | 14,877 / 69,416 (21%) | Reference | 3,824 / 69,416 (5.5%) | Reference | 6,186 / 521,925 (1.2%) | Reference | 6,649 / 69,416 (9.6%) | Reference |
| History of dementia | 2,836 / 6,608 (43%) | 1.33 (1.26-1.40) | 945 / 2,836 (33%) | 0.85 (0.79-0.91) | 265 / 2,836 (9.3%) | 0.79 (0.69-0.90) | 158 / 6,608 (2.4%) | 0.60 (0.51-0.71) | 611 / 2,836 (22%) | 1.17 (1.07-1.27) |
| Chronic pulmonary disease (CPD) |  |  |  |  |  |  |  |  |  |  |
| No History of CPD | 59,348 / 475,979 (12%) | Reference | 12,034 / 59,348 (20%) | Reference | 3,119 / 59,348 (5.3%) | Reference | 5,087 / 475,979 (1.1%) | Reference | 5,634 / 59,348 (9.5%) | Reference |
| History of CPD | 12,904 / 52,554 (25%) | 1.14 (1.11-1.17) | 3,788 / 12,904 (29%) | 0.92 (0.88-0.96) | 970 / 12,904 (7.5%) | 0.88 (0.81-0.96) | 1,257 / 52,554 (2.4%) | 1.21 (1.13-1.31) | 1,626 / 12,904 (13%) | 1.05 (0.98-1.12) |
| Rheumatologic disease (RD) |  |  |  |  |  |  |  |  |  |  |
| No History of RD | 67,247 / 508,913 (13%) | Reference | 14,028 / 67,247 (21%) | Reference | 3,645 / 67,247 (5.4%) | Reference | 5,863 / 508,913 (1.2%) | Reference | 6,645 / 67,247 (9.9%) | Reference |
| History of RD | 5,005 / 19,620 (26%) | 0.99 (0.95-1.03) | 1,794 / 5,005 (36%) | 1.11 (1.05-1.17) | 444 / 5,005 (8.9%) | 1.12 (1.01-1.24) | 481 / 19,620 (2.5%) | 0.98 (0.89-1.08) | 615 / 5,005 (12%) | 0.94 (0.86-1.03) |
| Peptic ulcer disease (PUD) |  |  |  |  |  |  |  |  |  |  |
| No History of PUD | 71,003 / 524,333 (14%) | Reference | 15,389 / 71,003 (22%) | Reference | 3,992 / 71,003 (5.6%) | Reference | 6,218 / 524,333 (1.2%) | Reference | 7,105 / 71,003 (10%) | Reference |
| History of PUD | 1,249 / 4,200 (30%) | 1.11 (1.03-1.20) | 433 / 1,249 (35%) | 1.00 (0.91-1.11) | 97 / 1,249 (7.8%) | 0.90 (0.73-1.11) | 126 / 4,200 (3.0%) | 1.06 (0.88-1.27) | 155 / 1,249 (12%) | 0.89 (0.75-1.04) |
| Liver Disease |  |  |  |  |  |  |  |  |  |  |
| No History of Liver Disease | 67,532 / 509,136 (13%) | Reference | 14,295 / 67,532 (21%) | Reference | 3,803 / 67,532 (5.6%) | Reference | 5,767 / 509,136 (1.1%) | Reference | 6,720 / 67,532 (10%) | Reference |
| Mild Liver Disease | 3,465 / 16,200 (21%) | 0.98 (0.94-1.03) | 995 / 3,465 (29%) | 1.07 (1.00-1.14) | 200 / 3,465 (5.8%) | 0.89 (0.77-1.03) | 374 / 16,200 (2.3%) | 1.21 (1.09-1.35) | 322 / 3,465 (9.3%) | 1.00 (0.89-1.12) |
| Moderate to Severe Liver Disease | 1,255 / 3,197 (39%) | 1.48 (1.37-1.61) | 532 / 1,255 (42%) | 1.47 (1.34-1.61) | 86 / 1,255 (6.9%) | 0.96 (0.77-1.19) | 203 / 3,197 (6.3%) | 2.02 (1.74-2.34) | 218 / 1,255 (17%) | 1.77 (1.54-2.04) |
| Diabetes Mellitus |  |  |  |  |  |  |  |  |  |  |
| No History of Diabetes | 55,754 / 470,634 (12%) | Reference | 10,215 / 55,754 (18%) | Reference | 2,788 / 55,754 (5.0%) | Reference | 4,706 / 470,634 (1.0%) | Reference | 5,183 / 55,754 (9.3%) | Reference |
| Diabetes without Chronic Complications | 6,566 / 28,973 (23%) | 1.32 (1.28-1.36) | 1,543 / 6,566 (23%) | 1.09 (1.03-1.15) | 391 / 6,566 (6.0%) | 1.03 (0.92-1.15) | 573 / 28,973 (2.0%) | 1.35 (1.23-1.48) | 659 / 6,566 (10%) | 1.04 (0.96-1.14) |
| Diabetes with Chronic Complications | 9,932 / 28,926 (34%) | 1.34 (1.30-1.39) | 4,064 / 9,932 (41%) | 1.26 (1.21-1.32) | 910 / 9,932 (9.2%) | 1.11 (1.01-1.22) | 1,065 / 28,926 (3.7%) | 1.52 (1.40-1.66) | 1,418 / 9,932 (14%) | 1.15 (1.07-1.24) |
| Hemiplegia or paraplegia |  |  |  |  |  |  |  |  |  |  |
| No History of Hemiplegia or paraplegia | 70,840 / 525,416 (13%) | Reference | 15,316 / 70,840 (22%) | Reference | 3,955 / 70,840 (5.6%) | Reference | 6,200 / 525,416 (1.2%) | Reference | 7,047 / 70,840 (10%) | Reference |
| History of Hemiplegia or paraplegia | 1,412 / 3,117 (45%) | 1.81 (1.67-1.97) | 506 / 1,412 (36%) | 1.01 (0.91-1.11) | 134 / 1,412 (9.5%) | 0.92 (0.76-1.11) | 144 / 3,117 (4.6%) | 1.37 (1.14-1.65) | 213 / 1,412 (15%) | 1.18 (1.01-1.37) |
| Renal Disease |  |  |  |  |  |  |  |  |  |  |
| No History of Renal Disease | 58,487 / 492,033 (12%) | Reference | 9,551 / 58,487 (16%) | Reference | 2,797 / 58,487 (4.8%) | Reference | 4,978 / 492,033 (1.0%) | Reference | 5,164 / 58,487 (8.8%) | Reference |
| History of Disease Renal | 13,765 / 36,500 (38%) | 1.69 (1.64-1.75) | 6,271 / 13,765 (46%) | 2.32 (2.22-2.42) | 1,292 / 13,765 (9.4%) | 1.14 (1.04-1.24) | 1,366 / 36,500 (3.7%) | 1.44 (1.33-1.56) | 2,096 / 13,765 (15%) | 1.24 (1.16-1.32) |
| Cancer |  |  |  |  |  |  |  |  |  |  |
| No History of Malignant Cancer | 64,932 / 500,329 (13%) | Reference | 13,765 / 64,932 (21%) | Reference | 3,608 / 64,932 (5.6%) | Reference | 5,741 / 500,329 (1.1%) | Reference | 6,169 / 64,932 (9.5%) | Reference |
| Any Malignancy Except Neoplasm of skin | 6,044 / 24,311 (25%) | 0.98 (0.95-1.01) | 1,716 / 6,044 (28%) | 0.91 (0.87-0.96) | 400 / 6,044 (6.6%) | 0.78 (0.70-0.87) | 490 / 24,311 (2.0%) | 0.81 (0.74-0.90) | 846 / 6,044 (14%) | 1.03 (0.95-1.11) |
| Metastatic Solid Tumor | 1,276 / 3,893 (33%) | 1.56 (1.45-1.68) | 341 / 1,276 (27%) | 0.91 (0.82-1.01) | 81 / 1,276 (6.3%) | 0.83 (0.67-1.04) | 113 / 3,893 (2.9%) | 1.15 (0.95-1.38) | 245 / 1,276 (19%) | 1.75 (1.54-2.00) |
| HIV |  |  |  |  |  |  |  |  |  |  |
| No History of HIV | 71,814 / 526,484 (14%) | Reference | 15,680 / 71,814 (22%) | Reference | 4,063 / 71,814 (5.7%) | Reference | 6,296 / 526,484 (1.2%) | Reference | 7,215 / 71,814 (10%) | Reference |
| History of HIV | 438 / 2,049 (21%) | 0.84 (0.75-0.94) | 142 / 438 (32%) | 0.97 (0.82-1.15) | 26 / 438 (5.9%) | 0.97 (0.66-1.43) | 48 / 2,049 (2.3%) | 0.87 (0.65-1.16) | 45 / 438 (10%) | 1.28 (0.95-1.72) |
| Obesity |  |  |  |  |  |  |  |  |  |  |
| No History of Obesity | 47,569 / 392,515 (12%) | Reference | 9,648 / 47,569 (20%) | Reference | 2,644 / 47,569 (5.6%) | Reference | 4,171 / 392,515 (1.1%) | Reference | 4,951 / 47,569 (10%) | Reference |
| History of Obesity | 24,683 / 136,018 (18%) | 1.09 (1.07-1.11) | 6,174 / 24,683 (25%) | 0.96 (0.92-0.99) | 1,445 / 24,683 (5.9%) | 0.92 (0.86-0.99) | 2,173 / 136,018 (1.6%) | 0.94 (0.89-1.00) | 2,309 / 24,683 (9.4%) | 0.97 (0.91-1.02) |
| Hypertension |  |  |  |  |  |  |  |  |  |  |
| No History of Hypertension | 43,538 / 407,525 (11%) | Reference | 6,911 / 43,538 (16%) | Reference | 1,874 / 43,538 (4.3%) | Reference | 3,814 / 407,525 (0.94%) | Reference | 3,748 / 43,538 (8.6%) | Reference |
| History of Hypertension | 28,714 / 121,008 (24%) | 0.82 (0.80-0.84) | 8,911 / 28,714 (31%) | 1.11 (1.06-1.16) | 2,215 / 28,714 (7.7%) | 1.04 (0.96-1.13) | 2,530 / 121,008 (2.1%) | 0.74 (0.69-0.79) | 3,512 / 28,714 (12%) | 0.81 (0.76-0.87) |
| Former or Current Tobacco User |  |  |  |  |  |  |  |  |  |  |
| No History of Tobacco Use | 68,192 / 512,818 (13%) | Reference | 14,689 / 68,192 (22%) | Reference | 3,804 / 68,192 (5.6%) | Reference | 5,907 / 512,818 (1.2%) | Reference | 6,898 / 68,192 (10%) | Reference |
| History of Tobacco Use | 4,060 / 15,715 (26%) | 1.46 (1.40-1.52) | 1,133 / 4,060 (28%) | 1.07 (1.00-1.14) | 285 / 4,060 (7.0%) | 1.13 (1.00-1.29) | 437 / 15,715 (2.8%) | 1.26 (1.13-1.40) | 362 / 4,060 (8.9%) | 0.98 (0.88-1.10) |
| History of Substance Abuse Disorder |  |  |  |  |  |  |  |  |  |  |
| No History of Substance Abuse | 68,618 / 517,180 (13%) | Reference | 14,896 / 68,618 (22%) | Reference | 3,896 / 68,618 (5.7%) | Reference | 5,937 / 517,180 (1.1%) | Reference | 7,000 / 68,618 (10%) | Reference |
| History of Substance Abuse | 3,634 / 11,353 (32%) | 2.62 (2.50-2.75) | 926 / 3,634 (25%) | 1.01 (0.94-1.09) | 193 / 3,634 (5.3%) | 0.96 (0.83-1.13) | 407 / 11,353 (3.6%) | 2.02 (1.80-2.26) | 260 / 3,634 (7.2%) | 0.91 (0.80-1.04) |
| Vaccination Status Prior to SARS-CoV-2 Infection |  |  |  |  |  |  |  |  |  |  |
| No Documented COVID-19 Vaccination | 71,349 / 522,511 (14%) | Reference | 15,649 / 71,349 (22%) | Reference | 4,047 / 71,349 (5.7%) | Reference | 6,278 / 522,511 (1.2%) | Reference | 7,188 / 71,349 (10%) | Reference |
| Primary Vaccination Series | 903 / 6,022 (15%) | 0.69 (0.64-0.75) | 173 / 903 (19%) | 0.67 (0.57-0.77) | 42 / 903 (4.7%) | 0.71 (0.52-0.96) | 66 / 6,022 (1.1%) | 0.62 (0.49-0.79) | 72 / 903 (8.0%) | 0.54 (0.43-0.68) |
| Census Region |  |  |  |  |  |  |  |  |  |  |
| Midwest | 26,344 / 252,527 (10%) | Reference | 4,060 / 26,344 (15%) | Reference | 802 / 26,344 (3.0%) | Reference | 1,143 / 252,527 (0.45%) | Reference | 2,205 / 26,344 (8.4%) | Reference |
| Northeast | 18,368 / 96,138 (19%) | 1.39 (1.36-1.42) | 4,497 / 18,368 (24%) | 1.71 (1.64-1.79) | 1,532 / 18,368 (8.3%) | 2.70 (2.46-2.95) | 1,289 / 96,138 (1.3%) | 2.18 (2.00-2.37) | 1,969 / 18,368 (11%) | 1.11 (1.04-1.19) |
| South | 19,557 / 120,283 (16%) | 1.23 (1.20-1.25) | 5,264 / 19,557 (27%) | 1.74 (1.67-1.81) | 1,307 / 19,557 (6.7%) | 2.08 (1.90-2.28) | 2,646 / 120,283 (2.2%) | 3.95 (3.68-4.24) | 2,158 / 19,557 (11%) | 1.35 (1.27-1.43) |
| West | 7,983 / 59,585 (13%) | 1.07 (1.04-1.10) | 2,001 / 7,983 (25%) | 1.87 (1.77-1.98) | 448 / 7,983 (5.6%) | 1.91 (1.70-2.16) | 1,266 / 59,585 (2.1%) | 4.08 (3.75-4.44) | 928 / 7,983 (12%) | 1.34 (1.24-1.45) |
| Rural-Dwelling Status |  |  |  |  |  |  |  |  |  |  |
| Urban | 63,122 / 448,083 (14%) | Reference | 13,818 / 63,122 (22%) | Reference | 3,578 / 63,122 (5.7%) | Reference | 5,254 / 448,083 (1.2%) | Reference | 6,144 / 63,122 (9.7%) | Reference |
| Urban-Adjacent Rural | 7,335 / 64,881 (11%) | 0.90 (0.88-0.93) | 1,600 / 7,335 (22%) | 1.18 (1.12-1.24) | 413 / 7,335 (5.6%) | 1.17 (1.05-1.30) | 846 / 64,881 (1.3%) | 1.44 (1.33-1.55) | 891 / 7,335 (12%) | 1.31 (1.22-1.41) |
| Nonurban-Adjacent Rural | 1,795 / 15,569 (12%) | 0.90 (0.85-0.94) | 404 / 1,795 (23%) | 1.18 (1.07-1.30) | 98 / 1,795 (5.5%) | 1.07 (0.88-1.31) | 244 / 15,569 (1.6%) | 1.78 (1.56-2.03) | 225 / 1,795 (13%) | 1.35 (1.18-1.54) |
| **Characteristic** | **Delta Dominance, June 15, 2021 – December 21, 2021** | | | | | | | | | |
|  | **Hospitalization** | | **AKI/Dialysis** | | **MACE** | | **ECMO/IMV** | | **Death** | |
|  | **N Events / N (%)** | **Adjusted Odds Ratio (95% CI)** | **N Events / N (%)** | **Adjusted Hazard Ratio (95% CI)** | **N Events / N (%)** | **Adjusted Hazard Ratio (95% CI)** | **N Events / N (%)** | **Adjusted Hazard Ratio (95% CI)** | **N Events / N (%)** | **Adjusted Hazard Ratio (95% CI)** |
| Sex |  |  |  |  |  |  |  |  |  |  |
| Female | 42,903 / 429,152 (10%) | Reference | 7,361 / 42,903 (17%) | Reference | 1,506 / 42,903 (3.5%) | Reference | 3,493 / 42,903 (8.1%) | Reference | 4,135 / 42,903 (9.6%) | Reference |
| Male | 44,160 / 349,278 (13%) | 1.25 (1.23-1.27) | 10,815 / 44,160 (24%) | 1.54 (1.49-1.59) | 2,275 / 44,160 (5.2%) | 1.43 (1.34-1.52) | 5,025 / 44,160 (11%) | 1.42 (1.36-1.48) | 5,718 / 44,160 (13%) | 1.38 (1.32-1.43) |
| Age at COVID-19 Diagnosis | 87,063 / 778,430 (11%) | 1.04 (1.04-1.04) | 18,176 / 87,063 (21%) | 1.02 (1.02-1.02) | 3,781 / 87,063 (4.3%) | 1.03 (1.02-1.03) | 8,518 / 87,063 (9.8%) | 1.00 (1.00-1.00) | 9,853 / 87,063 (11%) | 1.04 (1.04-1.04) |
| Race/Ethnicity |  |  |  |  |  |  |  |  |  |  |
| White Non-Hispanic | 59,353 / 565,119 (11%) | Reference | 11,206 / 59,353 (19%) | Reference | 2,478 / 59,353 (4.2%) | Reference | 5,521 / 59,353 (9.3%) | Reference | 7,217 / 59,353 (12%) | Reference |
| Black or African American Non-Hispanic | 14,764 / 94,782 (16%) | 1.81 (1.77-1.85) | 4,460 / 14,764 (30%) | 1.80 (1.73-1.86) | 755 / 14,764 (5.1%) | 1.37 (1.26-1.49) | 1,365 / 14,764 (9.2%) | 1.00 (0.94-1.06) | 1,277 / 14,764 (8.6%) | 0.94 (0.88-1.00) |
| Hispanic or Latino Any Race | 8,029 / 55,839 (14%) | 1.93 (1.88-1.99) | 1,475 / 8,029 (18%) | 1.11 (1.04-1.17) | 277 / 8,029 (3.4%) | 1.04 (0.91-1.19) | 961 / 8,029 (12%) | 1.15 (1.07-1.24) | 758 / 8,029 (9.4%) | 1.09 (1.01-1.18) |
| Other | 1,943 / 26,580 (7.3%) | 0.85 (0.80-0.89) | 408 / 1,943 (21%) | 1.25 (1.13-1.38) | 99 / 1,943 (5.1%) | 1.42 (1.16-1.74) | 239 / 1,943 (12%) | 1.31 (1.15-1.49) | 235 / 1,943 (12%) | 1.22 (1.07-1.39) |
| Missing/Unknown | 2,974 / 36,110 (8.2%) | 0.90 (0.86-0.94) | 627 / 2,974 (21%) | 1.25 (1.16-1.36) | 172 / 2,974 (5.8%) | 1.57 (1.34-1.83) | 432 / 2,974 (15%) | 1.40 (1.27-1.55) | 366 / 2,974 (12%) | 1.36 (1.22-1.51) |
| Myocardial infarction (MI) |  |  |  |  |  |  |  |  |  |  |
| No History of MI | 82,181 / 763,573 (11%) | Reference | 16,336 / 82,181 (20%) | Reference | 3,033 / 82,181 (3.7%) | Reference | 7,995 / 82,181 (9.7%) | Reference | 8,939 / 82,181 (11%) | Reference |
| History of MI | 4,882 / 14,857 (33%) | 1.10 (1.05-1.14) | 1,840 / 4,882 (38%) | 0.95 (0.90-1.00) | 748 / 4,882 (15%) | 2.62 (2.38-2.89) | 523 / 4,882 (11%) | 0.96 (0.87-1.06) | 914 / 4,882 (19%) | 1.05 (0.97-1.13) |
| Congestive heart failure (CHF) |  |  |  |  |  |  |  |  |  |  |
| No History of CHF | 76,963 / 749,851 (10%) | Reference | 14,329 / 76,963 (19%) | Reference | 2,861 / 76,963 (3.7%) | Reference | 7,493 / 76,963 (9.7%) | Reference | 8,041 / 76,963 (10%) | Reference |
| History of CHF | 10,100 / 28,579 (35%) | 1.49 (1.44-1.54) | 3,847 / 10,100 (38%) | 1.08 (1.03-1.13) | 920 / 10,100 (9.1%) | 1.28 (1.16-1.41) | 1,025 / 10,100 (10%) | 1.01 (0.94-1.10) | 1,812 / 10,100 (18%) | 1.10 (1.03-1.17) |
| Peripheral vascular disease (PVD) |  |  |  |  |  |  |  |  |  |  |
| No History of PVD | 81,276 / 760,852 (11%) | Reference | 16,015 / 81,276 (20%) | Reference | 3,298 / 81,276 (4.1%) | Reference | 7,915 / 81,276 (9.7%) | Reference | 8,823 / 81,276 (11%) | Reference |
| History of PVD | 5,787 / 17,578 (33%) | 1.13 (1.09-1.18) | 2,161 / 5,787 (37%) | 0.98 (0.93-1.03) | 483 / 5,787 (8.3%) | 1.02 (0.91-1.14) | 603 / 5,787 (10%) | 1.05 (0.95-1.15) | 1,030 / 5,787 (18%) | 1.01 (0.94-1.09) |
| Cerebrovascular disease (CVD) |  |  |  |  |  |  |  |  |  |  |
| No History of CVD | 81,843 / 760,673 (11%) | Reference | 16,367 / 81,843 (20%) | Reference | 3,328 / 81,843 (4.1%) | Reference | 8,046 / 81,843 (9.8%) | Reference | 8,952 / 81,843 (11%) | Reference |
| History of CVD | 5,220 / 17,757 (29%) | 0.96 (0.92-1.00) | 1,809 / 5,220 (35%) | 0.99 (0.94-1.05) | 453 / 5,220 (8.7%) | 1.08 (0.96-1.21) | 472 / 5,220 (9.0%) | 0.93 (0.83-1.03) | 901 / 5,220 (17%) | 0.93 (0.86-1.01) |
| Dementia |  |  |  |  |  |  |  |  |  |  |
| No History of dementia | 84,624 / 771,146 (11%) | Reference | 17,410 / 84,624 (21%) | Reference | 3,609 / 84,624 (4.3%) | Reference | 8,379 / 84,624 (9.9%) | Reference | 9,304 / 84,624 (11%) | Reference |
| History of dementia | 2,439 / 7,284 (33%) | 1.27 (1.20-1.35) | 766 / 2,439 (31%) | 0.83 (0.77-0.89) | 172 / 2,439 (7.1%) | 0.75 (0.64-0.88) | 139 / 2,439 (5.7%) | 0.55 (0.46-0.65) | 549 / 2,439 (23%) | 1.13 (1.03-1.24) |
| Chronic pulmonary disease (CPD) |  |  |  |  |  |  |  |  |  |  |
| No History of CPD | 71,416 / 701,104 (10%) | Reference | 13,752 / 71,416 (19%) | Reference | 2,858 / 71,416 (4.0%) | Reference | 7,024 / 71,416 (9.8%) | Reference | 7,559 / 71,416 (11%) | Reference |
| History of CPD | 15,647 / 77,326 (20%) | 1.19 (1.16-1.21) | 4,424 / 15,647 (28%) | 0.91 (0.88-0.95) | 923 / 15,647 (5.9%) | 0.89 (0.81-0.97) | 1,494 / 15,647 (9.5%) | 0.99 (0.93-1.06) | 2,294 / 15,647 (15%) | 1.07 (1.02-1.13) |
| Rheumatologic disease (RD) |  |  |  |  |  |  |  |  |  |  |
| No History of RD | 81,053 / 749,925 (11%) | Reference | 16,062 / 81,053 (20%) | Reference | 3,393 / 81,053 (4.2%) | Reference | 7,865 / 81,053 (9.7%) | Reference | 8,884 / 81,053 (11%) | Reference |
| History of RD | 6,010 / 28,505 (21%) | 1.00 (0.97-1.03) | 2,114 / 6,010 (35%) | 1.11 (1.06-1.16) | 388 / 6,010 (6.5%) | 0.99 (0.88-1.10) | 653 / 6,010 (11%) | 1.11 (1.02-1.21) | 969 / 6,010 (16%) | 1.07 (1.00-1.15) |
| Peptic ulcer disease (PUD) |  |  |  |  |  |  |  |  |  |  |
| No History of PUD | 85,609 / 772,173 (11%) | Reference | 17,694 / 85,609 (21%) | Reference | 3,683 / 85,609 (4.3%) | Reference | 8,355 / 85,609 (9.8%) | Reference | 9,623 / 85,609 (11%) | Reference |
| History of PUD | 1,454 / 6,257 (23%) | 1.00 (0.93-1.06) | 482 / 1,454 (33%) | 0.96 (0.87-1.05) | 98 / 1,454 (6.7%) | 1.00 (0.82-1.23) | 163 / 1,454 (11%) | 1.11 (0.94-1.30) | 230 / 1,454 (16%) | 1.01 (0.88-1.15) |
| Liver Disease |  |  |  |  |  |  |  |  |  |  |
| No History of Liver Disease | 81,397 / 750,884 (11%) | Reference | 16,392 / 81,397 (20%) | Reference | 3,490 / 81,397 (4.3%) | Reference | 7,871 / 81,397 (9.7%) | Reference | 9,078 / 81,397 (11%) | Reference |
| Mild Liver Disease | 4,273 / 23,647 (18%) | 1.02 (0.98-1.05) | 1,210 / 4,273 (28%) | 1.03 (0.97-1.09) | 220 / 4,273 (5.1%) | 0.94 (0.82-1.08) | 469 / 4,273 (11%) | 1.02 (0.93-1.13) | 515 / 4,273 (12%) | 1.03 (0.94-1.13) |
| Moderate to Severe Liver Disease | 1,393 / 3,899 (36%) | 1.65 (1.53-1.78) | 574 / 1,393 (41%) | 1.33 (1.22-1.45) | 71 / 1,393 (5.1%) | 0.84 (0.66-1.06) | 178 / 1,393 (13%) | 1.16 (0.99-1.35) | 260 / 1,393 (19%) | 1.49 (1.31-1.69) |
| Diabetes Mellitus |  |  |  |  |  |  |  |  |  |  |
| No History of Diabetes | 68,528 / 702,439 (9.8%) | Reference | 12,133 / 68,528 (18%) | Reference | 2,614 / 68,528 (3.8%) | Reference | 6,555 / 68,528 (9.6%) | Reference | 7,131 / 68,528 (10%) | Reference |
| Diabetes without Chronic Complications | 7,607 / 38,999 (20%) | 1.49 (1.44-1.53) | 1,739 / 7,607 (23%) | 1.05 (0.99-1.10) | 370 / 7,607 (4.9%) | 1.08 (0.96-1.21) | 736 / 7,607 (9.7%) | 1.06 (0.98-1.15) | 934 / 7,607 (12%) | 1.06 (0.99-1.14) |
| Diabetes with Chronic Complications | 10,928 / 36,992 (30%) | 1.46 (1.41-1.50) | 4,304 / 10,928 (39%) | 1.23 (1.18-1.28) | 797 / 10,928 (7.3%) | 1.11 (1.01-1.23) | 1,227 / 10,928 (11%) | 1.20 (1.11-1.29) | 1,788 / 10,928 (16%) | 1.11 (1.04-1.19) |
| Hemiplegia or paraplegia |  |  |  |  |  |  |  |  |  |  |
| No History of Hemiplegia or paraplegia | 85,532 / 774,477 (11%) | Reference | 17,658 / 85,532 (21%) | Reference | 3,655 / 85,532 (4.3%) | Reference | 8,384 / 85,532 (9.8%) | Reference | 9,622 / 85,532 (11%) | Reference |
| History of Hemiplegia or paraplegia | 1,531 / 3,953 (39%) | 1.67 (1.55-1.81) | 518 / 1,531 (34%) | 0.93 (0.85-1.03) | 126 / 1,531 (8.2%) | 1.02 (0.84-1.25) | 134 / 1,531 (8.8%) | 0.86 (0.72-1.04) | 231 / 1,531 (15%) | 1.0 (0.86-1.15) |
| Renal Disease |  |  |  |  |  |  |  |  |  |  |
| No History of Renal Disease | 71,671 / 729,314 (9.8%) | Reference | 11,421 / 71,671 (16%) | Reference | 2,649 / 71,671 (3.7%) | Reference | 6,923 / 71,671 (9.7%) | Reference | 7,181 / 71,671 (10%) | Reference |
| History of Disease Renal | 15,392 / 49,116 (31%) | 1.68 (1.63-1.72) | 6,755 / 15,392 (44%) | 2.21 (2.13-2.30) | 1,132 / 15,392 (7.4%) | 1.11 (1.01-1.22) | 1,595 / 15,392 (10%) | 1.05 (0.98-1.13) | 2,672 / 15,392 (17%) | 1.23 (1.16-1.30) |
| Cancer |  |  |  |  |  |  |  |  |  |  |
| No History of Malignant Cancer | 78,624 / 739,666 (11%) | Reference | 15,810 / 78,624 (20%) | Reference | 3,313 / 78,624 (4.2%) | Reference | 7,806 / 78,624 (9.9%) | Reference | 8,372 / 78,624 (11%) | Reference |
| Any Malignancy Except Neoplasm of skin | 6,959 / 33,850 (21%) | 0.98 (0.95-1.01) | 1,986 / 6,959 (29%) | 0.93 (0.89-0.98) | 391 / 6,959 (5.6%) | 0.89 (0.79-0.99) | 576 / 6,959 (8.3%) | 0.82 (0.75-0.90) | 1,141 / 6,959 (16%) | 1.05 (0.98-1.12) |
| Metastatic Solid Tumor | 1,480 / 4,914 (30%) | 1.69 (1.58-1.81) | 380 / 1,480 (26%) | 0.83 (0.75-0.92) | 77 / 1,480 (5.2%) | 0.85 (0.68-1.07) | 136 / 1,480 (9.2%) | 0.86 (0.72-1.02) | 340 / 1,480 (23%) | 1.75 (1.56-1.95) |
| HIV |  |  |  |  |  |  |  |  |  |  |
| No History of HIV | 86,658 / 776,064 (11%) | Reference | 18,048 / 86,658 (21%) | Reference | 3,759 / 86,658 (4.3%) | Reference | 8,480 / 86,658 (9.8%) | Reference | 9,822 / 86,658 (11%) | Reference |
| History of HIV | 405 / 2,366 (17%) | 0.88 (0.78-0.99) | 128 / 405 (32%) | 0.92 (0.77-1.09) | 22 / 405 (5.4%) | 0.94 (0.61-1.43) | 38 / 405 (9.4%) | 0.84 (0.61-1.15) | 31 / 405 (7.7%) | 0.79 (0.56-1.13) |
| Obesity |  |  |  |  |  |  |  |  |  |  |
| No History of Obesity | 59,319 / 591,292 (10%) | Reference | 10,688 / 59,319 (18%) | Reference | 2,435 / 59,319 (4.1%) | Reference | 5,355 / 59,319 (9.0%) | Reference | 6,540 / 59,319 (11%) | Reference |
| History of Obesity | 27,744 / 187,138 (15%) | 1.03 (1.01-1.05) | 7,488 / 27,744 (27%) | 1.13 (1.09-1.17) | 1,346 / 27,744 (4.9%) | 0.92 (0.86-1.00) | 3,163 / 27,744 (11%) | 1.21 (1.15-1.28) | 3,313 / 27,744 (12%) | 1.08 (1.02-1.13) |
| Hypertension |  |  |  |  |  |  |  |  |  |  |
| No History of Hypertension | 53,952 / 610,715 (8.8%) | Reference | 8,053 / 53,952 (15%) | Reference | 1,743 / 53,952 (3.2%) | Reference | 5,277 / 53,952 (9.8%) | Reference | 5,110 / 53,952 (9.5%) | Reference |
| History of Hypertension | 33,111 / 167,715 (20%) | 0.91 (0.89-0.93) | 10,123 / 33,111 (31%) | 1.23 (1.18-1.28) | 2,038 / 33,111 (6.2%) | 1.14 (1.04-1.24) | 3,241 / 33,111 (9.8%) | 0.95 (0.89-1.00) | 4,743 / 33,111 (14%) | 0.91 (0.86-0.96) |
| Former or Current Tobacco User |  |  |  |  |  |  |  |  |  |  |
| No History of Tobacco Use | 80,265 / 745,165 (11%) | Reference | 16,336 / 80,265 (20%) | Reference | 3,369 / 80,265 (4.2%) | Reference | 7,784 / 80,265 (9.7%) | Reference | 9,119 / 80,265 (11%) | Reference |
| History of Tobacco Use | 6,798 / 33,265 (20%) | 1.36 (1.31-1.40) | 1,840 / 6,798 (27%) | 1.07 (1.01-1.12) | 412 / 6,798 (6.1%) | 1.16 (1.04-1.30) | 734 / 6,798 (11%) | 0.91 (0.84-0.99) | 734 / 6,798 (11%) | 0.97 (0.89-1.05) |
| History of Substance Abuse Disorder |  |  |  |  |  |  |  |  |  |  |
| No History of Substance Abuse | 82,054 / 758,547 (11%) | Reference | 16,928 / 82,054 (21%) | Reference | 3,534 / 82,054 (4.3%) | Reference | 7,959 / 82,054 (9.7%) | Reference | 9,385 / 82,054 (11%) | Reference |
| History of Substance Abuse | 5,009 / 19,883 (25%) | 2.27 (2.18-2.36) | 1,248 / 5,009 (25%) | 0.99 (0.93-1.06) | 247 / 5,009 (4.9%) | 1.06 (0.92-1.22) | 559 / 5,009 (11%) | 1.04 (0.95-1.15) | 468 / 5,009 (9.3%) | 0.98 (0.89-1.08) |
| Vaccination Status Prior to SARS-CoV-2 Infection |  |  |  |  |  |  |  |  |  |  |
| No Documented COVID-19 Vaccination | 76,753 / 635,785 (12%) | Reference | 15,909 / 76,753 (21%) | Reference | 3,341 / 76,753 (4.4%) | Reference | 7,997 / 76,753 (10%) | Reference | 8,713 / 76,753 (11%) | Reference |
| Primary Vaccination Series | 9,591 / 134,768 (7.1%) | 0.37 (0.36-0.38) | 2,066 / 9,591 (22%) | 0.77 (0.73-0.81) | 415 / 9,591 (4.3%) | 0.74 (0.67-0.83) | 482 / 9,591 (5.0%) | 0.52 (0.47-0.57) | 1,054 / 9,591 (11%) | 0.60 (0.56-0.64) |
| Primary+ Vaccination Series | 719 / 7,877 (9.1%) | 0.39 (0.36-0.42) | 201 / 719 (28%) | 0.91 (0.79-1.05) | 25 / 719 (3.5%) | 0.58 (0.39-0.86) | 39 / 719 (5.4%) | 0.54 (0.40-0.74) | 86 / 719 (12%) | 0.60 (0.49-0.74) |
| Census Region |  |  |  |  |  |  |  |  |  |  |
| Midwest | 41,214 / 436,523 (9.4%) | Reference | 6,417 / 41,214 (16%) | Reference | 1,227 / 41,214 (3.0%) | Reference | 2,380 / 41,214 (5.8%) | Reference | 4,260 / 41,214 (10%) | Reference |
| Northeast | 8,113 / 62,934 (13%) | 0.96 (0.94-0.99) | 1,798 / 8,113 (22%) | 1.43 (1.35-1.51) | 447 / 8,113 (5.5%) | 1.64 (1.47-1.84) | 668 / 8,113 (8.2%) | 1.38 (1.27-1.51) | 815 / 8,113 (10%) | 0.86 (0.80-0.93) |
| South | 27,543 / 196,433 (14%) | 1.19 (1.17-1.21) | 7,320 / 27,543 (27%) | 1.77 (1.71-1.84) | 1,612 / 27,543 (5.9%) | 1.95 (1.81-2.11) | 3,755 / 27,543 (14%) | 2.39 (2.27-2.52) | 3,389 / 27,543 (12%) | 1.26 (1.20-1.32) |
| West | 10,193 / 82,540 (12%) | 1.06 (1.04-1.09) | 2,641 / 10,193 (26%) | 1.88 (1.79-1.97) | 495 / 10,193 (4.9%) | 1.58 (1.42-1.77) | 1,715 / 10,193 (17%) | 3.00 (2.81-3.21) | 1,389 / 10,193 (14%) | 1.32 (1.24-1.41) |
| Rural-Dwelling Status |  |  |  |  |  |  |  |  |  |  |
| Urban | 68,550 / 607,961 (11%) | Reference | 14,698 / 68,550 (21%) | Reference | 3,010 / 68,550 (4.4%) | Reference | 6,406 / 68,550 (9.3%) | Reference | 7,264 / 68,550 (11%) | Reference |
| Urban-Adjacent Rural | 14,695 / 136,429 (11%) | 1.04 (1.02-1.06) | 2,696 / 14,695 (18%) | 1.02 (0.98-1.07) | 621 / 14,695 (4.2%) | 1.08 (0.99-1.18) | 1,614 / 14,695 (11%) | 1.37 (1.29-1.45) | 1,999 / 14,695 (14%) | 1.29 (1.22-1.36) |
| Nonurban-Adjacent Rural | 3,818 / 34,040 (11%) | 1.03 (1.00-1.07) | 782 / 3,818 (20%) | 1.14 (1.06-1.23) | 150 / 3,818 (3.9%) | 0.94 (0.80-1.11) | 498 / 3,818 (13%) | 1.63 (1.48-1.79) | 590 / 3,818 (15%) | 1.42 (1.31-1.55) |
| **Characteristic** | **Omicron Dominance, December 22, 2021 – December 31, 2022** | | | | | | | | | |
|  | **Hospitalization** | | **AKI/Dialysis** | | **MACE** | | **ECMO/IMV** | | **Death** | |
|  | **N Events / N (%)** | **Adjusted Odds Ratio (95% CI)** | **N Events / N (%)** | **Adjusted Hazard Ratio (95% CI)** | **N Events / N (%)** | **Adjusted Hazard Ratio (95% CI)** | **N Events / N (%)** | **Adjusted Hazard Ratio (95% CI)** | **N Events / N (%)** | **Adjusted Hazard Ratio (95% CI)** |
| Sex |  |  |  |  |  |  |  |  |  |  |
| Female | 73,648 / 1,006,088 (7.3%) | Reference | 13,705 / 73,648 (19%) | Reference | 3,481 / 73,648 (4.7%) | Reference | 3,337 / 73,648 (4.5%) | Reference | 4,878 / 73,648 (6.6%) | Reference |
| Male | 66,542 / 705,549 (9.4%) | 1.16 (1.14-1.17) | 17,606 / 66,542 (26%) | 1.39 (1.36-1.42) | 4,806 / 66,542 (7.2%) | 1.41 (1.35-1.48) | 5,024 / 66,542 (7.6%) | 1.63 (1.56-1.70) | 6,190 / 66,542 (9.3%) | 1.31 (1.26-1.36) |
| Age at COVID-19 Diagnosis | 140,190 / 1,711,637 (8.2%) | 1.03 (1.03-1.04) | 31,311 / 140,190 (22%) | 1.01 (1.01-1.02) | 8,287 / 140,190 (5.9%) | 1.03 (1.02-1.03) | 8,361 / 140,190 (6.0%) | 1.00 (1.00-1.00) | 11,068 / 140,190 (7.9%) | 1.04 (1.03-1.04) |
| Race/Ethnicity |  |  |  |  |  |  |  |  |  |  |
| White Non-Hispanic | 89,936 / 1,149,639 (7.8%) | Reference | 18,456 / 89,936 (21%) | Reference | 5,258 / 89,936 (5.8%) | Reference | 5,167 / 89,936 (5.7%) | Reference | 7,762 / 89,936 (8.6%) | Reference |
| Black or African American Non-Hispanic | 26,153 / 224,965 (12%) | 1.65 (1.62-1.67) | 7,810 / 26,153 (30%) | 1.53 (1.49-1.58) | 1,702 / 26,153 (6.5%) | 1.25 (1.18-1.32) | 1,622 / 26,153 (6.2%) | 1.07 (1.01-1.14) | 1,620 / 26,153 (6.2%) | 0.91 (0.86-0.96) |
| Hispanic or Latino Any Race | 13,257 / 140,988 (9.4%) | 1.68 (1.64-1.71) | 2,696 / 13,257 (20%) | 1.10 (1.05-1.14) | 617 / 13,257 (4.7%) | 1.01 (0.93-1.11) | 785 / 13,257 (5.9%) | 1.05 (0.97-1.14) | 843 / 13,257 (6.4%) | 1.03 (0.95-1.11) |
| Other | 4,644 / 88,652 (5.2%) | 0.92 (0.89-0.95) | 1,113 / 4,644 (24%) | 1.25 (1.17-1.33) | 304 / 4,644 (6.5%) | 1.27 (1.13-1.43) | 337 / 4,644 (7.3%) | 1.30 (1.16-1.46) | 386 / 4,644 (8.3%) | 1.12 (1.01-1.24) |
| Missing/Unknown | 6,200 / 107,393 (5.8%) | 0.96 (0.93-0.98) | 1,236 / 6,200 (20%) | 1.16 (1.09-1.23) | 406 / 6,200 (6.5%) | 1.34 (1.21-1.49) | 450 / 6,200 (7.3%) | 1.34 (1.21-1.47) | 457 / 6,200 (7.4%) | 1.13 (1.03-1.25) |
| Myocardial infarction (MI) |  |  |  |  |  |  |  |  |  |  |
| No History of MI | 127,400 / 1,665,253 (7.7%) | Reference | 26,595 / 127,400 (21%) | Reference | 6,180 / 127,400 (4.9%) | Reference | 7,412 / 127,400 (5.8%) | Reference | 9,582 / 127,400 (7.5%) | Reference |
| History of MI | 12,790 / 46,384 (28%) | 1.22 (1.19-1.25) | 4,716 / 12,790 (37%) | 1.03 (1.00-1.07) | 2,107 / 12,790 (16%) | 2.69 (2.54-2.86) | 949 / 12,790 (7.4%) | 1.11 (1.03-1.20) | 1,486 / 12,790 (12%) | 1.09 (1.03-1.16) |
| Congestive heart failure (CHF) |  |  |  |  |  |  |  |  |  |  |
| No History of CHF | 113,160 / 1,620,292 (7.0%) | Reference | 21,550 / 113,160 (19%) | Reference | 5,657 / 113,160 (5.0%) | Reference | 6,505 / 113,160 (5.7%) | Reference | 7,993 / 113,160 (7.1%) | Reference |
| History of CHF | 27,030 / 91,345 (30%) | 1.87 (1.83-1.91) | 9,761 / 27,030 (36%) | 1.06 (1.03-1.10) | 2,630 / 27,030 (9.7%) | 1.20 (1.13-1.27) | 1,856 / 27,030 (6.9%) | 1.13 (1.05-1.20) | 3,075 / 27,030 (11%) | 1.18 (1.12-1.24) |
| Peripheral vascular disease (PVD) |  |  |  |  |  |  |  |  |  |  |
| No History of PVD | 125,768 / 1,653,282 (7.6%) | Reference | 26,081 / 125,768 (21%) | Reference | 6,850 / 125,768 (5.4%) | Reference | 7,461 / 125,768 (5.9%) | Reference | 9,482 / 125,768 (7.5%) | Reference |
| History of PVD | 14,422 / 58,355 (25%) | 1.10 (1.07-1.12) | 5,230 / 14,422 (36%) | 0.98 (0.94-1.01) | 1,437 / 14,422 (10%) | 1.07 (1.00-1.14) | 900 / 14,422 (6.2%) | 0.92 (0.85-1.0) | 1,586 / 14,422 (11%) | 1.04 (0.98-1.10) |
| Cerebrovascular disease (CVD) |  |  |  |  |  |  |  |  |  |  |
| No History of CVD | 125,099 / 1,645,684 (7.6%) | Reference | 26,521 / 125,099 (21%) | Reference | 6,798 / 125,099 (5.4%) | Reference | 7,448 / 125,099 (6.0%) | Reference | 9,431 / 125,099 (7.5%) | Reference |
| History of CVD | 15,091 / 65,953 (23%) | 1.01 (0.98-1.03) | 4,790 / 15,091 (32%) | 0.95 (0.91-0.98) | 1,489 / 15,091 (9.9%) | 1.20 (1.12-1.28) | 913 / 15,091 (6.0%) | 0.99 (0.91-1.07) | 1,637 / 15,091 (11%) | 0.95 (0.89-1.00) |
| Dementia |  |  |  |  |  |  |  |  |  |  |
| No History of dementia | 131,755 / 1,683,113 (7.8%) | Reference | 28,864 / 131,755 (22%) | Reference | 7,741 / 131,755 (5.9%) | Reference | 8,027 / 131,755 (6.1%) | Reference | 9,852 / 131,755 (7.5%) | Reference |
| History of dementia | 8,435 / 28,524 (30%) | 1.63 (1.58-1.68) | 2,447 / 8,435 (29%) | 0.84 (0.80-0.88) | 546 / 8,435 (6.5%) | 0.64 (0.59-0.70) | 334 / 8,435 (4.0%) | 0.65 (0.58-0.73) | 1,216 / 8,435 (14%) | 1.24 (1.16-1.32) |
| Chronic pulmonary disease (CPD) |  |  |  |  |  |  |  |  |  |  |
| No History of CPD | 103,740 / 1,474,673 (7.0%) | Reference | 20,922 / 103,740 (20%) | Reference | 5,716 / 103,740 (5.5%) | Reference | 6,013 / 103,740 (5.8%) | Reference | 7,582 / 103,740 (7.3%) | Reference |
| History of CPD | 36,450 / 236,964 (15%) | 1.20 (1.18-1.22) | 10,389 / 36,450 (29%) | 0.92 (0.90-0.95) | 2,571 / 36,450 (7.1%) | 0.89 (0.84-0.94) | 2,348 / 36,450 (6.4%) | 1.09 (1.03-1.15) | 3,486 / 36,450 (9.6%) | 1.08 (1.03-1.13) |
| Rheumatologic disease (RD) |  |  |  |  |  |  |  |  |  |  |
| No History of RD | 126,589 / 1,619,983 (7.8%) | Reference | 26,577 / 126,589 (21%) | Reference | 7,279 / 126,589 (5.8%) | Reference | 7,458 / 126,589 (5.9%) | Reference | 9,700 / 126,589 (7.7%) | Reference |
| History of RD | 13,601 / 91,654 (15%) | 0.93 (0.91-0.95) | 4,734 / 13,601 (35%) | 1.11 (1.08-1.15) | 1,008 / 13,601 (7.4%) | 0.96 (0.90-1.03) | 903 / 13,601 (6.6%) | 1.09 (1.01-1.18) | 1,368 / 13,601 (10%) | 1.03 (0.97-1.09) |
| Peptic ulcer disease (PUD) |  |  |  |  |  |  |  |  |  |  |
| No History of PUD | 136,074 / 1,690,219 (8.1%) | Reference | 29,794 / 136,074 (22%) | Reference | 7,997 / 136,074 (5.9%) | Reference | 8,077 / 136,074 (5.9%) | Reference | 10,655 / 136,074 (7.8%) | Reference |
| History of PUD | 4,116 / 21,418 (19%) | 1.10 (1.05-1.14) | 1,517 / 4,116 (37%) | 1.13 (1.07-1.19) | 290 / 4,116 (7.0%) | 0.92 (0.82-1.04) | 284 / 4,116 (6.9%) | 1.01 (0.90-1.14) | 413 / 4,116 (10%) | 0.99 (0.90-1.10) |
| Liver Disease |  |  |  |  |  |  |  |  |  |  |
| No History of Liver Disease | 126,759 / 1,625,696 (7.8%) | Reference | 27,101 / 126,759 (21%) | Reference | 7,520 / 126,759 (5.9%) | Reference | 7,309 / 126,759 (5.8%) | Reference | 9,824 / 126,759 (7.8%) | Reference |
| Mild Liver Disease | 9,324 / 72,561 (13%) | 0.99 (0.96-1.01) | 2,591 / 9,324 (28%) | 1.00 (0.96-1.04) | 538 / 9,324 (5.8%) | 0.92 (0.84-1.00) | 629 / 9,324 (6.7%) | 1.05 (0.96-1.14) | 692 / 9,324 (7.4%) | 1.02 (0.94-1.11) |
| Moderate to Severe Liver Disease | 4,107 / 13,380 (31%) | 1.77 (1.70-1.85) | 1,619 / 4,107 (39%) | 1.32 (1.26-1.39) | 229 / 4,107 (5.6%) | 0.86 (0.75-0.99) | 423 / 4,107 (10%) | 1.49 (1.35-1.66) | 552 / 4,107 (13%) | 1.82 (1.66-1.99) |
| Diabetes Mellitus |  |  |  |  |  |  |  |  |  |  |
| No History of Diabetes | 103,799 / 1,500,684 (6.9%) | Reference | 19,048 / 103,799 (18%) | Reference | 5,448 / 103,799 (5.2%) | Reference | 6,043 / 103,799 (5.8%) | Reference | 7,747 / 103,799 (7.5%) | Reference |
| Diabetes without Chronic Complications | 12,361 / 99,642 (12%) | 1.35 (1.32-1.38) | 2,751 / 12,361 (22%) | 1.06 (1.01-1.10) | 719 / 12,361 (5.8%) | 1.00 (0.92-1.08) | 648 / 12,361 (5.2%) | 0.94 (0.87-1.03) | 935 / 12,361 (7.6%) | 0.98 (0.91-1.05) |
| Diabetes with Chronic Complications | 24,030 / 111,311 (22%) | 1.35 (1.33-1.38) | 9,512 / 24,030 (40%) | 1.26 (1.23-1.30) | 2,120 / 24,030 (8.8%) | 1.16 (1.09-1.23) | 1,670 / 24,030 (6.9%) | 1.16 (1.08-1.24) | 2,386 / 24,030 (9.9%) | 1.04 (0.98-1.10) |
| Hemiplegia or paraplegia |  |  |  |  |  |  |  |  |  |  |
| No History of Hemiplegia or paraplegia | 135,471 / 1,697,780 (8.0%) | Reference | 29,879 / 135,471 (22%) | Reference | 7,914 / 135,471 (5.8%) | Reference | 8,041 / 135,471 (5.9%) | Reference | 10,595 / 135,471 (7.8%) | Reference |
| History of Hemiplegia or paraplegia | 4,719 / 13,857 (34%) | 2.00 (1.92-2.09) | 1,432 / 4,719 (30%) | 0.92 (0.87-0.98) | 373 / 4,719 (7.9%) | 0.87 (0.77-0.97) | 320 / 4,719 (6.8%) | 1.07 (0.95-1.21) | 473 / 4,719 (10%) | 1.12 (1.01-1.23) |
| Renal Disease |  |  |  |  |  |  |  |  |  |  |
| No History of Renal Disease | 101,324 / 1,554,639 (6.5%) | Reference | 15,018 / 101,324 (15%) | Reference | 5,222 / 101,324 (5.2%) | Reference | 5,799 / 101,324 (5.7%) | Reference | 6,919 / 101,324 (6.8%) | Reference |
| History of Disease Renal | 38,866 / 156,998 (25%) | 1.92 (1.89-1.95) | 16,293 / 38,866 (42%) | 2.48 (2.41-2.55) | 3,065 / 38,866 (7.9%) | 0.97 (0.91-1.03) | 2,562 / 38,866 (6.6%) | 1.07 (1.01-1.14) | 4,149 / 38,866 (11%) | 1.18 (1.12-1.24) |
| Cancer |  |  |  |  |  |  |  |  |  |  |
| No History of Malignant Cancer | 118,216 / 1,570,797 (7.5%) | Reference | 25,261 / 118,216 (21%) | Reference | 7,000 / 118,216 (5.9%) | Reference | 7,098 / 118,216 (6.0%) | Reference | 8,293 / 118,216 (7.0%) | Reference |
| Any Malignancy Except Neoplasm of skin | 17,397 / 122,272 (14%) | 0.99 (0.97-1.01) | 4,817 / 17,397 (28%) | 0.93 (0.90-0.96) | 1,070 / 17,397 (6.2%) | 0.79 (0.74-0.85) | 1,010 / 17,397 (5.8%) | 0.95 (0.88-1.01) | 1,934 / 17,397 (11%) | 1.23 (1.16-1.29) |
| Metastatic Solid Tumor | 4,577 / 18,568 (25%) | 2.07 (2.00-2.15) | 1,233 / 4,577 (27%) | 0.98 (0.92-1.04) | 217 / 4,577 (4.7%) | 0.70 (0.61-0.80) | 253 / 4,577 (5.5%) | 0.89 (0.79-1.01) | 841 / 4,577 (18%) | 2.53 (2.35-2.72) |
| HIV |  |  |  |  |  |  |  |  |  |  |
| No History of HIV | 139,118 / 1,704,616 (8.2%) | Reference | 31,013 / 139,118 (22%) | Reference | 8,232 / 139,118 (5.9%) | Reference | 8,294 / 139,118 (6.0%) | Reference | 11,006 / 139,118 (7.9%) | Reference |
| History of HIV | 1,072 / 7,021 (15%) | 1.10 (1.02-1.18) | 298 / 1,072 (28%) | 0.95 (0.84-1.06) | 55 / 1,072 (5.1%) | 0.89 (0.68-1.17) | 67 / 1,072 (6.2%) | 0.83 (0.65-1.06) | 62 / 1,072 (5.8%) | 0.99 (0.77-1.28) |
| Obesity |  |  |  |  |  |  |  |  |  |  |
| No History of Obesity | 92,213 / 1,212,709 (7.6%) | Reference | 18,408 / 92,213 (20%) | Reference | 5,345 / 92,213 (5.8%) | Reference | 5,402 / 92,213 (5.9%) | Reference | 7,533 / 92,213 (8.2%) | Reference |
| History of Obesity | 47,977 / 498,928 (9.6%) | 0.83 (0.82-0.84) | 12,903 / 47,977 (27%) | 1.05 (1.02-1.07) | 2,942 / 47,977 (6.1%) | 0.95 (0.90-1.00) | 2,959 / 47,977 (6.2%) | 1.04 (0.99-1.09) | 3,535 / 47,977 (7.4%) | 0.92 (0.88-0.96) |
| Hypertension |  |  |  |  |  |  |  |  |  |  |
| No History of Hypertension | 72,017 / 1,220,314 (5.9%) | Reference | 10,775 / 72,017 (15%) | Reference | 3,249 / 72,017 (4.5%) | Reference | 4,355 / 72,017 (6.0%) | Reference | 4,784 / 72,017 (6.6%) | Reference |
| History of Hypertension | 68,173 / 491,323 (14%) | 0.82 (0.81-0.84) | 20,536 / 68,173 (30%) | 1.09 (1.06-1.13) | 5,038 / 68,173 (7.4%) | 1.03 (0.97-1.09) | 4,006 / 68,173 (5.9%) | 0.84 (0.79-0.89) | 6,284 / 68,173 (9.2%) | 0.81 (0.77-0.85) |
| Former or Current Tobacco User |  |  |  |  |  |  |  |  |  |  |
| No History of Tobacco Use | 123,727 / 1,625,089 (7.6%) | Reference | 26,962 / 123,727 (22%) | Reference | 7,058 / 123,727 (5.7%) | Reference | 7,104 / 123,727 (5.7%) | Reference | 9,903 / 123,727 (8.0%) | Reference |
| History of Tobacco Use | 16,463 / 86,548 (19%) | 1.72 (1.69-1.76) | 4,349 / 16,463 (26%) | 1.12 (1.08-1.16) | 1,229 / 16,463 (7.5%) | 1.28 (1.20-1.37) | 1,257 / 16,463 (7.6%) | 1.06 (1.00-1.14) | 1,165 / 16,463 (7.1%) | 0.99 (0.92-1.05) |
| History of Substance Abuse Disorder |  |  |  |  |  |  |  |  |  |  |
| No History of Substance Abuse | 128,068 / 1,662,229 (7.7%) | Reference | 28,293 / 128,068 (22%) | Reference | 7,647 / 128,068 (6.0%) | Reference | 7,296 / 128,068 (5.7%) | Reference | 10,319 / 128,068 (8.1%) | Reference |
| History of Substance Abuse | 12,122 / 49,408 (25%) | 2.90 (2.82-2.97) | 3,018 / 12,122 (25%) | 1.02 (0.98-1.06) | 640 / 12,122 (5.3%) | 0.92 (0.84-1.00) | 1,065 / 12,122 (8.8%) | 1.26 (1.17-1.35) | 749 / 12,122 (6.2%) | 1.02 (0.94-1.10) |
| Vaccination Status Prior to SARS-CoV-2 Infection |  |  |  |  |  |  |  |  |  |  |
| No Documented COVID-19 Vaccination | 102,382 / 1,120,211 (9.1%) | Reference | 23,233 / 102,382 (23%) | Reference | 6,379 / 102,382 (6.2%) | Reference | 6,665 / 102,382 (6.5%) | Reference | 8,285 / 102,382 (8.1%) | Reference |
| Primary Vaccination Series | 18,854 / 293,409 (6.4%) | 0.68 (0.67-0.69) | 4,062 / 18,854 (22%) | 0.82 (0.79-0.84) | 941 / 18,854 (5.0%) | 0.75 (0.70-0.80) | 892 / 18,854 (4.7%) | 0.72 (0.67-0.77) | 1,420 / 18,854 (7.5%) | 0.79 (0.75-0.84) |
| Primary+ Vaccination Series | 18,954 / 298,017 (6.4%) | 0.50 (0.49-0.51) | 4,016 / 18,954 (21%) | 0.73 (0.70-0.75) | 967 / 18,954 (5.1%) | 0.68 (0.63-0.73) | 804 / 18,954 (4.2%) | 0.64 (0.59-0.69) | 1,363 / 18,954 (7.2%) | 0.61 (0.57-0.64) |
| Census Region |  |  |  |  |  |  |  |  |  |  |
| Midwest | 48,428 / 733,572 (6.6%) | Reference | 9,348 / 48,428 (19%) | Reference | 2,311 / 48,428 (4.8%) | Reference | 2,275 / 48,428 (4.7%) | Reference | 3,925 / 48,428 (8.1%) | Reference |
| Northeast | 25,052 / 211,496 (12%) | 1.30 (1.27-1.32) | 5,454 / 25,052 (22%) | 1.13 (1.10-1.18) | 1,563 / 25,052 (6.2%) | 1.17 (1.10-1.26) | 1,144 / 25,052 (4.6%) | 0.88 (0.82-0.95) | 1,827 / 25,052 (7.3%) | 0.79 (0.75-0.84) |
| South | 47,872 / 485,665 (9.9%) | 1.25 (1.23-1.27) | 11,817 / 47,872 (25%) | 1.30 (1.26-1.34) | 3,341 / 47,872 (7.0%) | 1.43 (1.36-1.51) | 3,450 / 47,872 (7.2%) | 1.47 (1.39-1.55) | 3,782 / 47,872 (7.9%) | 1.03 (0.98-1.08) |
| West | 18,838 / 280,904 (6.7%) | 0.81 (0.80-0.83) | 4,692 / 18,838 (25%) | 1.38 (1.33-1.43) | 1,072 / 18,838 (5.7%) | 1.17 (1.08-1.26) | 1,492 / 18,838 (7.9%) | 1.58 (1.48-1.70) | 1,534 / 18,838 (8.1%) | 0.94 (0.88-1.00) |
| Rural-Dwelling Status |  |  |  |  |  |  |  |  |  |  |
| Urban | 117,092 / 1,456,361 (8.0%) | Reference | 26,575 / 117,092 (23%) | Reference | 6,870 / 117,092 (5.9%) | Reference | 6,809 / 117,092 (5.8%) | Reference | 8,845 / 117,092 (7.6%) | Reference |
| Urban-Adjacent Rural | 18,298 / 207,261 (8.8%) | 1.15 (1.13-1.17) | 3,728 / 18,298 (20%) | 1.01 (0.98-1.05) | 1,080 / 18,298 (5.9%) | 1.05 (0.99-1.13) | 1,180 / 18,298 (6.4%) | 1.17 (1.10-1.25) | 1,745 / 18,298 (9.5%) | 1.25 (1.18-1.31) |
| Nonurban-Adjacent Rural | 4,800 / 48,015 (10%) | 1.16 (1.13-1.20) | 1,008 / 4,800 (21%) | 1.03 (0.96-1.10) | 337 / 4,800 (7.0%) | 1.19 (1.06-1.33) | 372 / 4,800 (7.8%) | 1.38 (1.24-1.53) | 478 / 4,800 (10%) | 1.28 (1.17-1.40) |

**eTable S-3. Univariable Regression for Adverse Acute COVID-19 Events by Rurality**

| **Characteristic** | **All Time Periods, January 2021 – December 2022** | | | | |
| --- | --- | --- | --- | --- | --- |
|  | **Hospitalization** | **AKI/Dialysis** | **MACE** | **ECMO/IMV** | **Death** |
|  | **Odds Ratio (95% CI)** | **Hazard Ratio (95% CI)** | **Hazard Ratio (95% CI)** | **Hazard Ratio (95% CI)** | **Hazard Ratio (95% CI)** |
| Rurality |  |  |  |  |  |
| Urban | Reference | Reference | Reference | Reference | Reference |
| Urban-Adjacent Rural | 1.00 (0.99-1.01) | 0.89 (0.86-0.91) | 0.97 (0.93-1.01) | 1.23 (1.19-1.28) | 1.30 (1.26-1.35) |
| Nonurban-Adjacent Rural | 1.09 (1.06-1.11) | 0.95 (0.91-0.99) | 1.04 (0.96-1.13) | 1.47 (1.38-1.56) | 1.42 (1.34-1.50) |
| **Characteristic** | **Pre-Delta Dominance, January 1, 2021 - June 14, 2021** | | | | |
|  | **Hospitalization** | **AKI/Dialysis** | **MACE** | **ECMO/IMV** | **Death** |
|  | **Odds Ratio (95% CI)** | **Hazard Ratio (95% CI)** | **Hazard Ratio (95% CI)** | **Hazard Ratio (95% CI)** | **Hazard Ratio (95% CI)** |
| Rurality |  |  |  |  |  |
| Urban | Reference | Reference | Reference | Reference | Reference |
| Urban-Adjacent Rural | 0.78 (0.76-0.80) | 0.99 (0.94-1.05) | 0.99 (0.90-1.10) | 1.11 (1.04-1.20) | 1.27 (1.18-1.36) |
| Nonurban-Adjacent Rural | 0.79 (0.76-0.84) | 1.03 (0.93-1.14) | 0.96 (0.79-1.17) | 1.34 (1.18-1.52) | 1.32 (1.16-1.51) |
| **Characteristic** | **Delta Dominance, June 15, 2021 – December 21, 2021** | | | | |
|  | **Hospitalization** | **AKI/Dialysis** | **MACE** | **ECMO/IMV** | **Death** |
|  | **Odds Ratio (95% CI)** | **Hazard Ratio (95% CI)** | **Hazard Ratio (95% CI)** | **Hazard Ratio (95% CI)** | **Hazard Ratio (95% CI)** |
| Rurality |  |  |  |  |  |
| Urban | Reference | Reference | Reference | Reference | Reference |
| Urban-Adjacent Rural | 0.95 (0.93-0.97) | 0.84 (0.81-0.87) | 0.96 (0.88-1.05) | 1.19 (1.12-1.25) | 1.30 (1.24-1.37) |
| Nonurban-Adjacent Rural | 0.99 (0.96-1.03) | 0.95 (0.89-1.02) | 0.89 (0.76-1.05) | 1.43 (1.30-1.57) | 1.49 (1.37-1.62) |
| **Characteristic** | **Omicron Dominance, December 22, 2021 – December 31, 2022** | | | | |
|  | **Hospitalization** | **AKI/Dialysis** | **MACE** | **ECMO/IMV** | **Death** |
|  | **Odds Ratio (95% CI)** | **Hazard Ratio (95% CI)** | **Hazard Ratio (95% CI)** | **Hazard Ratio (95% CI)** | **Hazard Ratio (95% CI)** |
| Rurality |  |  |  |  |  |
| Urban | Reference | Reference | Reference | Reference | Reference |
| Urban-Adjacent Rural | 1.11 (1.09-1.13) | 0.88 (0.85-0.92) | 1.01 (0.94-1.07) | 1.12 (1.05-1.19) | 1.28 (1.22-1.35) |
| Nonurban-Adjacent Rural | 1.27 (1.23-1.31) | 0.92 (0.86-0.98) | 1.21 (1.08-1.35) | 1.35 (1.22-1.50) | 1.34 (1.22-1.47) |

**eTable S-4. Multivariable Regression for Adverse Acute COVID-19 Events Stratified by Rurality**

| **Characteristic** | **All Time Periods, January 2021 – December 2022** | | | | | | | | | |
| --- | --- | --- | --- | --- | --- | --- | --- | --- | --- | --- |
|  | **Hospitalization** | | **AKI/Dialysis** | | **MACE** | | **ECMO/IMV** | | **Death** | |
|  | **Rural** | **Urban** | **Rural** | **Urban** | **Rural** | **Urban** | **Rural** | **Urban** | **Rural** | **Urban** |
|  | **Adjusted Odds Ratio (95% CI)** | **Adjusted Odds Ratio (95% CI)** | **Adjusted Hazard Ratio (95% CI)** | **Adjusted Hazard Ratio (95% CI)** | **Adjusted Hazard Ratio (95% CI)** | **Adjusted Hazard Ratio (95% CI)** | **Adjusted Hazard Ratio (95% CI)** | **Adjusted Hazard Ratio (95% CI)** | **Adjusted Hazard Ratio (95% CI)** | **Adjusted Hazard Ratio (95% CI)** |
| Sex |  |  |  |  |  |  |  |  |  |  |
| Female | Reference | Reference | Reference | Reference | Reference | Reference | Reference | Reference | Reference | Reference |
| Male | 1.24 (1.21-1.26) | 1.21 (1.19-1.22) | 1.40 (1.35-1.46) | 1.46 (1.43-1.48) | 1.45 (1.34-1.57) | 1.40 (1.35-1.45) | 1.44 (1.36-1.53) | 1.52 (1.48-1.57) | 1.26 (1.20-1.33) | 1.38 (1.34-1.41) |
| Age at COVID-19 Diagnosis | 1.04 (1.04-1.04) | 1.04 (1.04-1.04) | 1.01 (1.01-1.02) | 1.02 (1.02-1.02) | 1.02 (1.02-1.03) | 1.03 (1.03-1.03) | 1.00 (1.00-1.00) | 1.00 (1.00-1.00) | 1.04 (1.03-1.04) | 1.04 (1.04-1.04) |
| Race/Ethnicity |  |  |  |  |  |  |  |  |  |  |
| White Non-Hispanic | Reference | Reference | Reference | Reference | Reference | Reference | Reference | Reference | Reference | Reference |
| Black or African American Non-Hispanic | 1.26 (1.21-1.32) | 1.82 (1.80-1.85) | 1.71 (1.60-1.82) | 1.63 (1.59-1.66) | 1.41 (1.24-1.61) | 1.23 (1.17-1.28) | 1.17 (1.05-1.30) | 1.03 (0.99-1.07) | 1.06 (0.95-1.18) | 0.94 (0.91-0.98) |
| Hispanic or Latino Any Race | 1.53 (1.46-1.61) | 1.87 (1.84-1.90) | 1.16 (1.05-1.29) | 1.08 (1.05-1.12) | 0.97 (0.77-1.22) | 0.96 (0.90-1.02) | 1.24 (1.09-1.40) | 1.13 (1.08-1.18) | 1.05 (0.91-1.22) | 1.13 (1.08-1.18) |
| Other | 0.42 (0.39-0.46) | 1.12 (1.09-1.14) | 1.16 (0.96-1.39) | 1.25 (1.19-1.30) | 1.35 (0.96-1.90) | 1.28 (1.17-1.39) | 1.23 (0.95-1.58) | 1.33 (1.24-1.43) | 1.12 (0.87-1.44) | 1.21 (1.13-1.29) |
| Missing/Unknown | 0.87 (0.82-0.94) | 0.97 (0.95-0.99) | 1.52 (1.34-1.72) | 1.18 (1.13-1.23) | 1.84 (1.48-2.28) | 1.26 (1.17-1.36) | 1.71 (1.48-1.97) | 1.33 (1.25-1.42) | 1.85 (1.60-2.14) | 1.24 (1.16-1.32) |
| Myocardial infarction (MI) | 1.12 (1.06-1.17) | 1.19 (1.17-1.22) | 0.95 (0.89-1.02) | 1.02 (1.00-1.05) | 2.65 (2.37-2.96) | 2.72 (2.59-2.86) | 1.09 (0.96-1.24) | 1.03 (0.97-1.09) | 1.07 (0.97-1.18) | 1.05 (1.00-1.10) |
| Congestive heart failure (CHF) | 1.54 (1.48-1.60) | 1.73 (1.70-1.76) | 1.06 (1.00-1.13) | 1.06 (1.04-1.09) | 1.16 (1.04-1.30) | 1.23 (1.17-1.29) | 1.00 (0.90-1.11) | 1.06 (1.01-1.12) | 1.13 (1.04-1.22) | 1.15 (1.10-1.19) |
| Peripheral vascular disease (PVD) | 1.10 (1.05-1.16) | 1.09 (1.07-1.11) | 0.99 (0.93-1.06) | 0.97 (0.95-1.00) | 1.09 (0.96-1.24) | 1.04 (0.99-1.10) | 0.95 (0.83-1.08) | 0.96 (0.91-1.02) | 0.95 (0.86-1.05) | 1.05 (1.00-1.10) |
| Cerebrovascular disease (CVD) | 0.96 (0.91-1.01) | 0.98 (0.96-1.00) | 0.91 (0.84-0.98) | 0.97 (0.94-0.99) | 1.14 (1.00-1.30) | 1.16 (1.10-1.23) | 0.95 (0.83-1.09) | 0.98 (0.92-1.04) | 0.98 (0.88-1.08) | 0.94 (0.89-0.98) |
| Dementia | 1.13 (1.06-1.20) | 1.58 (1.54-1.62) | 0.91 (0.83-1.00) | 0.83 (0.80-0.85) | 0.53 (0.43-0.65) | 0.71 (0.66-0.76) | 0.46 (0.36-0.59) | 0.61 (0.55-0.66) | 1.20 (1.07-1.34) | 1.18 (1.13-1.24) |
| Chronic pulmonary disease (CPD) | 1.27 (1.23-1.31) | 1.17 (1.16-1.19) | 0.91 (0.87-0.96) | 0.92 (0.90-0.94) | 0.85 (0.76-0.94) | 0.90 (0.86-0.94) | 1.07 (0.98-1.17) | 1.06 (1.02-1.11) | 1.08 (1.01-1.16) | 1.07 (1.03-1.11) |
| Rheumatologic disease (RD) | 1.00 (0.96-1.04) | 0.95 (0.93-0.96) | 1.14 (1.07-1.22) | 1.11 (1.08-1.13) | 0.93 (0.81-1.06) | 1.01 (0.96-1.07) | 1.02 (0.90-1.14) | 1.10 (1.04-1.16) | 1.07 (0.97-1.17) | 1.01 (0.96-1.05) |
| Peptic ulcer disease (PUD) | 1.08 (1.00-1.17) | 1.08 (1.04-1.12) | 0.95 (0.85-1.07) | 1.09 (1.04-1.14) | 0.70 (0.53-0.93) | 0.97 (0.88-1.07) | 0.93 (0.74-1.17) | 1.07 (0.97-1.17) | 0.86 (0.72-1.03) | 0.99 (0.92-1.08) |
| Mild Liver Disease | 1.03 (0.98-1.08) | 0.99 (0.97-1.01) | 0.98 (0.90-1.07) | 1.03 (1.00-1.06) | 0.75 (0.61-0.91) | 0.95 (0.88-1.02) | 0.89 (0.77-1.03) | 1.11 (1.04-1.18) | 1.00 (0.88-1.14) | 1.03 (0.97-1.09) |
| Moderate to Severe Liver Disease | 1.70 (1.57-1.85) | 1.68 (1.62-1.75) | 1.29 (1.16-1.43) | 1.37 (1.31-1.43) | 0.77 (0.59-1.02) | 0.89 (0.80-1.00) | 1.37 (1.15-1.64) | 1.41 (1.30-1.54) | 1.67 (1.44-1.95) | 1.72 (1.60-1.85) |
| Diabetes without Chronic Complications | 1.43 (1.38-1.48) | 1.36 (1.34-1.39) | 1.01 (0.94-1.08) | 1.07 (1.04-1.11) | 0.98 (0.85-1.13) | 1.04 (0.98-1.11) | 0.97 (0.87-1.09) | 1.06 (1.00-1.12) | 1.01 (0.92-1.11) | 1.03 (0.99-1.09) |
| Diabetes with Chronic Complications | 1.51 (1.45-1.56) | 1.35 (1.32-1.37) | 1.24 (1.17-1.32) | 1.26 (1.23-1.29) | 1.23 (1.09-1.38) | 1.12 (1.07-1.18) | 1.11 (1.00-1.23) | 1.22 (1.16-1.28) | 1.10 (1.01-1.20) | 1.09 (1.04-1.13) |
| Hemiplegia or paraplegia | 1.63 (1.50-1.79) | 1.97 (1.89-2.04) | 0.96 (0.84-1.08) | 0.94 (0.90-0.99) | 0.93 (0.74-1.18) | 0.90 (0.82-0.99) | 0.94 (0.74-1.19) | 1.01 (0.92-1.11) | 1.09 (0.91-1.30) | 1.10 (1.02-1.19) |
| Renal Disease | 1.63 (1.58-1.69) | 1.83 (1.81-1.86) | 2.27 (2.15-2.39) | 2.39 (2.33-2.44) | 1.06 (0.95-1.18) | 1.03 (0.99-1.08) | 1.05 (0.96-1.16) | 1.05 (1.00-1.09) | 1.20 (1.11-1.29) | 1.21 (1.17-1.26) |
| Any Malignancy Except Neoplasm of skin | 1.07 (1.03-1.11) | 0.97 (0.95-0.98) | 0.97 (0.91-1.03) | 0.92 (0.89-0.94) | 0.82 (0.73-0.94) | 0.80 (0.76-0.84) | 0.88 (0.79-0.98) | 0.87 (0.83-0.92) | 1.15 (1.06-1.25) | 1.10 (1.06-1.15) |
| Metastatic Solid Tumor | 2.03 (1.88-2.19) | 1.84 (1.78-1.90) | 0.98 (0.87-1.10) | 0.92 (0.88-0.97) | 0.76 (0.59-0.98) | 0.75 (0.67-0.84) | 0.78 (0.62-0.97) | 0.89 (0.81-0.99) | 1.92 (1.69-2.18) | 2.17 (2.04-2.30) |
| HIV | 0.84 (0.68-1.04) | 0.99 (0.94-1.05) | 0.91 (0.65-1.28) | 0.96 (0.88-1.04) | 0.70 (0.31-1.57) | 0.95 (0.78-1.16) | 0.46 (0.21-1.04) | 0.91 (0.77-1.08) | 1.22 (0.72-2.07) | 0.99 (0.83-1.19) |
| Obesity | 0.87 (0.84-0.89) | 0.96 (0.95-0.97) | 1.07 (1.02-1.12) | 1.05 (1.03-1.07) | 0.99 (0.90-1.09) | 0.93 (0.89-0.97) | 1.08 (1.01-1.16) | 1.06 (1.03-1.10) | 0.89 (0.83-0.95) | 1.01 (0.98-1.04) |
| Hypertension | 0.86 (0.83-0.88) | 0.84 (0.83-0.85) | 1.14 (1.08-1.20) | 1.13 (1.10-1.16) | 1.07 (0.96-1.19) | 1.06 (1.01-1.11) | 0.86 (0.79-0.94) | 0.89 (0.85-0.92) | 0.87 (0.81-0.93) | 0.84 (0.81-0.87) |
| Former or Current Tobacco User | 1.69 (1.62-1.75) | 1.58 (1.55-1.61) | 1.07 (1.01-1.15) | 1.10 (1.07-1.13) | 1.24 (1.10-1.40) | 1.23 (1.16-1.30) | 0.95 (0.85-1.05) | 0.99 (0.94-1.04) | 0.98 (0.89-1.08) | 0.99 (0.94-1.04) |
| History of Substance Abuse Disorder | 2.38 (2.26-2.51) | 2.77 (2.71-2.83) | 1.06 (0.97-1.15) | 1.01 (0.97-1.04) | 0.97 (0.82-1.17) | 0.97 (0.90-1.04) | 1.19 (1.05-1.35) | 1.15 (1.08-1.21) | 0.96 (0.84-1.10) | 1.00 (0.94-1.06) |
| Variant Period |  |  |  |  |  |  |  |  |  |  |
| Pre-Delta (December 10, 2020 - June 14, 2021) | Reference | Reference | Reference | Reference | Reference | Reference | Reference | Reference | Reference | Reference |
| Delta (June 15, 2021 - December 21, 2021) | 1.19 (1.16-1.22) | 0.96 (0.94-0.97) | 1.02 (0.96-1.08) | 1.10 (1.07-1.13) | 0.87 (0.78-0.97) | 0.90 (0.86-0.95) | 1.14 (1.06-1.23) | 1.25 (1.21-1.30) | 1.35 (1.26-1.45) | 1.27 (1.22-1.31) |
| Omicron (>= December 22 ,2021) | 0.81 (0.79-0.83) | 0.55 (0.55-0.56) | 0.91 (0.86-0.96) | 0.96 (0.94-0.98) | 1.08 (0.98-1.20) | 0.99 (0.95-1.04) | 0.66 (0.61-0.72) | 0.77 (0.74-0.80) | 0.81 (0.75-0.87) | 0.72 (0.70-0.75) |
| Vaccination Status Prior to SARS-CoV-2 Infection |  |  |  |  |  |  |  |  |  |  |
| No Documented COVID-19 Vaccination | Reference | Reference | Reference | Reference | Reference | Reference | Reference | Reference | Reference | Reference |
| Primary Vaccination Series | 0.50 (0.49-0.52) | 0.56 (0.55-0.57) | 0.77 (0.72-0.83) | 0.81 (0.79-0.83) | 0.83 (0.73-0.95) | 0.75 (0.71-0.80) | 0.59 (0.51-0.67) | 0.67 (0.63-0.71) | 0.64 (0.59-0.71) | 0.72 (0.68-0.75) |
| Primary+ Vaccination Series | 0.43 (0.41-0.45) | 0.48 (0.47-0.49) | 0.74 (0.68-0.81) | 0.75 (0.73-0.78) | 0.73 (0.62-0.85) | 0.70 (0.65-0.75) | 0.67 (0.56-0.79) | 0.65 (0.60-0.71) | 0.56 (0.49-0.63) | 0.63 (0.59-0.67) |
| Census Region |  |  |  |  |  |  |  |  |  |  |
| Midwest | Reference | Reference | Reference | Reference | Reference | Reference | Reference | Reference | Reference | Reference |
| Northeast | 0.67 (0.64-0.70) | 1.32 (1.30-1.33) | 1.62 (1.50-1.76) | 1.32 (1.29-1.35) | 1.83 (1.59-2.12) | 1.59 (1.51-1.67) | 2.04 (1.80-2.30) | 1.13 (1.07-1.18) | 0.98 (0.88-1.10) | 0.90 (0.86-0.94) |
| South | 1.79 (1.75-1.83) | 1.14 (1.13-1.16) | 2.02 (1.93-2.11) | 1.43 (1.40-1.46) | 1.93 (1.77-2.11) | 1.63 (1.56-1.71) | 2.39 (2.23-2.56) | 2.08 (2.00-2.16) | 1.36 (1.28-1.44) | 1.13 (1.09-1.17) |
| West | 1.58 (1.51-1.66) | 0.87 (0.86-0.89) | 2.48 (2.27-2.70) | 1.53 (1.49-1.57) | 1.54 (1.27-1.87) | 1.38 (1.30-1.46) | 3.97 (3.57-4.41) | 2.35 (2.24-2.46) | 1.82 (1.63-2.02) | 1.08 (1.03-1.12) |
| **Characteristic** | **Pre-Delta Dominance, January 1, 2021 - June 14, 2021** | | | | | | | | | |
|  | **Hospitalization** | | **AKI/Dialysis** | | **MACE** | | **ECMO/IMV** | | **Death** | |
|  | **Rural** | **Urban** | **Rural** | **Urban** | **Rural** | **Urban** | **Rural** | **Urban** | **Rural** | **Urban** |
|  | **Adjusted Odds Ratio (95% CI)** | **Adjusted Odds Ratio (95% CI)** | **Adjusted Hazard Ratio (95% CI)** | **Adjusted Hazard Ratio (95% CI)** | **Adjusted Hazard Ratio (95% CI)** | **Adjusted Hazard Ratio (95% CI)** | **Adjusted Hazard Ratio (95% CI)** | **Adjusted Hazard Ratio (95% CI)** | **Adjusted Hazard Ratio (95% CI)** | **Adjusted Hazard Ratio (95% CI)** |
| Sex |  |  |  |  |  |  |  |  |  |  |
| Female | Reference | Reference | Reference | Reference | Reference | Reference | Reference | Reference | Reference | Reference |
| Male | 1.25 (1.19-1.31) | 1.28 (1.25-1.30) | 1.39 (1.27-1.52) | 1.49 (1.44-1.54) | 1.53 (1.28-1.84) | 1.37 (1.28-1.47) | 1.45 (1.28-1.65) | 1.46 (1.38-1.55) | 1.19 (1.06-1.35) | 1.44 (1.37-1.51) |
| Age at COVID-19 Diagnosis | 1.04 (1.04-1.04) | 1.04 (1.04-1.04) | 1.02 (1.01-1.02) | 1.02 (1.02-1.02) | 1.02 (1.02-1.03) | 1.03 (1.03-1.03) | 1.00 (1.00-1.01) | 1.01 (1.00-1.01) | 1.04 (1.03-1.04) | 1.05 (1.05-1.05) |
| Race/Ethnicity |  |  |  |  |  |  |  |  |  |  |
| White Non-Hispanic | Reference | Reference | Reference | Reference | Reference | Reference | Reference | Reference | Reference | Reference |
| Black or African American Non-Hispanic | 1.28 (1.17-1.41) | 2.14 (2.09-2.19) | 1.79 (1.58-2.04) | 1.70 (1.63-1.77) | 1.16 (0.88-1.54) | 1.15 (1.05-1.25) | 1.20 (0.99-1.46) | 1.06 (0.98-1.14) | 0.98 (0.79-1.21) | 1.06 (0.99-1.13) |
| Hispanic or Latino Any Race | 1.64 (1.47-1.82) | 2.14 (2.08-2.20) | 1.19 (0.96-1.48) | 1.07 (1.01-1.13) | 0.94 (0.59-1.51) | 0.81 (0.73-0.90) | 1.37 (1.08-1.75) | 1.17 (1.08-1.27) | 1.14 (0.83-1.55) | 1.26 (1.17-1.36) |
| Other | 0.37 (0.30-0.45) | 1.61 (1.54-1.68) | 0.88 (0.54-1.44) | 1.20 (1.11-1.30) | 1.37 (0.61-3.08) | 1.16 (1.01-1.33) | 1.11 (0.61-2.02) | 1.35 (1.20-1.52) | 0.72 (0.34-1.51) | 1.30 (1.17-1.44) |
| Missing/Unknown | 0.76 (0.65-0.89) | 1.07 (1.03-1.11) | 1.84 (1.41-2.40) | 1.21 (1.13-1.31) | 1.56 (0.94-2.59) | 1.04 (0.91-1.19) | 1.17 (0.82-1.68) | 1.41 (1.26-1.58) | 1.78 (1.28-2.47) | 1.47 (1.33-1.63) |
| Myocardial infarction (MI) | 1.12 (0.99-1.27) | 1.16 (1.10-1.22) | 0.88 (0.74-1.03) | 1.05 (0.99-1.12) | 2.21 (1.66-2.95) | 2.87 (2.60-3.16) | 0.95 (0.70-1.28) | 1.02 (0.90-1.15) | 0.99 (0.79-1.25) | 0.97 (0.88-1.07) |
| Congestive heart failure (CHF) | 1.30 (1.18-1.44) | 1.55 (1.50-1.61) | 1.08 (0.94-1.23) | 1.05 (1.00-1.10) | 0.84 (0.63-1.13) | 1.29 (1.17-1.42) | 0.82 (0.64-1.06) | 1.02 (0.93-1.13) | 1.29 (1.06-1.56) | 1.15 (1.07-1.24) |
| Peripheral vascular disease (PVD) | 1.11 (0.98-1.25) | 1.02 (0.97-1.07) | 1.13 (0.97-1.32) | 0.96 (0.90-1.01) | 1.11 (0.81-1.53) | 1.03 (0.92-1.15) | 0.91 (0.67-1.23) | 0.95 (0.85-1.08) | 0.94 (0.75-1.19) | 1.07 (0.98-1.17) |
| Cerebrovascular disease (CVD) | 1.01 (0.89-1.15) | 0.91 (0.87-0.96) | 0.82 (0.68-0.98) | 0.98 (0.92-1.04) | 0.95 (0.67-1.35) | 1.19 (1.06-1.33) | 0.96 (0.70-1.32) | 1.03 (0.91-1.17) | 1.05 (0.83-1.33) | 0.96 (0.88-1.06) |
| Dementia | 1.06 (0.90-1.24) | 1.39 (1.31-1.47) | 0.95 (0.77-1.18) | 0.84 (0.78-0.90) | 0.45 (0.25-0.78) | 0.82 (0.72-0.94) | 0.59 (0.36-0.96) | 0.51 (0.42-0.62) | 1.32 (1.02-1.71) | 1.15 (1.05-1.26) |
| Chronic pulmonary disease (CPD) | 1.25 (1.16-1.35) | 1.12 (1.09-1.15) | 0.83 (0.73-0.94) | 0.94 (0.90-0.98) | 0.94 (0.73-1.21) | 0.88 (0.81-0.96) | 1.17 (0.97-1.42) | 1.10 (1.01-1.20) | 1.04 (0.88-1.23) | 1.05 (0.98-1.13) |
| Rheumatologic disease (RD) | 1.03 (0.92-1.14) | 0.98 (0.94-1.02) | 1.21 (1.05-1.40) | 1.10 (1.04-1.16) | 1.18 (0.88-1.60) | 1.11 (1.00-1.25) | 1.09 (0.84-1.41) | 1.03 (0.92-1.15) | 1.30 (1.06-1.60) | 0.88 (0.80-0.97) |
| Peptic ulcer disease (PUD) | 1.34 (1.08-1.65) | 1.08 (1.00-1.17) | 0.90 (0.67-1.20) | 1.02 (0.92-1.13) | 0.75 (0.37-1.52) | 0.92 (0.74-1.14) | 0.57 (0.30-1.08) | 1.14 (0.94-1.39) | 0.57 (0.34-0.96) | 0.93 (0.79-1.11) |
| Mild Liver Disease | 1.19 (1.05-1.35) | 0.96 (0.92-1.01) | 1.03 (0.84-1.27) | 1.07 (1.00-1.15) | 0.65 (0.38-1.10) | 0.91 (0.78-1.06) | 1.09 (0.80-1.50) | 1.18 (1.04-1.33) | 1.08 (0.79-1.47) | 0.99 (0.87-1.12) |
| Moderate to Severe Liver Disease | 1.72 (1.39-2.12) | 1.44 (1.32-1.57) | 1.52 (1.19-1.93) | 1.46 (1.32-1.61) | 0.76 (0.38-1.49) | 1.0 (0.79-1.26) | 1.47 (0.99-2.17) | 1.54 (1.29-1.83) | 1.39 (0.94-2.04) | 1.85 (1.59-2.15) |
| Diabetes without Chronic Complications | 1.36 (1.25-1.49) | 1.31 (1.26-1.35) | 0.97 (0.83-1.15) | 1.11 (1.04-1.18) | 0.74 (0.52-1.07) | 1.07 (0.95-1.20) | 0.90 (0.70-1.16) | 1.20 (1.08-1.33) | 0.82 (0.65-1.04) | 1.09 (0.99-1.19) |
| Diabetes with Chronic Complications | 1.53 (1.40-1.68) | 1.32 (1.27-1.36) | 1.26 (1.10-1.43) | 1.27 (1.21-1.33) | 1.24 (0.94-1.63) | 1.10 (0.99-1.22) | 1.21 (0.96-1.52) | 1.30 (1.18-1.44) | 1.06 (0.87-1.29) | 1.16 (1.07-1.26) |
| Hemiplegia or paraplegia | 1.27 (1.01-1.60) | 1.90 (1.74-2.08) | 1.21 (0.91-1.62) | 0.98 (0.88-1.09) | 0.91 (0.49-1.70) | 0.92 (0.75-1.12) | 0.81 (0.47-1.42) | 1.00 (0.81-1.24) | 1.24 (0.82-1.86) | 1.16 (0.99-1.36) |
| Renal Disease | 1.66 (1.53-1.80) | 1.70 (1.64-1.75) | 2.09 (1.85-2.36) | 2.36 (2.25-2.47) | 1.14 (0.88-1.48) | 1.14 (1.04-1.25) | 0.93 (0.75-1.16) | 1.02 (0.93-1.12) | 1.20 (1.01-1.43) | 1.25 (1.16-1.34) |
| Any Malignancy Except Neoplasm of skin | 1.11 (1.01-1.22) | 0.96 (0.93-1.00) | 0.94 (0.82-1.09) | 0.91 (0.86-0.96) | 0.72 (0.52-0.99) | 0.79 (0.71-0.89) | 0.91 (0.72-1.17) | 0.80 (0.72-0.90) | 1.05 (0.86-1.27) | 1.02 (0.94-1.11) |
| Metastatic Solid Tumor | 2.11 (1.72-2.57) | 1.48 (1.36-1.60) | 1.04 (0.77-1.39) | 0.89 (0.79-1.00) | 1.00 (0.55-1.84) | 0.81 (0.64-1.02) | 0.66 (0.39-1.12) | 0.90 (0.73-1.12) | 1.95 (1.40-2.70) | 1.74 (1.51-2.00) |
| HIV | 0.47 (0.25-0.81) | 0.87 (0.77-0.98) | 1.38 (0.68-2.78) | 0.96 (0.81-1.14) | 0.99 (0.14-7.12) | 0.97 (0.65-1.44) | 0.48 (0.07-3.45) | 1.02 (0.75-1.39) | 2.41 (0.89-6.48) | 1.23 (0.90-1.69) |
| Obesity | 1.05 (0.99-1.11) | 1.10 (1.07-1.12) | 0.90 (0.81-1.00) | 0.97 (0.93-1.00) | 0.83 (0.67-1.03) | 0.94 (0.87-1.02) | 0.87 (0.74-1.01) | 0.93 (0.87-0.99) | 0.81 (0.70-0.94) | 1.00 (0.94-1.06) |
| Hypertension | 0.81 (0.76-0.87) | 0.81 (0.79-0.83) | 1.13 (1.00-1.28) | 1.10 (1.05-1.15) | 1.23 (0.96-1.57) | 1.02 (0.93-1.12) | 0.82 (0.68-0.98) | 0.86 (0.80-0.94) | 0.78 (0.66-0.92) | 0.82 (0.76-0.88) |
| Former or Current Tobacco User | 1.58 (1.41-1.77) | 1.45 (1.38-1.51) | 1.09 (0.91-1.30) | 1.06 (0.99-1.14) | 1.02 (0.71-1.45) | 1.15 (1.00-1.32) | 1.10 (0.87-1.41) | 0.88 (0.78-1.00) | 1.15 (0.89-1.48) | 0.95 (0.84-1.08) |
| History of Substance Abuse Disorder | 2.76 (2.39-3.18) | 2.62 (2.49-2.75) | 1.10 (0.88-1.37) | 1.00 (0.92-1.08) | 1.02 (0.63-1.64) | 0.95 (0.81-1.13) | 1.00 (0.73-1.36) | 1.08 (0.95-1.22) | 0.75 (0.51-1.11) | 0.94 (0.82-1.08) |
| Vaccination Status Prior to SARS-CoV-2 Infection |  |  |  |  |  |  |  |  |  |  |
| No Documented COVID-19 Vaccination | Reference | Reference | Reference | Reference | Reference | Reference | Reference | Reference | Reference | Reference |
| Primary Vaccination Series | 0.89 (0.73-1.08) | 0.66 (0.61-0.72) | 0.80 (0.56-1.16) | 0.65 (0.55-0.77) | 0.44 (0.16-1.18) | 0.76 (0.55-1.05) | 0.74 (0.38-1.43) | 0.76 (0.57-1.02) | 0.40 (0.21-0.74) | 0.59 (0.46-0.75) |
| Census Region |  |  |  |  |  |  |  |  |  |  |
| Midwest | Reference | Reference | Reference | Reference | Reference | Reference | Reference | Reference | Reference | Reference |
| Northeast | 0.74 (0.66-0.83) | 1.39 (1.36-1.43) | 1.85 (1.50-2.30) | 1.66 (1.58-1.74) | 1.73 (1.13-2.66) | 2.72 (2.47-2.99) | 2.62 (1.90-3.61) | 1.57 (1.43-1.72) | 1.21 (0.89-1.65) | 1.07 (1.00-1.14) |
| South | 1.88 (1.78-1.99) | 1.14 (1.11-1.17) | 2.34 (2.11-2.59) | 1.62 (1.54-1.69) | 2.39 (1.95-2.91) | 2.00 (1.81-2.21) | 4.08 (3.47-4.79) | 3.11 (2.86-3.38) | 1.82 (1.59-2.07) | 1.23 (1.15-1.32) |
| West | 1.56 (1.39-1.76) | 1.03 (1.00-1.06) | 2.11 (1.69-2.62) | 1.81 (1.70-1.91) | 1.38 (0.84-2.26) | 1.93 (1.70-2.19) | 5.35 (4.16-6.88) | 3.82 (3.48-4.20) | 1.69 (1.27-2.25) | 1.27 (1.17-1.39) |
| **Characteristic** | **Delta Dominance, June 15, 2021 – December 21, 2021** | | | | | | | | | |
|  | **Hospitalization** | | **AKI/Dialysis** | | **MACE** | | **ECMO/IMV** | | **Death** | |
|  | **Rural** | **Urban** | **Rural** | **Urban** | **Rural** | **Urban** | **Rural** | **Urban** | **Rural** | **Urban** |
|  | **Adjusted Odds Ratio (95% CI)** | **Adjusted Odds Ratio (95% CI)** | **Adjusted Hazard Ratio (95% CI)** | **Adjusted Hazard Ratio (95% CI)** | **Adjusted Hazard Ratio (95% CI)** | **Adjusted Hazard Ratio (95% CI)** | **Adjusted Hazard Ratio (95% CI)** | **Adjusted Hazard Ratio (95% CI)** | **Adjusted Hazard Ratio (95% CI)** | **Adjusted Hazard Ratio (95% CI)** |
| Sex |  |  |  |  |  |  |  |  |  |  |
| Female | Reference | Reference | Reference | Reference | Reference | Reference | Reference | Reference | Reference | Reference |
| Male | 1.27 (1.23-1.31) | 1.25 (1.23-1.27) | 1.46 (1.36-1.56) | 1.56 (1.51-1.61) | 1.35 (1.16-1.56) | 1.44 (1.34-1.55) | 1.31 (1.20-1.43) | 1.45 (1.38-1.53) | 1.28 (1.19-1.39) | 1.41 (1.34-1.48) |
| Age at COVID-19 Diagnosis | 1.05 (1.05-1.05) | 1.04 (1.04-1.04) | 1.01 (1.01-1.02) | 1.02 (1.02-1.02) | 1.03 (1.02-1.03) | 1.03 (1.03-1.03) | 1.00 (1.00-1.00) | 1.00 (1.00-1.01) | 1.04 (1.03-1.04) | 1.04 (1.04-1.04) |
| Race/Ethnicity |  |  |  |  |  |  |  |  |  |  |
| White Non-Hispanic | Reference | Reference | Reference | Reference | Reference | Reference | Reference | Reference | Reference | Reference |
| Black or African American Non-Hispanic | 1.36 (1.25-1.47) | 1.82 (1.78-1.87) | 1.79 (1.59-2.01) | 1.78 (1.71-1.86) | 1.74 (1.35-2.23) | 1.33 (1.21-1.45) | 1.05 (0.88-1.26) | 0.99 (0.92-1.05) | 1.08 (0.90-1.30) | 0.92 (0.86-0.98) |
| Hispanic or Latino Any Race | 1.67 (1.54-1.81) | 1.95 (1.89-2.01) | 1.21 (1.02-1.42) | 1.09 (1.03-1.16) | 1.11 (0.74-1.66) | 1.03 (0.90-1.18) | 1.16 (0.96-1.39) | 1.17 (1.08-1.27) | 0.92 (0.74-1.16) | 1.13 (1.04-1.23) |
| Other | 0.40 (0.34-0.46) | 0.95 (0.90-1.00) | 1.05 (0.72-1.52) | 1.27 (1.15-1.41) | 1.32 (0.63-2.79) | 1.41 (1.14-1.75) | 0.98 (0.63-1.52) | 1.37 (1.19-1.57) | 1.15 (0.77-1.70) | 1.25 (1.09-1.44) |
| Missing/Unknown | 0.90 (0.81-1.01) | 0.89 (0.86-0.93) | 1.52 (1.25-1.85) | 1.20 (1.10-1.31) | 2.40 (1.68-3.42) | 1.43 (1.19-1.70) | 1.93 (1.59-2.35) | 1.29 (1.14-1.45) | 2.01 (1.64-2.46) | 1.19 (1.04-1.35) |
| Myocardial infarction (MI) | 1.04 (0.95-1.14) | 1.11 (1.06-1.17) | 0.95 (0.83-1.08) | 0.96 (0.90-1.02) | 2.75 (2.20-3.43) | 2.60 (2.33-2.89) | 1.21 (0.99-1.49) | 0.91 (0.81-1.01) | 1.10 (0.94-1.29) | 1.04 (0.96-1.14) |
| Congestive heart failure (CHF) | 1.34 (1.24-1.44) | 1.52 (1.47-1.58) | 1.04 (0.93-1.16) | 1.08 (1.03-1.13) | 1.04 (0.83-1.31) | 1.33 (1.20-1.48) | 0.99 (0.82-1.18) | 1.02 (0.93-1.12) | 1.07 (0.93-1.22) | 1.10 (1.02-1.18) |
| Peripheral vascular disease (PVD) | 1.15 (1.04-1.26) | 1.14 (1.09-1.19) | 1.07 (0.94-1.21) | 0.96 (0.91-1.02) | 1.23 (0.95-1.58) | 0.98 (0.87-1.11) | 1.04 (0.83-1.30) | 1.04 (0.93-1.15) | 0.95 (0.81-1.13) | 1.03 (0.95-1.11) |
| Cerebrovascular disease (CVD) | 0.98 (0.89-1.08) | 0.95 (0.91-0.99) | 0.96 (0.83-1.10) | 1.0 (0.94-1.06) | 1.09 (0.83-1.43) | 1.07 (0.94-1.22) | 0.89 (0.70-1.13) | 0.93 (0.82-1.04) | 0.86 (0.72-1.03) | 0.95 (0.87-1.04) |
| Dementia | 0.91 (0.79-1.05) | 1.39 (1.30-1.48) | 0.95 (0.79-1.15) | 0.80 (0.74-0.87) | 0.57 (0.37-0.89) | 0.79 (0.66-0.94) | 0.40 (0.25-0.65) | 0.58 (0.48-0.69) | 1.08 (0.87-1.34) | 1.14 (1.03-1.26) |
| Chronic pulmonary disease (CPD) | 1.23 (1.16-1.29) | 1.18 (1.14-1.21) | 0.97 (0.88-1.06) | 0.90 (0.86-0.94) | 0.99 (0.81-1.21) | 0.86 (0.78-0.95) | 0.97 (0.84-1.12) | 1.00 (0.93-1.08) | 1.07 (0.96-1.20) | 1.07 (1.01-1.14) |
| Rheumatologic disease (RD) | 1.05 (0.97-1.14) | 0.99 (0.95-1.02) | 1.20 (1.07-1.34) | 1.09 (1.03-1.15) | 0.95 (0.73-1.22) | 1.00 (0.88-1.13) | 1.01 (0.84-1.23) | 1.14 (1.03-1.25) | 1.07 (0.93-1.25) | 1.07 (0.98-1.15) |
| Peptic ulcer disease (PUD) | 0.96 (0.82-1.12) | 1.00 (0.93-1.08) | 0.85 (0.67-1.08) | 0.98 (0.89-1.08) | 0.65 (0.35-1.18) | 1.09 (0.87-1.35) | 1.11 (0.77-1.62) | 1.10 (0.93-1.32) | 0.91 (0.67-1.25) | 1.04 (0.89-1.20) |
| Mild Liver Disease | 1.04 (0.95-1.14) | 1.01 (0.97-1.05) | 0.94 (0.80-1.10) | 1.05 (0.98-1.12) | 0.71 (0.49-1.03) | 1.00 (0.85-1.16) | 0.75 (0.58-0.96) | 1.09 (0.98-1.22) | 0.93 (0.75-1.15) | 1.06 (0.96-1.18) |
| Moderate to Severe Liver Disease | 1.69 (1.43-2.00) | 1.62 (1.49-1.76) | 1.29 (1.06-1.57) | 1.34 (1.21-1.47) | 0.72 (0.42-1.26) | 0.85 (0.65-1.11) | 1.31 (0.96-1.80) | 1.12 (0.94-1.34) | 1.58 (1.21-2.07) | 1.46 (1.26-1.69) |
| Diabetes without Chronic Complications | 1.48 (1.38-1.57) | 1.48 (1.44-1.53) | 0.94 (0.83-1.07) | 1.07 (1.01-1.14) | 1.03 (0.79-1.33) | 1.09 (0.96-1.24) | 1.08 (0.92-1.28) | 1.06 (0.97-1.16) | 1.01 (0.87-1.17) | 1.09 (1.00-1.18) |
| Diabetes with Chronic Complications | 1.52 (1.42-1.63) | 1.44 (1.40-1.50) | 1.10 (0.99-1.23) | 1.26 (1.20-1.32) | 1.12 (0.89-1.41) | 1.12 (1.00-1.25) | 1.12 (0.95-1.34) | 1.22 (1.12-1.33) | 1.14 (1.00-1.31) | 1.11 (1.03-1.19) |
| Hemiplegia or paraplegia | 1.38 (1.14-1.65) | 1.75 (1.61-1.91) | 0.98 (0.77-1.26) | 0.93 (0.83-1.03) | 1.16 (0.73-1.86) | 1.01 (0.81-1.25) | 0.67 (0.41-1.09) | 0.90 (0.74-1.10) | 1.12 (0.81-1.56) | 0.96 (0.82-1.13) |
| Renal Disease | 1.59 (1.49-1.69) | 1.70 (1.65-1.75) | 2.17 (1.97-2.38) | 2.22 (2.13-2.32) | 1.12 (0.91-1.39) | 1.11 (1.00-1.23) | 1.06 (0.91-1.23) | 1.05 (0.97-1.14) | 1.22 (1.08-1.38) | 1.24 (1.16-1.32) |
| Any Malignancy Except Neoplasm of skin | 1.03 (0.96-1.10) | 0.97 (0.94-1.00) | 0.97 (0.86-1.08) | 0.93 (0.88-0.98) | 0.87 (0.67-1.12) | 0.89 (0.79-1.00) | 0.75 (0.62-0.92) | 0.84 (0.76-0.93) | 1.02 (0.89-1.18) | 1.05 (0.98-1.13) |
| Metastatic Solid Tumor | 1.97 (1.69-2.28) | 1.62 (1.50-1.75) | 0.84 (0.66-1.06) | 0.83 (0.74-0.93) | 0.92 (0.56-1.50) | 0.84 (0.65-1.08) | 1.00 (0.72-1.40) | 0.83 (0.68-1.01) | 1.74 (1.39-2.18) | 1.77 (1.56-2.00) |
| HIV | 0.57 (0.35-0.88) | 0.91 (0.81-1.03) | 0.85 (0.40-1.79) | 0.94 (0.78-1.12) | 0.88 (0.22-3.57) | 0.96 (0.62-1.50) | 1.14 (0.42-3.06) | 0.81 (0.58-1.14) | 1.43 (0.59-3.47) | 0.74 (0.50-1.08) |
| Obesity | 0.93 (0.89-0.97) | 1.06 (1.04-1.08) | 1.18 (1.09-1.28) | 1.12 (1.08-1.16) | 0.98 (0.82-1.16) | 0.91 (0.83-0.99) | 1.21 (1.09-1.35) | 1.21 (1.14-1.28) | 0.98 (0.89-1.09) | 1.11 (1.05-1.17) |
| Hypertension | 0.92 (0.88-0.97) | 0.91 (0.89-0.93) | 1.23 (1.12-1.35) | 1.22 (1.17-1.28) | 1.30 (1.06-1.58) | 1.10 (1.00-1.21) | 0.92 (0.81-1.05) | 0.95 (0.89-1.02) | 0.91 (0.82-1.01) | 0.91 (0.86-0.97) |
| Former or Current Tobacco User | 1.37 (1.27-1.47) | 1.37 (1.32-1.42) | 1.03 (0.91-1.17) | 1.07 (1.01-1.14) | 1.12 (0.88-1.44) | 1.17 (1.03-1.33) | 0.77 (0.65-0.92) | 0.96 (0.87-1.05) | 0.94 (0.80-1.11) | 0.97 (0.88-1.07) |
| History of Substance Abuse Disorder | 2.07 (1.88-2.28) | 2.32 (2.22-2.42) | 1.07 (0.91-1.25) | 0.98 (0.92-1.05) | 1.39 (0.99-1.95) | 1.01 (0.87-1.19) | 1.18 (0.96-1.46) | 1.00 (0.90-1.12) | 0.96 (0.77-1.21) | 0.99 (0.88-1.10) |
| Vaccination Status Prior to SARS-CoV-2 Infection |  |  |  |  |  |  |  |  |  |  |
| No Documented COVID-19 Vaccination | Reference | Reference | Reference | Reference | Reference | Reference | Reference | Reference | Reference | Reference |
| Primary Vaccination Series | 0.34 (0.33-0.36) | 0.37 (0.36-0.38) | 0.81 (0.72-0.91) | 0.76 (0.72-0.80) | 0.88 (0.70-1.11) | 0.72 (0.63-0.81) | 0.44 (0.36-0.55) | 0.55 (0.49-0.60) | 0.56 (0.49-0.64) | 0.61 (0.57-0.66) |
| Primary+ Vaccination Series | 0.39 (0.32-0.48) | 0.39 (0.35-0.42) | 0.97 (0.69-1.35) | 0.91 (0.78-1.06) | 0.70 (0.29-1.69) | 0.56 (0.36-0.87) | 0.64 (0.32-1.29) | 0.53 (0.37-0.75) | 0.47 (0.28-0.79) | 0.64 (0.51-0.81) |
| Census Region |  |  |  |  |  |  |  |  |  |  |
| Midwest | Reference | Reference | Reference | Reference | Reference | Reference | Reference | Reference | Reference | Reference |
| Northeast | 0.63 (0.58-0.68) | 1.01 (0.98-1.04) | 1.88 (1.62-2.19) | 1.34 (1.26-1.42) | 2.00 (1.48-2.70) | 1.55 (1.37-1.75) | 2.31 (1.92-2.80) | 1.24 (1.12-1.37) | 1.14 (0.95-1.37) | 0.81 (0.74-0.88) |
| South | 1.79 (1.72-1.86) | 1.08 (1.06-1.10) | 2.44 (2.26-2.64) | 1.61 (1.55-1.68) | 2.34 (1.98-2.75) | 1.83 (1.67-2.00) | 2.50 (2.26-2.76) | 2.31 (2.17-2.46) | 1.46 (1.33-1.59) | 1.18 (1.11-1.24) |
| West | 1.98 (1.82-2.15) | 0.99 (0.96-1.01) | 3.13 (2.73-3.57) | 1.72 (1.63-1.81) | 2.18 (1.60-2.96) | 1.47 (1.31-1.66) | 4.52 (3.91-5.24) | 2.70 (2.51-2.91) | 2.17 (1.87-2.52) | 1.17 (1.09-1.26) |
| **Characteristic** | **Omicron Dominance, December 22, 2021 – December 31, 2022** | | | | | | | | | |
|  | **Hospitalization** | | **AKI/Dialysis** | | **MACE** | | **ECMO/IMV** | | **Death** | |
|  | **Rural** | **Urban** | **Rural** | **Urban** | **Rural** | **Urban** | **Rural** | **Urban** | **Rural** | **Urban** |
|  | **Adjusted Odds Ratio (95% CI)** | **Adjusted Odds Ratio (95% CI)** | **Adjusted Hazard Ratio (95% CI)** | **Adjusted Hazard Ratio (95% CI)** | **Adjusted Hazard Ratio (95% CI)** | **Adjusted Hazard Ratio (95% CI)** | **Adjusted Hazard Ratio (95% CI)** | **Adjusted Hazard Ratio (95% CI)** | **Adjusted Hazard Ratio (95% CI)** | **Adjusted Hazard Ratio (95% CI)** |
| Sex |  |  |  |  |  |  |  |  |  |  |
| Female | Reference | Reference | Reference | Reference | Reference | Reference | Reference | Reference | Reference | Reference |
| Male | 1.21 (1.18-1.25) | 1.15 (1.13-1.16) | 1.37 (1.29-1.45) | 1.39 (1.36-1.43) | 1.49 (1.34-1.66) | 1.40 (1.33-1.47) | 1.63 (1.46-1.81) | 1.63 (1.55-1.71) | 1.27 (1.17-1.39) | 1.32 (1.27-1.38) |
| Age at COVID-19 Diagnosis | 1.04 (1.04-1.04) | 1.03 (1.03-1.03) | 1.01 (1.01-1.02) | 1.02 (1.01-1.02) | 1.02 (1.02-1.03) | 1.03 (1.02-1.03) | 1.00 (1.00-1.00) | 1.00 (1.00-1.00) | 1.03 (1.03-1.04) | 1.04 (1.03-1.04) |
| Race/Ethnicity |  |  |  |  |  |  |  |  |  |  |
| White Non-Hispanic | Reference | Reference | Reference | Reference | Reference | Reference | Reference | Reference | Reference | Reference |
| Black or African American Non-Hispanic | 1.19 (1.12-1.27) | 1.67 (1.64-1.70) | 1.65 (1.51-1.81) | 1.51 (1.47-1.56) | 1.41 (1.18-1.68) | 1.23 (1.16-1.31) | 1.26 (1.06-1.50) | 1.05 (0.98-1.11) | 1.08 (0.91-1.29) | 0.89 (0.84-0.94) |
| Hispanic or Latino Any Race | 1.37 (1.27-1.49) | 1.67 (1.63-1.71) | 1.11 (0.94-1.32) | 1.09 (1.04-1.14) | 0.90 (0.63-1.29) | 1.03 (0.94-1.13) | 1.26 (0.99-1.60) | 1.04 (0.96-1.13) | 1.21 (0.95-1.56) | 1.01 (0.93-1.09) |
| Other | 0.46 (0.41-0.52) | 0.97 (0.94-1.00) | 1.35 (1.07-1.72) | 1.24 (1.16-1.32) | 1.43 (0.93-2.20) | 1.26 (1.12-1.43) | 1.61 (1.11-2.33) | 1.29 (1.14-1.45) | 1.33 (0.93-1.89) | 1.10 (0.99-1.23) |
| Missing/Unknown | 0.88 (0.79-0.98) | 0.96 (0.93-0.98) | 1.37 (1.12-1.67) | 1.14 (1.07-1.21) | 1.61 (1.15-2.24) | 1.33 (1.19-1.48) | 1.71 (1.31-2.23) | 1.30 (1.17-1.44) | 1.61 (1.23-2.12) | 1.08 (0.97-1.19) |
| Myocardial infarction (MI) | 1.15 (1.08-1.23) | 1.23 (1.19-1.26) | 0.98 (0.90-1.08) | 1.04 (1.00-1.08) | 2.79 (2.41-3.23) | 2.67 (2.51-2.85) | 1.07 (0.89-1.29) | 1.12 (1.03-1.22) | 1.07 (0.93-1.24) | 1.10 (1.03-1.17) |
| Congestive heart failure (CHF) | 1.73 (1.64-1.82) | 1.90 (1.86-1.94) | 1.08 (1.00-1.16) | 1.06 (1.03-1.10) | 1.33 (1.14-1.54) | 1.18 (1.10-1.25) | 1.11 (0.94-1.30) | 1.13 (1.05-1.21) | 1.13 (1.00-1.27) | 1.19 (1.12-1.26) |
| Peripheral vascular disease (PVD) | 1.08 (1.02-1.15) | 1.10 (1.07-1.13) | 0.92 (0.84-1.01) | 0.98 (0.95-1.02) | 1.03 (0.87-1.22) | 1.08 (1.00-1.16) | 0.90 (0.74-1.09) | 0.92 (0.85-1.01) | 0.96 (0.83-1.11) | 1.06 (0.99-1.13) |
| Cerebrovascular disease (CVD) | 0.94 (0.89-1.00) | 1.02 (0.99-1.05) | 0.91 (0.83-1.00) | 0.95 (0.92-0.99) | 1.23 (1.04-1.46) | 1.19 (1.11-1.28) | 0.99 (0.81-1.22) | 0.98 (0.90-1.07) | 1.04 (0.90-1.21) | 0.92 (0.87-0.99) |
| Dementia | 1.26 (1.16-1.36) | 1.72 (1.67-1.78) | 0.88 (0.78-1.00) | 0.83 (0.79-0.87) | 0.55 (0.42-0.71) | 0.65 (0.59-0.72) | 0.46 (0.32-0.66) | 0.68 (0.60-0.76) | 1.24 (1.05-1.45) | 1.24 (1.16-1.33) |
| Chronic pulmonary disease (CPD) | 1.30 (1.25-1.35) | 1.18 (1.16-1.20) | 0.92 (0.85-0.98) | 0.92 (0.90-0.95) | 0.76 (0.67-0.88) | 0.91 (0.86-0.97) | 1.12 (0.98-1.29) | 1.09 (1.02-1.16) | 1.10 (0.99-1.22) | 1.07 (1.02-1.13) |
| Rheumatologic disease (RD) | 0.98 (0.93-1.03) | 0.92 (0.90-0.95) | 1.10 (1.01-1.20) | 1.12 (1.08-1.16) | 0.86 (0.72-1.02) | 0.98 (0.91-1.06) | 1.00 (0.83-1.20) | 1.11 (1.02-1.20) | 0.99 (0.86-1.13) | 1.04 (0.98-1.11) |
| Peptic ulcer disease (PUD) | 1.08 (0.97-1.20) | 1.10 (1.05-1.15) | 1.03 (0.88-1.20) | 1.15 (1.08-1.21) | 0.72 (0.51-1.02) | 0.96 (0.84-1.09) | 0.94 (0.68-1.30) | 1.02 (0.90-1.16) | 0.94 (0.73-1.21) | 1.00 (0.90-1.12) |
| Mild Liver Disease | 0.98 (0.92-1.05) | 0.99 (0.96-1.02) | 0.99 (0.88-1.12) | 1.00 (0.96-1.05) | 0.79 (0.61-1.01) | 0.94 (0.85-1.04) | 0.94 (0.75-1.18) | 1.08 (0.98-1.18) | 1.05 (0.87-1.27) | 1.02 (0.93-1.11) |
| Moderate to Severe Liver Disease | 1.71 (1.54-1.91) | 1.76 (1.68-1.85) | 1.21 (1.04-1.39) | 1.34 (1.27-1.42) | 0.80 (0.56-1.14) | 0.87 (0.75-1.01) | 1.41 (1.09-1.83) | 1.51 (1.34-1.69) | 1.87 (1.50-2.31) | 1.81 (1.64-2.00) |
| Diabetes without Chronic Complications | 1.42 (1.35-1.50) | 1.33 (1.30-1.36) | 1.07 (0.97-1.19) | 1.05 (1.01-1.10) | 1.01 (0.83-1.23) | 1.00 (0.91-1.09) | 0.88 (0.73-1.08) | 0.96 (0.88-1.05) | 1.09 (0.94-1.26) | 0.95 (0.88-1.03) |
| Diabetes with Chronic Complications | 1.50 (1.42-1.57) | 1.33 (1.30-1.36) | 1.34 (1.24-1.45) | 1.25 (1.21-1.29) | 1.28 (1.10-1.50) | 1.13 (1.06-1.22) | 1.08 (0.91-1.27) | 1.18 (1.09-1.27) | 1.10 (0.96-1.25) | 1.03 (0.96-1.09) |
| Hemiplegia or paraplegia | 1.84 (1.65-2.06) | 2.04 (1.94-2.13) | 0.88 (0.74-1.04) | 0.93 (0.88-0.99) | 0.88 (0.66-1.18) | 0.86 (0.77-0.98) | 1.17 (0.86-1.60) | 1.06 (0.93-1.21) | 1.01 (0.78-1.30) | 1.15 (1.03-1.28) |
| Renal Disease | 1.67 (1.60-1.75) | 1.97 (1.93-2.00) | 2.41 (2.23-2.59) | 2.49 (2.42-2.57) | 1.01 (0.88-1.17) | 0.96 (0.90-1.02) | 1.11 (0.96-1.28) | 1.07 (1.00-1.14) | 1.18 (1.06-1.33) | 1.18 (1.12-1.25) |
| Any Malignancy Except Neoplasm of skin | 1.09 (1.04-1.15) | 0.98 (0.96-1.00) | 0.99 (0.91-1.07) | 0.92 (0.89-0.96) | 0.84 (0.71-0.99) | 0.78 (0.73-0.84) | 0.99 (0.84-1.16) | 0.94 (0.87-1.01) | 1.31 (1.17-1.48) | 1.20 (1.14-1.28) |
| Metastatic Solid Tumor | 2.08 (1.89-2.29) | 2.06 (1.98-2.15) | 1.04 (0.89-1.20) | 0.97 (0.91-1.03) | 0.65 (0.46-0.93) | 0.71 (0.61-0.82) | 0.68 (0.48-0.96) | 0.94 (0.82-1.08) | 2.15 (1.79-2.58) | 2.62 (2.42-2.83) |
| HIV | 1.16 (0.89-1.51) | 1.08 (1.00-1.16) | 0.84 (0.54-1.33) | 0.96 (0.85-1.08) | 0.54 (0.17-1.67) | 0.93 (0.70-1.22) | 0.14 (0.02-1.01) | 0.91 (0.71-1.16) | 0.82 (0.34-1.99) | 1.03 (0.79-1.34) |
| Obesity | 0.77 (0.74-0.79) | 0.85 (0.83-0.86) | 1.06 (0.99-1.13) | 1.04 (1.02-1.07) | 1.04 (0.92-1.19) | 0.93 (0.88-0.99) | 1.07 (0.94-1.20) | 1.03 (0.97-1.09) | 0.83 (0.75-0.93) | 0.94 (0.89-0.99) |
| Hypertension | 0.83 (0.80-0.86) | 0.82 (0.81-0.83) | 1.08 (1.00-1.17) | 1.09 (1.06-1.13) | 0.92 (0.79-1.06) | 1.05 (0.98-1.12) | 0.82 (0.72-0.95) | 0.84 (0.78-0.89) | 0.87 (0.78-0.97) | 0.79 (0.75-0.84) |
| Former or Current Tobacco User | 1.90 (1.80-1.99) | 1.69 (1.65-1.73) | 1.10 (1.01-1.20) | 1.12 (1.08-1.17) | 1.35 (1.16-1.58) | 1.26 (1.17-1.36) | 1.07 (0.92-1.23) | 1.06 (0.98-1.14) | 0.98 (0.85-1.13) | 0.99 (0.92-1.06) |
| History of Substance Abuse Disorder | 2.46 (2.30-2.63) | 3.01 (2.92-3.09) | 1.05 (0.94-1.18) | 1.01 (0.97-1.06) | 0.83 (0.66-1.05) | 0.93 (0.85-1.03) | 1.24 (1.03-1.48) | 1.26 (1.16-1.36) | 1.03 (0.84-1.25) | 1.02 (0.93-1.11) |
| Vaccination Status Prior to SARS-CoV-2 Infection |  |  |  |  |  |  |  |  |  |  |
| No Documented COVID-19 Vaccination | Reference | Reference | Reference | Reference | Reference | Reference | Reference | Reference | Reference | Reference |
| Primary Vaccination Series | 0.63 (0.61-0.66) | 0.68 (0.67-0.70) | 0.75 (0.68-0.82) | 0.83 (0.80-0.86) | 0.81 (0.69-0.96) | 0.74 (0.68-0.80) | 0.68 (0.57-0.81) | 0.74 (0.68-0.79) | 0.74 (0.65-0.84) | 0.81 (0.76-0.86) |
| Primary+ Vaccination Series | 0.47 (0.45-0.49) | 0.51 (0.50-0.51) | 0.70 (0.64-0.77) | 0.73 (0.70-0.76) | 0.72 (0.61-0.86) | 0.67 (0.62-0.73) | 0.64 (0.53-0.77) | 0.64 (0.59-0.70) | 0.55 (0.48-0.63) | 0.62 (0.58-0.66) |
| Census Region |  |  |  |  |  |  |  |  |  |  |
| Midwest | Reference | Reference | Reference | Reference | Reference | Reference | Reference | Reference | Reference | Reference |
| Northeast | 0.68 (0.65-0.72) | 1.40 (1.37-1.42) | 1.36 (1.22-1.51) | 1.09 (1.05-1.14) | 1.69 (1.41-2.02) | 1.10 (1.02-1.19) | 1.49 (1.24-1.79) | 0.81 (0.75-0.88) | 0.80 (0.68-0.94) | 0.79 (0.74-0.84) |
| South | 1.73 (1.67-1.79) | 1.17 (1.16-1.19) | 1.61 (1.50-1.72) | 1.23 (1.20-1.27) | 1.59 (1.41-1.81) | 1.38 (1.30-1.47) | 1.60 (1.42-1.80) | 1.42 (1.34-1.51) | 1.06 (0.96-1.16) | 1.01 (0.96-1.06) |
| West | 1.33 (1.23-1.43) | 0.79 (0.78-0.81) | 2.15 (1.88-2.46) | 1.31 (1.26-1.36) | 1.24 (0.92-1.66) | 1.13 (1.04-1.22) | 2.65 (2.17-3.23) | 1.47 (1.36-1.58) | 1.40 (1.15-1.70) | 0.90 (0.84-0.96) |

**eTable S-5. Multivariable Regression for Adverse Acute COVID-19 Events with COVID-19 Therapies Across All Time Periods**

| **Characteristic** | **All Medications Combined** | | | | | |
| --- | --- | --- | --- | --- | --- | --- |
|  | **Hospitalization** | **AKI/Dialysis** | **MACE** | **IMV/ECMO** | **Death** | **Death After IMV/ECMO** |
|  | **Adjusted Odds Ratio**  **(95% CI)** | **Adjusted Hazard Ratio**  **(95% CI)** | **Adjusted Hazard Ratio**  **(95% CI)** | **Adjusted Hazard Ratio**  **(95% CI)** | **Adjusted Hazard Ratio**  **(95% CI)** | **Adjusted Hazard Ratio**  **(95% CI)** |
| Sex |  |  |  |  |  |  |
| Female | Reference | Reference | Reference | Reference | Reference | Reference |
| Male | 1.23 (1.22-1.24) | 1.49 (1.46-1.51) | 1.48 (1.43-1.53) | 1.61 (1.57-1.66) | 1.36 (1.32-1.39) | 1.04 (1.01-1.08) |
| Age at COVID-19 Diagnosis | 1.04 (1.04-1.04) | 1.02 (1.02-1.02) | 1.03 (1.03-1.03) | 1.00 (1.00-1.01) | 1.04 (1.04-1.04) | 1.03 (1.03-1.03) |
| Race/Ethnicity |  |  |  |  |  |  |
| White Non-Hispanic | Reference | Reference | Reference | Reference | Reference | Reference |
| Black or African American Non-Hispanic | 1.69 (1.67-1.71) | 1.65 (1.62-1.68) | 1.33 (1.28-1.39) | 1.08 (1.04-1.12) | 0.94 (0.91-0.97) | 1.02 (0.98-1.07) |
| Hispanic or Latino Any Race | 1.83 (1.80-1.85) | 1.13 (1.10-1.16) | 1.03 (0.97-1.09) | 1.16 (1.11-1.21) | 1.12 (1.07-1.17) | 1.17 (1.11-1.24) |
| Other | 0.97 (0.95-0.99) | 1.23 (1.18-1.28) | 1.30 (1.20-1.41) | 1.27 (1.19-1.36) | 1.18 (1.10-1.25) | 1.18 (1.07-1.29) |
| Missing/Unknown | 0.95 (0.93-0.96) | 1.25 (1.20-1.30) | 1.38 (1.28-1.48) | 1.39 (1.31-1.47) | 1.29 (1.22-1.37) | 1.23 (1.14-1.33) |
| Myocardial infarction (MI) | 1.18 (1.16-1.21) | 0.99 (0.96-1.01) | 2.58 (2.47-2.70) | 1.02 (0.96-1.07) | 1.05 (1.00-1.09) | 1.04 (0.98-1.11) |
| Congestive heart failure (CHF) | 1.67 (1.65-1.70) | 0.98 (0.96-1.01) | 1.13 (1.08-1.18) | 0.97 (0.93-1.01) | 1.11 (1.07-1.15) | 0.88 (0.83-0.93) |
| Peripheral vascular disease (PVD) | 1.09 (1.06-1.11) | 0.96 (0.93-0.98) | 1.02 (0.97-1.07) | 0.94 (0.89-0.99) | 1.03 (0.99-1.07) | 1.05 (0.99-1.12) |
| Cerebrovascular disease (CVD) | 0.97 (0.96-0.99) | 0.95 (0.92-0.97) | 1.15 (1.10-1.21) | 0.96 (0.91-1.01) | 0.93 (0.89-0.97) | 0.99 (0.93-1.06) |
| Dementia | 1.41 (1.37-1.44) | 0.82 (0.80-0.85) | 0.67 (0.63-0.72) | 0.56 (0.51-0.60) | 1.15 (1.10-1.21) | 1.13 (1.04-1.22) |
| Chronic pulmonary disease (CPD) | 1.19 (1.18-1.20) | 0.97 (0.95-0.99) | 0.94 (0.90-0.98) | 1.07 (1.03-1.11) | 1.04 (1.01-1.08) | 0.89 (0.85-0.93) |
| Rheumatologic disease (RD) | 0.98 (0.96-1.00) | 1.12 (1.09-1.14) | 1.02 (0.97-1.08) | 1.11 (1.05-1.16) | 1.01 (0.97-1.06) | 1.02 (0.97-1.08) |
| Peptic ulcer disease (PUD) | 1.09 (1.05-1.12) | 1.04 (1.00-1.08) | 0.92 (0.84-1.01) | 1.02 (0.93-1.11) | 0.96 (0.89-1.03) | 0.97 (0.87-1.07) |
| Liver Disease |  |  |  |  |  |  |
| No History of Liver Disease | Reference | Reference | Reference | Reference | Reference | Reference |
| Mild Liver Disease | 0.99 (0.97-1.01) | 1.03 (1.00-1.06) | 0.92 (0.86-0.99) | 1.07 (1.01-1.13) | 1.02 (0.96-1.07) | 0.98 (0.91-1.05) |
| Moderate to Severe Liver Disease | 1.71 (1.65-1.76) | 1.25 (1.21-1.31) | 0.78 (0.71-0.87) | 1.28 (1.18-1.38) | 1.67 (1.56-1.78) | 1.38 (1.26-1.51) |
| Diabetes Mellitus |  |  |  |  |  |  |
| No History of Diabetes | Reference | Reference | Reference | Reference | Reference | Reference |
| Diabetes without Chronic Complications | 1.39 (1.37-1.42) | 1.06 (1.03-1.09) | 1.03 (0.97-1.09) | 1.03 (0.98-1.09) | 1.03 (0.99-1.07) | 1.00 (0.94-1.06) |
| Diabetes with Chronic Complications | 1.38 (1.36-1.40) | 1.19 (1.17-1.22) | 1.10 (1.05-1.16) | 1.17 (1.12-1.23) | 1.09 (1.05-1.13) | 1.01 (0.95-1.06) |
| Hemiplegia or paraplegia | 1.84 (1.78-1.91) | 0.93 (0.89-0.97) | 0.88 (0.81-0.96) | 0.96 (0.87-1.05) | 1.07 (1.00-1.15) | 1.00 (0.90-1.12) |
| Renal Disease | 1.78 (1.76-1.80) | 1.98 (1.94-2.02) | 0.89 (0.85-0.93) | 0.91 (0.88-0.95) | 1.16 (1.13-1.20) | 1.09 (1.04-1.15) |
| Cancer |  |  |  |  |  |  |
| No History of Malignant Cancer | Reference | Reference | Reference | Reference | Reference | Reference |
| Any Malignancy Except Neoplasm of skin | 0.99 (0.98-1.01) | 0.92 (0.89-0.94) | 0.80 (0.76-0.84) | 0.84 (0.80-0.88) | 1.10 (1.06-1.14) | 1.13 (1.07-1.19) |
| Metastatic Solid Tumor | 1.93 (1.87-1.99) | 0.89 (0.85-0.93) | 0.72 (0.65-0.80) | 0.79 (0.72-0.87) | 2.03 (1.92-2.14) | 1.66 (1.51-1.83) |
| HIV | 1.03 (0.97-1.09) | 0.94 (0.87-1.02) | 0.91 (0.75-1.11) | 0.87 (0.74-1.02) | 1.01 (0.85-1.19) | 1.20 (0.96-1.49) |
| Obesity | 1.00 (0.99-1.01) | 1.23 (1.21-1.25) | 1.14 (1.10-1.18) | 1.23 (1.19-1.27) | 1.00 (0.97-1.03) | 0.87 (0.83-0.90) |
| Hypertension | 0.85 (0.84-0.86) | 1.12 (1.09-1.14) | 1.05 (1.01-1.10) | 0.86 (0.83-0.90) | 0.84 (0.81-0.87) | 0.91 (0.86-0.95) |
| Former or Current Tobacco User | 1.55 (1.52-1.58) | 1.11 (1.08-1.14) | 1.28 (1.21-1.34) | 0.97 (0.92-1.01) | 0.95 (0.90-0.99) | 0.90 (0.85-0.96) |
| History of Substance Abuse Disorder | 2.58 (2.53-2.63) | 0.97 (0.94-1.00) | 0.91 (0.85-0.97) | 1.07 (1.01-1.12) | 0.97 (0.91-1.02) | 1.02 (0.95-1.10) |
| Vaccination Status Prior to SARS-CoV-2 Infection |  |  |  |  |  |  |
| No Documented COVID-19 Vaccination | Reference | Reference | Reference | Reference | Reference | Reference |
| Primary Vaccination Series | 0.52 (0.52-0.53) | 0.76 (0.74-0.78) | 0.71 (0.68-0.75) | 0.58 (0.55-0.61) | 0.68 (0.65-0.71) | 0.83 (0.78-0.89) |
| Primary+ Vaccination Series | 0.42 (0.42-0.43) | 0.69 (0.67-0.72) | 0.68 (0.64-0.73) | 0.50 (0.47-0.54) | 0.49 (0.47-0.52) | 0.57 (0.52-0.62) |
| Census Region |  |  |  |  |  |  |
| Midwest | Reference | Reference | Reference | Reference | Reference | Reference |
| Northeast | 1.16 (1.15-1.18) | 1.78 (1.74-1.82) | 2.39 (2.27-2.50) | 1.43 (1.36-1.49) | 0.88 (0.85-0.92) | 1.11 (1.05-1.18) |
| South | 1.32 (1.31-1.33) | 2.02 (1.98-2.06) | 2.55 (2.45-2.65) | 2.58 (2.49-2.67) | 1.14 (1.11-1.17) | 1.11 (1.07-1.16) |
| West | 0.88 (0.87-0.89) | 1.78 (1.73-1.83) | 1.67 (1.58-1.77) | 2.75 (2.64-2.87) | 1.10 (1.06-1.14) | 0.88 (0.83-0.93) |
| Rurality |  |  |  |  |  |  |
| Urban | Reference | Reference | Reference | Reference | Reference | Reference |
| Urban-Adjacent Rural | 1.07 (1.05-1.08) | 1.00 (0.98-1.03) | 1.02 (0.97-1.06) | 1.32 (1.27-1.37) | 1.30 (1.26-1.35) | 1.19 (1.14-1.24) |
| Nonurban-Adjacent Rural | 1.06 (1.03-1.08) | 1.08 (1.03-1.13) | 1.05 (0.97-1.14) | 1.58 (1.49-1.68) | 1.37 (1.30-1.45) | 1.28 (1.18-1.38) |
| Nirmatrelvir/ritonavir | 0.06 (0.06-0.07) | 0.36 (0.30-0.43) | 0.62 (0.46-0.83) | 0.17 (0.11-0.27) | 0.38 (0.28-0.50) | 0.54 (0.32-0.90) |
| Molnupiravir | 0.06 (0.05-0.07) | 0.63 (0.49-0.80) | 0.70 (0.44-1.11) | 0.72 (0.45-1.14) | 0.68 (0.46-1.01) | 1.10 (0.66-1.83) |
| Monoclonal Therapies | 0.11 (0.11-0.12) | 0.67 (0.63-0.72) | 0.57 (0.49-0.66) | 0.52 (0.46-0.58) | 0.56 (0.51-0.62) | 0.67 (0.59-0.76) |
| Remdesivir |  | 0.49 (0.48-0.50) | 0.34 (0.32-0.36) | 0.92 (0.89-0.96) | 1.33 (1.29-1.37) | 0.79 (0.76-0.82) |
| Anticoagulants |  | 0.21 (0.20-0.21) | 0.18 (0.17-0.19) | 0.35 (0.34-0.37) | 0.80 (0.78-0.83) | 0.54 (0.52-0.56) |
| Tocilizumab |  |  |  |  |  | 1.36 (1.29-1.44) |
| Dexamethasone |  |  |  |  |  | 0.95 (0.91-0.98) |

**eTable S-6. Multivariable Regression for Adverse Acute COVID-19 Events with COVID-19 Therapies Stratified by Rurality Across All Time Periods**

**A. Hospitalization, Acute Kidney Injury (AKI) or Dialysis, and Major Adverse Cardiovascular Event (MACE)**

| **Characteristic** | **Hospitalization** | | **AKI/Dialysis** | | **MACE** | |
| --- | --- | --- | --- | --- | --- | --- |
|  | **Urban** | **Rural** | **Urban** | **Rural** | **Urban** | **Rural** |
|  | **Adjusted Odds Ratio**  **(95% CI)** | **Adjusted Odds Ratio**  **(95% CI)** | **Adjusted Hazard Ratio**  **(95% CI)** | **Adjusted Hazard Ratio**  **(95% CI)** | **Adjusted Hazard Ratio**  **(95% CI)** | **Adjusted Hazard Ratio**  **(95% CI)** |
| Sex |  |  |  |  |  |  |
| Female | Reference | Reference | Reference | Reference | Reference | Reference |
| Male | 1.23 (1.22-1.24) | 1.25 (1.23-1.28) | 1.50 (1.47-1.53) | 1.42 (1.37-1.48) | 1.48 (1.43-1.53) | 1.51 (1.39-1.63) |
| Age at COVID-19 Diagnosis | 1.04 (1.04-1.04) | 1.04 (1.04-1.04) | 1.02 (1.02-1.02) | 1.02 (1.01-1.02) | 1.03 (1.03-1.03) | 1.03 (1.02-1.03) |
| Race/Ethnicity |  |  |  |  |  |  |
| White Non-Hispanic | Reference | Reference | Reference | Reference | Reference | Reference |
| Black or African American Non-Hispanic | 1.72 (1.70-1.74) | 1.18 (1.13-1.23) | 1.63 (1.60-1.66) | 1.70 (1.60-1.81) | 1.31 (1.25-1.36) | 1.50 (1.32-1.70) |
| Hispanic or Latino Any Race | 1.83 (1.80-1.86) | 1.51 (1.43-1.58) | 1.11 (1.08-1.15) | 1.24 (1.12-1.37) | 1.01 (0.95-1.08) | 1.06 (0.84-1.33) |
| Other | 1.05 (1.02-1.07) | 0.39 (0.36-0.43) | 1.22 (1.17-1.28) | 1.14 (0.95-1.38) | 1.28 (1.18-1.39) | 1.28 (0.91-1.79) |
| Missing/Unknown | 0.95 (0.93-0.97) | 0.87 (0.81-0.93) | 1.20 (1.15-1.25) | 1.60 (1.41-1.81) | 1.31 (1.21-1.41) | 1.93 (1.56-2.41) |
| Myocardial infarction (MI) | 1.20 (1.17-1.23) | 1.10 (1.05-1.16) | 1.00 (0.98-1.03) | 0.90 (0.85-0.97) | 2.60 (2.48-2.73) | 2.44 (2.18-2.73) |
| Congestive heart failure (CHF) | 1.70 (1.67-1.73) | 1.52 (1.46-1.58) | 0.98 (0.95-1.00) | 1.02 (0.96-1.08) | 1.13 (1.08-1.19) | 1.11 (0.99-1.25) |
| Peripheral vascular disease (PVD) | 1.08 (1.06-1.11) | 1.11 (1.06-1.16) | 0.95 (0.93-0.98) | 0.98 (0.92-1.05) | 1.01 (0.96-1.07) | 1.09 (0.96-1.24) |
| Cerebrovascular disease (CVD) | 0.97 (0.95-0.99) | 0.98 (0.93-1.02) | 0.95 (0.92-0.98) | 0.93 (0.87-1.01) | 1.14 (1.08-1.21) | 1.19 (1.04-1.35) |
| Dementia | 1.49 (1.45-1.53) | 1.09 (1.02-1.16) | 0.81 (0.78-0.84) | 0.92 (0.84-1.01) | 0.69 (0.65-0.74) | 0.53 (0.43-0.66) |
| Chronic pulmonary disease (CPD) | 1.17 (1.16-1.19) | 1.28 (1.25-1.32) | 0.97 (0.95-0.99) | 0.95 (0.90-1.00) | 0.95 (0.91-1.00) | 0.88 (0.80-0.98) |
| Rheumatologic disease (RD) | 0.97 (0.95-0.99) | 1.04 (1.00-1.08) | 1.12 (1.09-1.15) | 1.12 (1.05-1.19) | 1.04 (0.99-1.10) | 0.92 (0.80-1.05) |
| Peptic ulcer disease (PUD) | 1.08 (1.05-1.12) | 1.11 (1.02-1.21) | 1.05 (1.01-1.10) | 0.94 (0.83-1.06) | 0.96 (0.87-1.06) | 0.72 (0.55-0.95) |
| Liver Disease |  |  |  |  |  |  |
| No History of Liver Disease | Reference | Reference | Reference | Reference | Reference | Reference |
| Mild Liver Disease | 0.98 (0.96-1.00) | 1.03 (0.98-1.09) | 1.04 (1.01-1.08) | 0.98 (0.90-1.07) | 0.96 (0.89-1.03) | 0.74 (0.61-0.90) |
| Moderate to Severe Liver Disease | 1.69 (1.63-1.75) | 1.70 (1.56-1.85) | 1.27 (1.21-1.32) | 1.17 (1.05-1.30) | 0.80 (0.72-0.90) | 0.67 (0.51-0.87) |
| Diabetes Mellitus |  |  |  |  |  |  |
| No History of Diabetes | Reference | Reference | Reference | Reference | Reference | Reference |
| Diabetes without Chronic Complications | 1.38 (1.35-1.40) | 1.42 (1.37-1.48) | 1.08 (1.05-1.11) | 0.99 (0.93-1.07) | 1.04 (0.98-1.11) | 0.95 (0.82-1.09) |
| Diabetes with Chronic Complications | 1.36 (1.34-1.38) | 1.50 (1.44-1.56) | 1.20 (1.17-1.23) | 1.17 (1.11-1.24) | 1.10 (1.04-1.15) | 1.17 (1.04-1.31) |
| Hemiplegia or paraplegia | 1.90 (1.83-1.97) | 1.55 (1.42-1.70) | 0.94 (0.90-0.99) | 0.89 (0.79-1.01) | 0.88 (0.80-0.97) | 0.89 (0.70-1.12) |
| Renal Disease | 1.81 (1.78-1.83) | 1.63 (1.58-1.69) | 1.99 (1.94-2.03) | 1.90 (1.80-2.01) | 0.88 (0.84-0.92) | 0.90 (0.81-1.00) |
| Cancer |  |  |  |  |  |  |
| No History of Malignant Cancer | Reference | Reference | Reference | Reference | Reference | Reference |
| Any Malignancy Except Neoplasm of skin | 0.97 (0.96-0.99) | 1.12 (1.08-1.17) | 0.91 (0.88-0.93) | 0.97 (0.91-1.03) | 0.80 (0.75-0.84) | 0.83 (0.73-0.94) |
| Metastatic Solid Tumor | 1.87 (1.81-1.93) | 2.16 (2.00-2.33) | 0.88 (0.84-0.92) | 0.94 (0.83-1.05) | 0.72 (0.64-0.80) | 0.72 (0.56-0.94) |
| HIV | 1.04 (0.98-1.10) | 0.84 (0.67-1.04) | 0.96 (0.88-1.04) | 0.86 (0.61-1.20) | 0.94 (0.77-1.15) | 0.74 (0.33-1.65) |
| Obesity | 1.02 (1.01-1.03) | 0.95 (0.93-0.98) | 1.22 (1.20-1.25) | 1.27 (1.21-1.33) | 1.12 (1.08-1.17) | 1.24 (1.13-1.37) |
| Hypertension | 0.84 (0.83-0.85) | 0.87 (0.85-0.90) | 1.10 (1.08-1.13) | 1.17 (1.11-1.24) | 1.04 (0.99-1.09) | 1.13 (1.01-1.25) |
| Former or Current Tobacco User | 1.54 (1.51-1.57) | 1.67 (1.61-1.74) | 1.12 (1.09-1.15) | 1.07 (1.00-1.14) | 1.29 (1.21-1.36) | 1.23 (1.09-1.40) |
| History of Substance Abuse Disorder | 2.65 (2.60-2.71) | 2.33 (2.21-2.45) | 0.97 (0.94-1.00) | 1.04 (0.95-1.13) | 0.90 (0.84-0.97) | 1.00 (0.83-1.19) |
| Vaccination Status Prior to SARS-CoV-2 Infection |  |  |  |  |  |  |
| No Documented COVID-19 Vaccination | Reference | Reference | Reference | Reference | Reference | Reference |
| Primary Vaccination Series | 0.52 (0.51-0.53) | 0.50 (0.49-0.52) | 0.77 (0.75-0.79) | 0.70 (0.66-0.75) | 0.71 (0.66-0.75) | 0.76 (0.67-0.86) |
| Primary+ Vaccination Series | 0.42 (0.41-0.43) | 0.42 (0.40-0.43) | 0.70 (0.67-0.72) | 0.67 (0.62-0.73) | 0.67 (0.63-0.72) | 0.72 (0.62-0.84) |
| Census Region |  |  |  |  |  |  |
| Midwest | Reference | Reference | Reference | Reference | Reference | Reference |
| Northeast | 1.24 (1.22-1.25) | 0.56 (0.54-0.58) | 1.73 (1.69-1.78) | 1.85 (1.71-2.01) | 2.35 (2.24-2.48) | 2.28 (1.97-2.64) |
| South | 1.23 (1.22-1.24) | 1.89 (1.84-1.93) | 1.88 (1.84-1.92) | 2.67 (2.55-2.80) | 2.41 (2.30-2.52) | 3.00 (2.74-3.29) |
| West | 0.84 (0.83-0.85) | 1.70 (1.62-1.79) | 1.65 (1.60-1.69) | 3.48 (3.19-3.80) | 1.57 (1.48-1.67) | 2.72 (2.24-3.31) |
| Nirmatrelvir/ritonavir | 0.07 (0.06-0.07) | 0.05 (0.04-0.06) | 0.34 (0.28-0.42) | 0.51 (0.32-0.83) | 0.61 (0.44-0.83) | 0.71 (0.32-1.58) |
| Molnupiravir | 0.07 (0.06-0.08) | 0.04 (0.03-0.05) | 0.64 (0.49-0.83) | 0.58 (0.31-1.08) | 0.77 (0.47-1.26) | 0.43 (0.11-1.71) |
| Monoclonal Therapies | 0.11 (0.11-0.12) | 0.12 (0.11-0.13) | 0.67 (0.62-0.72) | 0.69 (0.60-0.81) | 0.54 (0.46-0.64) | 0.68 (0.49-0.92) |
| Remdesivir |  |  | 0.47 (0.46-0.49) | 0.59 (0.56-0.64) | 0.32 (0.31-0.35) | 0.43 (0.38-0.50) |
| Anticoagulants |  |  | 0.20 (0.20-0.21) | 0.23 (0.22-0.25) | 0.18 (0.17-0.19) | 0.18 (0.16-0.20) |
| Tocilizumab |  |  |  |  |  |  |
| Dexamethasone |  |  |  |  |  |  |

**B. Invasive Mechanical Ventilation (IMV) or Extracorporeal Membrane Oxygenation (ECMO), Death, and Death after IMV/ECMO**

| **Characteristic** | **IMV/ECMO** | | **Death** | | **Death After IMV/ECMO** | |
| --- | --- | --- | --- | --- | --- | --- |
|  | **Urban** | **Rural** | **Urban** | **Rural** | **Urban** | **Rural** |
|  | **Adjusted Hazard Ratio**  **(95% CI)** | **Adjusted Hazard Ratio**  **(95% CI)** | **Adjusted Hazard Ratio (95% CI)** | **Adjusted Hazard Ratio (95% CI)** | **Adjusted Hazard Ratio (95% CI)** | **Adjusted Hazard Ratio (95% CI)** |
| Sex |  |  |  |  |  |  |
| Female | Reference | Reference | Reference | Reference | Reference | Reference |
| Male | 1.63 (1.58-1.68) | 1.53 (1.44-1.62) | 1.38 (1.35-1.42) | 1.26 (1.20-1.33) | 1.08 (1.04-1.12) | 0.94 (0.87-1.01) |
| Age at COVID-19 Diagnosis | 1.01 (1.00-1.01) | 1.00 (1.00-1.00) | 1.04 (1.04-1.04) | 1.03 (1.03-1.04) | 1.03 (1.03-1.03) | 1.03 (1.03-1.03) |
| Race/Ethnicity |  |  |  |  |  |  |
| White Non-Hispanic | Reference | Reference | Reference | Reference | Reference | Reference |
| Black or African American Non-Hispanic | 1.06 (1.02-1.10) | 1.20 (1.08-1.33) | 0.93 (0.89-0.96) | 1.01 (0.91-1.13) | 1.02 (0.97-1.07) | 1.04 (0.91-1.18) |
| Hispanic or Latino Any Race | 1.15 (1.10-1.21) | 1.28 (1.13-1.46) | 1.12 (1.07-1.17) | 1.03 (0.89-1.19) | 1.19 (1.11-1.26) | 1.04 (0.88-1.24) |
| Other | 1.29 (1.20-1.39) | 1.16 (0.90-1.50) | 1.19 (1.11-1.27) | 1.09 (0.85-1.40) | 1.20 (1.09-1.32) | 0.90 (0.61-1.31) |
| Missing/Unknown | 1.33 (1.25-1.42) | 1.75 (1.52-2.02) | 1.22 (1.15-1.30) | 1.86 (1.61-2.15) | 1.17 (1.08-1.28) | 1.44 (1.22-1.71) |
| Myocardial infarction (MI) | 1.02 (0.96-1.08) | 1.03 (0.91-1.17) | 1.05 (1.00-1.09) | 1.06 (0.97-1.17) | 1.03 (0.97-1.11) | 1.09 (0.95-1.25) |
| Congestive heart failure (CHF) | 0.97 (0.93-1.02) | 0.95 (0.85-1.05) | 1.11 (1.07-1.15) | 1.10 (1.02-1.20) | 0.88 (0.83-0.93) | 0.88 (0.78-0.99) |
| Peripheral vascular disease (PVD) | 0.94 (0.88-0.99) | 0.94 (0.82-1.07) | 1.05 (1.00-1.10) | 0.95 (0.86-1.04) | 1.07 (1.00-1.14) | 1.00 (0.86-1.16) |
| Cerebrovascular disease (CVD) | 0.95 (0.89-1.01) | 0.96 (0.84-1.10) | 0.92 (0.88-0.96) | 0.98 (0.88-1.08) | 0.98 (0.92-1.06) | 1.04 (0.90-1.21) |
| Dementia | 0.57 (0.52-0.62) | 0.44 (0.34-0.57) | 1.15 (1.10-1.21) | 1.16 (1.04-1.31) | 1.14 (1.05-1.24) | 1.10 (0.89-1.35) |
| Chronic pulmonary disease (CPD) | 1.07 (1.03-1.11) | 1.08 (1.0-1.18) | 1.05 (1.01-1.08) | 1.05 (0.98-1.13) | 0.89 (0.85-0.94) | 0.87 (0.79-0.97) |
| Rheumatologic disease (RD) | 1.12 (1.07-1.19) | 1.02 (0.91-1.15) | 1.00 (0.96-1.05) | 1.07 (0.98-1.17) | 1.03 (0.96-1.09) | 1.01 (0.89-1.15) |
| Peptic ulcer disease (PUD) | 1.03 (0.94-1.13) | 0.91 (0.73-1.15) | 0.98 (0.90-1.06) | 0.86 (0.72-1.04) | 1.01 (0.90-1.13) | 0.80 (0.61-1.05) |
| Liver Disease |  |  |  |  |  |  |
| No History of Liver Disease | Reference | Reference | Reference | Reference | Reference | Reference |
| Mild Liver Disease | 1.11 (1.05-1.18) | 0.89 (0.77-1.03) | 1.02 (0.96-1.08) | 1.00 (0.88-1.14) | 0.98 (0.90-1.06) | 0.97 (0.81-1.17) |
| Moderate to Severe Liver Disease | 1.29 (1.19-1.40) | 1.22 (1.02-1.46) | 1.67 (1.55-1.79) | 1.64 (1.41-1.91) | 1.37 (1.24-1.52) | 1.37 (1.10-1.71) |
| Diabetes Mellitus |  |  |  |  |  |  |
| No History of Diabetes | Reference | Reference | Reference | Reference | Reference | Reference |
| Diabetes without Chronic Complications | 1.06 (1.00-1.12) | 0.96 (0.85-1.07) | 1.04 (0.99-1.09) | 1.01 (0.92-1.11) | 1.01 (0.95-1.09) | 0.94 (0.82-1.07) |
| Diabetes with Chronic Complications | 1.20 (1.14-1.26) | 1.08 (0.97-1.20) | 1.08 (1.04-1.13) | 1.10 (1.01-1.20) | 1.03 (0.97-1.09) | 0.93 (0.82-1.05) |
| Hemiplegia or paraplegia | 0.97 (0.88-1.07) | 0.88 (0.69-1.11) | 1.08 (1.00-1.17) | 1.05 (0.88-1.26) | 1.00 (0.89-1.12) | 1.05 (0.81-1.37) |
| Renal Disease | 0.91 (0.87-0.95) | 0.91 (0.83-1.00) | 1.16 (1.12-1.21) | 1.16 (1.08-1.25) | 1.09 (1.03-1.15) | 1.10 (0.99-1.23) |
| Cancer |  |  |  |  |  |  |
| No History of Malignant Cancer | Reference | Reference | Reference | Reference | Reference | Reference |
| Any Malignancy Except Neoplasm of skin | 0.83 (0.79-0.88) | 0.85 (0.76-0.95) | 1.09 (1.05-1.14) | 1.14 (1.05-1.24) | 1.14 (1.07-1.21) | 1.10 (0.97-1.24) |
| Metastatic Solid Tumor | 0.81 (0.74-0.90) | 0.70 (0.56-0.87) | 2.07 (1.95-2.19) | 1.87 (1.64-2.13) | 1.65 (1.48-1.83) | 1.75 (1.42-2.17) |
| HIV | 0.91 (0.77-1.08) | 0.45 (0.20-1.00) | 1.00 (0.84-1.19) | 1.19 (0.70-2.02) | 1.14 (0.90-1.44) | 2.04 (1.01-4.11) |
| Obesity | 1.22 (1.18-1.26) | 1.26 (1.17-1.35) | 1.03 (1.00-1.06) | 0.90 (0.84-0.96) | 0.88 (0.84-0.92) | 0.84 (0.77-0.92) |
| Hypertension | 0.87 (0.83-0.90) | 0.86 (0.79-0.93) | 0.84 (0.81-0.87) | 0.87 (0.81-0.93) | 0.90 (0.85-0.95) | 0.94 (0.85-1.04) |
| Former or Current Tobacco User | 0.99 (0.94-1.04) | 0.89 (0.81-0.99) | 0.95 (0.90-1.00) | 0.94 (0.86-1.04) | 0.91 (0.85-0.97) | 0.90 (0.79-1.02) |
| History of Substance Abuse Disorder | 1.05 (1.00-1.12) | 1.11 (0.98-1.25) | 0.98 (0.92-1.04) | 0.94 (0.82-1.08) | 1.02 (0.94-1.10) | 1.05 (0.88-1.25) |
| Vaccination Status Prior to SARS-CoV-2 Infection |  |  |  |  |  |  |
| No Documented COVID-19 Vaccination | Reference | Reference | Reference | Reference | Reference | Reference |
| Primary Vaccination Series | 0.60 (0.57-0.64) | 0.50 (0.44-0.57) | 0.70 (0.67-0.73) | 0.64 (0.58-0.70) | 0.83 (0.78-0.90) | 0.81 (0.70-0.94) |
| Primary+ Vaccination Series | 0.51 (0.47-0.55) | 0.47 (0.40-0.56) | 0.50 (0.47-0.53) | 0.45 (0.40-0.51) | 0.60 (0.55-0.67) | 0.43 (0.34-0.54) |
| Census Region |  |  |  |  |  |  |
| Midwest | Reference | Reference | Reference | Reference | Reference | Reference |
| Northeast | 1.31 (1.25-1.38) | 2.21 (1.95-2.50) | 0.86 (0.82-0.89) | 0.95 (0.85-1.07) | 1.16 (1.09-1.23) | 0.78 (0.66-0.92) |
| South | 2.43 (2.33-2.52) | 3.01 (2.81-3.24) | 1.07 (1.04-1.11) | 1.33 (1.25-1.41) | 1.11 (1.06-1.17) | 1.10 (1.01-1.19) |
| West | 2.44 (2.33-2.56) | 5.46 (4.90-6.08) | 1.01 (0.97-1.06) | 1.83 (1.64-2.05) | 0.86 (0.81-0.92) | 1.07 (0.93-1.22) |
| Nirmatrelvir/ritonavir | 0.18 (0.11-0.29) | 0.07 (0.01-0.50) | 0.36 (0.26-0.49) | 0.51 (0.26-0.98) | 0.58 (0.33-0.99) | 0.29 (0.04-2.08) |
| Molnupiravir | 0.71 (0.42-1.20) | 0.79 (0.30-2.11) | 0.71 (0.46-1.09) | 0.54 (0.20-1.44) | 1.04 (0.60-1.79) | 1.48 (0.37-5.96) |
| Monoclonal Therapies | 0.51 (0.44-0.58) | 0.57 (0.44-0.73) | 0.57 (0.51-0.64) | 0.56 (0.46-0.69) | 0.70 (0.60-0.80) | 0.59 (0.45-0.78) |
| Remdesivir | 0.94 (0.90-0.97) | 0.86 (0.79-0.94) | 1.35 (1.31-1.39) | 1.24 (1.17-1.33) | 0.81 (0.78-0.84) | 0.72 (0.67-0.78) |
| Anticoagulants | 0.35 (0.34-0.37) | 0.34 (0.32-0.37) | 0.79 (0.77-0.81) | 0.83 (0.78-0.88) | 0.55 (0.53-0.58) | 0.49 (0.45-0.53) |
| Tocilizumab |  |  |  |  | 1.37 (1.29-1.46) | 1.29 (1.15-1.45) |
| Dexamethasone |  |  |  |  | 0.91 (0.87-0.95) | 1.11 (1.02-1.21) |

**eTable S-7. Baseline Characteristics of Patients with SARS-CoV-2 Infection by Rural-Dwelling Status after Propensity-Score Matching**

| **Charactersitic** | **SARS-CoV-2 Infected** | | | | **Hospitalized within 14 Days of SARS-CoV-2 Infection** | | | |
| --- | --- | --- | --- | --- | --- | --- | --- | --- |
|  | **Overall,**  **N = 990,106** | **Urban,**  **N = 495,053** | **Urban-Adjacent Rural,**  **N = 402,118** | **Nonurban-Adjacent Rural,**  **N = 92,935** | **Overall,**  **N = 92,850** | **Urban,**  **N = 42,676** | **Urban-Adjacent Rural,**  **N = 39,967** | **Nonurban-Adjacent Rural,  N = 10,207** |
| Sex |  |  |  |  |  |  |  |  |
| Female | 549,988 (56%) | 274,994 (56%) | 224,203 (56%) | 50,791 (55%) | 45,705 (49%) | 21,053 (49%) | 19,781 (49%) | 4,871 (48%) |
| Male | 440,118 (44%) | 220,059 (44%) | 177,915 (44%) | 42,144 (45%) | 47,145 (51%) | 21,623 (51%) | 20,186 (51%) | 5,336 (52%) |
| Age at COVID-19 Diagnosis | 49 (34, 64) | 49 (33, 63) | 49 (34, 64) | 52 (36, 66) | 64 (50, 75) | 64 (50, 76) | 64 (50, 75) | 65 (52, 75) |
| Race/Ethnicity |  |  |  |  |  |  |  |  |
| White Non-Hispanic | 853,446 (86%) | 426,723 (86%) | 342,886 (85%) | 83,837 (90%) | 79,730 (86%) | 36,697 (86%) | 33,966 (85%) | 9,067 (89%) |
| Black or African American Non-Hispanic | 45,886 (4.6%) | 22,943 (4.6%) | 20,085 (5.0%) | 2,858 (3.1%) | 6,566 (7.1%) | 3,078 (7.2%) | 2,940 (7.4%) | 548 (5.4%) |
| Hispanic or Latino Any Race | 40,632 (4.1%) | 20,316 (4.1%) | 17,808 (4.4%) | 2,508 (2.7%) | 3,784 (4.1%) | 1,717 (4.0%) | 1,762 (4.4%) | 305 (3.0%) |
| Other | 28,778 (2.9%) | 14,389 (2.9%) | 12,359 (3.1%) | 2,030 (2.2%) | 1,138 (1.2%) | 552 (1.3%) | 514 (1.3%) | 72 (0.7%) |
| Missing/Unknown | 21,364 (2.2%) | 10,682 (2.2%) | 8,980 (2.2%) | 1,702 (1.8%) | 1,632 (1.8%) | 632 (1.5%) | 785 (2.0%) | 215 (2.1%) |
| Vaccination Status Prior to SARS-CoV-2 Infection |  |  |  |  |  |  |  |  |
| No Documented COVID-19 Vaccination | 730,507 (74%) | 348,834 (70%) | 306,689 (76%) | 74,984 (81%) | 75,405 (81%) | 34,008 (80%) | 32,678 (82%) | 8,719 (85%) |
| Primary Vaccination Series | 162,291 (16%) | 89,259 (18%) | 61,148 (15%) | 11,884 (13%) | 10,629 (11%) | 5,270 (12%) | 4,439 (11%) | 920 (9.0%) |
| Primary+ Vaccination Series | 97,308 (9.8%) | 56,960 (12%) | 34,281 (8.5%) | 6,067 (6.5%) | 6,816 (7.3%) | 3,398 (8.0%) | 2,850 (7.1%) | 568 (5.6%) |
| Variant Period |  |  |  |  |  |  |  |  |
| Pre-Delta (January 1, 2021 - June 14, 2021) | 158,346 (16%) | 79,173 (16%) | 64,063 (16%) | 15,110 (16%) | 17,316 (19%) | 8,264 (19%) | 7,274 (18%) | 1,778 (17%) |
| Delta (June 15, 2021 - December 21, 2021) | 332,056 (34%) | 166,028 (34%) | 133,934 (33%) | 32,094 (35%) | 34,608 (37%) | 16,279 (38%) | 14,575 (36%) | 3,754 (37%) |
| Omicron (>= December 22 ,2021) | 499,704 (50%) | 249,852 (50%) | 204,121 (51%) | 45,731 (49%) | 40,926 (44%) | 18,133 (42%) | 18,118 (45%) | 4,675 (46%) |
| Comorbidities Before SARS-CoV-2 Infection |  |  |  |  |  |  |  |  |
| Charlson Comorbidity Index, Median (IQR) | 0 (0, 1) | 0 (0, 1) | 0 (0, 1) | 0 (0, 1) | 2 (0, 4) | 1 (0, 4) | 2 (0, 4) | 2 (0, 4) |
| Myocardial infarction | 22,897 (2.3%) | 10,690 (2.2%) | 9,596 (2.4%) | 2,611 (2.8%) | 6,489 (7.0%) | 2,968 (7.0%) | 2,751 (6.9%) | 770 (7.5%) |
| Congestive heart failure | 41,770 (4.2%) | 19,498 (3.9%) | 17,583 (4.4%) | 4,689 (5.0%) | 12,888 (14%) | 5,949 (14%) | 5,472 (14%) | 1,467 (14%) |
| Peripheral vascular disease | 25,671 (2.6%) | 12,330 (2.5%) | 10,553 (2.6%) | 2,788 (3.0%) | 7,060 (7.6%) | 3,332 (7.8%) | 2,954 (7.4%) | 774 (7.6%) |
| Cerebrovascular disease | 27,553 (2.8%) | 13,636 (2.8%) | 10,957 (2.7%) | 2,960 (3.2%) | 6,903 (7.4%) | 3,373 (7.9%) | 2,773 (6.9%) | 757 (7.4%) |
| Dementia | 12,400 (1.3%) | 6,383 (1.3%) | 4,923 (1.2%) | 1,094 (1.2%) | 3,577 (3.9%) | 1,872 (4.4%) | 1,390 (3.5%) | 315 (3.1%) |
| Chronic pulmonary disease | 104,012 (11%) | 51,534 (10%) | 41,962 (10%) | 10,516 (11%) | 19,606 (21%) | 9,239 (22%) | 8,285 (21%) | 2,082 (20%) |
| Rheumatologic disease | 39,835 (4.0%) | 19,521 (3.9%) | 16,073 (4.0%) | 4,241 (4.6%) | 7,198 (7.8%) | 3,321 (7.8%) | 3,050 (7.6%) | 827 (8.1%) |
| Peptic ulcer disease | 8,670 (0.9%) | 4,255 (0.9%) | 3,489 (0.9%) | 926 (1.0%) | 1,804 (1.9%) | 851 (2.0%) | 749 (1.9%) | 204 (2.0%) |
| Mild Liver Disease | 29,993 (3.0%) | 15,083 (3.0%) | 11,882 (3.0%) | 3,028 (3.3%) | 4,544 (4.9%) | 2,241 (5.3%) | 1,804 (4.5%) | 499 (4.9%) |
| Moderate to Severe Liver Disease | 5,759 (0.6%) | 2,711 (0.5%) | 2,457 (0.6%) | 591 (0.6%) | 1,868 (2.0%) | 858 (2.0%) | 820 (2.1%) | 190 (1.9%) |
| Diabetes without Chronic Complications | 51,957 (5.2%) | 24,201 (4.9%) | 22,577 (5.6%) | 5,179 (5.6%) | 8,114 (8.7%) | 3,603 (8.4%) | 3,628 (9.1%) | 883 (8.7%) |
| Diabetes with Chronic Complications | 51,593 (5.2%) | 24,436 (4.9%) | 21,716 (5.4%) | 5,441 (5.9%) | 12,790 (14%) | 5,833 (14%) | 5,553 (14%) | 1,404 (14%) |
| Hemiplegia or paraplegia | 5,718 (0.6%) | 2,665 (0.5%) | 2,446 (0.6%) | 607 (0.7%) | 1,923 (2.1%) | 919 (2.2%) | 779 (1.9%) | 225 (2.2%) |
| Renal disease | 70,205 (7.1%) | 33,359 (6.7%) | 29,564 (7.4%) | 7,282 (7.8%) | 18,745 (20%) | 8,631 (20%) | 8,035 (20%) | 2,079 (20%) |
| Any Malignancy Except Neoplasm of skin | 51,754 (5.2%) | 26,533 (5.4%) | 19,669 (4.9%) | 5,552 (6.0%) | 8,984 (9.7%) | 4,174 (9.8%) | 3,786 (9.5%) | 1,024 (10%) |
| Metastatic Solid Tumor | 7,152 (0.7%) | 3,235 (0.7%) | 3,079 (0.8%) | 838 (0.9%) | 1,978 (2.1%) | 805 (1.9%) | 928 (2.3%) | 245 (2.4%) |
| HIV | 1,689 (0.2%) | 924 (0.2%) | 654 (0.2%) | 111 (0.1%) | 264 (0.3%) | 146 (0.3%) | <120* | <20* |
| Hypertension | 239,507 (24%) | 120,453 (24%) | 95,266 (24%) | 23,788 (26%) | 38,960 (42%) | 18,665 (44%) | 16,203 (41%) | 4,092 (40%) |
| Obesity | 233,335 (24%) | 117,016 (24%) | 92,711 (23%) | 23,608 (25%) | 28,404 (31%) | 13,362 (31%) | 11,847 (30%) | 3,195 (31%) |
| Former or Current Tobacco User | 42,729 (4.3%) | 18,736 (3.8%) | 19,588 (4.9%) | 4,405 (4.7%) | 8,686 (9.4%) | 3,906 (9.2%) | 3,810 (9.5%) | 970 (9.5%) |
| History of Substance Abuse Disorder | 23,795 (2.4%) | 12,528 (2.5%) | 9,294 (2.3%) | 1,973 (2.1%) | 5,470 (5.9%) | 2,895 (6.8%) | 2,072 (5.2%) | 503 (4.9%) |
| Census Region |  |  |  |  |  |  |  |  |
| Midwest | 646,095 (65%) | 314,226 (63%) | 272,810 (68%) | 59,059 (64%) | 48,488 (52%) | 22,035 (52%) | 21,441 (54%) | 5,012 (49%) |
| Northeast | 58,098 (5.9%) | 28,805 (5.8%) | 17,953 (4.5%) | 11,340 (12%) | 6,404 (6.9%) | 3,626 (8.5%) | 1,711 (4.3%) | 1,067 (10%) |
| South | 247,364 (25%) | 129,422 (26%) | 98,106 (24%) | 19,836 (21%) | 33,580 (36%) | 14,875 (35%) | 15,112 (38%) | 3,593 (35%) |
| West | 38,549 (3.9%) | 22,600 (4.6%) | 13,249 (3.3%) | 2,700 (2.9%) | 4,378 (4.7%) | 2,140 (5.0%) | 1,703 (4.3%) | 535 (5.2%) |
| COVID-19 Hospitalization (-3/+14 Days within Diagnosis) | 92,850 (9.4%) | 42,676 (8.6%) | 39,967 (9.9%) | 10,207 (11%) | N/A | N/A | N/A | N/A |
| Adverse Inpatient Events within 45 Days |  |  |  |  |  |  |  |  |
| AKI/Dialysis | N/A | N/A | N/A | N/A | 18,014 (19%) | 7,917 (19%) | 7,946 (20%) | 2,151 (21%) |
| MACE | N/A | N/A | N/A | N/A | 4,612 (5.0%) | 1,951 (4.6%) | 2,087 (5.2%) | 574 (5.6%) |
| ECMO/Invasive Mechanical Ventilation | N/A | N/A | N/A | N/A | 7,343 (7.9%) | 2,764 (6.5%) | 3,517 (8.8%) | 1,062 (10%) |
| Inpatient Death | N/A | N/A | N/A | N/A | 9,874 (11%) | 3,990 (9.3%) | 4,613 (12%) | 1,271 (12%) |
| Number of Visits Before COVID-19, Median (IQR) | 13 (3, 39) | 14 (3, 40) | 12 (3, 37) | 12 (2, 39) | 16 (3, 51) | 19 (4, 55) | 14 (2, 47) | 13 (1, 45) |
| Observation Period Before COVID-19, Median (IQR) | 935 (181, 1,317) | 979 (241, 1,327) | 884 (131, 1,306) | 869 (97, 1,308) | 945 (64, 1,317) | 1,018 (185, 1,332) | 870 (18, 1,305) | 791 (0, 1,288) |
| Number of Visits After COVID-19, Median (IQR) | 5 (1, 15) | 5 (1, 15) | 5 (1, 15) | 5 (1, 15) | 7 (2, 22) | 8 (2, 24) | 6 (1, 20) | 6 (1, 20) |
| Observation Period After COVID-19, Median (IQR) | 176 (0, 379) | 184 (0, 383) | 168 (0, 375) | 164 (0, 376) | 172 (1, 399) | 201 (8, 414) | 150 (0, 386) | 142 (0, 386) |
| * Obfuscated to comply with N3C privacy policies, which require censoring small cell counts (<20) and obfuscating adjacent cells to prevent back-calculating for all summary statistics. | | | | | | | | |

**eTable S-8. Multivariable Regression for Adverse Acute COVID-19 Events by Rurality Across All Time Periods and within COVID-19 Epochs after Propensity-Score Matching**

| **Charactersitic** | **All Medications Combined** | | | | | |
| --- | --- | --- | --- | --- | --- | --- |
|  | **Hospitalization** | **AKI/Dialysis** | **MACE** | **IMV/ECMO** | **Death** | **Death After IMV/ECMO** |
|  | **Adjusted Odds Ratio (95% CI)** | **Adjusted Hazard Ratio (95% CI)** | **Adjusted Hazard Ratio (95% CI)** | **Adjusted Hazard Ratio (95% CI)** | **Adjusted Hazard Ratio (95% CI)** | **Adjusted Hazard Ratio (95% CI)** |
| Sex |  |  |  |  |  |  |
| Female | Reference | Reference | Reference | Reference | Reference | Reference |
| Male | 1.24(1.22, 1.26) | 1.41(1.37, 1.46) | 1.46(1.38, 1.55) | 1.57(1.50, 1.65) | 1.31(1.26, 1.37) | 0.98(0.92, 1.03) |
| Age at COVID-19 Diagnosis | 1.04(1.04, 1.04) | 1.02(1.02, 1.02) | 1.03(1.02, 1.03) | 1.00(1.00, 1.00) | 1.03(1.03, 1.04) | 1.03(1.03, 1.03) |
| Race/Ethnicity |  |  |  |  |  |  |
| White Non-Hispanic | Reference | Reference | Reference | Reference | Reference | Reference |
| Black or African American Non-Hispanic | 1.30(1.26, 1.34) | 1.72(1.64, 1.81) | 1.38(1.25, 1.53) | 1.20(1.10, 1.30) | 0.97(0.89, 1.06) | 1.02(0.92, 1.13) |
| Hispanic or Latino Any Race | 1.54(1.48, 1.60) | 1.24(1.15, 1.35) | 1.11(0.93, 1.33) | 1.34(1.21, 1.48) | 1.03(0.92, 1.16) | 1.05(0.91, 1.20) |
| Other | 0.45(0.43, 0.48) | 1.15(1.00, 1.32) | 1.04(0.78, 1.37) | 1.20(0.99, 1.47) | 1.12(0.92, 1.35) | 1.02(0.78, 1.35) |
| Missing/Unknown | 0.86(0.81, 0.90) | 1.55(1.40, 1.72) | 1.89(1.58, 2.26) | 1.89(1.68, 2.14) | 1.84(1.63, 2.08) | 1.37(1.18, 1.59) |
| Myocardial infarction (MI) | 1.11(1.07, 1.15) | 0.94(0.89, 0.98) | 2.54(2.33, 2.76) | 0.98(0.89, 1.08) | 1.07(0.99, 1.15) | 1.10(0.99, 1.23) |
| Congestive heart failure (CHF) | 1.53(1.48, 1.57) | 1.01(0.97, 1.05) | 1.07(0.98, 1.16) | 0.93(0.86, 1.01) | 1.09(1.02, 1.16) | 0.86(0.78, 0.94) |
| Peripheral vascular disease (PVD) | 1.12(1.08, 1.16) | 0.98(0.93, 1.03) | 1.03(0.94, 1.14) | 0.93(0.84, 1.03) | 0.99(0.92, 1.07) | 1.03(0.92, 1.16) |
| Cerebrovascular disease (CVD) | 1.01(0.98, 1.05) | 0.94(0.89, 0.99) | 1.18(1.07, 1.30) | 0.95(0.85, 1.06) | 0.94(0.87, 1.02) | 0.99(0.88, 1.11) |
| Dementia | 1.21(1.16, 1.27) | 0.87(0.81, 0.93) | 0.63(0.55, 0.73) | 0.45(0.38, 0.54) | 1.22(1.12, 1.32) | 1.20(1.03, 1.39) |
| Chronic pulmonary disease (CPD) | 1.32(1.29, 1.35) | 0.97(0.93, 1.00) | 0.91(0.84, 0.98) | 1.08(1.01, 1.15) | 1.09(1.04, 1.15) | 0.89(0.82, 0.96) |
| Rheumatologic disease (RD) | 1.04(1.01, 1.08) | 1.12(1.06, 1.17) | 0.94(0.85, 1.04) | 1.03(0.94, 1.13) | 1.03(0.96, 1.11) | 0.98(0.89, 1.08) |
| Peptic ulcer disease (PUD) | 1.12(1.05, 1.19) | 1.01(0.93, 1.10) | 0.83(0.68, 1.02) | 0.95(0.80, 1.13) | 0.87(0.76, 1.00) | 0.80(0.65, 0.98) |
| Liver Disease |  |  |  |  |  |  |
| No History of Liver Disease | Reference | Reference | Reference | Reference | Reference | Reference |
| Mild Liver Disease | 1.06(1.02, 1.10) | 0.97(0.91, 1.04) | 0.81(0.70, 0.93) | 0.91(0.81, 1.01) | 0.99(0.89, 1.09) | 0.97(0.84, 1.11) |
| Moderate to Severe Liver Disease | 1.70(1.60, 1.82) | 1.19(1.10, 1.29) | 0.68(0.55, 0.84) | 1.19(1.03, 1.37) | 1.68(1.49, 1.89) | 1.51(1.28, 1.79) |
| Diabetes Mellitus |  |  |  |  |  |  |
| No History of Diabetes | Reference | Reference | Reference | Reference | Reference | Reference |
| Diabetes without Chronic Complications | 1.43(1.39, 1.47) | 1.01(0.96, 1.07) | 1.02(0.91, 1.13) | 1.01(0.93, 1.10) | 1.02(0.95, 1.10) | 0.99(0.89, 1.10) |
| Diabetes with Chronic Complications | 1.50(1.45, 1.54) | 1.17(1.12, 1.22) | 1.16(1.06, 1.26) | 1.10(1.02, 1.20) | 1.06(1.00, 1.14) | 0.95(0.86, 1.04) |
| Hemiplegia or paraplegia | 1.68(1.57, 1.79) | 0.89(0.81, 0.98) | 0.95(0.80, 1.12) | 0.90(0.75, 1.08) | 1.11(0.97, 1.27) | 1.25(1.03, 1.53) |
| Renal Disease | 1.64(1.60, 1.68) | 2.04(1.96, 2.12) | 0.90(0.83, 0.98) | 0.90(0.84, 0.97) | 1.18(1.11, 1.24) | 1.11(1.02, 1.21) |
| Cancer |  |  |  |  |  |  |
| No History of Malignant Cancer | Reference | Reference | Reference | Reference | Reference | Reference |
| Any Malignancy Except Neoplasm of skin | 1.07(1.04, 1.10) | 0.97(0.93, 1.02) | 0.83(0.76, 0.92) | 0.85(0.78, 0.93) | 1.12(1.05, 1.19) | 1.11(1.01, 1.22) |
| Metastatic Solid Tumor | 2.05(1.93, 2.17) | 0.92(0.84, 1.01) | 0.71(0.58, 0.87) | 0.68(0.57, 0.82) | 1.94(1.75, 2.14) | 1.88(1.58, 2.23) |
| HIV | 0.97(0.83, 1.12) | 0.88(0.70, 1.10) | 0.61(0.33, 1.14) | 0.73(0.46, 1.15) | 0.96(0.63, 1.46) | 1.75(1.03, 2.96) |
| Obesity | 0.97(0.95, 0.99) | 1.33(1.28, 1.38) | 1.30(1.21, 1.39) | 1.38(1.30, 1.46) | 0.96(0.91, 1.01) | 0.86(0.80, 0.92) |
| Hypertension | 0.92(0.91, 0.94) | 1.17(1.12, 1.21) | 1.11(1.03, 1.20) | 0.87(0.82, 0.93) | 0.87(0.83, 0.92) | 0.92(0.85, 1.0) |
| Former or Current Tobacco User | 1.73(1.68, 1.78) | 1.03(0.98, 1.09) | 1.17(1.07, 1.29) | 0.91(0.84, 0.99) | 0.98(0.90, 1.05) | 0.89(0.81, 0.99) |
| History of Substance Abuse Disorder | 2.45(2.36, 2.54) | 1.01(0.94, 1.07) | 0.97(0.85, 1.11) | 1.06(0.96, 1.17) | 0.91(0.82, 1.01) | 1.09(0.96, 1.25) |
| Vaccination Status Prior to SARS-CoV-2 Infection |  |  |  |  |  |  |
| No Documented COVID-19 Vaccination | Reference | Reference | Reference | Reference | Reference | Reference |
| Primary Vaccination Series | 0.50(0.49, 0.51) | 0.71(0.67, 0.74) | 0.75(0.68, 0.82) | 0.51(0.46, 0.56) | 0.65(0.61, 0.70) | 0.80(0.72, 0.90) |
| Primary+ Vaccination Series | 0.39(0.38, 0.40) | 0.68(0.64, 0.72) | 0.78(0.70, 0.87) | 0.50(0.44, 0.56) | 0.46(0.42, 0.50) | 0.47(0.40, 0.55) |
| Rural-Dwelling Status |  |  |  |  |  |  |
| Urban | Reference | Reference | Reference | Reference | Reference | Reference |
| Urban-Adjacent Rural | 1.10(1.08, 1.12) | 1.08(1.05, 1.12) | 1.16(1.09, 1.24) | 1.37(1.30, 1.44) | 1.25(1.20, 1.31) | 1.17(1.10, 1.24) |
| Nonurban-Adjacent Rural | 1.15(1.12, 1.18) | 1.17(1.11, 1.22) | 1.22(1.11, 1.34) | 1.60(1.49, 1.71) | 1.31(1.23, 1.40) | 1.28(1.17, 1.39) |
| Nirmatrelvir/ritonavir | 0.04(0.04, 0.05) | 0.45(0.32, 0.64) | 1.04(0.65, 1.65) | 0.16(0.06, 0.43) | 0.40(0.23, 0.67) | 0.32(0.08, 1.28) |
| Molnupiravir | 0.04(0.03, 0.06) | 0.63(0.40, 0.99) | 0.83(0.40, 1.75) | 0.99(0.49, 1.98) | 0.71(0.37, 1.37) | 1.35(0.60, 3.02) |
| Monoclonal Therapies | 0.12(0.11, 0.12) | 0.73(0.66, 0.81) | 0.61(0.48, 0.78) | 0.57(0.47, 0.69) | 0.51(0.44, 0.60) | 0.59(0.48, 0.73) |
| Remdesivir |  | 0.59(0.56, 0.62) | 0.44(0.40, 0.49) | 0.91(0.85, 0.97) | 1.26(1.20, 1.32) | 0.73(0.68, 0.77) |
| Anticoagulants |  | 0.24(0.23, 0.25) | 0.20(0.18, 0.21) | 0.36(0.34, 0.38) | 0.81(0.77, 0.84) | 0.50(0.47, 0.54) |
| Tocilizumab |  |  |  |  |  | 1.25(1.14, 1.37) |
| Dexamethasone |  |  |  |  |  | 1.11(1.04, 1.19) |

**eTable S-9. Multivariable Regression for Adverse Acute COVID-19 Events Stratified by Rurality after Propensity-Score Matching**

**A. Hospitalization, Acute Kidney Injury (AKI) or Dialysis, and Major Adverse Cardiovascular Event (MACE)**

| **Characteristic** | **All Medications Combined** | | | | | |
| --- | --- | --- | --- | --- | --- | --- |
|  | **Hospitalization** | | **AKI/Dialysis** | | **MACE** | |
|  | **Urban** | **Rural** | **Urban** | **Rural** | **Urban** | **Rural** |
|  | **Adjusted Odds Ratio (95% CI)** | **Adjusted Odds Ratio (95% CI)** | **Adjusted Hazard Ratio (95% CI)** | **Adjusted Hazard Ratio (95% CI)** | **Adjusted Hazard Ratio (95% CI)** | **Adjusted Hazard Ratio (95% CI)** |
| Sex |  |  |  |  |  |  |
| Female | Reference | Reference | Reference | Reference | Reference | Reference |
| Male | 1.23(1.20, 1.25) | 1.25(1.23, 1.28) | 1.41(1.35, 1.48) | 1.42(1.36, 1.48) | 1.42(1.29, 1.56) | 1.49(1.38, 1.62) |
| Age at COVID-19 Diagnosis | 1.04(1.04, 1.04) | 1.04(1.04, 1.04) | 1.02(1.02, 1.02) | 1.02(1.01, 1.02) | 1.03(1.02, 1.03) | 1.03(1.02, 1.03) |
| Race/Ethnicity |  |  |  |  |  |  |
| White Non-Hispanic | Reference | Reference | Reference | Reference | Reference | Reference |
| Black or African American Non-Hispanic | 1.43(1.36, 1.49) | 1.19(1.14, 1.24) | 1.75(1.63, 1.88) | 1.70(1.60, 1.81) | 1.22(1.03, 1.44) | 1.50(1.32, 1.71) |
| Hispanic or Latino Any Race | 1.57(1.49, 1.66) | 1.52(1.44, 1.60) | 1.23(1.08, 1.40) | 1.23(1.11, 1.37) | 1.17(0.89, 1.55) | 1.06(0.84, 1.34) |
| Other | 0.53(0.48, 0.57) | 0.40(0.36, 0.43) | 1.16(0.94, 1.43) | 1.13(0.94, 1.37) | 0.73(0.45, 1.20) | 1.29(0.92, 1.80) |
| Missing/Unknown | 0.76(0.70, 0.83) | 0.93(0.87, 1.00) | 1.41(1.18, 1.68) | 1.59(1.40, 1.80) | 1.75(1.28, 2.39) | 1.94(1.56, 2.42) |
| Myocardial infarction (MI) | 1.12(1.06, 1.18) | 1.11(1.05, 1.16) | 0.99(0.92, 1.07) | 0.90(0.84, 0.96) | 2.63(2.31, 2.99) | 2.48(2.21, 2.78) |
| Congestive heart failure (CHF) | 1.54(1.48, 1.61) | 1.51(1.45, 1.57) | 0.99(0.93, 1.05) | 1.02(0.96, 1.08) | 1.02(0.90, 1.16) | 1.11(0.99, 1.24) |
| Peripheral vascular disease (PVD) | 1.15(1.09, 1.21) | 1.11(1.06, 1.16) | 0.97(0.90, 1.04) | 0.98(0.92, 1.05) | 0.94(0.81, 1.09) | 1.11(0.97, 1.26) |
| Cerebrovascular disease (CVD) | 1.05(1.00, 1.11) | 0.98(0.93, 1.03) | 0.93(0.86, 1.00) | 0.94(0.87, 1.01) | 1.18(1.02, 1.36) | 1.17(1.03, 1.34) |
| Dementia | 1.33(1.25, 1.42) | 1.09(1.02, 1.16) | 0.81(0.74, 0.89) | 0.92(0.84, 1.02) | 0.75(0.62, 0.91) | 0.52(0.42, 0.65) |
| Chronic pulmonary disease (CPD) | 1.36(1.32, 1.41) | 1.29(1.25, 1.33) | 0.99(0.94, 1.05) | 0.95(0.90, 1.00) | 0.95(0.84, 1.07) | 0.88(0.79, 0.97) |
| Rheumatologic disease (RD) | 1.05(1.01, 1.10) | 1.04(1.00, 1.09) | 1.11(1.03, 1.19) | 1.12(1.05, 1.19) | 0.95(0.82, 1.11) | 0.93(0.82, 1.06) |
| Peptic ulcer disease (PUD) | 1.14(1.04, 1.24) | 1.11(1.03, 1.21) | 1.12(0.99, 1.27) | 0.94(0.83, 1.06) | 1.01(0.76, 1.34) | 0.71(0.53, 0.94) |
| Liver Disease |  |  |  |  |  |  |
| No History of Liver Disease | Reference | Reference | Reference | Reference | Reference | Reference |
| Mild Liver Disease | 1.08(1.03, 1.14) | 1.03(0.98, 1.09) | 0.96(0.88, 1.06) | 0.98(0.90, 1.07) | 0.90(0.73, 1.10) | 0.74(0.61, 0.90) |
| Moderate to Severe Liver Disease | 1.62(1.48, 1.79) | 1.72(1.57, 1.87) | 1.23(1.09, 1.38) | 1.16(1.04, 1.29) | 0.70(0.52, 0.96) | 0.64(0.49, 0.85) |
| Diabetes Mellitus |  |  |  |  |  |  |
| No History of Diabetes | Reference | Reference | Reference | Reference | Reference | Reference |
| Diabetes without Chronic Complications | 1.45(1.39, 1.51) | 1.42(1.36, 1.47) | 1.04(0.96, 1.13) | 1.0(0.93, 1.07) | 1.13(0.96, 1.33) | 0.94(0.82, 1.09) |
| Diabetes with Chronic Complications | 1.49(1.43, 1.55) | 1.50(1.44, 1.56) | 1.17(1.10, 1.25) | 1.17(1.11, 1.24) | 1.18(1.03, 1.35) | 1.14(1.01, 1.29) |
| Hemiplegia or paraplegia | 1.86(1.68, 2.05) | 1.54(1.40, 1.68) | 0.89(0.78, 1.02) | 0.89(0.79, 1.01) | 1.05(0.82, 1.34) | 0.87(0.69, 1.11) |
| Renal Disease | 1.66(1.60, 1.72) | 1.63(1.58, 1.69) | 2.21(2.09, 2.34) | 1.91(1.81, 2.01) | 0.89(0.79, 1.01) | 0.90(0.81, 1.00) |
| Cancer |  |  |  |  |  |  |
| No History of Malignant Cancer | Reference | Reference | Reference | Reference | Reference | Reference |
| Any Malignancy Except Neoplasm of skin | 1.01(0.97, 1.05) | 1.13(1.09, 1.17) | 0.98(0.92, 1.05) | 0.97(0.91, 1.03) | 0.83(0.72, 0.96) | 0.83(0.73, 0.94) |
| Metastatic Solid Tumor | 1.91(1.74, 2.09) | 2.17(2.00, 2.34) | 0.89(0.77, 1.03) | 0.93(0.83, 1.05) | 0.75(0.55, 1.03) | 0.69(0.53, 0.91) |
| HIV | 1.04(0.84, 1.27) | 0.87(0.70, 1.08) | 0.90(0.67, 1.23) | 0.84(0.59, 1.18) | 0.64(0.27, 1.56) | 0.62(0.26, 1.49) |
| Obesity | 0.99(0.96, 1.02) | 0.95(0.92, 0.97) | 1.39(1.32, 1.47) | 1.28(1.22, 1.34) | 1.35(1.21, 1.50) | 1.25(1.14, 1.38) |
| Hypertension | 0.98(0.95, 1.01) | 0.87(0.85, 0.90) | 1.16(1.10, 1.24) | 1.17(1.11, 1.24) | 1.10(0.97, 1.24) | 1.13(1.01, 1.26) |
| Former or Current Tobacco User | 1.79(1.71, 1.87) | 1.67(1.61, 1.74) | 0.98(0.91, 1.06) | 1.07(1.00, 1.14) | 1.06(0.91, 1.24) | 1.26(1.11, 1.42) |
| History of Substance Abuse Disorder | 2.57(2.44, 2.70) | 2.30(2.18, 2.42) | 0.98(0.89, 1.07) | 1.04(0.95, 1.14) | 0.91(0.74, 1.11) | 1.03(0.86, 1.23) |
| Vaccination Status Prior to SARS-CoV-2 Infection |  |  |  |  |  |  |
| No Documented COVID-19 Vaccination | Reference | Reference | Reference | Reference | Reference | Reference |
| Primary Vaccination Series | 0.49(0.47, 0.51) | 0.50(0.49, 0.52) | 0.71(0.66, 0.76) | 0.70(0.66, 0.75) | 0.73(0.63, 0.85) | 0.76(0.67, 0.87) |
| Primary+ Vaccination Series | 0.37(0.36, 0.38) | 0.41(0.40, 0.43) | 0.67(0.61, 0.73) | 0.68(0.62, 0.73) | 0.85(0.72, 1.00) | 0.72(0.62, 0.85) |
| Nirmatrelvir/ritonavir | 0.04(0.03, 0.05) | 0.05(0.04, 0.06) | 0.40(0.24, 0.67) | 0.51(0.32, 0.83) | 1.34(0.76, 2.38) | 0.71(0.32, 1.58) |
| Molnupiravir | 0.05(0.03, 0.07) | 0.04(0.03, 0.05) | 0.69(0.36, 1.33) | 0.58(0.31, 1.08) | 1.44(0.60, 3.47) | 0.43(0.11, 1.71) |
| Monoclonal Therapies | 0.12(0.11, 0.13) | 0.12(0.11, 0.13) | 0.80(0.68, 0.93) | 0.69(0.60, 0.80) | 0.52(0.35, 0.78) | 0.68(0.50, 0.92) |
| Remdesivir |  |  | 0.60(0.56, 0.65) | 0.59(0.55, 0.63) | 0.45(0.38, 0.53) | 0.43(0.38, 0.50) |
| Anticoagulants |  |  | 0.25(0.24, 0.27) | 0.23(0.22, 0.25) | 0.21(0.19, 0.25) | 0.18(0.16, 0.21) |
| Tocilizumab |  |  |  |  |  |  |
| Dexamethasone |  |  |  |  |  |  |

**B. Invasive Mechanical Ventilation (IMV) or Extracorporeal Membrane Oxygenation (ECMO), Death, and Death after IMV/ECMO**

| **Characteristic** | **All Medications Combined** | | | | | |
| --- | --- | --- | --- | --- | --- | --- |
|  | **IMV/ECMO** | | **Death** | | **Death after IMV/ECMO** | |
|  | **Urban** | **Rural** | **Urban** | **Rural** | **Urban** | **Rural** |
|  | **Adjusted Hazard Ratio (95% CI)** | **Adjusted Hazard Ratio (95% CI)** | **Adjusted Hazard Ratio (95% CI)** | **Adjusted Hazard Ratio (95% CI)** | **Adjusted Hazard Ratio (95% CI)** | **Adjusted Hazard Ratio (95% CI)** |
| Sex |  |  |  |  |  |  |
| Female | Reference | Reference | Reference | Reference | Reference | Reference |
| Male | 1.67(1.55, 1.81) | 1.52(1.43, 1.61) | 1.40(1.31, 1.49) | 1.26(1.20, 1.33) | 1.05(0.95, 1.15) | 0.94(0.87, 1.01) |
| Age at COVID-19 Diagnosis | 1.00(1.00, 1.01) | 1.00(1.00, 1.00) | 1.04(1.03, 1.04) | 1.03(1.03, 1.04) | 1.03(1.03, 1.03) | 1.03(1.03, 1.03) |
| Race/Ethnicity |  |  |  |  |  |  |
| White Non-Hispanic | Reference | Reference | Reference | Reference | Reference | Reference |
| Black or African American Non-Hispanic | 1.17(1.02, 1.33) | 1.21(1.09, 1.34) | 0.89(0.78, 1.03) | 1.01(0.91, 1.13) | 0.97(0.82, 1.15) | 1.04(0.91, 1.18) |
| Hispanic or Latino Any Race | 1.38(1.17, 1.64) | 1.29(1.14, 1.46) | 1.00(0.82, 1.23) | 1.03(0.89, 1.20) | 1.04(0.81, 1.33) | 1.04(0.88, 1.24) |
| Other | 1.31(0.97, 1.77) | 1.12(0.86, 1.46) | 1.17(0.88, 1.56) | 1.08(0.84, 1.39) | 1.17(0.79, 1.73) | 0.89(0.60, 1.31) |
| Missing/Unknown | 2.02(1.62, 2.50) | 1.77(1.53, 2.04) | 1.57(1.24, 1.98) | 1.86(1.61, 2.16) | 1.16(0.87, 1.54) | 1.45(1.22, 1.72) |
| Myocardial infarction (MI) | 0.92(0.78, 1.09) | 1.02(0.90, 1.16) | 1.08(0.96, 1.21) | 1.06(0.96, 1.17) | 1.11(0.94, 1.32) | 1.08(0.94, 1.25) |
| Congestive heart failure (CHF) | 0.90(0.79, 1.03) | 0.94(0.85, 1.05) | 1.06(0.96, 1.16) | 1.10(1.01, 1.20) | 0.82(0.71, 0.95) | 0.88(0.78, 1.00) |
| Peripheral vascular disease (PVD) | 0.90(0.76, 1.05) | 0.95(0.83, 1.08) | 1.05(0.94, 1.17) | 0.95(0.86, 1.04) | 1.10(0.92, 1.30) | 1.00(0.86, 1.16) |
| Cerebrovascular disease (CVD) | 0.95(0.81, 1.13) | 0.95(0.82, 1.09) | 0.90(0.80, 1.00) | 0.98(0.88, 1.08) | 0.88(0.73, 1.06) | 1.06(0.91, 1.22) |
| Dementia | 0.47(0.36, 0.61) | 0.43(0.33, 0.55) | 1.28(1.14, 1.44) | 1.16(1.03, 1.30) | 1.36(1.10, 1.68) | 1.07(0.87, 1.32) |
| Chronic pulmonary disease (CPD) | 1.09(0.98, 1.21) | 1.08(0.99, 1.18) | 1.16(1.07, 1.26) | 1.06(0.98, 1.13) | 0.94(0.83, 1.07) | 0.86(0.78, 0.96) |
| Rheumatologic disease (RD) | 1.03(0.89, 1.19) | 1.03(0.91, 1.16) | 0.99(0.89, 1.11) | 1.07(0.98, 1.18) | 0.93(0.79, 1.10) | 1.01(0.89, 1.15) |
| Peptic ulcer disease (PUD) | 1.00(0.76, 1.30) | 0.93(0.74, 1.18) | 0.90(0.73, 1.12) | 0.86(0.71, 1.03) | 0.80(0.57, 1.10) | 0.81(0.62, 1.06) |
| Liver Disease |  |  |  |  |  |  |
| No History of Liver Disease | Reference | Reference | Reference | Reference | Reference | Reference |
| Mild Liver Disease | 0.95(0.80, 1.13) | 0.87(0.75, 1.02) | 0.98(0.84, 1.14) | 1.00(0.88, 1.14) | 0.94(0.75, 1.18) | 0.98(0.81, 1.17) |
| Moderate to Severe Liver Disease | 1.12(0.89, 1.42) | 1.22(1.02, 1.46) | 1.72(1.44, 2.05) | 1.64(1.40, 1.91) | 1.76(1.36, 2.28) | 1.39(1.11, 1.73) |
| Diabetes Mellitus |  |  |  |  |  |  |
| No History of Diabetes | Reference | Reference | Reference | Reference | Reference | Reference |
| Diabetes without Chronic Complications | 1.11(0.96, 1.27) | 0.95(0.85, 1.07) | 1.06(0.95, 1.19) | 1.00(0.91, 1.10) | 1.09(0.93, 1.29) | 0.93(0.81, 1.07) |
| Diabetes with Chronic Complications | 1.14(1.00, 1.30) | 1.08(0.97, 1.20) | 1.03(0.93, 1.14) | 1.10(1.01, 1.19) | 0.97(0.83, 1.12) | 0.93(0.82, 1.06) |
| Hemiplegia or paraplegia | 0.91(0.69, 1.21) | 0.88(0.70, 1.12) | 1.21(1.00, 1.48) | 1.03(0.86, 1.24) | 1.65(1.23, 2.22) | 1.06(0.81, 1.39) |
| Renal Disease | 0.91(0.81, 1.03) | 0.90(0.82, 1.00) | 1.21(1.11, 1.32) | 1.16(1.07, 1.25) | 1.14(1.00, 1.31) | 1.10(0.98, 1.23) |
| Cancer |  |  |  |  |  |  |
| No History of Malignant Cancer | Reference | Reference | Reference | Reference | Reference | Reference |
| Any Malignancy Except Neoplasm of skin | 0.85(0.74, 0.98) | 0.85(0.76, 0.95) | 1.08(0.98, 1.19) | 1.15(1.06, 1.24) | 1.12(0.97, 1.30) | 1.10(0.97, 1.25) |
| Metastatic Solid Tumor | 0.64(0.47, 0.88) | 0.71(0.57, 0.89) | 2.13(1.82, 2.51) | 1.85(1.62, 2.11) | 2.16(1.62, 2.89) | 1.74(1.41, 2.16) |
| HIV | 1.08(0.63, 1.83) | 0.38(0.16, 0.91) | 0.78(0.40, 1.50) | 1.12(0.65, 1.94) | 1.59(0.75, 3.37) | 1.86(0.88, 3.94) |
| Obesity | 1.56(1.42, 1.70) | 1.27(1.18, 1.37) | 1.06(0.99, 1.15) | 0.90(0.84, 0.96) | 0.87(0.78, 0.97) | 0.85(0.77, 0.93) |
| Hypertension | 0.88(0.80, 0.97) | 0.86(0.80, 0.94) | 0.88(0.81, 0.95) | 0.87(0.81, 0.94) | 0.88(0.77, 1.00) | 0.94(0.85, 1.05) |
| Former or Current Tobacco User | 0.94(0.82, 1.07) | 0.90(0.81, 0.99) | 1.04(0.92, 1.17) | 0.94(0.85, 1.04) | 0.89(0.75, 1.05) | 0.89(0.79, 1.02) |
| History of Substance Abuse Disorder | 1.02(0.88, 1.18) | 1.09(0.96, 1.24) | 0.89(0.76, 1.04) | 0.92(0.80, 1.06) | 1.15(0.93, 1.40) | 1.04(0.87, 1.24) |
| Vaccination Status Prior to SARS-CoV-2 Infection |  |  |  |  |  |  |
| No Documented COVID-19 Vaccination | Reference | Reference | Reference | Reference | Reference | Reference |
| Primary Vaccination Series | 0.53(0.45, 0.61) | 0.50(0.44, 0.57) | 0.67(0.60, 0.74) | 0.64(0.58, 0.70) | 0.79(0.67, 0.94) | 0.81(0.70, 0.94) |
| Primary+ Vaccination Series | 0.51(0.42, 0.62) | 0.48(0.40, 0.56) | 0.46(0.40, 0.52) | 0.45(0.40, 0.51) | 0.53(0.42, 0.66) | 0.43(0.34, 0.54) |
| Nirmatrelvir/ritonavir | 0.28(0.09, 0.87) | 0.07(0.01, 0.50) | 0.30(0.12, 0.71) | 0.51(0.26, 0.98) | 0.34(0.05, 2.42) | 0.29(0.04, 2.10) |
| Molnupiravir | 1.31(0.49, 3.52) | 0.79(0.30, 2.12) | 0.96(0.40, 2.31) | 0.54(0.20, 1.44) | 1.27(0.47, 3.41) | 1.48(0.37, 5.96) |
| Monoclonal Therapies | 0.59(0.44, 0.78) | 0.57(0.44, 0.73) | 0.45(0.35, 0.59) | 0.56(0.46, 0.69) | 0.58(0.42, 0.80) | 0.59(0.45, 0.78) |
| Remdesivir | 1.02(0.92, 1.13) | 0.86(0.79, 0.94) | 1.31(1.21, 1.42) | 1.24(1.17, 1.33) | 0.74(0.67, 0.82) | 0.72(0.67, 0.78) |
| Anticoagulants | 0.39(0.35, 0.42) | 0.34(0.32, 0.37) | 0.76(0.71, 0.82) | 0.83(0.78, 0.88) | 0.54(0.49, 0.60) | 0.49(0.45, 0.53) |
| Tocilizumab |  |  |  |  | 1.14(0.98, 1.33) | 1.29(1.15, 1.45) |
| Dexamethasone |  |  |  |  | 1.11(0.98, 1.24) | 1.11(1.01, 1.21) |

**eTable S-10. Multivariable Regression for Adverse Acute COVID-19 Events with COVID-19 Therapies Combined Across All Time Periods after Propensity-Score Matching**

| **Characteristic** | **All Medications Combined** | | | | | |
| --- | --- | --- | --- | --- | --- | --- |
|  | **Hospitalization** | **AKI/Dialysis** | **MACE** | **IMV/ECMO** | **Death** | **Death After IMV/ECMO** |
|  | **Adjusted Odds Ratio**  **(95% CI)** | **Adjusted Hazard Ratio**  **(95% CI)** | **Adjusted Hazard Ratio**  **(95% CI)** | **Adjusted Hazard Ratio**  **(95% CI)** | **Adjusted Hazard Ratio**  **(95% CI)** | **Adjusted Hazard Ratio**  **(95% CI)** |
| Sex |  |  |  |  |  |  |
| Female | Reference | Reference | Reference | Reference | Reference | Reference |
| Male | 1.23 (1.22-1.24) | 1.49 (1.46-1.51) | 1.48 (1.43-1.53) | 1.61 (1.57-1.66) | 1.36 (1.32-1.39) | 1.04 (1.01-1.08) |
| Age at COVID-19 Diagnosis | 1.04 (1.04-1.04) | 1.02 (1.02-1.02) | 1.03 (1.03-1.03) | 1.00 (1.00-1.01) | 1.04 (1.04-1.04) | 1.03 (1.03-1.03) |
| Race/Ethnicity |  |  |  |  |  |  |
| White Non-Hispanic | Reference | Reference | Reference | Reference | Reference | Reference |
| Black or African American Non-Hispanic | 1.69 (1.67-1.71) | 1.65 (1.62-1.68) | 1.33 (1.28-1.39) | 1.08 (1.04-1.12) | 0.94 (0.91-0.97) | 1.02 (0.98-1.07) |
| Hispanic or Latino Any Race | 1.83 (1.80-1.85) | 1.13 (1.10-1.16) | 1.03 (0.97-1.09) | 1.16 (1.11-1.21) | 1.12 (1.07-1.17) | 1.17 (1.11-1.24) |
| Other | 0.97 (0.95-0.99) | 1.23 (1.18-1.28) | 1.30 (1.20-1.41) | 1.27 (1.19-1.36) | 1.18 (1.10-1.25) | 1.18 (1.07-1.29) |
| Missing/Unknown | 0.95 (0.93-0.96) | 1.25 (1.20-1.30) | 1.38 (1.28-1.48) | 1.39 (1.31-1.47) | 1.29 (1.22-1.37) | 1.23 (1.14-1.33) |
| Myocardial infarction (MI) | 1.18 (1.16-1.21) | 0.99 (0.96-1.01) | 2.58 (2.47-2.70) | 1.02 (0.96-1.07) | 1.05 (1.00-1.09) | 1.04 (0.98-1.11) |
| Congestive heart failure (CHF) | 1.67 (1.65-1.70) | 0.98 (0.96-1.01) | 1.13 (1.08-1.18) | 0.97 (0.93-1.01) | 1.11 (1.07-1.15) | 0.88 (0.83-0.93) |
| Peripheral vascular disease (PVD) | 1.09 (1.06-1.11) | 0.96 (0.93-0.98) | 1.02 (0.97-1.07) | 0.94 (0.89-0.99) | 1.03 (0.99-1.07) | 1.05 (0.99-1.12) |
| Cerebrovascular disease (CVD) | 0.97 (0.96-0.99) | 0.95 (0.92-0.97) | 1.15 (1.10-1.21) | 0.96 (0.91-1.01) | 0.93 (0.89-0.97) | 0.99 (0.93-1.06) |
| Dementia | 1.41 (1.37-1.44) | 0.82 (0.80-0.85) | 0.67 (0.63-0.72) | 0.56 (0.51-0.60) | 1.15 (1.10-1.21) | 1.13 (1.04-1.22) |
| Chronic pulmonary disease (CPD) | 1.19 (1.18-1.20) | 0.97 (0.95-0.99) | 0.94 (0.90-0.98) | 1.07 (1.03-1.11) | 1.04 (1.01-1.08) | 0.89 (0.85-0.93) |
| Rheumatologic disease (RD) | 0.98 (0.96-1.00) | 1.12 (1.09-1.14) | 1.02 (0.97-1.08) | 1.11 (1.05-1.16) | 1.01 (0.97-1.06) | 1.02 (0.97-1.08) |
| Peptic ulcer disease (PUD) | 1.09 (1.05-1.12) | 1.04 (1.00-1.08) | 0.92 (0.84-1.01) | 1.02 (0.93-1.11) | 0.96 (0.89-1.03) | 0.97 (0.87-1.07) |
| Liver Disease |  |  |  |  |  |  |
| No History of Liver Disease | Reference | Reference | Reference | Reference | Reference | Reference |
| Mild Liver Disease | 0.99 (0.97-1.01) | 1.03 (1.00-1.06) | 0.92 (0.86-0.99) | 1.07 (1.01-1.13) | 1.02 (0.96-1.07) | 0.98 (0.91-1.05) |
| Moderate to Severe Liver Disease | 1.71 (1.65-1.76) | 1.25 (1.21-1.31) | 0.78 (0.71-0.87) | 1.28 (1.18-1.38) | 1.67 (1.56-1.78) | 1.38 (1.26-1.51) |
| Diabetes Mellitus |  |  |  |  |  |  |
| No History of Diabetes | Reference | Reference | Reference | Reference | Reference | Reference |
| Diabetes without Chronic Complications | 1.39 (1.37-1.42) | 1.06 (1.03-1.09) | 1.03 (0.97-1.09) | 1.03 (0.98-1.09) | 1.03 (0.99-1.07) | 1.00 (0.94-1.06) |
| Diabetes with Chronic Complications | 1.38 (1.36-1.40) | 1.19 (1.17-1.22) | 1.10 (1.05-1.16) | 1.17 (1.12-1.23) | 1.09 (1.05-1.13) | 1.01 (0.95-1.06) |
| Hemiplegia or paraplegia | 1.84 (1.78-1.91) | 0.93 (0.89-0.97) | 0.88 (0.81-0.96) | 0.96 (0.87-1.05) | 1.07 (1.00-1.15) | 1.00 (0.90-1.12) |
| Renal Disease | 1.78 (1.76-1.80) | 1.98 (1.94-2.02) | 0.89 (0.85-0.93) | 0.91 (0.88-0.95) | 1.16 (1.13-1.20) | 1.09 (1.04-1.15) |
| Cancer |  |  |  |  |  |  |
| No History of Malignant Cancer | Reference | Reference | Reference | Reference | Reference | Reference |
| Any Malignancy Except Neoplasm of skin | 0.99 (0.98-1.01) | 0.92 (0.89-0.94) | 0.80 (0.76-0.84) | 0.84 (0.80-0.88) | 1.10 (1.06-1.14) | 1.13 (1.07-1.19) |
| Metastatic Solid Tumor | 1.93 (1.87-1.99) | 0.89 (0.85-0.93) | 0.72 (0.65-0.80) | 0.79 (0.72-0.87) | 2.03 (1.92-2.14) | 1.66 (1.51-1.83) |
| HIV | 1.03 (0.97-1.09) | 0.94 (0.87-1.02) | 0.91 (0.75-1.11) | 0.87 (0.74-1.02) | 1.01 (0.85-1.19) | 1.20 (0.96-1.49) |
| Obesity | 1.00 (0.99-1.01) | 1.23 (1.21-1.25) | 1.14 (1.10-1.18) | 1.23 (1.19-1.27) | 1.00 (0.97-1.03) | 0.87 (0.83-0.90) |
| Hypertension | 0.85 (0.84-0.86) | 1.12 (1.09-1.14) | 1.05 (1.01-1.10) | 0.86 (0.83-0.90) | 0.84 (0.81-0.87) | 0.91 (0.86-0.95) |
| Former or Current Tobacco User | 1.55 (1.52-1.58) | 1.11 (1.08-1.14) | 1.28 (1.21-1.34) | 0.97 (0.92-1.01) | 0.95 (0.90-0.99) | 0.90 (0.85-0.96) |
| History of Substance Abuse Disorder | 2.58 (2.53-2.63) | 0.97 (0.94-1.00) | 0.91 (0.85-0.97) | 1.07 (1.01-1.12) | 0.97 (0.91-1.02) | 1.02 (0.95-1.10) |
| Vaccination Status Prior to SARS-CoV-2 Infection |  |  |  |  |  |  |
| No Documented COVID-19 Vaccination | Reference | Reference | Reference | Reference | Reference | Reference |
| Primary Vaccination Series | 0.52 (0.52-0.53) | 0.76 (0.74-0.78) | 0.71 (0.68-0.75) | 0.58 (0.55-0.61) | 0.68 (0.65-0.71) | 0.83 (0.78-0.89) |
| Primary+ Vaccination Series | 0.42 (0.42-0.43) | 0.69 (0.67-0.72) | 0.68 (0.64-0.73) | 0.50 (0.47-0.54) | 0.49 (0.47-0.52) | 0.57 (0.52-0.62) |
| Census Region |  |  |  |  |  |  |
| Midwest | Reference | Reference | Reference | Reference | Reference | Reference |
| Northeast | 1.16 (1.15-1.18) | 1.78 (1.74-1.82) | 2.39 (2.27-2.50) | 1.43 (1.36-1.49) | 0.88 (0.85-0.92) | 1.11 (1.05-1.18) |
| South | 1.32 (1.31-1.33) | 2.02 (1.98-2.06) | 2.55 (2.45-2.65) | 2.58 (2.49-2.67) | 1.14 (1.11-1.17) | 1.11 (1.07-1.16) |
| West | 0.88 (0.87-0.89) | 1.78 (1.73-1.83) | 1.67 (1.58-1.77) | 2.75 (2.64-2.87) | 1.10 (1.06-1.14) | 0.88 (0.83-0.93) |
| Rurality |  |  |  |  |  |  |
| Urban | Reference | Reference | Reference | Reference | Reference | Reference |
| Urban-Adjacent Rural | 1.07 (1.05-1.08) | 1.00 (0.98-1.03) | 1.02 (0.97-1.06) | 1.32 (1.27-1.37) | 1.30 (1.26-1.35) | 1.19 (1.14-1.24) |
| Nonurban-Adjacent Rural | 1.06 (1.03-1.08) | 1.08 (1.03-1.13) | 1.05 (0.97-1.14) | 1.58 (1.49-1.68) | 1.37 (1.30-1.45) | 1.28 (1.18-1.38) |
| Nirmatrelvir/ritonavir | 0.06 (0.06-0.07) | 0.36 (0.30-0.43) | 0.62 (0.46-0.83) | 0.17 (0.11-0.27) | 0.38 (0.28-0.50) | 0.54 (0.32-0.90) |
| Molnupiravir | 0.06 (0.05-0.07) | 0.63 (0.49-0.80) | 0.70 (0.44-1.11) | 0.72 (0.45-1.14) | 0.68 (0.46-1.01) | 1.10 (0.66-1.83) |
| Monoclonal Therapies | 0.11 (0.11-0.12) | 0.67 (0.63-0.72) | 0.57 (0.49-0.66) | 0.52 (0.46-0.58) | 0.56 (0.51-0.62) | 0.67 (0.59-0.76) |
| Remdesivir |  | 0.49 (0.48-0.50) | 0.34 (0.32-0.36) | 0.92 (0.89-0.96) | 1.33 (1.29-1.37) | 0.79 (0.76-0.82) |
| Anticoagulants |  | 0.21 (0.20-0.21) | 0.18 (0.17-0.19) | 0.35 (0.34-0.37) | 0.80 (0.78-0.83) | 0.54 (0.52-0.56) |
| Tocilizumab |  |  |  |  |  | 1.36 (1.29-1.44) |
| Dexamethasone |  |  |  |  |  | 0.95 (0.91-0.98) |

**eTable S-11. Multivariable Regression for Adverse Acute COVID-19 Events with COVID-19 Therapies Stratified by Rurality Across All Time Periods after Propensity-Score Matching**

**A. Hospitalization, Acute Kidney Injury (AKI) or Dialysis, and Major Adverse Cardiovascular Event (MACE)**

| **Characteristic** | **Hospitalization** | | **AKI/Dialysis** | | **MACE** | |
| --- | --- | --- | --- | --- | --- | --- |
|  | **Urban** | **Rural** | **Urban** | **Rural** | **Urban** | **Rural** |
|  | **Adjusted Odds Ratio**  **(95% CI)** | **Adjusted Odds Ratio**  **(95% CI)** | **Adjusted Hazard Ratio**  **(95% CI)** | **Adjusted Hazard Ratio**  **(95% CI)** | **Adjusted Hazard Ratio**  **(95% CI)** | **Adjusted Hazard Ratio**  **(95% CI)** |
| Sex |  |  |  |  |  |  |
| Female | Reference | Reference | Reference | Reference | Reference | Reference |
| Male | 1.23 (1.22-1.24) | 1.25 (1.23-1.28) | 1.50 (1.47-1.53) | 1.42 (1.37-1.48) | 1.48 (1.43-1.53) | 1.51 (1.39-1.63) |
| Age at COVID-19 Diagnosis | 1.04 (1.04-1.04) | 1.04 (1.04-1.04) | 1.02 (1.02-1.02) | 1.02 (1.01-1.02) | 1.03 (1.03-1.03) | 1.03 (1.02-1.03) |
| Race/Ethnicity |  |  |  |  |  |  |
| White Non-Hispanic | Reference | Reference | Reference | Reference | Reference | Reference |
| Black or African American Non-Hispanic | 1.72 (1.70-1.74) | 1.18 (1.13-1.23) | 1.63 (1.60-1.66) | 1.70 (1.60-1.81) | 1.31 (1.25-1.36) | 1.50 (1.32-1.70) |
| Hispanic or Latino Any Race | 1.83 (1.80-1.86) | 1.51 (1.43-1.58) | 1.11 (1.08-1.15) | 1.24 (1.12-1.37) | 1.01 (0.95-1.08) | 1.06 (0.84-1.33) |
| Other | 1.05 (1.02-1.07) | 0.39 (0.36-0.43) | 1.22 (1.17-1.28) | 1.14 (0.95-1.38) | 1.28 (1.18-1.39) | 1.28 (0.91-1.79) |
| Missing/Unknown | 0.95 (0.93-0.97) | 0.87 (0.81-0.93) | 1.20 (1.15-1.25) | 1.60 (1.41-1.81) | 1.31 (1.21-1.41) | 1.93 (1.56-2.41) |
| Myocardial infarction (MI) | 1.20 (1.17-1.23) | 1.10 (1.05-1.16) | 1.00 (0.98-1.03) | 0.90 (0.85-0.97) | 2.60 (2.48-2.73) | 2.44 (2.18-2.73) |
| Congestive heart failure (CHF) | 1.70 (1.67-1.73) | 1.52 (1.46-1.58) | 0.98 (0.95-1.00) | 1.02 (0.96-1.08) | 1.13 (1.08-1.19) | 1.11 (0.99-1.25) |
| Peripheral vascular disease (PVD) | 1.08 (1.06-1.11) | 1.11 (1.06-1.16) | 0.95 (0.93-0.98) | 0.98 (0.92-1.05) | 1.01 (0.96-1.07) | 1.09 (0.96-1.24) |
| Cerebrovascular disease (CVD) | 0.97 (0.95-0.99) | 0.98 (0.93-1.02) | 0.95 (0.92-0.98) | 0.93 (0.87-1.01) | 1.14 (1.08-1.21) | 1.19 (1.04-1.35) |
| Dementia | 1.49 (1.45-1.53) | 1.09 (1.02-1.16) | 0.81 (0.78-0.84) | 0.92 (0.84-1.01) | 0.69 (0.65-0.74) | 0.53 (0.43-0.66) |
| Chronic pulmonary disease (CPD) | 1.17 (1.16-1.19) | 1.28 (1.25-1.32) | 0.97 (0.95-0.99) | 0.95 (0.90-1.00) | 0.95 (0.91-1.00) | 0.88 (0.80-0.98) |
| Rheumatologic disease (RD) | 0.97 (0.95-0.99) | 1.04 (1.00-1.08) | 1.12 (1.09-1.15) | 1.12 (1.05-1.19) | 1.04 (0.99-1.10) | 0.92 (0.80-1.05) |
| Peptic ulcer disease (PUD) | 1.08 (1.05-1.12) | 1.11 (1.02-1.21) | 1.05 (1.01-1.10) | 0.94 (0.83-1.06) | 0.96 (0.87-1.06) | 0.72 (0.55-0.95) |
| Liver Disease |  |  |  |  |  |  |
| No History of Liver Disease | Reference | Reference | Reference | Reference | Reference | Reference |
| Mild Liver Disease | 0.98 (0.96-1.00) | 1.03 (0.98-1.09) | 1.04 (1.01-1.08) | 0.98 (0.90-1.07) | 0.96 (0.89-1.03) | 0.74 (0.61-0.90) |
| Moderate to Severe Liver Disease | 1.69 (1.63-1.75) | 1.70 (1.56-1.85) | 1.27 (1.21-1.32) | 1.17 (1.05-1.30) | 0.80 (0.72-0.90) | 0.67 (0.51-0.87) |
| Diabetes Mellitus |  |  |  |  |  |  |
| No History of Diabetes | Reference | Reference | Reference | Reference | Reference | Reference |
| Diabetes without Chronic Complications | 1.38 (1.35-1.40) | 1.42 (1.37-1.48) | 1.08 (1.05-1.11) | 0.99 (0.93-1.07) | 1.04 (0.98-1.11) | 0.95 (0.82-1.09) |
| Diabetes with Chronic Complications | 1.36 (1.34-1.38) | 1.50 (1.44-1.56) | 1.20 (1.17-1.23) | 1.17 (1.11-1.24) | 1.10 (1.04-1.15) | 1.17 (1.04-1.31) |
| Hemiplegia or paraplegia | 1.90 (1.83-1.97) | 1.55 (1.42-1.70) | 0.94 (0.90-0.99) | 0.89 (0.79-1.01) | 0.88 (0.80-0.97) | 0.89 (0.70-1.12) |
| Renal Disease | 1.81 (1.78-1.83) | 1.63 (1.58-1.69) | 1.99 (1.94-2.03) | 1.90 (1.80-2.01) | 0.88 (0.84-0.92) | 0.90 (0.81-1.00) |
| Cancer |  |  |  |  |  |  |
| No History of Malignant Cancer | Reference | Reference | Reference | Reference | Reference | Reference |
| Any Malignancy Except Neoplasm of skin | 0.97 (0.96-0.99) | 1.12 (1.08-1.17) | 0.91 (0.88-0.93) | 0.97 (0.91-1.03) | 0.80 (0.75-0.84) | 0.83 (0.73-0.94) |
| Metastatic Solid Tumor | 1.87 (1.81-1.93) | 2.16 (2.00-2.33) | 0.88 (0.84-0.92) | 0.94 (0.83-1.05) | 0.72 (0.64-0.80) | 0.72 (0.56-0.94) |
| HIV | 1.04 (0.98-1.10) | 0.84 (0.67-1.04) | 0.96 (0.88-1.04) | 0.86 (0.61-1.20) | 0.94 (0.77-1.15) | 0.74 (0.33-1.65) |
| Obesity | 1.02 (1.01-1.03) | 0.95 (0.93-0.98) | 1.22 (1.20-1.25) | 1.27 (1.21-1.33) | 1.12 (1.08-1.17) | 1.24 (1.13-1.37) |
| Hypertension | 0.84 (0.83-0.85) | 0.87 (0.85-0.90) | 1.10 (1.08-1.13) | 1.17 (1.11-1.24) | 1.04 (0.99-1.09) | 1.13 (1.01-1.25) |
| Former or Current Tobacco User | 1.54 (1.51-1.57) | 1.67 (1.61-1.74) | 1.12 (1.09-1.15) | 1.07 (1.00-1.14) | 1.29 (1.21-1.36) | 1.23 (1.09-1.40) |
| History of Substance Abuse Disorder | 2.65 (2.60-2.71) | 2.33 (2.21-2.45) | 0.97 (0.94-1.00) | 1.04 (0.95-1.13) | 0.90 (0.84-0.97) | 1.00 (0.83-1.19) |
| Vaccination Status Prior to SARS-CoV-2 Infection |  |  |  |  |  |  |
| No Documented COVID-19 Vaccination | Reference | Reference | Reference | Reference | Reference | Reference |
| Primary Vaccination Series | 0.52 (0.51-0.53) | 0.50 (0.49-0.52) | 0.77 (0.75-0.79) | 0.70 (0.66-0.75) | 0.71 (0.66-0.75) | 0.76 (0.67-0.86) |
| Primary+ Vaccination Series | 0.42 (0.41-0.43) | 0.42 (0.40-0.43) | 0.70 (0.67-0.72) | 0.67 (0.62-0.73) | 0.67 (0.63-0.72) | 0.72 (0.62-0.84) |
| Census Region |  |  |  |  |  |  |
| Midwest | Reference | Reference | Reference | Reference | Reference | Reference |
| Northeast | 1.24 (1.22-1.25) | 0.56 (0.54-0.58) | 1.73 (1.69-1.78) | 1.85 (1.71-2.01) | 2.35 (2.24-2.48) | 2.28 (1.97-2.64) |
| South | 1.23 (1.22-1.24) | 1.89 (1.84-1.93) | 1.88 (1.84-1.92) | 2.67 (2.55-2.80) | 2.41 (2.30-2.52) | 3.00 (2.74-3.29) |
| West | 0.84 (0.83-0.85) | 1.70 (1.62-1.79) | 1.65 (1.60-1.69) | 3.48 (3.19-3.80) | 1.57 (1.48-1.67) | 2.72 (2.24-3.31) |
| Nirmatrelvir/ritonavir | 0.07 (0.06-0.07) | 0.05 (0.04-0.06) | 0.34 (0.28-0.42) | 0.51 (0.32-0.83) | 0.61 (0.44-0.83) | 0.71 (0.32-1.58) |
| Molnupiravir | 0.07 (0.06-0.08) | 0.04 (0.03-0.05) | 0.64 (0.49-0.83) | 0.58 (0.31-1.08) | 0.77 (0.47-1.26) | 0.43 (0.11-1.71) |
| Monoclonal Therapies | 0.11 (0.11-0.12) | 0.12 (0.11-0.13) | 0.67 (0.62-0.72) | 0.69 (0.60-0.81) | 0.54 (0.46-0.64) | 0.68 (0.49-0.92) |
| Remdesivir |  |  | 0.47 (0.46-0.49) | 0.59 (0.56-0.64) | 0.32 (0.31-0.35) | 0.43 (0.38-0.50) |
| Anticoagulants |  |  | 0.20 (0.20-0.21) | 0.23 (0.22-0.25) | 0.18 (0.17-0.19) | 0.18 (0.16-0.20) |
| Tocilizumab |  |  |  |  |  |  |
| Dexamethasone |  |  |  |  |  |  |

**B. Invasive Mechanical Ventilation (IMV) or Extracorporeal Membrane Oxygenation (ECMO), Death, and Death after IMV/ECMO**

| **Characteristic** | **IMV/ECMO** | | **Death** | | **Death After IMV/ECMO** | |
| --- | --- | --- | --- | --- | --- | --- |
|  | **Urban** | **Rural** | **Urban** | **Rural** | **Urban** | **Rural** |
|  | **Adjusted Hazard Ratio**  **(95% CI)** | **Adjusted Hazard Ratio**  **(95% CI)** | **Adjusted Hazard Ratio (95% CI)** | **Adjusted Hazard Ratio (95% CI)** | **Adjusted Hazard Ratio (95% CI)** | **Adjusted Hazard Ratio (95% CI)** |
| Sex |  |  |  |  |  |  |
| Female | Reference | Reference | Reference | Reference | Reference | Reference |
| Male | 1.63 (1.58-1.68) | 1.53 (1.44-1.62) | 1.38 (1.35-1.42) | 1.26 (1.20-1.33) | 1.08 (1.04-1.12) | 0.94 (0.87-1.01) |
| Age at COVID-19 Diagnosis | 1.01 (1.00-1.01) | 1.00 (1.00-1.00) | 1.04 (1.04-1.04) | 1.03 (1.03-1.04) | 1.03 (1.03-1.03) | 1.03 (1.03-1.03) |
| Race/Ethnicity |  |  |  |  |  |  |
| White Non-Hispanic | Reference | Reference | Reference | Reference | Reference | Reference |
| Black or African American Non-Hispanic | 1.06 (1.02-1.10) | 1.20 (1.08-1.33) | 0.93 (0.89-0.96) | 1.01 (0.91-1.13) | 1.02 (0.97-1.07) | 1.04 (0.91-1.18) |
| Hispanic or Latino Any Race | 1.15 (1.10-1.21) | 1.28 (1.13-1.46) | 1.12 (1.07-1.17) | 1.03 (0.89-1.19) | 1.19 (1.11-1.26) | 1.04 (0.88-1.24) |
| Other | 1.29 (1.20-1.39) | 1.16 (0.90-1.50) | 1.19 (1.11-1.27) | 1.09 (0.85-1.40) | 1.20 (1.09-1.32) | 0.90 (0.61-1.31) |
| Missing/Unknown | 1.33 (1.25-1.42) | 1.75 (1.52-2.02) | 1.22 (1.15-1.30) | 1.86 (1.61-2.15) | 1.17 (1.08-1.28) | 1.44 (1.22-1.71) |
| Myocardial infarction (MI) | 1.02 (0.96-1.08) | 1.03 (0.91-1.17) | 1.05 (1.00-1.09) | 1.06 (0.97-1.17) | 1.03 (0.97-1.11) | 1.09 (0.95-1.25) |
| Congestive heart failure (CHF) | 0.97 (0.93-1.02) | 0.95 (0.85-1.05) | 1.11 (1.07-1.15) | 1.10 (1.02-1.20) | 0.88 (0.83-0.93) | 0.88 (0.78-0.99) |
| Peripheral vascular disease (PVD) | 0.94 (0.88-0.99) | 0.94 (0.82-1.07) | 1.05 (1.00-1.10) | 0.95 (0.86-1.04) | 1.07 (1.00-1.14) | 1.00 (0.86-1.16) |
| Cerebrovascular disease (CVD) | 0.95 (0.89-1.01) | 0.96 (0.84-1.10) | 0.92 (0.88-0.96) | 0.98 (0.88-1.08) | 0.98 (0.92-1.06) | 1.04 (0.90-1.21) |
| Dementia | 0.57 (0.52-0.62) | 0.44 (0.34-0.57) | 1.15 (1.10-1.21) | 1.16 (1.04-1.31) | 1.14 (1.05-1.24) | 1.10 (0.89-1.35) |
| Chronic pulmonary disease (CPD) | 1.07 (1.03-1.11) | 1.08 (1.0-1.18) | 1.05 (1.01-1.08) | 1.05 (0.98-1.13) | 0.89 (0.85-0.94) | 0.87 (0.79-0.97) |
| Rheumatologic disease (RD) | 1.12 (1.07-1.19) | 1.02 (0.91-1.15) | 1.00 (0.96-1.05) | 1.07 (0.98-1.17) | 1.03 (0.96-1.09) | 1.01 (0.89-1.15) |
| Peptic ulcer disease (PUD) | 1.03 (0.94-1.13) | 0.91 (0.73-1.15) | 0.98 (0.90-1.06) | 0.86 (0.72-1.04) | 1.01 (0.90-1.13) | 0.80 (0.61-1.05) |
| Liver Disease |  |  |  |  |  |  |
| No History of Liver Disease | Reference | Reference | Reference | Reference | Reference | Reference |
| Mild Liver Disease | 1.11 (1.05-1.18) | 0.89 (0.77-1.03) | 1.02 (0.96-1.08) | 1.00 (0.88-1.14) | 0.98 (0.90-1.06) | 0.97 (0.81-1.17) |
| Moderate to Severe Liver Disease | 1.29 (1.19-1.40) | 1.22 (1.02-1.46) | 1.67 (1.55-1.79) | 1.64 (1.41-1.91) | 1.37 (1.24-1.52) | 1.37 (1.10-1.71) |
| Diabetes Mellitus |  |  |  |  |  |  |
| No History of Diabetes | Reference | Reference | Reference | Reference | Reference | Reference |
| Diabetes without Chronic Complications | 1.06 (1.00-1.12) | 0.96 (0.85-1.07) | 1.04 (0.99-1.09) | 1.01 (0.92-1.11) | 1.01 (0.95-1.09) | 0.94 (0.82-1.07) |
| Diabetes with Chronic Complications | 1.20 (1.14-1.26) | 1.08 (0.97-1.20) | 1.08 (1.04-1.13) | 1.10 (1.01-1.20) | 1.03 (0.97-1.09) | 0.93 (0.82-1.05) |
| Hemiplegia or paraplegia | 0.97 (0.88-1.07) | 0.88 (0.69-1.11) | 1.08 (1.00-1.17) | 1.05 (0.88-1.26) | 1.00 (0.89-1.12) | 1.05 (0.81-1.37) |
| Renal Disease | 0.91 (0.87-0.95) | 0.91 (0.83-1.00) | 1.16 (1.12-1.21) | 1.16 (1.08-1.25) | 1.09 (1.03-1.15) | 1.10 (0.99-1.23) |
| Cancer |  |  |  |  |  |  |
| No History of Malignant Cancer | Reference | Reference | Reference | Reference | Reference | Reference |
| Any Malignancy Except Neoplasm of skin | 0.83 (0.79-0.88) | 0.85 (0.76-0.95) | 1.09 (1.05-1.14) | 1.14 (1.05-1.24) | 1.14 (1.07-1.21) | 1.10 (0.97-1.24) |
| Metastatic Solid Tumor | 0.81 (0.74-0.90) | 0.70 (0.56-0.87) | 2.07 (1.95-2.19) | 1.87 (1.64-2.13) | 1.65 (1.48-1.83) | 1.75 (1.42-2.17) |
| HIV | 0.91 (0.77-1.08) | 0.45 (0.20-1.00) | 1.00 (0.84-1.19) | 1.19 (0.70-2.02) | 1.14 (0.90-1.44) | 2.04 (1.01-4.11) |
| Obesity | 1.22 (1.18-1.26) | 1.26 (1.17-1.35) | 1.03 (1.00-1.06) | 0.90 (0.84-0.96) | 0.88 (0.84-0.92) | 0.84 (0.77-0.92) |
| Hypertension | 0.87 (0.83-0.90) | 0.86 (0.79-0.93) | 0.84 (0.81-0.87) | 0.87 (0.81-0.93) | 0.90 (0.85-0.95) | 0.94 (0.85-1.04) |
| Former or Current Tobacco User | 0.99 (0.94-1.04) | 0.89 (0.81-0.99) | 0.95 (0.90-1.00) | 0.94 (0.86-1.04) | 0.91 (0.85-0.97) | 0.90 (0.79-1.02) |
| History of Substance Abuse Disorder | 1.05 (1.00-1.12) | 1.11 (0.98-1.25) | 0.98 (0.92-1.04) | 0.94 (0.82-1.08) | 1.02 (0.94-1.10) | 1.05 (0.88-1.25) |
| Vaccination Status Prior to SARS-CoV-2 Infection |  |  |  |  |  |  |
| No Documented COVID-19 Vaccination | Reference | Reference | Reference | Reference | Reference | Reference |
| Primary Vaccination Series | 0.60 (0.57-0.64) | 0.50 (0.44-0.57) | 0.70 (0.67-0.73) | 0.64 (0.58-0.70) | 0.83 (0.78-0.90) | 0.81 (0.70-0.94) |
| Primary+ Vaccination Series | 0.51 (0.47-0.55) | 0.47 (0.40-0.56) | 0.50 (0.47-0.53) | 0.45 (0.40-0.51) | 0.60 (0.55-0.67) | 0.43 (0.34-0.54) |
| Census Region |  |  |  |  |  |  |
| Midwest | Reference | Reference | Reference | Reference | Reference | Reference |
| Northeast | 1.31 (1.25-1.38) | 2.21 (1.95-2.50) | 0.86 (0.82-0.89) | 0.95 (0.85-1.07) | 1.16 (1.09-1.23) | 0.78 (0.66-0.92) |
| South | 2.43 (2.33-2.52) | 3.01 (2.81-3.24) | 1.07 (1.04-1.11) | 1.33 (1.25-1.41) | 1.11 (1.06-1.17) | 1.10 (1.01-1.19) |
| West | 2.44 (2.33-2.56) | 5.46 (4.90-6.08) | 1.01 (0.97-1.06) | 1.83 (1.64-2.05) | 0.86 (0.81-0.92) | 1.07 (0.93-1.22) |
| Nirmatrelvir/ritonavir | 0.18 (0.11-0.29) | 0.07 (0.01-0.50) | 0.36 (0.26-0.49) | 0.51 (0.26-0.98) | 0.58 (0.33-0.99) | 0.29 (0.04-2.08) |
| Molnupiravir | 0.71 (0.42-1.20) | 0.79 (0.30-2.11) | 0.71 (0.46-1.09) | 0.54 (0.20-1.44) | 1.04 (0.60-1.79) | 1.48 (0.37-5.96) |
| Monoclonal Therapies | 0.51 (0.44-0.58) | 0.57 (0.44-0.73) | 0.57 (0.51-0.64) | 0.56 (0.46-0.69) | 0.70 (0.60-0.80) | 0.59 (0.45-0.78) |
| Remdesivir | 0.94 (0.90-0.97) | 0.86 (0.79-0.94) | 1.35 (1.31-1.39) | 1.24 (1.17-1.33) | 0.81 (0.78-0.84) | 0.72 (0.67-0.78) |
| Anticoagulants | 0.35 (0.34-0.37) | 0.34 (0.32-0.37) | 0.79 (0.77-0.81) | 0.83 (0.78-0.88) | 0.55 (0.53-0.58) | 0.49 (0.45-0.53) |
| Tocilizumab |  |  |  |  | 1.37 (1.29-1.46) | 1.29 (1.15-1.45) |
| Dexamethasone |  |  |  |  | 0.91 (0.87-0.95) | 1.11 (1.02-1.21) |

**eTable S-12. Sensitivity Analysis 1: Multivariable Regression of Adverse Acute COVID-19 Events by Rural Binary Across All Time Periods**

| **Characteristic** | **All Time Periods, January 2021 – December 2022** | | | | | | | | | |
| --- | --- | --- | --- | --- | --- | --- | --- | --- | --- | --- |
|  | **Hospitalization** | | **AKI/Dialysis** | | **MACE** | | **ECMO/IMV** | | **Death** | |
|  | **N Events / N (%)** | **Adjusted Odds Ratio (95% CI)** | **N Events / N (%)** | **Adjusted Hazard Ratio (95% CI)** | **N Events / N (%)** | **Adjusted Hazard Ratio (95% CI)** | **N Events / N (%)** | **Adjusted Hazard Ratio (95% CI)** | **N Events / N (%)** | **Adjusted Hazard Ratio (95% CI)** |
| Sex |  |  |  |  |  |  |  |  |  |  |
| Female | 152,354 / 1,723,967 (8.8%) | Reference | 27,568 / 152,354 (18%) | Reference | 6,653 / 152,354 (4.4%) | Reference | 9,168 / 152,354 (6.0%) | Reference | 12,074 / 152,354 (7.9%) | Reference |
| Male | 147,159 / 1,294,679 (11%) | 1.21(1.20, 1.22) | 37,743 / 147,159 (26%) | 1.45(1.43, 1.47) | 9,505 / 147,159 (6.5%) | 1.41(1.36, 1.45) | 13,550 / 147,159 (9.2%) | 1.50(1.46, 1.55) | 16,107 / 147,159 (11%) | 1.35(1.32, 1.38) |
| Age at COVID-19 Diagnosis | 299,513 / 3,018,646 (9.9%) | 1.04(1.04, 1.04) | 65,311 / 299,513 (22%) | 1.02(1.02, 1.02) | 16,158 / 299,513 (5.4%) | 1.03(1.03, 1.03) | 22,718 / 299,513 (7.6%) | 1.00(1.00, 1.00) | 28,181 / 299,513 (9.4%) | 1.04(1.04, 1.04) |
| Race/Ethnicity |  |  |  |  |  |  |  |  |  |  |
| White Non-Hispanic | 188,126 / 2,050,535 (9.2%) | Reference | 37,228 / 188,126 (20%) | Reference | 9,942 / 188,126 (5.3%) | Reference | 13,630 / 188,126 (7.2%) | Reference | 19,007 / 188,126 (10%) | Reference |
| Black or African American Non-Hispanic | 55,922 / 393,047 (14%) | 1.79(1.77, 1.81) | 16,755 / 55,922 (30%) | 1.64(1.61, 1.68) | 3,329 / 55,922 (6.0%) | 1.25(1.20, 1.30) | 4,142 / 55,922 (7.4%) | 1.05(1.01, 1.09) | 4,164 / 55,922 (7.4%) | 0.96(0.93, 0.99) |
| Hispanic or Latino Any Race | 32,094 / 254,459 (13%) | 1.86(1.84, 1.89) | 6,251 / 32,094 (19%) | 1.10(1.06, 1.13) | 1,377 / 32,094 (4.3%) | 0.96(0.91, 1.02) | 2,718 / 32,094 (8.5%) | 1.13(1.08, 1.18) | 2,648 / 32,094 (8.3%) | 1.12(1.08, 1.17) |
| Other | 10,073 / 139,640 (7.2%) | 1.03(1.01, 1.05) | 2,307 / 10,073 (23%) | 1.25(1.19, 1.30) | 658 / 10,073 (6.5%) | 1.29(1.19, 1.40) | 925 / 10,073 (9.2%) | 1.31(1.22, 1.40) | 1,037 / 10,073 (10%) | 1.20(1.13, 1.28) |
| Missing/Unknown | 13,298 / 180,965 (7.3%) | 0.96(0.95, 0.98) | 2,770 / 13,298 (21%) | 1.21(1.17, 1.26) | 852 / 13,298 (6.4%) | 1.31(1.22, 1.41) | 1,303 / 13,298 (9.8%) | 1.37(1.29, 1.45) | 1,325 / 13,298 (10%) | 1.31(1.24, 1.39) |
| Myocardial infarction (MI) |  |  |  |  |  |  |  |  |  |  |
| No History of MI | 277,566 / 2,946,537 (9.4%) | Reference | 57,039 / 277,566 (21%) | Reference | 12,454 / 277,566 (4.5%) | Reference | 20,849 / 277,566 (7.5%) | Reference | 25,119 / 277,566 (9.0%) | Reference |
| History of MI | 21,947 / 72,109 (30%) | 1.18(1.16, 1.20) | 8,272 / 21,947 (38%) | 1.01(0.99, 1.04) | 3,704 / 21,947 (17%) | 2.71(2.59, 2.83) | 1,869 / 21,947 (8.5%) | 1.04(0.98, 1.09) | 3,062 / 21,947 (14%) | 1.05(1.01, 1.10) |
| Congestive heart failure (CHF) |  |  |  |  |  |  |  |  |  |  |
| No History of CHF | 253,151 / 2,876,555 (8.8%) | Reference | 48,143 / 253,151 (19%) | Reference | 11,563 / 253,151 (4.6%) | Reference | 19,055 / 253,151 (7.5%) | Reference | 21,823 / 253,151 (8.6%) | Reference |
| History of CHF | 46,362 / 142,091 (33%) | 1.70(1.67, 1.72) | 17,168 / 46,362 (37%) | 1.06(1.04, 1.09) | 4,595 / 46,362 (9.9%) | 1.22(1.17, 1.28) | 3,663 / 46,362 (7.9%) | 1.06(1.01, 1.10) | 6,358 / 46,362 (14%) | 1.15(1.11, 1.19) |
| Peripheral vascular disease (PVD) |  |  |  |  |  |  |  |  |  |  |
| No History of PVD | 274,298 / 2,929,336 (9.4%) | Reference | 55,968 / 274,298 (20%) | Reference | 13,690 / 274,298 (5.0%) | Reference | 20,784 / 274,298 (7.6%) | Reference | 24,773 / 274,298 (9.0%) | Reference |
| History of PVD | 25,215 / 89,310 (28%) | 1.09(1.07, 1.11) | 9,343 / 25,215 (37%) | 0.98(0.95, 1.00) | 2,468 / 25,215 (9.8%) | 1.05(1.00, 1.10) | 1,934 / 25,215 (7.7%) | 0.96(0.91, 1.02) | 3,408 / 25,215 (14%) | 1.03(0.99, 1.07) |
| Cerebrovascular disease (CVD) |  |  |  |  |  |  |  |  |  |  |
| No History of CVD | 274,577 / 2,921,549 (9.4%) | Reference | 57,072 / 274,577 (21%) | Reference | 13,690 / 274,577 (5.0%) | Reference | 20,942 / 274,577 (7.6%) | Reference | 24,902 / 274,577 (9.1%) | Reference |
| History of CVD | 24,936 / 97,097 (26%) | 0.98(0.96, 1.00) | 8,239 / 24,936 (33%) | 0.96(0.93, 0.99) | 2,468 / 24,936 (9.9%) | 1.16(1.10, 1.22) | 1,776 / 24,936 (7.1%) | 0.98(0.92, 1.03) | 3,279 / 24,936 (13%) | 0.95(0.91, 0.99) |
| Dementia |  |  |  |  |  |  |  |  |  |  |
| No History of dementia | 285,803 / 2,976,228 (9.6%) | Reference | 61,153 / 285,803 (21%) | Reference | 15,175 / 285,803 (5.3%) | Reference | 22,109 / 285,803 (7.7%) | Reference | 25,805 / 285,803 (9.0%) | Reference |
| History of dementia | 13,710 / 42,418 (32%) | 1.49(1.45, 1.52) | 4,158 / 13,710 (30%) | 0.84(0.81, 0.86) | 983 / 13,710 (7.2%) | 0.69(0.65, 0.74) | 609 / 13,710 (4.4%) | 0.59(0.54, 0.64) | 2,376 / 13,710 (17%) | 1.19(1.14, 1.24) |
| Chronic pulmonary disease (CPD) |  |  |  |  |  |  |  |  |  |  |
| No History of CPD | 234,508 / 2,651,794 (8.8%) | Reference | 46,709 / 234,508 (20%) | Reference | 11,694 / 234,508 (5.0%) | Reference | 17,776 / 234,508 (7.6%) | Reference | 20,775 / 234,508 (8.9%) | Reference |
| History of CPD | 65,005 / 366,852 (18%) | 1.19(1.17, 1.20) | 18,602 / 65,005 (29%) | 0.92(0.90, 0.94) | 4,464 / 65,005 (6.9%) | 0.89(0.85, 0.92) | 4,942 / 65,005 (7.6%) | 1.06(1.02, 1.10) | 7,406 / 65,005 (11%) | 1.07(1.04, 1.10) |
| Rheumatologic disease (RD) |  |  |  |  |  |  |  |  |  |  |
| No History of RD | 274,895 / 2,878,861 (9.5%) | Reference | 56,668 / 274,895 (21%) | Reference | 14,318 / 274,895 (5.2%) | Reference | 20,714 / 274,895 (7.5%) | Reference | 25,229 / 274,895 (9.2%) | Reference |
| History of RD | 24,618 / 139,785 (18%) | 0.95(0.94, 0.97) | 8,643 / 24,618 (35%) | 1.11(1.08, 1.14) | 1,840 / 24,618 (7.5%) | 1.00(0.95, 1.05) | 2,004 / 24,618 (8.1%) | 1.09(1.03, 1.14) | 2,952 / 24,618 (12%) | 1.02(0.98, 1.06) |
| Peptic ulcer disease (PUD) |  |  |  |  |  |  |  |  |  |  |
| No History of PUD | 292,694 / 2,986,771 (9.8%) | Reference | 62,879 / 292,694 (21%) | Reference | 15,673 / 292,694 (5.4%) | Reference | 22,154 / 292,694 (7.6%) | Reference | 27,383 / 292,694 (9.4%) | Reference |
| History of PUD | 6,819 / 31,875 (21%) | 1.08(1.05, 1.11) | 2,432 / 6,819 (36%) | 1.07(1.03, 1.12) | 485 / 6,819 (7.1%) | 0.93(0.85, 1.02) | 564 / 6,819 (8.3%) | 1.05(0.96, 1.14) | 798 / 6,819 (12%) | 0.97(0.90, 1.04) |
| Liver Disease |  |  |  |  |  |  |  |  |  |  |
| No History of Liver Disease | 275,696 / 2,885,760 (9.6%) | Reference | 57,790 / 275,696 (21%) | Reference | 14,814 / 275,696 (5.4%) | Reference | 20,511 / 275,696 (7.4%) | Reference | 25,622 / 275,696 (9.3%) | Reference |
| Mild Liver Disease | 17,062 / 112,409 (15%) | 0.99(0.97, 1.01) | 4,796 / 17,062 (28%) | 1.02(0.99, 1.06) | 958 / 17,062 (5.6%) | 0.92(0.86, 0.98) | 1,432 / 17,062 (8.4%) | 1.07(1.01, 1.13) | 1,529 / 17,062 (9.0%) | 1.02(0.97, 1.08) |
| Moderate to Severe Liver Disease | 6,755 / 20,477 (33%) | 1.70(1.64, 1.76) | 2,725 / 6,755 (40%) | 1.36(1.31, 1.41) | 386 / 6,755 (5.7%) | 0.87(0.79, 0.97) | 775 / 6,755 (11%) | 1.41(1.30, 1.52) | 1,030 / 6,755 (15%) | 1.71(1.61, 1.83) |
| Diabetes Mellitus |  |  |  |  |  |  |  |  |  |  |
| No History of Diabetes | 228,087 / 2,673,796 (8.5%) | Reference | 41,397 / 228,087 (18%) | Reference | 10,850 / 228,087 (4.8%) | Reference | 16,944 / 228,087 (7.4%) | Reference | 20,061 / 228,087 (8.8%) | Reference |
| Diabetes without Chronic Complications | 26,535 / 167,616 (16%) | 1.38(1.36, 1.40) | 6,034 / 26,535 (23%) | 1.06(1.03, 1.09) | 1,481 / 26,535 (5.6%) | 1.03(0.97, 1.09) | 1,915 / 26,535 (7.2%) | 1.04(0.99, 1.09) | 2,528 / 26,535 (9.5%) | 1.03(0.98, 1.07) |
| Diabetes with Chronic Complications | 44,891 / 177,234 (25%) | 1.37(1.35, 1.39) | 17,880 / 44,891 (40%) | 1.25(1.22, 1.28) | 3,827 / 44,891 (8.5%) | 1.14(1.09, 1.19) | 3,859 / 44,891 (8.6%) | 1.20(1.15, 1.25) | 5,592 / 44,891 (12%) | 1.09(1.05, 1.13) |
| Hemiplegia or paraplegia |  |  |  |  |  |  |  |  |  |  |
| No History of Hemiplegia or paraplegia | 291,851 / 2,997,719 (9.7%) | Reference | 62,855 / 291,851 (22%) | Reference | 15,525 / 291,851 (5.3%) | Reference | 22,143 / 291,851 (7.6%) | Reference | 27,264 / 291,851 (9.3%) | Reference |
| History of Hemiplegia or paraplegia | 7,662 / 20,927 (37%) | 1.91(1.85, 1.98) | 2,456 / 7,662 (32%) | 0.94(0.90, 0.98) | 633 / 7,662 (8.3%) | 0.91(0.83, 0.99) | 575 / 7,662 (7.5%) | 1.00(0.91, 1.10) | 917 / 7,662 (12%) | 1.10(1.02, 1.18) |
| Renal Disease |  |  |  |  |  |  |  |  |  |  |
| No History of Renal Disease | 231,487 / 2,776,023 (8.3%) | Reference | 35,991 / 231,487 (16%) | Reference | 10,669 / 231,487 (4.6%) | Reference | 17,360 / 231,487 (7.5%) | Reference | 19,264 / 231,487 (8.3%) | Reference |
| History of Disease Renal | 68,026 / 242,623 (28%) | 1.80(1.78, 1.82) | 29,320 / 68,026 (43%) | 2.37(2.32, 2.42) | 5,489 / 68,026 (8.1%) | 1.04(1.0, 1.08) | 5,358 / 68,026 (7.9%) | 1.05(1.01, 1.09) | 8,917 / 68,026 (13%) | 1.21(1.17, 1.25) |
| Cancer |  |  |  |  |  |  |  |  |  |  |
| No History of Malignant Cancer | 261,778 / 2,810,830 (9.3%) | Reference | 54,838 / 261,778 (21%) | Reference | 13,922 / 261,778 (5.3%) | Reference | 20,212 / 261,778 (7.7%) | Reference | 22,834 / 261,778 (8.7%) | Reference |
| Any Malignancy Except Neoplasm of skin | 30,402 / 180,441 (17%) | 0.98(0.96, 0.99) | 8,519 / 30,402 (28%) | 0.93(0.90, 0.95) | 1,861 / 30,402 (6.1%) | 0.80(0.76, 0.85) | 2,016 / 30,402 (6.6%) | 0.87(0.83, 0.92) | 3,921 / 30,402 (13%) | 1.11(1.07, 1.15) |
| Metastatic Solid Tumor | 7,333 / 27,375 (27%) | 1.88(1.82, 1.94) | 1,954 / 7,333 (27%) | 0.93(0.89, 0.98) | 375 / 7,333 (5.1%) | 0.75(0.68, 0.83) | 490 / 7,333 (6.7%) | 0.87(0.79, 0.95) | 1,426 / 7,333 (19%) | 2.11(2.00, 2.23) |
| HIV |  |  |  |  |  |  |  |  |  |  |
| No History of HIV | 297,598 / 3,007,210 (9.9%) | Reference | 64,743 / 297,598 (22%) | Reference | 16,055 / 297,598 (5.4%) | Reference | 22,571 / 297,598 (7.6%) | Reference | 28,043 / 297,598 (9.4%) | Reference |
| History of HIV | 1,915 / 11,436 (17%) | 0.99(0.93, 1.04) | 568 / 1,915 (30%) | 0.95(0.87, 1.03) | 103 / 1,915 (5.4%) | 0.93(0.76, 1.13) | 147 / 1,915 (7.7%) | 0.87(0.74, 1.03) | 138 / 1,915 (7.2%) | 1.00(0.85, 1.19) |
| Obesity |  |  |  |  |  |  |  |  |  |  |
| No History of Obesity | 199,107 / 2,196,551 (9.1%) | Reference | 38,746 / 199,107 (19%) | Reference | 10,425 / 199,107 (5.2%) | Reference | 14,634 / 199,107 (7.3%) | Reference | 19,024 / 199,107 (9.6%) | Reference |
| History of Obesity | 100,406 / 822,095 (12%) | 0.94(0.93, 0.95) | 26,565 / 100,406 (26%) | 1.05(1.03, 1.07) | 5,733 / 100,406 (5.7%) | 0.94(0.90, 0.97) | 8,084 / 100,406 (8.1%) | 1.07(1.04, 1.10) | 9,157 / 100,406 (9.1%) | 0.99(0.96, 1.01) |
| Hypertension |  |  |  |  |  |  |  |  |  |  |
| No History of Hypertension | 169,511 / 2,238,582 (7.6%) | Reference | 25,741 / 169,511 (15%) | Reference | 6,867 / 169,511 (4.1%) | Reference | 13,196 / 169,511 (7.8%) | Reference | 13,642 / 169,511 (8.0%) | Reference |
| History of Hypertension | 130,002 / 780,064 (17%) | 0.84(0.83, 0.85) | 39,570 / 130,002 (30%) | 1.13(1.11, 1.16) | 9,291 / 130,002 (7.1%) | 1.06(1.01, 1.11) | 9,522 / 130,002 (7.3%) | 0.88(0.85, 0.91) | 14,539 / 130,002 (11%) | 0.85(0.82, 0.87) |
| Former or Current Tobacco User |  |  |  |  |  |  |  |  |  |  |
| No History of Tobacco Use | 272,191 / 2,883,115 (9.4%) | Reference | 57,989 / 272,191 (21%) | Reference | 14,232 / 272,191 (5.2%) | Reference | 20,339 / 272,191 (7.5%) | Reference | 25,920 / 272,191 (9.5%) | Reference |
| History of Tobacco Use | 27,322 / 135,531 (20%) | 1.59(1.57, 1.62) | 7,322 / 27,322 (27%) | 1.10(1.07, 1.13) | 1,926 / 27,322 (7.0%) | 1.23(1.17, 1.30) | 2,379 / 27,322 (8.7%) | 0.98(0.94, 1.03) | 2,261 / 27,322 (8.3%) | 0.99(0.94, 1.03) |
| History of Substance Abuse Disorder |  |  |  |  |  |  |  |  |  |  |
| No History of Substance Abuse | 278,746 / 2,937,997 (9.5%) | Reference | 60,118 / 278,746 (22%) | Reference | 15,078 / 278,746 (5.4%) | Reference | 20,730 / 278,746 (7.4%) | Reference | 26,704 / 278,746 (9.6%) | Reference |
| History of Substance Abuse | 20,767 / 80,649 (26%) | 2.69(2.64, 2.75) | 5,193 / 20,767 (25%) | 1.01(0.98, 1.04) | 1,080 / 20,767 (5.2%) | 0.97(0.91, 1.04) | 1,988 / 20,767 (9.6%) | 1.16(1.10, 1.22) | 1,477 / 20,767 (7.1%) | 0.99(0.93, 1.05) |
| Variant Period |  |  |  |  |  |  |  |  |  |  |
| Pre-Delta (December 10, 2020 - June 14, 2021) | 72,260 / 528,579 (14%) | Reference | 15,824 / 72,260 (22%) | Reference | 4,090 / 72,260 (5.7%) | Reference | 5,839 / 72,260 (8.1%) | Reference | 7,260 / 72,260 (10%) | Reference |
| Delta (June 15, 2021 - December 21, 2021) | 87,063 / 778,430 (11%) | 0.97(0.96, 0.98) | 18,176 / 87,063 (21%) | 1.09(1.06, 1.11) | 3,781 / 87,063 (4.3%) | 0.89(0.85, 0.93) | 8,518 / 87,063 (9.8%) | 1.24(1.20, 1.29) | 9,853 / 87,063 (11%) | 1.28(1.24, 1.33) |
| Omicron (>= December 22 ,2021) | 140,190 / 1,711,637 (8.2%) | 0.58(0.57, 0.58) | 31,311 / 140,190 (22%) | 0.95(0.93, 0.97) | 8,287 / 140,190 (5.9%) | 1.01(0.97, 1.05) | 8,361 / 140,190 (6.0%) | 0.75(0.73, 0.78) | 11,068 / 140,190 (7.9%) | 0.74(0.72, 0.76) |
| Vaccination Status Prior to SARS-CoV-2 Infection |  |  |  |  |  |  |  |  |  |  |
| No Documented COVID-19 Vaccination | 250,484 / 2,278,507 (11%) | Reference | 54,791 / 250,484 (22%) | Reference | 13,767 / 250,484 (5.5%) | Reference | 20,444 / 250,484 (8.2%) | Reference | 24,186 / 250,484 (9.7%) | Reference |
| Primary Vaccination Series | 29,348 / 434,199 (6.8%) | 0.56(0.55, 0.56) | 6,301 / 29,348 (21%) | 0.80(0.78, 0.82) | 1,398 / 29,348 (4.8%) | 0.76(0.72, 0.81) | 1,430 / 29,348 (4.9%) | 0.65(0.61, 0.68) | 2,546 / 29,348 (8.7%) | 0.70(0.67, 0.73) |
| Primary+ Vaccination Series | 19,681 / 305,940 (6.4%) | 0.47(0.47, 0.48) | 4,219 / 19,681 (21%) | 0.75(0.73, 0.77) | 993 / 19,681 (5.0%) | 0.70(0.65, 0.75) | 844 / 19,681 (4.3%) | 0.65(0.61, 0.70) | 1,449 / 19,681 (7.4%) | 0.61(0.58, 0.65) |
| Census Region |  |  |  |  |  |  |  |  |  |  |
| Midwest | 115,990 / 1,422,648 (8.2%) | Reference | 19,826 / 115,990 (17%) | Reference | 4,341 / 115,990 (3.7%) | Reference | 5,690 / 115,990 (4.9%) | Reference | 10,390 / 115,990 (9.0%) | Reference |
| Northeast | 51,533 / 370,570 (14%) | 1.26(1.25, 1.28) | 11,749 / 51,533 (23%) | 1.37(1.34, 1.41) | 3,542 / 51,533 (6.9%) | 1.63(1.56, 1.71) | 3,009 / 51,533 (5.8%) | 1.22(1.17, 1.28) | 4,611 / 51,533 (8.9%) | 0.93(0.89, 0.96) |
| South | 94,976 / 802,396 (12%) | 1.23(1.22, 1.24) | 24,402 / 94,976 (26%) | 1.53(1.50, 1.56) | 6,260 / 94,976 (6.6%) | 1.70(1.64, 1.77) | 9,607 / 94,976 (10%) | 2.17(2.10, 2.25) | 9,329 / 94,976 (9.8%) | 1.19(1.16, 1.23) |
| West | 37,014 / 423,032 (8.7%) | 0.91(0.90, 0.93) | 9,334 / 37,014 (25%) | 1.63(1.59, 1.67) | 2,015 / 37,014 (5.4%) | 1.42(1.34, 1.50) | 4,412 / 37,014 (12%) | 2.57(2.47, 2.68) | 3,851 / 37,014 (10%) | 1.16(1.11, 1.21) |
| Rural-Dwelling Status |  |  |  |  |  |  |  |  |  |  |
| Urban | 248,770 / 2,512,442 (9.9%) | Reference | 55,092 / 248,770 (22%) | Reference | 13,458 / 248,770 (5.4%) | Reference | 18,060 / 248,770 (7.3%) | Reference | 22,253 / 248,770 (8.9%) | Reference |
| Rural | 50,743 / 506,204 (10%) | 1.06(1.05, 1.07) | 10,219 / 50,743 (20%) | 1.05(1.03, 1.08) | 2,700 / 50,743 (5.3%) | 1.08(1.03, 1.13) | 4,658 / 50,743 (9.2%) | 1.38(1.33, 1.43) | 5,928 / 50,743 (12%) | 1.29(1.25, 1.33) |

**eTable S-13. Sensitivity Analysis 2: Multivariable Regression of Adverse Acute COVID-19 Events by Rurality Across All Time Periods among Patients who Survived the Initial 3 days After Acute COVID-19 Events**

| **Characteristic** | **Overall** | | | | |
| --- | --- | --- | --- | --- | --- |
|  | **Hospitalization** | **AKI/Dialysis** | **MACE** | **IMV/ECMO** | **Death** |
|  | **Adjusted Odds Ratio**  **(95% CI)** | **Adjusted Hazard Ratio**  **(95% CI)** | **Adjusted Hazard Ratio**  **(95% CI)** | **Adjusted Hazard Ratio**  **(95% CI)** | **Adjusted Hazard Ratio**  **(95% CI)** |
| Sex |  |  |  |  |  |
| Female | Reference | Reference | Reference | Reference | Reference |
| Male | 1.21 (1.20-1.22) | 1.46 (1.43-1.48) | 1.42 (1.37-1.47) | 1.52 (1.48-1.56) | 1.39 (1.35-1.43) |
| Age at COVID-19 Diagnosis, Median (IQR) | 1.04 (1.04-1.04) | 1.02 (1.02-1.02) | 1.03 (1.03-1.03) | 1.00 (1.00-1.00) | 1.04 (1.04-1.04) |
| Race/Ethnicity |  |  |  |  |  |
| White Non-Hispanic | Reference | Reference | Reference | Reference | Reference |
| Black or African American Non-Hispanic | 1.79 (1.77-1.81) | 1.65 (1.62-1.69) | 1.25 (1.20-1.31) | 1.04 (1.00-1.08) | 0.96 (0.92-0.99) |
| Hispanic or Latino Any Race | 1.86 (1.84-1.89) | 1.10 (1.07-1.13) | 0.97 (0.91-1.03) | 1.14 (1.09-1.19) | 1.15 (1.10-1.20) |
| Other | 1.03 (1.01-1.05) | 1.25 (1.19-1.30) | 1.28 (1.18-1.39) | 1.30 (1.21-1.39) | 1.21 (1.13-1.29) |
| Missing/Unknown | 0.96 (0.94-0.98) | 1.19 (1.15-1.24) | 1.30 (1.20-1.40) | 1.31 (1.23-1.40) | 1.22 (1.14-1.30) |
| Myocardial infarction (MI) | 1.18 (1.16-1.20) | 1.01 (0.99-1.04) | 2.74 (2.62-2.87) | 1.03 (0.97-1.08) | 1.05 (1.00-1.09) |
| Congestive heart failure (CHF) | 1.69 (1.67-1.72) | 1.06 (1.04-1.09) | 1.23 (1.17-1.28) | 1.05 (1.01-1.10) | 1.11 (1.07-1.15) |
| Peripheral vascular disease (PVD) | 1.09 (1.07-1.11) | 0.97 (0.95-1.00) | 1.05 (1.00-1.11) | 0.95 (0.90-1.01) | 1.04 (0.99-1.08) |
| Cerebrovascular disease (CVD) | 0.98 (0.96-1.00) | 0.96 (0.94-0.99) | 1.15 (1.10-1.22) | 0.97 (0.92-1.03) | 0.96 (0.92-1.00) |
| Dementia | 1.49 (1.45-1.52) | 0.83 (0.80-0.86) | 0.68 (0.64-0.73) | 0.58 (0.53-0.63) | 1.17 (1.12-1.23) |
| Chronic pulmonary disease (CPD) | 1.19 (1.17-1.20) | 0.92 (0.90-0.94) | 0.89 (0.85-0.92) | 1.07 (1.03-1.12) | 1.07 (1.04-1.11) |
| Rheumatologic disease (RD) | 0.95 (0.94-0.97) | 1.11 (1.09-1.14) | 1.00 (0.95-1.05) | 1.10 (1.04-1.15) | 1.04 (1.0-1.08) |
| Peptic ulcer disease (PUD) | 1.08 (1.05-1.12) | 1.08 (1.03-1.12) | 0.93 (0.85-1.02) | 1.05 (0.96-1.15) | 0.99 (0.92-1.07) |
| Liver Disease |  |  |  |  |  |
| No History of Liver Disease | Reference | Reference | Reference | Reference | Reference |
| Mild Liver Disease | 0.99 (0.97-1.01) | 1.02 (0.99-1.06) | 0.92 (0.86-0.98) | 1.07 (1.01-1.14) | 1.04 (0.98-1.10) |
| Moderate to Severe Liver Disease | 1.69 (1.63-1.75) | 1.35 (1.30-1.41) | 0.88 (0.79-0.98) | 1.36 (1.26-1.48) | 1.68 (1.57-1.80) |
| Diabetes Mellitus |  |  |  |  |  |
| No History of Diabetes | Reference | Reference | Reference | Reference | Reference |
| Diabetes without Chronic Complications | 1.38 (1.36-1.40) | 1.07 (1.04-1.10) | 1.03 (0.98-1.09) | 1.05 (1.00-1.10) | 1.05 (1.00-1.10) |
| Diabetes with Chronic Complications | 1.37 (1.35-1.39) | 1.26 (1.23-1.28) | 1.14 (1.09-1.20) | 1.21 (1.16-1.27) | 1.11 (1.06-1.15) |
| Hemiplegia or paraplegia | 1.91 (1.84-1.97) | 0.94 (0.90-0.98) | 0.91 (0.83-0.99) | 0.99 (0.90-1.09) | 1.07 (0.99-1.15) |
| Renal Disease | 1.80 (1.78-1.82) | 2.40 (2.35-2.45) | 1.04 (1.00-1.09) | 1.05 (1.01-1.09) | 1.21 (1.17-1.25) |
| Cancer |  |  |  |  |  |
| No History of Malignant Cancer | Reference | Reference | Reference | Reference | Reference |
| Any Malignancy Except Neoplasm of skin | 0.98 (0.97-1.0) | 0.93 (0.90-0.95) | 0.80 (0.76-0.84) | 0.88 (0.84-0.92) | 1.14 (1.10-1.18) |
| Metastatic Solid Tumor | 1.87 (1.82-1.93) | 0.93 (0.89-0.98) | 0.74 (0.67-0.83) | 0.84 (0.77-0.93) | 2.17 (2.04-2.30) |
| HIV | 0.99 (0.93-1.04) | 0.94 (0.87-1.02) | 0.92 (0.76-1.13) | 0.88 (0.74-1.04) | 1.02 (0.86-1.23) |
| Obesity | 0.94 (0.93-0.95) | 1.05 (1.03-1.07) | 0.93 (0.90-0.97) | 1.08 (1.05-1.12) | 1.01 (0.98-1.04) |
| Hypertension | 0.84 (0.84-0.85) | 1.14 (1.12-1.17) | 1.07 (1.02-1.11) | 0.90 (0.86-0.93) | 0.87 (0.84-0.90) |
| Former or Current Tobacco User | 1.59 (1.56-1.62) | 1.09 (1.07-1.12) | 1.24 (1.18-1.31) | 0.97 (0.92-1.02) | 0.97 (0.93-1.02) |
| History of Substance Abuse Disorder | 2.69 (2.64-2.74) | 1.00 (0.97-1.04) | 0.97 (0.90-1.04) | 1.15 (1.10-1.22) | 0.96 (0.91-1.02) |
| Variant Period |  |  |  |  |  |
| Pre-Delta (December 10, 2020 - June 14, 2021) | Reference | Reference | Reference | Reference | Reference |
| Delta (June 15, 2021 - December 21, 2021) | 0.97 (0.96-0.98) | 1.09 (1.06-1.11) | 0.89 (0.85-0.93) | 1.25 (1.21-1.29) | 1.32 (1.27-1.36) |
| Omicron (>= December 22 ,2021) | 0.57 (0.57-0.58) | 0.94 (0.92-0.96) | 1.00 (0.96-1.04) | 0.73 (0.70-0.75) | 0.70 (0.68-0.72) |
| Vaccination Status Prior to SARS-CoV-2 Infection |  |  |  |  |  |
| No Documented COVID-19 Vaccination | Reference | Reference | Reference | Reference | Reference |
| Primary Vaccination Series | 0.56 (0.55-0.56) | 0.80 (0.78-0.83) | 0.76 (0.72-0.81) | 0.65 (0.61-0.68) | 0.69 (0.66-0.72) |
| Primary+ Vaccination Series | 0.48 (0.47-0.48) | 0.75 (0.73-0.78) | 0.71 (0.66-0.76) | 0.66 (0.61-0.71) | 0.62 (0.58-0.66) |
| Census Region |  |  |  |  |  |
| Midwest | Reference | Reference | Reference | Reference | Reference |
| Northeast | 1.26 (1.25-1.28) | 1.38 (1.34-1.41) | 1.65 (1.57-1.73) | 1.25 (1.19-1.31) | 0.90 (0.87-0.94) |
| South | 1.23 (1.22-1.24) | 1.53 (1.50-1.56) | 1.70 (1.63-1.77) | 2.20 (2.12-2.27) | 1.16 (1.12-1.20) |
| West | 0.91 (0.90-0.92) | 1.62 (1.58-1.67) | 1.41 (1.33-1.49) | 2.63 (2.52-2.74) | 1.13 (1.09-1.18) |
| Number of Visits before Acute COVID-19 |  |  |  |  |  |
| Rural-Dwelling Status |  |  |  |  |  |
| Urban | Reference | Reference | Reference | Reference | Reference |
| Urban-Adjacent Rural | 1.06 (1.04-1.07) | 1.04 (1.01-1.07) | 1.07 (1.02-1.13) | 1.32 (1.27-1.38) | 1.25 (1.21-1.30) |
| Nonurban-Adjacent Rural | 1.05 (1.03-1.08) | 1.08 (1.03-1.13) | 1.07 (0.99-1.17) | 1.56 (1.46-1.66) | 1.31 (1.24-1.40) |

**eTable S-14. Sensitivity Analysis 3: Multivariable Regression of Adverse Acute COVID-19 Events by Rurality Across All Time Periods, Including Adjustments for Visit History Prior to COVID-19**

| **Characteristic** | **Overall** | | | | |
| --- | --- | --- | --- | --- | --- |
|  | **Hospitalization** | **AKI/Dialysis** | **MACE** | **IMV/ECMO** | **Death** |
|  | **Adjusted Odds Ratio**  **(95% CI)** | **Adjusted Hazard Ratio**  **(95% CI)** | **Adjusted Hazard Ratio**  **(95% CI)** | **Adjusted Hazard Ratio**  **(95% CI)** | **Adjusted Hazard Ratio**  **(95% CI)** |
| Sex |  |  |  |  |  |
| Female | Reference | Reference | Reference | Reference | Reference |
| Male | 1.19 (1.18-1.20) | 1.45 (1.42-1.47) | 1.39 (1.35-1.44) | 1.50 (1.46-1.54) | 1.35 (1.32-1.38) |
| Age at COVID-19 Diagnosis, Median (IQR) | 1.04 (1.04-1.04) | 1.02 (1.02-1.02) | 1.03 (1.03-1.03) | 1.00 (1.00-1.00) | 1.04 (1.04-1.04) |
| Race/Ethnicity |  |  |  |  |  |
| White Non-Hispanic | Reference | Reference | Reference | Reference | Reference |
| Black or African American Non-Hispanic | 1.76 (1.74-1.78) | 1.64 (1.61-1.67) | 1.23 (1.18-1.29) | 1.05 (1.01-1.09) | 0.96 (0.92-0.99) |
| Hispanic or Latino Any Race | 1.83 (1.80-1.85) | 1.09 (1.06-1.12) | 0.95 (0.89-1.01) | 1.13 (1.08-1.18) | 1.12 (1.08-1.17) |
| Other | 1.01 (0.99-1.03) | 1.24 (1.19-1.30) | 1.27 (1.17-1.38) | 1.30 (1.22-1.40) | 1.20 (1.13-1.28) |
| Missing/Unknown | 0.94 (0.93-0.96) | 1.21 (1.16-1.26) | 1.29 (1.20-1.39) | 1.37 (1.29-1.45) | 1.31 (1.23-1.38) |
| Myocardial infarction (MI) | 1.19 (1.17-1.22) | 1.01 (0.99-1.04) | 2.72 (2.60-2.84) | 1.04 (0.98-1.09) | 1.05 (1.01-1.10) |
| Congestive heart failure (CHF) | 1.76 (1.74-1.79) | 1.07 (1.05-1.09) | 1.25 (1.19-1.30) | 1.06 (1.02-1.11) | 1.15 (1.11-1.19) |
| Peripheral vascular disease (PVD) | 1.13 (1.11-1.15) | 0.98 (0.96-1.01) | 1.08 (1.03-1.14) | 0.97 (0.92-1.03) | 1.04 (1.00-1.08) |
| Cerebrovascular disease (CVD) | 1.01 (0.99-1.03) | 0.96 (0.94-0.99) | 1.18 (1.13-1.25) | 0.98 (0.93-1.04) | 0.95 (0.91-0.99) |
| Dementia | 1.52 (1.48-1.55) | 0.84 (0.81-0.86) | 0.69 (0.65-0.74) | 0.59 (0.54-0.64) | 1.19 (1.14-1.24) |
| Chronic pulmonary disease (CPD) | 1.24 (1.23-1.26) | 0.93 (0.91-0.94) | 0.91 (0.87-0.95) | 1.07 (1.03-1.11) | 1.07 (1.04-1.11) |
| Rheumatologic disease (RD) | 1.02 (1.00-1.03) | 1.12 (1.09-1.15) | 1.04 (0.99-1.09) | 1.10 (1.05-1.15) | 1.02 (0.98-1.07) |
| Peptic ulcer disease (PUD) | 1.15 (1.11-1.18) | 1.08 (1.03-1.12) | 0.96 (0.88-1.05) | 1.06 (0.97-1.15) | 0.98 (0.91-1.05) |
| Liver Disease |  |  |  |  |  |
| No History of Liver Disease | Reference | Reference | Reference | Reference | Reference |
| Mild Liver Disease | 1.04 (1.02-1.06) | 1.03 (1.00-1.06) | 0.94 (0.88-1.01) | 1.08 (1.02-1.14) | 1.03 (0.97-1.08) |
| Moderate to Severe Liver Disease | 1.79 (1.73-1.86) | 1.37 (1.31-1.42) | 0.90 (0.81-0.99) | 1.42 (1.32-1.53) | 1.72 (1.61-1.84) |
| Diabetes Mellitus |  |  |  |  |  |
| No History of Diabetes | Reference | Reference | Reference | Reference | Reference |
| Diabetes without Chronic Complications | 1.38 (1.36-1.41) | 1.06 (1.03-1.09) | 1.02 (0.97-1.08) | 1.04 (0.99-1.09) | 1.03 (0.98-1.07) |
| Diabetes with Chronic Complications | 1.42 (1.40-1.44) | 1.26 (1.23-1.29) | 1.16 (1.11-1.21) | 1.21 (1.15-1.26) | 1.09 (1.05-1.13) |
| Hemiplegia or paraplegia | 1.94 (1.88-2.01) | 0.94 (0.90-0.98) | 0.91 (0.83-0.99) | 1.00 (0.92-1.10) | 1.10 (1.02-1.18) |
| Renal Disease | 1.86 (1.84-1.89) | 2.38 (2.33-2.43) | 1.06 (1.02-1.11) | 1.06 (1.02-1.10) | 1.21 (1.17-1.25) |
| Cancer |  |  |  |  |  |
| No History of Malignant Cancer | Reference | Reference | Reference | Reference | Reference |
| Any Malignancy Except Neoplasm of skin | 1.05 (1.04-1.07) | 0.94 (0.91-0.96) | 0.85 (0.81-0.89) | 0.89 (0.85-0.93) | 1.12 (1.08-1.16) |
| Metastatic Solid Tumor | 2.15 (2.09-2.22) | 0.95 (0.91-0.99) | 0.81 (0.73-0.90) | 0.89 (0.82-0.98) | 2.14 (2.03-2.26) |
| HIV | 1.03 (0.97-1.09) | 0.95 (0.87-1.03) | 0.95 (0.78-1.15) | 0.88 (0.75-1.04) | 1.01 (0.85-1.19) |
| Obesity | 0.98 (0.97-0.99) | 1.05 (1.03-1.07) | 0.96 (0.93-1.00) | 1.08 (1.04-1.11) | 0.99 (0.96-1.02) |
| Hypertension | 0.87 (0.86-0.88) | 1.14 (1.11-1.16) | 1.08 (1.03-1.13) | 0.89 (0.85-0.92) | 0.85 (0.82-0.87) |
| Former or Current Tobacco User | 1.61 (1.58-1.64) | 1.10 (1.07-1.13) | 1.23 (1.17-1.30) | 0.98 (0.94-1.03) | 0.99 (0.94-1.03) |
| History of Substance Abuse Disorder | 2.74 (2.69-2.80) | 1.01 (0.98-1.04) | 0.97 (0.91-1.04) | 1.16 (1.10-1.22) | 0.99 (0.93-1.05) |
| Variant Period |  |  |  |  |  |
| Pre-Delta (December 10, 2020 - June 14, 2021) | Reference | Reference | Reference | Reference | Reference |
| Delta (June 15, 2021 - December 21, 2021) | 0.98 (0.97-1.0) | 1.09 (1.06-1.11) | 0.90 (0.86-0.94) | 1.25 (1.20-1.29) | 1.29 (1.25-1.33) |
| Omicron (>= December 22 ,2021) | 0.59 (0.59-0.60) | 0.95 (0.93-0.97) | 1.02 (0.98-1.06) | 0.75 (0.73-0.78) | 0.74 (0.72-0.76) |
| Vaccination Status Prior to SARS-CoV-2 Infection |  |  |  |  |  |
| No Documented COVID-19 Vaccination | Reference | Reference | Reference | Reference | Reference |
| Primary Vaccination Series | 0.56 (0.56-0.57) | 0.80 (0.78-0.83) | 0.78 (0.73-0.82) | 0.65 (0.62-0.69) | 0.70 (0.67-0.73) |
| Primary+ Vaccination Series | 0.49 (0.49-0.50) | 0.76 (0.73-0.78) | 0.73 (0.68-0.78) | 0.66 (0.61-0.71) | 0.62 (0.58-0.65) |
| Census Region |  |  |  |  |  |
| Midwest | Reference | Reference | Reference | Reference | Reference |
| Northeast | 1.27 (1.25-1.29) | 1.37 (1.34-1.41) | 1.63 (1.55-1.71) | 1.22 (1.16-1.28) | 0.92 (0.89-0.96) |
| South | 1.20 (1.18-1.21) | 1.53 (1.50-1.56) | 1.66 (1.60-1.73) | 2.16 (2.08-2.23) | 1.19 (1.15-1.22) |
| West | 0.92 (0.90-0.93) | 1.63 (1.59-1.67) | 1.42 (1.35-1.50) | 2.56 (2.46-2.67) | 1.16 (1.11-1.21) |
| Rural-Dwelling Status |  |  |  |  |  |
| Urban | Reference | Reference | Reference | Reference | Reference |
| Urban-Adjacent Rural | 1.06 (1.05-1.08) | 1.04 (1.02-1.07) | 1.08 (1.03-1.13) | 1.33 (1.28-1.38) | 1.28 (1.24-1.32) |
| Nonurban-Adjacent Rural | 1.06 (1.04-1.09) | 1.09 (1.04-1.14) | 1.09 (1.00-1.18) | 1.56 (1.47-1.66) | 1.35 (1.28-1.43) |
| Number of Visit Before Acute COVID-19 | 1.00 (1.00-1.00) | 1.00 (1.00-1.00) | 1.00 (1.00-1.00) | 1.00 (1.00-1.00) | 1.00 (1.00-1.00) |

**eTable 15. Sensitivity Analysis 4: Multivariable Regression for Adverse Acute COVID-19 Events with COVID-19 Therapies Individually During the Time Periods they were Widely in Use by Rurality**

**A. Nirmatrelvir/Ritonavir**

| **Characteristic** | **Nirmatrelvir/Ritonavir, Available after December 22, 2021** | | | | |
| --- | --- | --- | --- | --- | --- |
|  | **Hospitalization** | **AKI/Dialysis** | **MACE** | **IMV/ECMO** | **Death** |
|  | **Adjusted Odds Ratio**  **(95% CI)** | **Adjusted Hazard Ratio**  **(95% CI)** | **Adjusted Hazard Ratio**  **(95% CI)** | **Adjusted Hazard Ratio**  **(95% CI)** | **Adjusted Hazard Ratio**  **(95% CI)** |
| Sex |  |  |  |  |  |
| Female | Reference | Reference | Reference | Reference | Reference |
| Male | 1.16 (1.14-1.17) | 1.39 (1.35-1.42) | 1.41 (1.35-1.47) | 1.62 (1.55-1.70) | 1.31 (1.26-1.36) |
| Age at COVID-19 Diagnosis | 1.04 (1.03-1.04) | 1.01 (1.01-1.02) | 1.03 (1.02-1.03) | 1.00 (1.00-1.00) | 1.04 (1.03-1.04) |
| Race/Ethnicity |  |  |  |  |  |
| White Non-Hispanic | Reference | Reference | Reference | Reference | Reference |
| Black or African American Non-Hispanic | 1.61 (1.59-1.64) | 1.53 (1.48-1.57) | 1.24 (1.17-1.32) | 1.07 (1.01-1.14) | 0.91 (0.86-0.96) |
| Hispanic or Latino Any Race | 1.66 (1.62-1.69) | 1.09 (1.05-1.14) | 1.01 (0.92-1.10) | 1.05 (0.97-1.13) | 1.02 (0.95-1.10) |
| Other | 0.91 (0.88-0.94) | 1.25 (1.17-1.33) | 1.28 (1.14-1.44) | 1.31 (1.17-1.46) | 1.12 (1.01-1.25) |
| Missing/Unknown | 0.95 (0.93-0.98) | 1.16 (1.09-1.23) | 1.34 (1.21-1.49) | 1.34 (1.22-1.48) | 1.13 (1.03-1.25) |
| Myocardial infarction (MI) | 1.21 (1.17-1.24) | 1.03 (1.00-1.07) | 2.69 (2.53-2.85) | 1.11 (1.03-1.20) | 1.09 (1.03-1.16) |
| Congestive heart failure (CHF) | 1.83 (1.79-1.87) | 1.06 (1.03-1.09) | 1.20 (1.13-1.27) | 1.12 (1.05-1.19) | 1.18 (1.12-1.24) |
| Peripheral vascular disease (PVD) | 1.08 (1.06-1.11) | 0.98 (0.94-1.01) | 1.07 (1.00-1.14) | 0.92 (0.85-0.99) | 1.05 (0.98-1.11) |
| Cerebrovascular disease (CVD) | 1.01 (0.98-1.03) | 0.95 (0.92-0.98) | 1.20 (1.12-1.28) | 0.99 (0.91-1.07) | 0.95 (0.89-1.01) |
| Dementia | 1.61 (1.56-1.66) | 0.84 (0.80-0.87) | 0.64 (0.59-0.70) | 0.65 (0.58-0.73) | 1.24 (1.16-1.32) |
| Chronic pulmonary disease (CPD) | 1.21 (1.19-1.23) | 0.92 (0.90-0.95) | 0.89 (0.84-0.94) | 1.09 (1.03-1.16) | 1.08 (1.03-1.13) |
| Rheumatologic disease (RD) | 0.93 (0.91-0.95) | 1.12 (1.08-1.15) | 0.96 (0.90-1.03) | 1.10 (1.02-1.18) | 1.03 (0.97-1.10) |
| Peptic ulcer disease (PUD) | 1.10 (1.06-1.15) | 1.14 (1.08-1.20) | 0.93 (0.82-1.05) | 1.01 (0.89-1.14) | 0.99 (0.90-1.10) |
| Liver Disease |  |  |  |  |  |
| No History of Liver Disease | Reference | Reference | Reference | Reference | Reference |
| Mild Liver Disease | 0.99 (0.97-1.02) | 1.01 (0.96-1.05) | 0.92 (0.84-1.01) | 1.05 (0.96-1.14) | 1.02 (0.94-1.11) |
| Moderate to Severe Liver Disease | 1.75 (1.68-1.83) | 1.32 (1.26-1.40) | 0.86 (0.75-0.99) | 1.49 (1.34-1.65) | 1.81 (1.65-1.98) |
| Diabetes Mellitus |  |  |  |  |  |
| No History of Diabetes | Reference | Reference | Reference | Reference | Reference |
| Diabetes without Chronic Complications | 1.35 (1.32-1.38) | 1.06 (1.01-1.10) | 1.00 (0.92-1.08) | 0.94 (0.87-1.02) | 0.98 (0.91-1.05) |
| Diabetes with Chronic Complications | 1.34 (1.31-1.37) | 1.26 (1.22-1.30) | 1.16 (1.09-1.23) | 1.16 (1.08-1.24) | 1.03 (0.98-1.09) |
| Hemiplegia or paraplegia | 1.98 (1.90-2.07) | 0.92 (0.87-0.98) | 0.87 (0.78-0.97) | 1.07 (0.95-1.21) | 1.12 (1.01-1.23) |
| Renal Disease | 1.86 (1.82-1.89) | 2.47 (2.40-2.54) | 0.97 (0.91-1.03) | 1.07 (1.01-1.13) | 1.18 (1.12-1.24) |
| Cancer |  |  |  |  |  |
| No History of Malignant Cancer | Reference | Reference | Reference | Reference | Reference |
| Any Malignancy Except Neoplasm of skin | 1.01 (0.99-1.03) | 0.94 (0.91-0.97) | 0.79 (0.74-0.85) | 0.95 (0.89-1.02) | 1.23 (1.17-1.30) |
| Metastatic Solid Tumor | 2.10 (2.02-2.18) | 0.98 (0.93-1.04) | 0.70 (0.61-0.80) | 0.90 (0.80-1.03) | 2.55 (2.37-2.74) |
| HIV | 1.12 (1.04-1.20) | 0.95 (0.84-1.06) | 0.89 (0.68-1.17) | 0.82 (0.64-1.04) | 1.00 (0.78-1.28) |
| Obesity | 0.85 (0.84-0.86) | 1.05 (1.02-1.08) | 0.95 (0.91-1.00) | 1.04 (0.99-1.09) | 0.92 (0.88-0.96) |
| Hypertension | 0.83 (0.82-0.84) | 1.09 (1.06-1.13) | 1.03 (0.97-1.09) | 0.84 (0.79-0.89) | 0.81 (0.77-0.85) |
| Former or Current Tobacco User | 1.75 (1.71-1.79) | 1.12 (1.08-1.16) | 1.28 (1.20-1.37) | 1.07 (1.00-1.14) | 0.99 (0.92-1.06) |
| History of Substance Abuse Disorder | 2.89 (2.81-2.96) | 1.02 (0.98-1.06) | 0.92 (0.84-1.00) | 1.25 (1.16-1.35) | 1.02 (0.94-1.11) |
| Vaccination Status Prior to SARS-CoV-2 Infection |  |  |  |  |  |
| No Documented COVID-19 Vaccination | Reference | Reference | Reference | Reference | Reference |
| Primary Vaccination Series | 0.71 (0.69-0.72) | 0.82 (0.79-0.85) | 0.75 (0.70-0.80) | 0.73 (0.68-0.78) | 0.79 (0.75-0.84) |
| Primary+ Vaccination Series | 0.57 (0.56-0.58) | 0.73 (0.71-0.76) | 0.68 (0.64-0.73) | 0.65 (0.60-0.70) | 0.61 (0.58-0.65) |
| Census Region |  |  |  |  |  |
| Midwest | Reference | Reference | Reference | Reference | Reference |
| Northeast | 1.33 (1.31-1.36) | 1.13 (1.09-1.17) | 1.17 (1.09-1.25) | 0.88 (0.82-0.95) | 0.80 (0.75-0.84) |
| South | 1.33 (1.31-1.35) | 1.30 (1.27-1.34) | 1.43 (1.35-1.51) | 1.48 (1.40-1.56) | 1.04 (0.99-1.09) |
| West | 0.87 (0.85-0.88) | 1.38 (1.33-1.43) | 1.16 (1.08-1.26) | 1.59 (1.49-1.71) | 0.95 (0.89-1.01) |
| Rurality |  |  |  |  |  |
| Urban | Reference | Reference | Reference | Reference | Reference |
| Urban-Adjacent Rural | 1.14 (1.12-1.16) | 1.01 (0.98-1.05) | 1.05 (0.98-1.12) | 1.17 (1.09-1.24) | 1.25 (1.18-1.32) |
| Nonurban-Adjacent Rural | 1.14 (1.10-1.18) | 1.03 (0.96-1.10) | 1.19 (1.07-1.33) | 1.37 (1.23-1.53) | 1.28 (1.17-1.40) |
| Nirmatrelvir/ritonavir | 0.08 (0.08-0.09) | 0.36 (0.30-0.44) | 0.57 (0.42-0.76) | 0.21 (0.13-0.33) | 0.44 (0.33-0.58) |

**B. Molnupiravir**

| **Characteristic** | **Molnupiravir, Available after December 23, 2021** | | | | |
| --- | --- | --- | --- | --- | --- |
|  | **Hospitalization** | **AKI/Dialysis** | **MACE** | **IMV/ECMO** | **Death** |
|  | **Adjusted Odds Ratio**  **(95% CI)** | **Adjusted Hazard Ratio**  **(95% CI)** | **Adjusted Hazard Ratio**  **(95% CI)** | **Adjusted Hazard Ratio**  **(95% CI)** | **Adjusted Hazard Ratio**  **(95% CI)** |
| Sex |  |  |  |  |  |
| Female | Reference | Reference | Reference | Reference | Reference |
| Male | 1.16 (1.14-1.17) | 1.39 (1.36-1.42) | 1.41 (1.35-1.48) | 1.62 (1.55-1.70) | 1.31 (1.26-1.36) |
| Age at COVID-19 Diagnosis | 1.03 (1.03-1.04) | 1.01 (1.01-1.02) | 1.03 (1.02-1.03) | 1.00 (1.00-1.00) | 1.04 (1.03-1.04) |
| Race/Ethnicity |  |  |  |  |  |
| White Non-Hispanic | Reference | Reference | Reference | Reference | Reference |
| Black or African American Non-Hispanic | 1.63 (1.61-1.66) | 1.53 (1.49-1.57) | 1.24 (1.17-1.32) | 1.07 (1.01-1.14) | 0.91 (0.86-0.96) |
| Hispanic or Latino Any Race | 1.67 (1.64-1.71) | 1.10 (1.05-1.14) | 1.01 (0.92-1.10) | 1.05 (0.97-1.13) | 1.02 (0.95-1.10) |
| Other | 0.92 (0.89-0.95) | 1.24 (1.17-1.32) | 1.27 (1.13-1.43) | 1.30 (1.16-1.45) | 1.12 (1.01-1.24) |
| Missing/Unknown | 0.96 (0.93-0.98) | 1.16 (1.09-1.23) | 1.34 (1.21-1.48) | 1.34 (1.21-1.48) | 1.13 (1.03-1.25) |
| Myocardial infarction (MI) | 1.22 (1.19-1.25) | 1.03 (1.00-1.07) | 2.69 (2.54-2.85) | 1.11 (1.03-1.20) | 1.09 (1.03-1.16) |
| Congestive heart failure (CHF) | 1.88 (1.84-1.91) | 1.06 (1.03-1.10) | 1.20 (1.13-1.27) | 1.12 (1.05-1.20) | 1.18 (1.12-1.24) |
| Peripheral vascular disease (PVD) | 1.09 (1.07-1.12) | 0.98 (0.94-1.01) | 1.07 (1.00-1.14) | 0.92 (0.85-0.99) | 1.05 (0.98-1.11) |
| Cerebrovascular disease (CVD) | 1.01 (0.99-1.04) | 0.95 (0.92-0.98) | 1.19 (1.12-1.28) | 0.99 (0.91-1.07) | 0.95 (0.89-1.01) |
| Dementia | 1.63 (1.58-1.68) | 0.84 (0.80-0.88) | 0.64 (0.59-0.70) | 0.65 (0.58-0.73) | 1.24 (1.16-1.32) |
| Chronic pulmonary disease (CPD) | 1.20 (1.19-1.22) | 0.92 (0.90-0.95) | 0.89 (0.84-0.94) | 1.09 (1.03-1.15) | 1.07 (1.02-1.13) |
| Rheumatologic disease (RD) | 0.93 (0.91-0.95) | 1.12 (1.08-1.15) | 0.96 (0.90-1.03) | 1.10 (1.02-1.18) | 1.03 (0.97-1.10) |
| Peptic ulcer disease (PUD) | 1.09 (1.05-1.14) | 1.13 (1.07-1.19) | 0.93 (0.82-1.04) | 1.00 (0.89-1.13) | 0.99 (0.90-1.10) |
| Liver Disease |  |  |  |  |  |
| No History of Liver Disease | Reference | Reference | Reference | Reference | Reference |
| Mild Liver Disease | 0.99 (0.96-1.01) | 1.00 (0.96-1.05) | 0.92 (0.84-1.01) | 1.04 (0.96-1.14) | 1.02 (0.94-1.11) |
| Moderate to Severe Liver Disease | 1.78 (1.70-1.85) | 1.33 (1.26-1.40) | 0.86 (0.75-0.99) | 1.49 (1.34-1.65) | 1.81 (1.66-1.99) |
| Diabetes Mellitus |  |  |  |  |  |
| No History of Diabetes | Reference | Reference | Reference | Reference | Reference |
| Diabetes without Chronic Complications | 1.35 (1.32-1.38) | 1.06 (1.01-1.10) | 1.00 (0.92-1.08) | 0.94 (0.87-1.02) | 0.98 (0.91-1.05) |
| Diabetes with Chronic Complications | 1.36 (1.33-1.39) | 1.26 (1.22-1.30) | 1.16 (1.09-1.24) | 1.16 (1.08-1.24) | 1.04 (0.98-1.10) |
| Hemiplegia or paraplegia | 2.00 (1.92-2.09) | 0.92 (0.87-0.98) | 0.87 (0.78-0.97) | 1.07 (0.95-1.21) | 1.12 (1.01-1.24) |
| Renal Disease | 1.93 (1.90-1.96) | 2.48 (2.41-2.55) | 0.97 (0.92-1.03) | 1.07 (1.01-1.14) | 1.18 (1.13-1.24) |
| Cancer |  |  |  |  |  |
| No History of Malignant Cancer | Reference | Reference | Reference | Reference | Reference |
| Any Malignancy Except Neoplasm of skin | 1.00 (0.98-1.02) | 0.93 (0.91-0.97) | 0.79 (0.74-0.85) | 0.95 (0.89-1.02) | 1.23 (1.17-1.29) |
| Metastatic Solid Tumor | 2.08 (2.00-2.16) | 0.98 (0.92-1.04) | 0.70 (0.61-0.80) | 0.90 (0.79-1.02) | 2.55 (2.37-2.74) |
| HIV | 1.09 (1.02-1.18) | 0.94 (0.84-1.06) | 0.90 (0.68-1.17) | 0.81 (0.64-1.04) | 1.00 (0.77-1.28) |
| Obesity | 0.83 (0.82-0.84) | 1.05 (1.02-1.07) | 0.95 (0.90-1.00) | 1.03 (0.98-1.09) | 0.92 (0.88-0.96) |
| Hypertension | 0.82 (0.81-0.84) | 1.09 (1.06-1.13) | 1.03 (0.97-1.09) | 0.84 (0.79-0.89) | 0.81 (0.77-0.85) |
| Former or Current Tobacco User | 1.72 (1.69-1.76) | 1.12 (1.08-1.16) | 1.28 (1.20-1.37) | 1.07 (1.00-1.14) | 0.99 (0.93-1.06) |
| History of Substance Abuse Disorder | 2.90 (2.82-2.97) | 1.02 (0.98-1.06) | 0.92 (0.84-1.00) | 1.25 (1.17-1.35) | 1.02 (0.94-1.11) |
| Vaccination Status Prior to SARS-CoV-2 Infection |  |  |  |  |  |
| No Documented COVID-19 Vaccination | Reference | Reference | Reference | Reference | Reference |
| Primary Vaccination Series | 0.69 (0.67-0.70) | 0.82 (0.79-0.85) | 0.75 (0.70-0.80) | 0.72 (0.67-0.77) | 0.79 (0.75-0.84) |
| Primary+ Vaccination Series | 0.51 (0.50-0.52) | 0.73 (0.70-0.75) | 0.68 (0.63-0.73) | 0.64 (0.59-0.69) | 0.61 (0.57-0.65) |
| Census Region |  |  |  |  |  |
| Midwest | Reference | Reference | Reference | Reference | Reference |
| Northeast | 1.30 (1.27-1.32) | 1.13 (1.09-1.17) | 1.17 (1.09-1.25) | 0.89 (0.82-0.95) | 0.80 (0.75-0.85) |
| South | 1.28 (1.27-1.30) | 1.30 (1.27-1.34) | 1.43 (1.35-1.51) | 1.47 (1.40-1.56) | 1.03 (0.99-1.08) |
| West | 0.81 (0.80-0.83) | 1.37 (1.32-1.43) | 1.16 (1.07-1.25) | 1.59 (1.48-1.70) | 0.94 (0.89-1.01) |
| Rurality |  |  |  |  |  |
| Urban | Reference | Reference | Reference | Reference | Reference |
| Urban-Adjacent Rural | 1.16 (1.14-1.18) | 1.01 (0.98-1.05) | 1.05 (0.99-1.13) | 1.17 (1.10-1.25) | 1.25 (1.18-1.32) |
| Nonurban-Adjacent Rural | 1.17 (1.13-1.21) | 1.03 (0.97-1.10) | 1.19 (1.07-1.33) | 1.38 (1.24-1.53) | 1.28 (1.17-1.41) |
| Molnupiravir | 0.09 (0.07-0.10) | 0.66 (0.52-0.84) | 0.74 (0.47-1.18) | 0.87 (0.55-1.38) | 0.79 (0.53-1.17) |

**C. Monoclonal Antibody Therapies**

| **Characteristic** | **Monoclonal Antibodies, Available from November 10, 2020 – January 26, 2023** | | | | |
| --- | --- | --- | --- | --- | --- |
|  | **Hospitalization** | **AKI/Dialysis** | **MACE** | **IMV/ECMO** | **Death** |
|  | **Adjusted Odds Ratio**  **(95% CI)** | **Adjusted Hazard Ratio**  **(95% CI)** | **Adjusted Hazard Ratio**  **(95% CI)** | **Adjusted Hazard Ratio**  **(95% CI)** | **Adjusted Hazard Ratio**  **(95% CI)** |
| Sex |  |  |  |  |  |
| Female | Reference | Reference | Reference | Reference | Reference |
| Male | 1.23 (1.22-1.24) | 1.46 (1.43-1.48) | 1.41 (1.37-1.46) | 1.52 (1.48-1.56) | 1.36 (1.33-1.39) |
| Age at COVID-19 Diagnosis | 1.04 (1.04-1.04) | 1.02 (1.02-1.02) | 1.03 (1.03-1.03) | 1.00 (1.00-1.00) | 1.04 (1.04-1.04) |
| Race/Ethnicity |  |  |  |  |  |
| White Non-Hispanic | Reference | Reference | Reference | Reference | Reference |
| Black or African American Non-Hispanic | 1.72 (1.70-1.74) | 1.63 (1.60-1.67) | 1.25 (1.19-1.30) | 1.04 (1.00-1.08) | 0.95 (0.91-0.98) |
| Hispanic or Latino Any Race | 1.86 (1.83-1.88) | 1.09 (1.06-1.12) | 0.96 (0.90-1.02) | 1.12 (1.08-1.18) | 1.12 (1.07-1.17) |
| Other | 0.97 (0.95-1.00) | 1.24 (1.19-1.30) | 1.29 (1.19-1.40) | 1.28 (1.20-1.37) | 1.18 (1.10-1.26) |
| Missing/Unknown | 0.95 (0.93-0.97) | 1.21 (1.16-1.26) | 1.33 (1.24-1.43) | 1.36 (1.28-1.44) | 1.29 (1.21-1.36) |
| Myocardial infarction (MI) | 1.19 (1.17-1.21) | 1.02 (0.99-1.04) | 2.74 (2.62-2.86) | 1.03 (0.98-1.09) | 1.05 (1.01-1.10) |
| Congestive heart failure (CHF) | 1.68 (1.66-1.71) | 1.06 (1.03-1.08) | 1.22 (1.16-1.27) | 1.02 (0.98-1.07) | 1.12 (1.08-1.16) |
| Peripheral vascular disease (PVD) | 1.09 (1.07-1.11) | 0.98 (0.95-1.00) | 1.05 (1.0-1.10) | 0.96 (0.91-1.02) | 1.03 (0.99-1.07) |
| Cerebrovascular disease (CVD) | 0.97 (0.95-0.99) | 0.95 (0.93-0.98) | 1.15 (1.09-1.21) | 0.97 (0.91-1.02) | 0.93 (0.90-0.98) |
| Dementia | 1.41 (1.38-1.45) | 0.83 (0.80-0.86) | 0.71 (0.66-0.76) | 0.56 (0.52-0.61) | 1.15 (1.10-1.21) |
| Chronic pulmonary disease (CPD) | 1.18 (1.16-1.19) | 0.92 (0.90-0.94) | 0.89 (0.85-0.92) | 1.05 (1.01-1.09) | 1.06 (1.02-1.09) |
| Rheumatologic disease (RD) | 0.97 (0.95-0.99) | 1.11 (1.09-1.14) | 1.01 (0.96-1.06) | 1.10 (1.04-1.15) | 1.03 (0.99-1.07) |
| Peptic ulcer disease (PUD) | 1.08 (1.04-1.11) | 1.07 (1.02-1.11) | 0.95 (0.86-1.04) | 1.03 (0.95-1.13) | 0.96 (0.90-1.04) |
| Liver Disease |  |  |  |  |  |
| No History of Liver Disease | Reference | Reference | Reference | Reference | Reference |
| Mild Liver Disease | 0.99 (0.97-1.01) | 1.03 (0.99-1.06) | 0.91 (0.84-0.97) | 1.07 (1.01-1.13) | 1.02 (0.96-1.07) |
| Moderate to Severe Liver Disease | 1.71 (1.65-1.77) | 1.36 (1.31-1.42) | 0.89 (0.80-0.99) | 1.38 (1.28-1.49) | 1.67 (1.56-1.79) |
| Diabetes Mellitus |  |  |  |  |  |
| No History of Diabetes | Reference | Reference | Reference | Reference | Reference |
| Diabetes without Chronic Complications | 1.40 (1.38-1.42) | 1.06 (1.03-1.09) | 1.02 (0.97-1.09) | 1.04 (0.99-1.09) | 1.03 (0.99-1.08) |
| Diabetes with Chronic Complications | 1.39 (1.37-1.41) | 1.26 (1.23-1.29) | 1.15 (1.09-1.20) | 1.21 (1.16-1.26) | 1.10 (1.06-1.14) |
| Hemiplegia or paraplegia | 1.87 (1.80-1.93) | 0.94 (0.90-0.98) | 0.92 (0.84-1.00) | 0.98 (0.89-1.07) | 1.08 (1.01-1.17) |
| Renal Disease | 1.81 (1.78-1.83) | 2.35 (2.30-2.40) | 1.04 (1.00-1.09) | 1.03 (0.99-1.08) | 1.19 (1.16-1.23) |
| Cancer |  |  |  |  |  |
| No History of Malignant Cancer | Reference | Reference | Reference | Reference | Reference |
| Any Malignancy Except Neoplasm of skin | 0.98 (0.97-1.00) | 0.93 (0.91-0.95) | 0.81 (0.77-0.86) | 0.86 (0.82-0.91) | 1.10 (1.06-1.14) |
| Metastatic Solid Tumor | 1.91 (1.85-1.97) | 0.93 (0.88-0.97) | 0.79 (0.71-0.87) | 0.85 (0.77-0.93) | 2.05 (1.94-2.17) |
| HIV | 1.03 (0.97-1.09) | 0.95 (0.87-1.03) | 0.96 (0.79-1.17) | 0.88 (0.74-1.04) | 1.02 (0.86-1.20) |
| Obesity | 0.98 (0.97-0.99) | 1.05 (1.03-1.07) | 0.95 (0.91-0.98) | 1.09 (1.06-1.13) | 1.00 (0.97-1.03) |
| Hypertension | 0.84 (0.83-0.85) | 1.14 (1.11-1.16) | 1.06 (1.02-1.11) | 0.88 (0.85-0.92) | 0.84 (0.82-0.87) |
| Former or Current Tobacco User | 1.53 (1.50-1.55) | 1.08 (1.05-1.11) | 1.22 (1.16-1.29) | 0.93 (0.89-0.98) | 0.94 (0.90-0.99) |
| History of Substance Abuse Disorder | 2.58 (2.53-2.63) | 0.99 (0.96-1.03) | 0.99 (0.92-1.06) | 1.12 (1.06-1.17) | 0.95 (0.90-1.00) |
| Vaccination Status Prior to SARS-CoV-2 Infection |  |  |  |  |  |
| No Documented COVID-19 Vaccination | Reference | Reference | Reference | Reference | Reference |
| Primary Vaccination Series | 0.50 (0.49-0.51) | 0.80 (0.78-0.82) | 0.76 (0.71-0.80) | 0.61 (0.58-0.65) | 0.69 (0.66-0.72) |
| Primary+ Vaccination Series | 0.34 (0.34-0.35) | 0.72 (0.69-0.74) | 0.73 (0.68-0.78) | 0.52 (0.48-0.56) | 0.49 (0.46-0.51) |
| Census Region |  |  |  |  |  |
| Midwest | Reference | Reference | Reference | Reference | Reference |
| Northeast | 1.14 (1.13-1.16) | 1.34 (1.31-1.38) | 1.68 (1.60-1.77) | 1.13 (1.08-1.19) | 0.85 (0.82-0.88) |
| South | 1.25 (1.24-1.27) | 1.54 (1.51-1.57) | 1.74 (1.67-1.81) | 2.13 (2.06-2.20) | 1.15 (1.12-1.19) |
| West | 0.85 (0.84-0.87) | 1.61 (1.57-1.65) | 1.44 (1.36-1.53) | 2.50 (2.40-2.61) | 1.11 (1.07-1.16) |
| Rurality |  |  |  |  |  |
| Urban | Reference | Reference | Reference | Reference | Reference |
| Urban-Adjacent Rural | 1.08 (1.06-1.09) | 1.05 (1.03-1.08) | 1.09 (1.04-1.14) | 1.35 (1.30-1.40) | 1.30 (1.25-1.34) |
| Nonurban-Adjacent Rural | 1.07 (1.05-1.10) | 1.09 (1.05-1.14) | 1.07 (0.98-1.16) | 1.57 (1.48-1.67) | 1.38 (1.30-1.46) |
| Nirmatrelvir/ritonavir |  |  |  |  |  |
| Molnupiravir |  |  |  |  |  |
| Monoclonal Therapies | 0.12 (0.11-0.12) | 0.54 (0.51-0.58) | 0.45 (0.39-0.52) | 0.45 (0.40-0.50) | 0.56 (0.51-0.61) |

**D. Remdesivir**

| **Characteristic** | **Remdesivir, Available After May 1, 2020** | | | | |
| --- | --- | --- | --- | --- | --- |
|  | **Hospitalization** | **AKI/Dialysis** | **MACE** | **IMV/ECMO** | **Death** |
|  | **Adjusted Odds Ratio**  **(95% CI)** | **Adjusted Hazard Ratio**  **(95% CI)** | **Adjusted Hazard Ratio**  **(95% CI)** | **Adjusted Hazard Ratio**  **(95% CI)** | **Adjusted Hazard Ratio**  **(95% CI)** |
| Sex |  |  |  |  |  |
| Female | N/A | Reference | Reference | Reference | Reference |
| Male | N/A | 1.47 (1.45-1.50) | 1.44 (1.40-1.49) | 1.56 (1.51-1.60) | 1.35 (1.32-1.38) |
| Age at COVID-19 Diagnosis | N/A | 1.02 (1.02-1.02) | 1.03 (1.03-1.03) | 1.00 (1.00-1.00) | 1.04 (1.04-1.04) |
| Race/Ethnicity |  |  |  |  |  |
| White Non-Hispanic | N/A | Reference | Reference | Reference | Reference |
| Black or African American Non-Hispanic | N/A | 1.63 (1.60-1.66) | 1.28 (1.23-1.34) | 1.06 (1.02-1.09) | 0.94 (0.91-0.97) |
| Hispanic or Latino Any Race | N/A | 1.10 (1.07-1.14) | 1.0 (0.94-1.06) | 1.14 (1.09-1.19) | 1.11 (1.07-1.16) |
| Other | N/A | 1.23 (1.18-1.29) | 1.31 (1.20-1.42) | 1.28 (1.19-1.37) | 1.17 (1.10-1.25) |
| Missing/Unknown | N/A | 1.20 (1.16-1.25) | 1.32 (1.23-1.42) | 1.36 (1.28-1.44) | 1.29 (1.21-1.36) |
| Myocardial infarction (MI) | N/A | 1.00 (0.98-1.03) | 2.67 (2.55-2.79) | 1.03 (0.98-1.09) | 1.05 (1.01-1.10) |
| Congestive heart failure (CHF) | N/A | 1.05 (1.03-1.08) | 1.21 (1.16-1.26) | 1.03 (0.99-1.08) | 1.13 (1.09-1.17) |
| Peripheral vascular disease (PVD) | N/A | 0.97 (0.95-1.00) | 1.04 (0.99-1.10) | 0.96 (0.91-1.01) | 1.03 (0.99-1.08) |
| Cerebrovascular disease (CVD) | N/A | 0.97 (0.94-0.99) | 1.17 (1.11-1.23) | 0.97 (0.92-1.02) | 0.93 (0.89-0.97) |
| Dementia | N/A | 0.83 (0.80-0.85) | 0.68 (0.64-0.73) | 0.57 (0.52-0.62) | 1.16 (1.11-1.21) |
| Chronic pulmonary disease (CPD) | N/A | 0.97 (0.95-0.99) | 0.95 (0.91-0.99) | 1.08 (1.04-1.12) | 1.05 (1.01-1.08) |
| Rheumatologic disease (RD) | N/A | 1.13 (1.10-1.16) | 1.03 (0.98-1.08) | 1.10 (1.05-1.16) | 1.01 (0.97-1.05) |
| Peptic ulcer disease (PUD) | N/A | 1.07 (1.03-1.12) | 0.95 (0.87-1.04) | 1.03 (0.95-1.13) | 0.96 (0.89-1.03) |
| Liver Disease |  |  |  |  |  |
| No History of Liver Disease | N/A | Reference | Reference | Reference | Reference |
| Mild Liver Disease | N/A | 1.03 (1.0-1.06) | 0.92 (0.86-0.98) | 1.07 (1.01-1.13) | 1.01 (0.96-1.07) |
| Moderate to Severe Liver Disease | N/A | 1.33 (1.27-1.38) | 0.84 (0.76-0.94) | 1.36 (1.26-1.46) | 1.69 (1.58-1.80) |
| Diabetes Mellitus |  |  |  |  |  |
| No History of Diabetes | N/A | Reference | Reference | Reference | Reference |
| Diabetes without Chronic Complications | N/A | 1.07 (1.04-1.10) | 1.03 (0.97-1.09) | 1.04 (0.99-1.09) | 1.03 (0.99-1.08) |
| Diabetes with Chronic Complications | N/A | 1.24 (1.21-1.26) | 1.13 (1.08-1.19) | 1.20 (1.15-1.25) | 1.09 (1.06-1.14) |
| Hemiplegia or paraplegia | N/A | 0.94 (0.90-0.98) | 0.90 (0.83-0.98) | 0.98 (0.89-1.07) | 1.08 (1.01-1.16) |
| Renal Disease | N/A | 2.27 (2.23-2.32) | 1.02 (0.98-1.07) | 1.02 (0.98-1.06) | 1.19 (1.15-1.23) |
| Cancer |  |  |  |  |  |
| No History of Malignant Cancer | N/A | Reference | Reference | Reference | Reference |
| Any Malignancy Except Neoplasm of skin | N/A | 0.93 (0.91-0.95) | 0.81 (0.77-0.86) | 0.86 (0.82-0.90) | 1.10 (1.06-1.14) |
| Metastatic Solid Tumor | N/A | 0.94 (0.90-0.99) | 0.77 (0.69-0.85) | 0.84 (0.77-0.92) | 2.04 (1.93-2.15) |
| HIV | N/A | 0.95 (0.87-1.03) | 0.93 (0.76-1.13) | 0.87 (0.74-1.03) | 1.00 (0.85-1.19) |
| Obesity | N/A | 1.12 (1.10-1.14) | 1.02 (0.98-1.06) | 1.13 (1.09-1.16) | 0.97 (0.95-1.00) |
| Hypertension | N/A | 1.14 (1.12-1.17) | 1.08 (1.03-1.12) | 0.88 (0.85-0.91) | 0.84 (0.82-0.87) |
| Former or Current Tobacco User | N/A | 1.09 (1.06-1.12) | 1.25 (1.19-1.32) | 0.95 (0.91-1.00) | 0.95 (0.90-0.99) |
| History of Substance Abuse Disorder | N/A | 0.96 (0.93-0.99) | 0.91 (0.85-0.97) | 1.08 (1.03-1.14) | 0.97 (0.92-1.03) |
| Vaccination Status Prior to SARS-CoV-2 Infection |  |  |  |  |  |
| No Documented COVID-19 Vaccination | N/A | Reference | Reference | Reference | Reference |
| Primary Vaccination Series | N/A | 0.78 (0.76-0.80) | 0.75 (0.71-0.80) | 0.60 (0.57-0.63) | 0.68 (0.66-0.71) |
| Primary+ Vaccination Series | N/A | 0.71 (0.69-0.74) | 0.71 (0.67-0.76) | 0.51 (0.48-0.55) | 0.49 (0.46-0.52) |
| Census Region |  |  |  |  |  |
| Midwest | N/A | Reference | Reference | Reference | Reference |
| Northeast | N/A | 1.37 (1.34-1.41) | 1.71 (1.63-1.79) | 1.13 (1.08-1.19) | 0.85 (0.82-0.88) |
| South | N/A | 1.75 (1.71-1.78) | 2.06 (1.98-2.15) | 2.23 (2.16-2.31) | 1.10 (1.07-1.13) |
| West | N/A | 1.69 (1.64-1.73) | 1.54 (1.46-1.63) | 2.54 (2.44-2.65) | 1.08 (1.04-1.12) |
| Rurality |  |  |  |  |  |
| Urban | N/A | Reference | Reference | Reference | Reference |
| Urban-Adjacent Rural | N/A | 1.01 (0.99-1.04) | 1.01 (0.97-1.06) | 1.32 (1.27-1.37) | 1.31 (1.26-1.35) |
| Nonurban-Adjacent Rural | N/A | 1.07 (1.03-1.12) | 1.05 (0.97-1.14) | 1.57 (1.47-1.67) | 1.37 (1.29-1.45) |
| Remdesivir | N/A | 0.29 (0.28-0.30) | 0.21 (0.20-0.22) | 0.63 (0.61-0.65) | 1.23 (1.20-1.26) |

**E. Anticoagulants**

| **Characteristic** | **Anticoagulants, All Time Periods** | | | | |
| --- | --- | --- | --- | --- | --- |
|  | **Hospitalization** | **AKI/Dialysis** | **MACE** | **IMV/ECMO** | **Death** |
|  | **Adjusted Odds Ratio**  **(95% CI)** | **Adjusted Hazard Ratio**  **(95% CI)** | **Adjusted Hazard Ratio**  **(95% CI)** | **Adjusted Hazard Ratio**  **(95% CI)** | **Adjusted Hazard Ratio**  **(95% CI)** |
| Sex |  |  |  |  |  |
| Female | N/A | Reference | Reference | Reference | Reference |
| Male | N/A | 1.48 (1.46-1.51) | 1.46 (1.42-1.51) | 1.61 (1.57-1.65) | 1.36 (1.33-1.40) |
| Age at COVID-19 Diagnosis | N/A | 1.02 (1.02-1.02) | 1.03 (1.03-1.03) | 1.00 (1.00-1.01) | 1.04 (1.04-1.04) |
| Race/Ethnicity |  |  |  |  |  |
| White Non-Hispanic | N/A | Reference | Reference | Reference | Reference |
| Black or African American Non-Hispanic | N/A | 1.65 (1.62-1.68) | 1.31 (1.26-1.37) | 1.08 (1.04-1.12) | 0.95 (0.92-0.98) |
| Hispanic or Latino Any Race | N/A | 1.13 (1.10-1.17) | 1.02 (0.97-1.09) | 1.16 (1.11-1.21) | 1.13 (1.08-1.18) |
| Other | N/A | 1.22 (1.17-1.27) | 1.29 (1.19-1.40) | 1.26 (1.18-1.35) | 1.18 (1.10-1.25) |
| Missing/Unknown | N/A | 1.25 (1.20-1.30) | 1.38 (1.28-1.48) | 1.39 (1.31-1.47) | 1.29 (1.22-1.37) |
| Myocardial infarction (MI) | N/A | 0.99 (0.97-1.02) | 2.60 (2.49-2.72) | 1.02 (0.96-1.07) | 1.05 (1.01-1.09) |
| Congestive heart failure (CHF) | N/A | 0.97 (0.95-0.99) | 1.11 (1.06-1.16) | 0.97 (0.93-1.02) | 1.12 (1.08-1.16) |
| Peripheral vascular disease (PVD) | N/A | 0.96 (0.93-0.98) | 1.03 (0.98-1.08) | 0.94 (0.89-0.99) | 1.03 (0.99-1.07) |
| Cerebrovascular disease (CVD) | N/A | 0.94 (0.92-0.97) | 1.14 (1.09-1.20) | 0.95 (0.90-1.01) | 0.93 (0.90-0.97) |
| Dementia | N/A | 0.82 (0.79-0.85) | 0.67 (0.63-0.72) | 0.56 (0.51-0.61) | 1.16 (1.11-1.21) |
| Chronic pulmonary disease (CPD) | N/A | 0.94 (0.92-0.96) | 0.90 (0.86-0.93) | 1.06 (1.02-1.10) | 1.06 (1.03-1.09) |
| Rheumatologic disease (RD) | N/A | 1.11 (1.08-1.13) | 1.00 (0.95-1.05) | 1.10 (1.05-1.15) | 1.02 (0.98-1.06) |
| Peptic ulcer disease (PUD) | N/A | 1.02 (0.98-1.06) | 0.91 (0.83-1.00) | 1.01 (0.93-1.10) | 0.96 (0.89-1.03) |
| Liver Disease |  |  |  |  |  |
| No History of Liver Disease | N/A | Reference | Reference | Reference | Reference |
| Mild Liver Disease | N/A | 1.02 (0.99-1.05) | 0.91 (0.85-0.98) | 1.07 (1.01-1.13) | 1.01 (0.96-1.07) |
| Moderate to Severe Liver Disease | N/A | 1.25 (1.20-1.30) | 0.78 (0.71-0.87) | 1.28 (1.18-1.38) | 1.65 (1.55-1.77) |
| Diabetes Mellitus |  |  |  |  |  |
| No History of Diabetes | N/A | Reference | Reference | Reference | Reference |
| Diabetes without Chronic Complications | N/A | 1.07 (1.04-1.10) | 1.03 (0.97-1.09) | 1.03 (0.98-1.09) | 1.03 (0.99-1.08) |
| Diabetes with Chronic Complications | N/A | 1.20 (1.17-1.23) | 1.10 (1.05-1.15) | 1.17 (1.12-1.23) | 1.09 (1.05-1.13) |
| Hemiplegia or paraplegia | N/A | 0.93 (0.89-0.97) | 0.88 (0.81-0.96) | 0.96 (0.88-1.05) | 1.08 (1.00-1.16) |
| Renal Disease | N/A | 1.96 (1.92-2.00) | 0.86 (0.83-0.90) | 0.91 (0.87-0.94) | 1.17 (1.13-1.21) |
| Cancer |  |  |  |  |  |
| No History of Malignant Cancer | N/A | Reference | Reference | Reference | Reference |
| Any Malignancy Except Neoplasm of skin | N/A | 0.90 (0.88-0.92) | 0.78 (0.74-0.82) | 0.83 (0.79-0.87) | 1.10 (1.06-1.14) |
| Metastatic Solid Tumor | N/A | 0.86 (0.82-0.90) | 0.68 (0.61-0.75) | 0.78 (0.71-0.85) | 2.03 (1.92-2.14) |
| HIV | N/A | 0.93 (0.85-1.01) | 0.89 (0.73-1.09) | 0.86 (0.73-1.01) | 1.00 (0.84-1.18) |
| Obesity | N/A | 1.20 (1.18-1.22) | 1.09 (1.05-1.13) | 1.21 (1.18-1.25) | 1.01 (0.98-1.04) |
| Hypertension | N/A | 1.11 (1.08-1.13) | 1.04 (0.99-1.08) | 0.86 (0.83-0.89) | 0.84 (0.82-0.87) |
| Former or Current Tobacco User | N/A | 1.11 (1.08-1.14) | 1.26 (1.19-1.33) | 0.97 (0.93-1.02) | 0.96 (0.91-1.00) |
| History of Substance Abuse Disorder | N/A | 0.99 (0.96-1.02) | 0.94 (0.87-1.00) | 1.07 (1.02-1.13) | 0.96 (0.90-1.01) |
| Vaccination Status Prior to SARS-CoV-2 Infection |  |  |  |  |  |
| No Documented COVID-19 Vaccination | N/A | Reference | Reference | Reference | Reference |
| Primary Vaccination Series | N/A | 0.75 (0.73-0.77) | 0.71 (0.67-0.75) | 0.57 (0.54-0.60) | 0.68 (0.65-0.71) |
| Primary+ Vaccination Series | N/A | 0.67 (0.65-0.69) | 0.66 (0.62-0.71) | 0.49 (0.45-0.52) | 0.48 (0.46-0.51) |
| Census Region |  |  |  |  |  |
| Midwest | N/A | Reference | Reference | Reference | Reference |
| Northeast | N/A | 1.77 (1.73-1.81) | 2.36 (2.25-2.47) | 1.44 (1.37-1.50) | 0.88 (0.85-0.92) |
| South | N/A | 1.89 (1.86-1.93) | 2.29 (2.20-2.39) | 2.54 (2.45-2.63) | 1.18 (1.14-1.21) |
| West | N/A | 1.75 (1.71-1.80) | 1.62 (1.53-1.72) | 2.73 (2.62-2.85) | 1.11 (1.07-1.15) |
| Rurality |  |  |  |  |  |
| Urban | N/A | Reference | Reference | Reference | Reference |
| Urban-Adjacent Rural | N/A | 1.02 (1.00-1.04) | 1.05 (1.00-1.10) | 1.33 (1.28-1.38) | 1.29 (1.25-1.33) |
| Nonurban-Adjacent Rural | N/A | 1.09 (1.04-1.14) | 1.07 (0.99-1.17) | 1.59 (1.49-1.69) | 1.37 (1.29-1.45) |
| Anticoagulants | N/A | 0.17 (0.17-0.18) | 0.14 (0.13-0.15) | 0.34 (0.33-0.35) | 0.87 (0.85-0.89) |

**F. Tocilizumab**

| **Characteristic** | **Tocilizumab, Available after June 24, 2021** | | | | |
| --- | --- | --- | --- | --- | --- |
|  | **Hospitalization** | **AKI/Dialysis** | **MACE** | **IMV/ECMO** | **Death** |
|  | **Adjusted Odds Ratio**  **(95% CI)** | **Adjusted Hazard Ratio**  **(95% CI)** | **Adjusted Hazard Ratio**  **(95% CI)** | **Adjusted Hazard Ratio**  **(95% CI)** | **Adjusted Hazard Ratio**  **(95% CI)** |
| Sex |  |  |  |  |  |
| Female | N/A | N/A | N/A | N/A | Reference |
| Male | N/A | N/A | N/A | N/A | 1.33 (1.29-1.36) |
| Age at COVID-19 Diagnosis | N/A | N/A | N/A | N/A | 1.04 (1.03-1.04) |
| Race/Ethnicity |  |  |  |  |  |
| White Non-Hispanic | N/A | N/A | N/A | N/A | Reference |
| Black or African American Non-Hispanic | N/A | N/A | N/A | N/A | 0.91 (0.87-0.95) |
| Hispanic or Latino Any Race | N/A | N/A | N/A | N/A | 1.03 (0.98-1.09) |
| Other | N/A | N/A | N/A | N/A | 1.09 (1.00-1.18) |
| Missing/Unknown | N/A | N/A | N/A | N/A | 1.17 (1.09-1.26) |
| Myocardial infarction (MI) | N/A | N/A | N/A | N/A | 1.07 (1.02-1.13) |
| Congestive heart failure (CHF) | N/A | N/A | N/A | N/A | 1.12 (1.07-1.16) |
| Peripheral vascular disease (PVD) | N/A | N/A | N/A | N/A | 1.03 (0.98-1.08) |
| Cerebrovascular disease (CVD) | N/A | N/A | N/A | N/A | 0.93 (0.89-0.98) |
| Dementia | N/A | N/A | N/A | N/A | 1.18 (1.12-1.24) |
| Chronic pulmonary disease (CPD) | N/A | N/A | N/A | N/A | 1.06 (1.03-1.10) |
| Rheumatologic disease (RD) | N/A | N/A | N/A | N/A | 1.04 (0.99-1.08) |
| Peptic ulcer disease (PUD) | N/A | N/A | N/A | N/A | 0.98 (0.91-1.06) |
| Liver Disease |  |  |  |  |  |
| No History of Liver Disease | N/A | N/A | N/A | N/A | Reference |
| Mild Liver Disease | N/A | N/A | N/A | N/A | 1.03 (0.97-1.09) |
| Moderate to Severe Liver Disease | N/A | N/A | N/A | N/A | 1.68 (1.56-1.81) |
| Diabetes Mellitus |  |  |  |  |  |
| No History of Diabetes | N/A | N/A | N/A | N/A | Reference |
| Diabetes without Chronic Complications | N/A | N/A | N/A | N/A | 1.03 (0.98-1.09) |
| Diabetes with Chronic Complications | N/A | N/A | N/A | N/A | 1.07 (1.02-1.11) |
| Hemiplegia or paraplegia | N/A | N/A | N/A | N/A | 1.06 (0.98-1.15) |
| Renal Disease | N/A | N/A | N/A | N/A | 1.18 (1.14-1.23) |
| Cancer |  |  |  |  |  |
| No History of Malignant Cancer | N/A | N/A | N/A | N/A | Reference |
| Any Malignancy Except Neoplasm of skin | N/A | N/A | N/A | N/A | 1.14 (1.09-1.19) |
| Metastatic Solid Tumor | N/A | N/A | N/A | N/A | 2.18 (2.05-2.32) |
| HIV | N/A | N/A | N/A | N/A | 0.93 (0.76-1.14) |
| Obesity | N/A | N/A | N/A | N/A | 0.98 (0.95-1.01) |
| Hypertension | N/A | N/A | N/A | N/A | 0.86 (0.83-0.89) |
| Former or Current Tobacco User | N/A | N/A | N/A | N/A | 0.96 (0.91-1.01) |
| History of Substance Abuse Disorder | N/A | N/A | N/A | N/A | 1.00 (0.94-1.06) |
| Vaccination Status Prior to SARS-CoV-2 Infection |  |  |  |  |  |
| No Documented COVID-19 Vaccination | N/A | N/A | N/A | N/A | Reference |
| Primary Vaccination Series | N/A | N/A | N/A | N/A | 0.71 (0.68-0.74) |
| Primary+ Vaccination Series | N/A | N/A | N/A | N/A | 0.51 (0.48-0.54) |
| Census Region |  |  |  |  |  |
| Midwest | N/A | N/A | N/A | N/A | Reference |
| Northeast | N/A | N/A | N/A | N/A | 0.76 (0.72-0.79) |
| South | N/A | N/A | N/A | N/A | 1.07 (1.03-1.10) |
| West | N/A | N/A | N/A | N/A | 1.02 (0.97-1.07) |
| Rurality |  |  |  |  |  |
| Urban | N/A | N/A | N/A | N/A | Reference |
| Urban-Adjacent Rural | N/A | N/A | N/A | N/A | 1.30 (1.25-1.34) |
| Nonurban-Adjacent Rural | N/A | N/A | N/A | N/A | 1.38 (1.29-1.47) |
| Tocilizumab | N/A | N/A | N/A | N/A | 3.98 (3.76-4.21) |

**G. Dexamethasone**

| **Characteristic** | **Dexamethasone, Available after June 16, 2020** | | | | |
| --- | --- | --- | --- | --- | --- |
|  | **Hospitalization** | **AKI/Dialysis** | **MACE** | **IMV/ECMO** | **Death** |
|  | **Adjusted Odds Ratio**  **(95% CI)** | **Adjusted Hazard Ratio**  **(95% CI)** | **Adjusted Hazard Ratio**  **(95% CI)** | **Adjusted Hazard Ratio**  **(95% CI)** | **Adjusted Hazard Ratio**  **(95% CI)** |
| Sex |  |  |  |  |  |
| Female | N/A | N/A | N/A | N/A | Reference |
| Male | N/A | N/A | N/A | N/A | 1.33 (1.30-1.36) |
| Age at COVID-19 Diagnosis | N/A | N/A | N/A | N/A | 1.04 (1.04-1.04) |
| Race/Ethnicity |  |  |  |  |  |
| White Non-Hispanic | N/A | N/A | N/A | N/A | Reference |
| Black or African American Non-Hispanic | N/A | N/A | N/A | N/A | 0.95 (0.92-0.99) |
| Hispanic or Latino Any Race | N/A | N/A | N/A | N/A | 1.10 (1.06-1.15) |
| Other | N/A | N/A | N/A | N/A | 1.19 (1.12-1.27) |
| Missing/Unknown | N/A | N/A | N/A | N/A | 1.28 (1.20-1.35) |
| Myocardial infarction (MI) | N/A | N/A | N/A | N/A | 1.05 (1.01-1.10) |
| Congestive heart failure (CHF) | N/A | N/A | N/A | N/A | 1.14 (1.10-1.18) |
| Peripheral vascular disease (PVD) | N/A | N/A | N/A | N/A | 1.03 (0.99-1.08) |
| Cerebrovascular disease (CVD) | N/A | N/A | N/A | N/A | 0.93 (0.90-0.98) |
| Dementia | N/A | N/A | N/A | N/A | 1.20 (1.15-1.26) |
| Chronic pulmonary disease (CPD) | N/A | N/A | N/A | N/A | 1.02 (0.99-1.05) |
| Rheumatologic disease (RD) | N/A | N/A | N/A | N/A | 1.00 (0.96-1.05) |
| Peptic ulcer disease (PUD) | N/A | N/A | N/A | N/A | 0.96 (0.90-1.04) |
| Liver Disease |  |  |  |  |  |
| No History of Liver Disease | N/A | N/A | N/A | N/A | Reference |
| Mild Liver Disease | N/A | N/A | N/A | N/A | 1.02 (0.96-1.07) |
| Moderate to Severe Liver Disease | N/A | N/A | N/A | N/A | 1.75 (1.64-1.86) |
| Diabetes Mellitus |  |  |  |  |  |
| No History of Diabetes | N/A | N/A | N/A | N/A | Reference |
| Diabetes without Chronic Complications | N/A | N/A | N/A | N/A | 1.04 (1.00-1.09) |
| Diabetes with Chronic Complications | N/A | N/A | N/A | N/A | 1.11 (1.07-1.15) |
| Hemiplegia or paraplegia | N/A | N/A | N/A | N/A | 1.09 (1.02-1.18) |
| Renal Disease | N/A | N/A | N/A | N/A | 1.21 (1.17-1.25) |
| Cancer |  |  |  |  |  |
| No History of Malignant Cancer | N/A | N/A | N/A | N/A | Reference |
| Any Malignancy Except Neoplasm of skin | N/A | N/A | N/A | N/A | 1.08 (1.05-1.12) |
| Metastatic Solid Tumor | N/A | N/A | N/A | N/A | 1.94 (1.84-2.05) |
| HIV | N/A | N/A | N/A | N/A | 1.05 (0.89-1.25) |
| Obesity | N/A | N/A | N/A | N/A | 0.90 (0.88-0.93) |
| Hypertension | N/A | N/A | N/A | N/A | 0.85 (0.82-0.87) |
| Former or Current Tobacco User | N/A | N/A | N/A | N/A | 0.94 (0.90-0.98) |
| History of Substance Abuse Disorder | N/A | N/A | N/A | N/A | 1.01 (0.96-1.07) |
| Vaccination Status Prior to SARS-CoV-2 Infection |  |  |  |  |  |
| No Documented COVID-19 Vaccination | N/A | N/A | N/A | N/A | Reference |
| Primary Vaccination Series | N/A | N/A | N/A | N/A | 0.73 (0.70-0.77) |
| Primary+ Vaccination Series | N/A | N/A | N/A | N/A | 0.54 (0.51-0.57) |
| Census Region |  |  |  |  |  |
| Midwest | N/A | N/A | N/A | N/A | Reference |
| Northeast | N/A | N/A | N/A | N/A | 0.78 (0.75-0.81) |
| South | N/A | N/A | N/A | N/A | 0.98 (0.95-1.01) |
| West | N/A | N/A | N/A | N/A | 0.93 (0.90-0.97) |
| Rurality |  |  |  |  |  |
| Urban | N/A | N/A | N/A | N/A | Reference |
| Urban-Adjacent Rural | N/A | N/A | N/A | N/A | 1.30 (1.26-1.35) |
| Nonurban-Adjacent Rural | N/A | N/A | N/A | N/A | 1.35 (1.28-1.43) |
| Dexamethasone | N/A | N/A | N/A | N/A | 1.93 (1.88-1.98) |

**eTable 16. Sensitivity Analysis 4: Multivariable Regression for Adverse Acute COVID-19 Events with COVID-19 Therapies Individually During the Time Periods they were Widely in Use Stratified by Rural-Dwelling Status**

**A. Nirmatrelvir/Ritonavir**

| **Characteristic** | **Nirmatrelvir/Ritonavir, Available after December 22, 2021** | | | | | | | | | |
| --- | --- | --- | --- | --- | --- | --- | --- | --- | --- | --- |
|  | **Hospitalization** | | **AKI/Dialysis** | | **MACE** | | **IMV/ECMO** | | **Death** | |
|  | **Urban** | **Rural** | **Urban** | **Rural** | **Urban** | **Rural** | **Urban** | **Rural** | **Urban** | **Rural** |
|  | **Adjusted Odds Ratio  (95% CI)** | **Adjusted Odds Ratio  (95% CI)** | **Adjusted Hazard Ratio  (95% CI)** | **Adjusted Hazard Ratio  (95% CI)** | **Adjusted Hazard Ratio (95% CI)** | **Adjusted Hazard Ratio (95% CI)** | **Adjusted Hazard Ratio (95% CI)** | **Adjusted Hazard Ratio (95% CI)** | **Adjusted Hazard Ratio (95% CI)** | **Adjusted Hazard Ratio (95% CI)** |
| Sex |  |  |  |  |  |  |  |  |  |  |
| Female | Reference | Reference | Reference | Reference | Reference | Reference | Reference | Reference | Reference | Reference |
| Male | 1.15 (1.13-1.16) | 1.21 (1.18-1.25) | 1.39 (1.36-1.42) | 1.36 (1.29-1.45) | 1.39 (1.33-1.46) | 1.49 (1.34-1.66) | 1.62 (1.54-1.70) | 1.63 (1.47-1.81) | 1.32 (1.26-1.38) | 1.26 (1.16-1.38) |
| Age at COVID-19 Diagnosis | 1.03 (1.03-1.04) | 1.04 (1.04-1.04) | 1.02 (1.01-1.02) | 1.01 (1.01-1.02) | 1.03 (1.02-1.03) | 1.02 (1.02-1.03) | 1.00 (1.00-1.00) | 1.00 (1.00-1.00) | 1.04 (1.03-1.04) | 1.03 (1.03-1.04) |
| Race/Ethnicity |  |  |  |  |  |  |  |  |  |  |
| White Non-Hispanic | Reference | Reference | Reference | Reference | Reference | Reference | Reference | Reference | Reference | Reference |
| Black or African American Non-Hispanic | 1.64 (1.61-1.67) | 1.18 (1.11-1.25) | 1.51 (1.46-1.55) | 1.65 (1.51-1.81) | 1.23 (1.15-1.30) | 1.41 (1.18-1.68) | 1.05 (0.98-1.12) | 1.27 (1.06-1.50) | 0.89 (0.83-0.94) | 1.08 (0.91-1.29) |
| Hispanic or Latino Any Race | 1.65 (1.61-1.68) | 1.38 (1.27-1.49) | 1.09 (1.04-1.14) | 1.12 (0.95-1.32) | 1.02 (0.93-1.12) | 0.90 (0.63-1.30) | 1.03 (0.95-1.12) | 1.27 (0.99-1.61) | 1.00 (0.93-1.08) | 1.22 (0.95-1.56) |
| Other | 0.96 (0.93-0.99) | 0.45 (0.40-0.51) | 1.24 (1.16-1.32) | 1.37 (1.08-1.73) | 1.27 (1.12-1.43) | 1.44 (0.94-2.23) | 1.29 (1.14-1.45) | 1.63 (1.13-2.36) | 1.11 (0.99-1.24) | 1.34 (0.94-1.92) |
| Missing/Unknown | 0.95 (0.92-0.98) | 0.87 (0.78-0.97) | 1.14 (1.07-1.21) | 1.37 (1.12-1.67) | 1.33 (1.19-1.48) | 1.61 (1.15-2.24) | 1.30 (1.17-1.45) | 1.74 (1.33-2.27) | 1.08 (0.97-1.20) | 1.62 (1.24-2.12) |
| Myocardial infarction (MI) | 1.22 (1.19-1.25) | 1.13 (1.06-1.20) | 1.04 (1.00-1.08) | 0.98 (0.90-1.07) | 2.67 (2.50-2.85) | 2.79 (2.41-3.23) | 1.12 (1.03-1.22) | 1.07 (0.89-1.30) | 1.09 (1.02-1.17) | 1.08 (0.94-1.24) |
| Congestive heart failure (CHF) | 1.86 (1.82-1.90) | 1.70 (1.62-1.79) | 1.06 (1.03-1.09) | 1.08 (1.00-1.17) | 1.17 (1.10-1.25) | 1.32 (1.14-1.53) | 1.12 (1.04-1.20) | 1.09 (0.93-1.28) | 1.19 (1.12-1.26) | 1.12 (0.99-1.27) |
| Peripheral vascular disease (PVD) | 1.09 (1.06-1.11) | 1.08 (1.02-1.15) | 0.98 (0.95-1.02) | 0.93 (0.84-1.01) | 1.07 (1.00-1.15) | 1.03 (0.88-1.22) | 0.92 (0.84-1.00) | 0.89 (0.73-1.09) | 1.06 (1.00-1.14) | 0.96 (0.83-1.11) |
| Cerebrovascular disease (CVD) | 1.02 (0.99-1.05) | 0.94 (0.88-1.00) | 0.95 (0.92-0.99) | 0.91 (0.83-1.01) | 1.19 (1.11-1.28) | 1.24 (1.04-1.47) | 0.99 (0.90-1.08) | 0.99 (0.80-1.21) | 0.93 (0.87-0.99) | 1.05 (0.91-1.21) |
| Dementia | 1.69 (1.64-1.75) | 1.26 (1.16-1.37) | 0.83 (0.79-0.87) | 0.87 (0.77-0.99) | 0.65 (0.59-0.72) | 0.55 (0.42-0.72) | 0.67 (0.60-0.76) | 0.46 (0.32-0.66) | 1.24 (1.15-1.32) | 1.24 (1.06-1.45) |
| Chronic pulmonary disease (CPD) | 1.19 (1.17-1.21) | 1.31 (1.26-1.36) | 0.93 (0.90-0.95) | 0.91 (0.85-0.98) | 0.91 (0.86-0.97) | 0.76 (0.67-0.88) | 1.09 (1.02-1.16) | 1.13 (0.99-1.30) | 1.07 (1.02-1.13) | 1.10 (0.99-1.22) |
| Rheumatologic disease (RD) | 0.93 (0.91-0.95) | 0.98 (0.93-1.04) | 1.12 (1.08-1.16) | 1.10 (1.01-1.20) | 0.98 (0.91-1.06) | 0.85 (0.71-1.02) | 1.12 (1.03-1.21) | 1.00 (0.83-1.20) | 1.05 (0.98-1.12) | 0.98 (0.85-1.13) |
| Peptic ulcer disease (PUD) | 1.11 (1.06-1.16) | 1.09 (0.98-1.21) | 1.15 (1.09-1.22) | 1.03 (0.88-1.20) | 0.96 (0.85-1.09) | 0.73 (0.52-1.03) | 1.02 (0.89-1.16) | 0.93 (0.67-1.28) | 1.00 (0.90-1.12) | 0.94 (0.73-1.21) |
| Liver Disease |  |  |  |  |  |  |  |  |  |  |
| No History of Liver Disease | Reference | Reference | Reference | Reference | Reference | Reference | Reference | Reference | Reference | Reference |
| Mild Liver Disease | 0.99 (0.97-1.02) | 0.98 (0.92-1.05) | 1.01 (0.96-1.05) | 1.00 (0.89-1.12) | 0.94 (0.86-1.04) | 0.80 (0.62-1.03) | 1.07 (0.97-1.17) | 0.94 (0.75-1.18) | 1.02 (0.93-1.11) | 1.05 (0.86-1.27) |
| Moderate to Severe Liver Disease | 1.74 (1.66-1.83) | 1.70 (1.52-1.90) | 1.34 (1.27-1.42) | 1.21 (1.04-1.39) | 0.87 (0.75-1.01) | 0.81 (0.57-1.15) | 1.49 (1.33-1.68) | 1.43 (1.10-1.85) | 1.80 (1.63-1.99) | 1.85 (1.49-2.29) |
| Diabetes Mellitus |  |  |  |  |  |  |  |  |  |  |
| No History of Diabetes | Reference | Reference | Reference | Reference | Reference | Reference | Reference | Reference | Reference | Reference |
| Diabetes without Chronic Complications | 1.33 (1.30-1.36) | 1.42 (1.34-1.50) | 1.05 (1.01-1.10) | 1.07 (0.97-1.19) | 1.00 (0.91-1.09) | 1.01 (0.83-1.23) | 0.96 (0.87-1.05) | 0.89 (0.73-1.09) | 0.95 (0.88-1.03) | 1.10 (0.94-1.27) |
| Diabetes with Chronic Complications | 1.32 (1.29-1.35) | 1.47 (1.40-1.55) | 1.25 (1.21-1.29) | 1.33 (1.23-1.45) | 1.13 (1.06-1.22) | 1.29 (1.10-1.50) | 1.17 (1.09-1.27) | 1.09 (0.92-1.29) | 1.02 (0.96-1.09) | 1.10 (0.97-1.25) |
| Hemiplegia or paraplegia | 2.02 (1.92-2.11) | 1.82 (1.62-2.04) | 0.93 (0.87-0.99) | 0.88 (0.74-1.04) | 0.87 (0.77-0.98) | 0.88 (0.65-1.18) | 1.05 (0.92-1.20) | 1.17 (0.86-1.60) | 1.15 (1.03-1.28) | 1.00 (0.78-1.29) |
| Renal Disease | 1.90 (1.86-1.93) | 1.64 (1.57-1.71) | 2.48 (2.41-2.56) | 2.40 (2.23-2.59) | 0.96 (0.90-1.02) | 1.01 (0.88-1.17) | 1.06 (0.99-1.13) | 1.11 (0.95-1.28) | 1.18 (1.12-1.24) | 1.19 (1.06-1.33) |
| Cancer |  |  |  |  |  |  |  |  |  |  |
| No History of Malignant Cancer | Reference | Reference | Reference | Reference | Reference | Reference | Reference | Reference | Reference | Reference |
| Any Malignancy Except Neoplasm of skin | 0.99 (0.97-1.01) | 1.12 (1.06-1.17) | 0.93 (0.89-0.96) | 0.99 (0.91-1.08) | 0.78 (0.73-0.84) | 0.84 (0.72-0.99) | 0.94 (0.87-1.02) | 0.99 (0.84-1.17) | 1.21 (1.14-1.28) | 1.32 (1.17-1.48) |
| Metastatic Solid Tumor | 2.08 (2.00-2.17) | 2.12 (1.92-2.33) | 0.97 (0.91-1.04) | 1.04 (0.89-1.21) | 0.71 (0.61-0.82) | 0.65 (0.46-0.93) | 0.95 (0.83-1.09) | 0.68 (0.48-0.97) | 2.65 (2.45-2.87) | 2.15 (1.80-2.58) |
| HIV | 1.10 (1.02-1.19) | 1.16 (0.88-1.51) | 0.96 (0.85-1.08) | 0.81 (0.51-1.29) | 0.93 (0.71-1.23) | 0.54 (0.17-1.69) | 0.90 (0.71-1.16) | 0.00 (0.00-Inf) | 1.03 (0.79-1.34) | 0.83 (0.34-2.00) |
| Obesity | 0.87 (0.86-0.89) | 0.79 (0.76-0.82) | 1.05 (1.02-1.08) | 1.06 (0.99-1.13) | 0.93 (0.88-0.99) | 1.05 (0.92-1.19) | 1.03 (0.97-1.08) | 1.07 (0.95-1.21) | 0.94 (0.89-0.99) | 0.83 (0.75-0.92) |
| Hypertension | 0.83 (0.81-0.84) | 0.84 (0.81-0.87) | 1.09 (1.06-1.13) | 1.08 (1.00-1.17) | 1.05 (0.98-1.12) | 0.91 (0.78-1.05) | 0.84 (0.79-0.90) | 0.82 (0.71-0.94) | 0.79 (0.75-0.84) | 0.87 (0.78-0.97) |
| Former or Current Tobacco User | 1.72 (1.68-1.76) | 1.92 (1.83-2.02) | 1.12 (1.08-1.17) | 1.10 (1.01-1.20) | 1.26 (1.17-1.36) | 1.36 (1.16-1.58) | 1.06 (0.98-1.14) | 1.07 (0.92-1.24) | 0.99 (0.92-1.06) | 0.98 (0.85-1.13) |
| History of Substance Abuse Disorder | 2.99 (2.91-3.07) | 2.48 (2.31-2.65) | 1.01 (0.97-1.06) | 1.05 (0.94-1.18) | 0.93 (0.85-1.02) | 0.83 (0.66-1.05) | 1.25 (1.15-1.36) | 1.23 (1.03-1.47) | 1.02 (0.93-1.11) | 1.03 (0.85-1.26) |
| Vaccination Status Prior to SARS-CoV-2 Infection |  |  |  |  |  |  |  |  |  |  |
| No Documented COVID-19 Vaccination | Reference | Reference | Reference | Reference | Reference | Reference | Reference | Reference | Reference | Reference |
| Primary Vaccination Series | 0.71 (0.70-0.72) | 0.65 (0.62-0.68) | 0.83 (0.80-0.87) | 0.75 (0.68-0.82) | 0.74 (0.68-0.80) | 0.81 (0.68-0.96) | 0.74 (0.69-0.80) | 0.68 (0.57-0.81) | 0.81 (0.76-0.86) | 0.74 (0.65-0.84) |
| Primary+ Vaccination Series | 0.57 (0.56-0.59) | 0.51 (0.49-0.54) | 0.74 (0.71-0.77) | 0.71 (0.65-0.77) | 0.68 (0.63-0.73) | 0.72 (0.61-0.86) | 0.65 (0.60-0.71) | 0.64 (0.53-0.77) | 0.63 (0.59-0.67) | 0.55 (0.48-0.63) |
| Census Region |  |  |  |  |  |  |  |  |  |  |
| Midwest | Reference | Reference | Reference | Reference | Reference | Reference | Reference | Reference | Reference | Reference |
| Northeast | 1.44 (1.41-1.47) | 0.68 (0.64-0.72) | 1.09 (1.05-1.13) | 1.36 (1.22-1.52) | 1.10 (1.02-1.19) | 1.68 (1.40-2.01) | 0.81 (0.75-0.88) | 1.47 (1.22-1.77) | 0.79 (0.74-0.85) | 0.80 (0.68-0.94) |
| South | 1.25 (1.23-1.28) | 1.86 (1.80-1.92) | 1.24 (1.20-1.27) | 1.61 (1.50-1.72) | 1.38 (1.29-1.46) | 1.60 (1.41-1.81) | 1.42 (1.34-1.51) | 1.61 (1.43-1.82) | 1.02 (0.96-1.07) | 1.06 (0.96-1.17) |
| West | 0.84 (0.83-0.86) | 1.53 (1.42-1.65) | 1.31 (1.26-1.36) | 2.14 (1.87-2.45) | 1.12 (1.04-1.22) | 1.24 (0.93-1.67) | 1.48 (1.37-1.59) | 2.66 (2.18-3.24) | 0.91 (0.85-0.97) | 1.41 (1.16-1.71) |
| Nirmatrelvir/ritonavir | 0.08 (0.08-0.09) | 0.06 (0.05-0.07) | 0.35 (0.28-0.42) | 0.51 (0.32-0.82) | 0.55 (0.40-0.76) | 0.65 (0.29-1.46) | 0.22 (0.14-0.36) | 0.09 (0.01-0.67) | 0.42 (0.30-0.57) | 0.59 (0.31-1.14) |

**B. Molnupiravir**

| **Characteristic** | **Molnupinavir, Available after December 23, 2021** | | | | | | | | | |
| --- | --- | --- | --- | --- | --- | --- | --- | --- | --- | --- |
|  | **Hospitalization** | | **AKI/Dialysis** | | **MACE** | | **IMV/ECMO** | | **Death** | |
|  | **Urban** | **Rural** | **Urban** | **Rural** | **Urban** | **Rural** | **Urban** | **Rural** | **Urban** | **Rural** |
|  | **Adjusted Odds Ratio  (95% CI)** | **Adjusted Odds Ratio  (95% CI)** | **Adjusted Hazard Ratio  (95% CI)** | **Adjusted Hazard Ratio  (95% CI)** | **Adjusted Hazard Ratio (95% CI)** | **Adjusted Hazard Ratio (95% CI)** | **Adjusted Hazard Ratio (95% CI)** | **Adjusted Hazard Ratio (95% CI)** | **Adjusted Hazard Ratio (95% CI)** | **Adjusted Hazard Ratio (95% CI)** |
| Sex |  |  |  |  |  |  |  |  |  |  |
| Female | Reference | Reference | Reference | Reference | Reference | Reference | Reference | Reference | Reference | Reference |
| Male | 1.15 (1.13-1.16) | 1.21 (1.18-1.25) | 1.39 (1.36-1.43) | 1.37 (1.29-1.45) | 1.40 (1.33-1.47) | 1.49 (1.34-1.66) | 1.62 (1.54-1.70) | 1.63 (1.47-1.81) | 1.32 (1.27-1.38) | 1.27 (1.16-1.38) |
| Age at COVID-19 Diagnosis | 1.03 (1.03-1.03) | 1.04 (1.04-1.04) | 1.02 (1.01-1.02) | 1.01 (1.01-1.02) | 1.03 (1.02-1.03) | 1.02 (1.02-1.03) | 1.00 (1.00-1.00) | 1.00 (1.00-1.00) | 1.04 (1.03-1.04) | 1.03 (1.03-1.04) |
| Race/Ethnicity |  |  |  |  |  |  |  |  |  |  |
| White Non-Hispanic | Reference | Reference | Reference | Reference | Reference | Reference | Reference | Reference | Reference | Reference |
| Black or African American Non-Hispanic | 1.66 (1.63-1.69) | 1.18 (1.11-1.25) | 1.51 (1.46-1.55) | 1.65 (1.51-1.81) | 1.23 (1.15-1.30) | 1.41 (1.18-1.68) | 1.05 (0.98-1.12) | 1.27 (1.06-1.51) | 0.89 (0.84-0.94) | 1.08 (0.91-1.29) |
| Hispanic or Latino Any Race | 1.67 (1.63-1.70) | 1.37 (1.27-1.48) | 1.09 (1.04-1.14) | 1.12 (0.95-1.32) | 1.02 (0.93-1.12) | 0.90 (0.63-1.29) | 1.03 (0.95-1.13) | 1.27 (0.99-1.61) | 1.00 (0.93-1.08) | 1.21 (0.95-1.55) |
| Other | 0.97 (0.94-1.00) | 0.46 (0.41-0.52) | 1.23 (1.16-1.32) | 1.36 (1.08-1.73) | 1.26 (1.12-1.43) | 1.44 (0.94-2.23) | 1.28 (1.14-1.44) | 1.63 (1.13-2.35) | 1.10 (0.99-1.23) | 1.34 (0.94-1.91) |
| Missing/Unknown | 0.96 (0.93-0.98) | 0.88 (0.79-0.97) | 1.14 (1.07-1.21) | 1.37 (1.12-1.67) | 1.33 (1.19-1.48) | 1.60 (1.15-2.24) | 1.30 (1.16-1.44) | 1.73 (1.33-2.26) | 1.08 (0.97-1.19) | 1.62 (1.23-2.12) |
| Myocardial infarction (MI) | 1.24 (1.20-1.27) | 1.15 (1.08-1.22) | 1.04 (1.00-1.08) | 0.98 (0.90-1.07) | 2.67 (2.50-2.85) | 2.79 (2.41-3.23) | 1.12 (1.03-1.22) | 1.08 (0.89-1.30) | 1.09 (1.02-1.17) | 1.08 (0.94-1.24) |
| Congestive heart failure (CHF) | 1.91 (1.87-1.95) | 1.74 (1.65-1.83) | 1.06 (1.03-1.09) | 1.08 (1.00-1.17) | 1.18 (1.10-1.25) | 1.32 (1.14-1.53) | 1.12 (1.05-1.21) | 1.10 (0.93-1.29) | 1.19 (1.13-1.26) | 1.12 (0.99-1.27) |
| Peripheral vascular disease (PVD) | 1.10 (1.07-1.13) | 1.08 (1.02-1.15) | 0.99 (0.95-1.02) | 0.92 (0.84-1.01) | 1.08 (1.00-1.16) | 1.03 (0.87-1.22) | 0.92 (0.85-1.01) | 0.89 (0.73-1.08) | 1.07 (1.00-1.14) | 0.96 (0.83-1.11) |
| Cerebrovascular disease (CVD) | 1.02 (1.00-1.05) | 0.95 (0.89-1.01) | 0.95 (0.92-0.99) | 0.91 (0.83-1.01) | 1.19 (1.11-1.28) | 1.24 (1.05-1.47) | 0.98 (0.90-1.07) | 0.98 (0.80-1.20) | 0.93 (0.87-0.99) | 1.05 (0.91-1.21) |
| Dementia | 1.72 (1.66-1.77) | 1.26 (1.16-1.36) | 0.83 (0.79-0.87) | 0.87 (0.77-0.99) | 0.65 (0.59-0.72) | 0.55 (0.42-0.72) | 0.67 (0.60-0.76) | 0.46 (0.32-0.66) | 1.24 (1.15-1.32) | 1.24 (1.06-1.45) |
| Chronic pulmonary disease (CPD) | 1.19 (1.17-1.21) | 1.31 (1.26-1.36) | 0.92 (0.90-0.95) | 0.91 (0.85-0.98) | 0.91 (0.86-0.97) | 0.76 (0.67-0.88) | 1.08 (1.02-1.15) | 1.13 (0.99-1.29) | 1.07 (1.02-1.13) | 1.10 (0.98-1.22) |
| Rheumatologic disease (RD) | 0.93 (0.90-0.95) | 0.98 (0.93-1.04) | 1.12 (1.08-1.16) | 1.10 (1.01-1.20) | 0.98 (0.91-1.06) | 0.85 (0.71-1.02) | 1.12 (1.03-1.21) | 1.00 (0.83-1.20) | 1.04 (0.98-1.12) | 0.98 (0.85-1.13) |
| Peptic ulcer disease (PUD) | 1.10 (1.05-1.14) | 1.08 (0.98-1.20) | 1.15 (1.08-1.21) | 1.03 (0.88-1.20) | 0.96 (0.84-1.09) | 0.73 (0.52-1.03) | 1.02 (0.89-1.16) | 0.92 (0.67-1.28) | 1.00 (0.90-1.12) | 0.94 (0.73-1.21) |
| Liver Disease |  |  |  |  |  |  |  |  |  |  |
| No History of Liver Disease | Reference | Reference | Reference | Reference | Reference | Reference | Reference | Reference | Reference | Reference |
| Mild Liver Disease | 0.99 (0.96-1.02) | 0.99 (0.92-1.05) | 1.01 (0.96-1.05) | 1.00 (0.89-1.12) | 0.94 (0.85-1.04) | 0.80 (0.62-1.03) | 1.07 (0.97-1.17) | 0.94 (0.74-1.18) | 1.02 (0.93-1.11) | 1.05 (0.86-1.27) |
| Moderate to Severe Liver Disease | 1.77 (1.69-1.85) | 1.74 (1.55-1.94) | 1.34 (1.27-1.42) | 1.21 (1.05-1.40) | 0.87 (0.75-1.01) | 0.81 (0.57-1.15) | 1.50 (1.34-1.68) | 1.42 (1.10-1.85) | 1.80 (1.63-1.99) | 1.85 (1.49-2.30) |
| Diabetes Mellitus |  |  |  |  |  |  |  |  |  |  |
| No History of Diabetes | Reference | Reference | Reference | Reference | Reference | Reference | Reference | Reference | Reference | Reference |
| Diabetes without Chronic Complications | 1.33 (1.30-1.36) | 1.42 (1.35-1.50) | 1.05 (1.01-1.10) | 1.08 (0.97-1.19) | 1.00 (0.91-1.09) | 1.01 (0.83-1.23) | 0.96 (0.87-1.05) | 0.89 (0.73-1.09) | 0.95 (0.88-1.03) | 1.10 (0.94-1.27) |
| Diabetes with Chronic Complications | 1.33 (1.30-1.36) | 1.50 (1.42-1.58) | 1.25 (1.21-1.29) | 1.34 (1.24-1.45) | 1.14 (1.06-1.22) | 1.29 (1.10-1.51) | 1.18 (1.09-1.27) | 1.09 (0.92-1.28) | 1.02 (0.96-1.09) | 1.10 (0.97-1.26) |
| Hemiplegia or paraplegia | 2.03 (1.94-2.13) | 1.83 (1.63-2.05) | 0.93 (0.88-0.99) | 0.88 (0.74-1.04) | 0.87 (0.77-0.98) | 0.88 (0.65-1.18) | 1.05 (0.92-1.20) | 1.19 (0.87-1.62) | 1.15 (1.03-1.28) | 1.00 (0.78-1.29) |
| Renal Disease | 1.98 (1.94-2.02) | 1.68 (1.60-1.75) | 2.49 (2.42-2.57) | 2.40 (2.23-2.59) | 0.96 (0.90-1.02) | 1.01 (0.87-1.17) | 1.07 (1.00-1.14) | 1.11 (0.96-1.29) | 1.18 (1.12-1.25) | 1.19 (1.06-1.33) |
| Cancer |  |  |  |  |  |  |  |  |  |  |
| No History of Malignant Cancer | Reference | Reference | Reference | Reference | Reference | Reference | Reference | Reference | Reference | Reference |
| Any Malignancy Except Neoplasm of skin | 0.98 (0.96-1.00) | 1.10 (1.05-1.16) | 0.92 (0.89-0.96) | 0.99 (0.91-1.07) | 0.78 (0.73-0.84) | 0.84 (0.72-0.99) | 0.94 (0.87-1.01) | 0.99 (0.84-1.16) | 1.21 (1.14-1.28) | 1.31 (1.17-1.48) |
| Metastatic Solid Tumor | 2.06 (1.98-2.15) | 2.07 (1.88-2.28) | 0.97 (0.91-1.03) | 1.04 (0.89-1.20) | 0.71 (0.61-0.82) | 0.65 (0.46-0.92) | 0.95 (0.83-1.09) | 0.68 (0.48-0.97) | 2.64 (2.44-2.86) | 2.15 (1.80-2.58) |
| HIV | 1.08 (1.00-1.16) | 1.13 (0.86-1.47) | 0.96 (0.85-1.08) | 0.80 (0.50-1.28) | 0.93 (0.71-1.23) | 0.54 (0.17-1.69) | 0.90 (0.70-1.16) | 0.00 (0.00-Inf) | 1.03 (0.79-1.34) | 0.83 (0.34-2.00) |
| Obesity | 0.85 (0.84-0.86) | 0.77 (0.74-0.80) | 1.04 (1.02-1.07) | 1.06 (0.99-1.13) | 0.93 (0.88-0.99) | 1.05 (0.92-1.19) | 1.02 (0.97-1.08) | 1.07 (0.95-1.21) | 0.94 (0.89-0.99) | 0.83 (0.75-0.92) |
| Hypertension | 0.82 (0.81-0.84) | 0.84 (0.80-0.87) | 1.09 (1.06-1.13) | 1.08 (1.00-1.17) | 1.04 (0.98-1.11) | 0.91 (0.78-1.05) | 0.84 (0.79-0.90) | 0.82 (0.71-0.94) | 0.79 (0.75-0.84) | 0.87 (0.78-0.97) |
| Former or Current Tobacco User | 1.69 (1.65-1.73) | 1.90 (1.81-2.00) | 1.12 (1.08-1.17) | 1.10 (1.01-1.20) | 1.26 (1.17-1.36) | 1.36 (1.16-1.58) | 1.06 (0.99-1.14) | 1.07 (0.92-1.24) | 0.99 (0.92-1.06) | 0.98 (0.85-1.13) |
| History of Substance Abuse Disorder | 3.01 (2.93-3.09) | 2.46 (2.30-2.63) | 1.01 (0.97-1.06) | 1.05 (0.94-1.18) | 0.93 (0.85-1.03) | 0.83 (0.66-1.05) | 1.25 (1.16-1.36) | 1.23 (1.03-1.47) | 1.02 (0.93-1.12) | 1.03 (0.85-1.26) |
| Vaccination Status Prior to SARS-CoV-2 Infection |  |  |  |  |  |  |  |  |  |  |
| No Documented COVID-19 Vaccination | Reference | Reference | Reference | Reference | Reference | Reference | Reference | Reference | Reference | Reference |
| Primary Vaccination Series | 0.69 (0.67-0.70) | 0.63 (0.61-0.66) | 0.83 (0.80-0.86) | 0.75 (0.68-0.82) | 0.74 (0.68-0.80) | 0.81 (0.68-0.95) | 0.74 (0.68-0.80) | 0.68 (0.57-0.81) | 0.81 (0.76-0.86) | 0.74 (0.65-0.84) |
| Primary+ Vaccination Series | 0.51 (0.50-0.52) | 0.48 (0.46-0.50) | 0.73 (0.70-0.76) | 0.70 (0.64-0.77) | 0.67 (0.62-0.73) | 0.72 (0.61-0.85) | 0.64 (0.59-0.70) | 0.63 (0.53-0.76) | 0.62 (0.58-0.67) | 0.55 (0.48-0.63) |
| Census Region |  |  |  |  |  |  |  |  |  |  |
| Midwest | Reference | Reference | Reference | Reference | Reference | Reference | Reference | Reference | Reference | Reference |
| Northeast | 1.39 (1.37-1.42) | 0.68 (0.64-0.72) | 1.09 (1.05-1.13) | 1.36 (1.22-1.51) | 1.10 (1.02-1.19) | 1.68 (1.40-2.01) | 0.82 (0.75-0.88) | 1.47 (1.22-1.77) | 0.79 (0.74-0.85) | 0.80 (0.68-0.94) |
| South | 1.20 (1.18-1.22) | 1.82 (1.76-1.88) | 1.24 (1.20-1.27) | 1.61 (1.50-1.72) | 1.37 (1.29-1.46) | 1.60 (1.41-1.81) | 1.42 (1.34-1.51) | 1.61 (1.43-1.81) | 1.01 (0.96-1.07) | 1.06 (0.96-1.17) |
| West | 0.79 (0.78-0.81) | 1.34 (1.24-1.44) | 1.30 (1.25-1.36) | 2.14 (1.87-2.45) | 1.12 (1.03-1.21) | 1.24 (0.92-1.66) | 1.47 (1.36-1.58) | 2.64 (2.17-3.23) | 0.91 (0.85-0.97) | 1.41 (1.16-1.71) |
| Molnupiravir | 0.09 (0.08-0.11) | 0.05 (0.04-0.07) | 0.66 (0.51-0.85) | 0.68 (0.37-1.27) | 0.80 (0.49-1.30) | 0.51 (0.13-2.06) | 0.81 (0.48-1.38) | 1.22 (0.46-3.26) | 0.81 (0.53-1.24) | 0.69 (0.26-1.84) |

**C. Monoclonal Therapies**

| **Characteristic** | **Monoclonal Antibodies, November 10, 2020 – January 26, 2023** | | | | | | | | | |
| --- | --- | --- | --- | --- | --- | --- | --- | --- | --- | --- |
|  | **Hospitalization** | | **AKI/Dialysis** | | **MACE** | | **IMV/ECMO** | | **Death** | |
|  | **Urban** | **Rural** | **Urban** | **Rural** | **Urban** | **Rural** | **Urban** | **Rural** | **Urban** | **Rural** |
|  | **Adjusted Odds Ratio  (95% CI)** | **Adjusted Odds Ratio  (95% CI)** | **Adjusted Hazard Ratio  (95% CI)** | **Adjusted Hazard Ratio  (95% CI)** | **Adjusted Hazard Ratio (95% CI)** | **Adjusted Hazard Ratio (95% CI)** | **Adjusted Hazard Ratio (95% CI)** | **Adjusted Hazard Ratio (95% CI)** | **Adjusted Hazard Ratio (95% CI)** | **Adjusted Hazard Ratio (95% CI)** |
| Sex |  |  |  |  |  |  |  |  |  |  |
| Female | Reference | Reference | Reference | Reference | Reference | Reference | Reference | Reference | Reference | Reference |
| Male | 1.23 (1.22-1.24) | 1.25 (1.22-1.27) | 1.47 (1.44-1.49) | 1.40 (1.35-1.46) | 1.41 (1.36-1.46) | 1.44 (1.33-1.56) | 1.54 (1.50-1.59) | 1.45 (1.37-1.54) | 1.39 (1.35-1.42) | 1.27 (1.20-1.34) |
| Age at COVID-19 Diagnosis | 1.04 (1.04-1.04) | 1.04 (1.04-1.04) | 1.02 (1.02-1.02) | 1.01 (1.01-1.02) | 1.03 (1.03-1.03) | 1.02 (1.02-1.03) | 1.00 (1.00-1.00) | 1.00 (1.00-1.00) | 1.04 (1.04-1.04) | 1.03 (1.03-1.04) |
| Race/Ethnicity |  |  |  |  |  |  |  |  |  |  |
| White Non-Hispanic | Reference | Reference | Reference | Reference | Reference | Reference | Reference | Reference | Reference | Reference |
| Black or African American Non-Hispanic | 1.75 (1.73-1.77) | 1.20 (1.15-1.25) | 1.62 (1.58-1.65) | 1.70 (1.59-1.81) | 1.22 (1.17-1.28) | 1.42 (1.25-1.62) | 1.01 (0.98-1.06) | 1.16 (1.05-1.29) | 0.93 (0.90-0.96) | 1.05 (0.94-1.17) |
| Hispanic or Latino Any Race | 1.86 (1.83-1.89) | 1.52 (1.45-1.60) | 1.08 (1.05-1.11) | 1.16 (1.04-1.29) | 0.95 (0.90-1.02) | 0.96 (0.75-1.22) | 1.12 (1.07-1.17) | 1.25 (1.10-1.41) | 1.12 (1.07-1.17) | 1.05 (0.91-1.22) |
| Other | 1.06 (1.03-1.08) | 0.39 (0.36-0.43) | 1.24 (1.19-1.30) | 1.13 (0.93-1.37) | 1.28 (1.17-1.39) | 1.32 (0.92-1.89) | 1.29 (1.21-1.39) | 1.24 (0.95-1.60) | 1.19 (1.11-1.27) | 1.10 (0.85-1.42) |
| Missing/Unknown | 0.95 (0.93-0.97) | 0.88 (0.82-0.94) | 1.17 (1.13-1.23) | 1.52 (1.35-1.73) | 1.28 (1.18-1.38) | 1.85 (1.49-2.31) | 1.31 (1.23-1.40) | 1.68 (1.45-1.94) | 1.21 (1.14-1.29) | 1.83 (1.59-2.12) |
| Myocardial infarction (MI) | 1.20 (1.17-1.23) | 1.13 (1.07-1.18) | 1.03 (1.00-1.06) | 0.95 (0.89-1.02) | 2.75 (2.62-2.89) | 2.69 (2.40-3.02) | 1.03 (0.97-1.09) | 1.08 (0.95-1.23) | 1.05 (1.00-1.10) | 1.07 (0.97-1.18) |
| Congestive heart failure (CHF) | 1.72 (1.69-1.75) | 1.53 (1.47-1.59) | 1.06 (1.03-1.08) | 1.05 (0.99-1.11) | 1.22 (1.17-1.29) | 1.17 (1.04-1.31) | 1.04 (0.99-1.09) | 0.96 (0.86-1.07) | 1.12 (1.08-1.17) | 1.11 (1.02-1.20) |
| Peripheral vascular disease (PVD) | 1.10 (1.07-1.12) | 1.10 (1.05-1.15) | 0.97 (0.95-1.00) | 0.99 (0.93-1.06) | 1.04 (0.99-1.10) | 1.08 (0.94-1.23) | 0.96 (0.90-1.02) | 0.96 (0.84-1.09) | 1.05 (1.00-1.10) | 0.95 (0.86-1.05) |
| Cerebrovascular disease (CVD) | 0.97 (0.95-0.99) | 0.97 (0.92-1.02) | 0.96 (0.93-0.99) | 0.91 (0.84-0.98) | 1.15 (1.09-1.22) | 1.10 (0.96-1.26) | 0.97 (0.91-1.03) | 0.94 (0.82-1.09) | 0.92 (0.88-0.97) | 0.98 (0.89-1.09) |
| Dementia | 1.50 (1.46-1.54) | 1.06 (0.99-1.14) | 0.82 (0.79-0.85) | 0.91 (0.82-1.00) | 0.73 (0.68-0.78) | 0.54 (0.44-0.67) | 0.58 (0.53-0.63) | 0.44 (0.34-0.57) | 1.15 (1.10-1.21) | 1.17 (1.04-1.31) |
| Chronic pulmonary disease (CPD) | 1.16 (1.15-1.18) | 1.27 (1.23-1.31) | 0.92 (0.90-0.94) | 0.93 (0.88-0.97) | 0.89 (0.85-0.94) | 0.85 (0.77-0.95) | 1.05 (1.01-1.09) | 1.06 (0.97-1.15) | 1.06 (1.02-1.10) | 1.05 (0.98-1.13) |
| Rheumatologic disease (RD) | 0.96 (0.94-0.98) | 1.03 (0.98-1.07) | 1.10 (1.08-1.13) | 1.16 (1.09-1.23) | 1.02 (0.96-1.08) | 0.93 (0.81-1.06) | 1.11 (1.05-1.18) | 1.02 (0.91-1.15) | 1.02 (0.97-1.07) | 1.07 (0.98-1.18) |
| Peptic ulcer disease (PUD) | 1.07 (1.04-1.11) | 1.10 (1.01-1.20) | 1.08 (1.04-1.14) | 0.94 (0.84-1.06) | 0.99 (0.89-1.09) | 0.72 (0.55-0.95) | 1.05 (0.96-1.16) | 0.92 (0.73-1.16) | 0.99 (0.91-1.07) | 0.86 (0.71-1.03) |
| Liver Disease |  |  |  |  |  |  |  |  |  |  |
| No History of Liver Disease | Reference | Reference | Reference | Reference | Reference | Reference | Reference | Reference | Reference | Reference |
| Mild Liver Disease | 0.98 (0.96-1.00) | 1.03 (0.98-1.08) | 1.03 (1.00-1.07) | 0.98 (0.90-1.07) | 0.93 (0.87-1.01) | 0.74 (0.61-0.91) | 1.10 (1.04-1.17) | 0.90 (0.77-1.04) | 1.02 (0.96-1.08) | 1.01 (0.88-1.15) |
| Moderate to Severe Liver Disease | 1.70 (1.63-1.76) | 1.71 (1.56-1.86) | 1.37 (1.31-1.43) | 1.29 (1.16-1.43) | 0.91 (0.81-1.02) | 0.81 (0.61-1.06) | 1.38 (1.27-1.51) | 1.37 (1.14-1.64) | 1.66 (1.54-1.79) | 1.70 (1.45-1.98) |
| Diabetes Mellitus |  |  |  |  |  |  |  |  |  |  |
| No History of Diabetes | Reference | Reference | Reference | Reference | Reference | Reference | Reference | Reference | Reference | Reference |
| Diabetes without Chronic Complications | 1.38 (1.36-1.41) | 1.43 (1.38-1.49) | 1.07 (1.04-1.11) | 1.00 (0.93-1.08) | 1.04 (0.98-1.11) | 0.96 (0.83-1.11) | 1.06 (1.01-1.12) | 0.98 (0.87-1.09) | 1.04 (0.99-1.09) | 1.02 (0.92-1.12) |
| Diabetes with Chronic Complications | 1.37 (1.34-1.39) | 1.53 (1.47-1.59) | 1.26 (1.23-1.29) | 1.24 (1.17-1.31) | 1.13 (1.07-1.19) | 1.24 (1.10-1.40) | 1.23 (1.17-1.29) | 1.12 (1.01-1.25) | 1.10 (1.06-1.15) | 1.10 (1.01-1.20) |
| Hemiplegia or paraplegia | 1.92 (1.85-2.00) | 1.57 (1.43-1.72) | 0.94 (0.89-0.98) | 0.95 (0.83-1.08) | 0.91 (0.82-1.00) | 0.98 (0.78-1.25) | 1.00 (0.90-1.10) | 0.90 (0.71-1.14) | 1.09 (1.00-1.17) | 1.09 (0.91-1.30) |
| Renal Disease | 1.84 (1.81-1.86) | 1.66 (1.60-1.72) | 2.37 (2.32-2.42) | 2.25 (2.13-2.37) | 1.03 (0.99-1.08) | 1.06 (0.95-1.18) | 1.03 (0.99-1.08) | 1.05 (0.95-1.15) | 1.20 (1.16-1.24) | 1.18 (1.09-1.27) |
| Cancer |  |  |  |  |  |  |  |  |  |  |
| No History of Malignant Cancer | Reference | Reference | Reference | Reference | Reference | Reference | Reference | Reference | Reference | Reference |
| Any Malignancy Except Neoplasm of skin | 0.96 (0.95-0.98) | 1.10 (1.06-1.15) | 0.92 (0.90-0.95) | 0.98 (0.93-1.05) | 0.81 (0.77-0.86) | 0.84 (0.73-0.95) | 0.86 (0.82-0.91) | 0.86 (0.77-0.97) | 1.09 (1.05-1.14) | 1.13 (1.05-1.23) |
| Metastatic Solid Tumor | 1.86 (1.79-1.92) | 2.13 (1.97-2.30) | 0.92 (0.87-0.96) | 0.97 (0.87-1.10) | 0.79 (0.70-0.88) | 0.78 (0.60-1.01) | 0.87 (0.79-0.97) | 0.76 (0.60-0.94) | 2.09 (1.97-2.22) | 1.88 (1.65-2.14) |
| HIV | 1.04 (0.98-1.10) | 0.80 (0.64-0.99) | 0.96 (0.88-1.04) | 0.91 (0.65-1.29) | 0.99 (0.81-1.21) | 0.72 (0.32-1.61) | 0.92 (0.78-1.09) | 0.46 (0.20-1.02) | 1.01 (0.84-1.20) | 1.24 (0.73-2.09) |
| Obesity | 1.00 (0.99-1.01) | 0.93 (0.91-0.95) | 1.05 (1.03-1.07) | 1.08 (1.03-1.13) | 0.94 (0.90-0.98) | 1.00 (0.91-1.09) | 1.09 (1.05-1.13) | 1.10 (1.03-1.19) | 1.03 (0.99-1.06) | 0.90 (0.85-0.97) |
| Hypertension | 0.84 (0.83-0.85) | 0.85 (0.83-0.88) | 1.14 (1.11-1.16) | 1.14 (1.08-1.20) | 1.06 (1.01-1.11) | 1.08 (0.97-1.20) | 0.89 (0.85-0.93) | 0.86 (0.80-0.94) | 0.84 (0.81-0.87) | 0.87 (0.81-0.93) |
| Former or Current Tobacco User | 1.51 (1.48-1.54) | 1.65 (1.59-1.72) | 1.09 (1.06-1.12) | 1.05 (0.98-1.12) | 1.22 (1.15-1.29) | 1.24 (1.09-1.40) | 0.94 (0.89-1.00) | 0.89 (0.80-0.98) | 0.94 (0.89-0.99) | 0.95 (0.86-1.04) |
| History of Substance Abuse Disorder | 2.65 (2.59-2.71) | 2.32 (2.20-2.44) | 0.99 (0.95-1.02) | 1.06 (0.97-1.16) | 0.98 (0.91-1.06) | 1.02 (0.85-1.22) | 1.11 (1.05-1.17) | 1.13 (1.00-1.28) | 0.96 (0.90-1.02) | 0.92 (0.80-1.05) |
| Vaccination Status Prior to SARS-CoV-2 Infection |  |  |  |  |  |  |  |  |  |  |
| No Documented COVID-19 Vaccination | Reference | Reference | Reference | Reference | Reference | Reference | Reference | Reference | Reference | Reference |
| Primary Vaccination Series | 0.50 (0.49-0.50) | 0.49 (0.47-0.50) | 0.81 (0.78-0.83) | 0.77 (0.72-0.83) | 0.74 (0.69-0.79) | 0.85 (0.74-0.97) | 0.63 (0.60-0.67) | 0.54 (0.47-0.62) | 0.70 (0.67-0.74) | 0.64 (0.59-0.71) |
| Primary+ Vaccination Series | 0.34 (0.34-0.35) | 0.34 (0.33-0.36) | 0.72 (0.69-0.75) | 0.71 (0.65-0.77) | 0.71 (0.66-0.77) | 0.80 (0.68-0.94) | 0.52 (0.48-0.57) | 0.50 (0.41-0.59) | 0.50 (0.47-0.53) | 0.44 (0.38-0.50) |
| Census Region |  |  |  |  |  |  |  |  |  |  |
| Midwest | Reference | Reference | Reference | Reference | Reference | Reference | Reference | Reference | Reference | Reference |
| Northeast | 1.21 (1.20-1.23) | 0.57 (0.54-0.59) | 1.29 (1.26-1.33) | 1.57 (1.45-1.70) | 1.63 (1.55-1.72) | 1.92 (1.66-2.22) | 1.05 (1.00-1.11) | 1.81 (1.60-2.04) | 0.83 (0.80-0.87) | 0.89 (0.79-0.99) |
| South | 1.17 (1.16-1.18) | 1.78 (1.74-1.82) | 1.44 (1.40-1.47) | 2.02 (1.93-2.11) | 1.66 (1.59-1.74) | 2.00 (1.83-2.19) | 2.05 (1.97-2.13) | 2.32 (2.16-2.48) | 1.09 (1.05-1.13) | 1.31 (1.23-1.39) |
| West | 0.82 (0.81-0.83) | 1.59 (1.51-1.67) | 1.51 (1.47-1.56) | 2.44 (2.23-2.66) | 1.40 (1.32-1.48) | 1.57 (1.29-1.90) | 2.29 (2.19-2.40) | 3.92 (3.52-4.36) | 1.04 (0.99-1.08) | 1.78 (1.59-1.99) |
| Monoclonal Therapies | 0.12 (0.11-0.12) | 0.13 (0.12-0.14) | 0.54 (0.50-0.58) | 0.55 (0.47-0.64) | 0.44 (0.37-0.52) | 0.52 (0.38-0.71) | 0.44 (0.39-0.50) | 0.48 (0.37-0.61) | 0.56 (0.50-0.63) | 0.56 (0.46-0.68) |

**D. Remdesivir**

| **Characteristic** | **Remdesivir, Available After May 1, 2020** | | | | | | | | | |
| --- | --- | --- | --- | --- | --- | --- | --- | --- | --- | --- |
|  | **Hospitalization** | | **AKI/Dialysis** | | **MACE** | | **IMV/ECMO** | | **Death** | |
|  | **Urban** | **Rural** | **Urban** | **Rural** | **Urban** | **Rural** | **Urban** | **Rural** | **Urban** | **Rural** |
|  | **Adjusted Odds Ratio**  **(95% CI)** | **Adjusted Odds Ratio**  **(95% CI)** | **Adjusted Hazard Ratio (95% CI)** | **Adjusted Hazard Ratio (95% CI)** | **Adjusted Hazard Ratio (95% CI)** | **Adjusted Hazard Ratio (95% CI)** | **Adjusted Hazard Ratio (95% CI)** | **Adjusted Hazard Ratio (95% CI)** | **Adjusted Hazard Ratio (95% CI)** | **Adjusted Hazard Ratio (95% CI)** |
| Sex |  |  |  |  |  |  |  |  |  |  |
| Female | N/A | N/A | Reference | Reference | Reference | Reference | Reference | Reference | Reference | Reference |
| Male | N/A | N/A | 1.48 (1.46-1.51) | 1.41 (1.36-1.47) | 1.44 (1.39-1.49) | 1.47 (1.36-1.59) | 1.57 (1.53-1.62) | 1.48 (1.39-1.57) | 1.38 (1.34-1.42) | 1.26 (1.20-1.33) |
| Age at COVID-19 Diagnosis | N/A | N/A | 1.02 (1.02-1.02) | 1.02 (1.01-1.02) | 1.03 (1.03-1.03) | 1.03 (1.02-1.03) | 1.00 (1.00-1.01) | 1.00 (1.00-1.00) | 1.04 (1.04-1.04) | 1.03 (1.03-1.04) |
| Race/Ethnicity |  |  |  |  |  |  |  |  |  |  |
| White Non-Hispanic | N/A | N/A | Reference | Reference | Reference | Reference | Reference | Reference | Reference | Reference |
| Black or African American Non-Hispanic | N/A | N/A | 1.60 (1.57-1.64) | 1.78 (1.67-1.89) | 1.25 (1.20-1.30) | 1.55 (1.36-1.76) | 1.03 (0.99-1.07) | 1.22 (1.10-1.35) | 0.92 (0.89-0.96) | 1.02 (0.92-1.14) |
| Hispanic or Latino Any Race | N/A | N/A | 1.08 (1.05-1.11) | 1.25 (1.13-1.39) | 0.98 (0.92-1.04) | 1.05 (0.84-1.33) | 1.13 (1.08-1.18) | 1.29 (1.14-1.47) | 1.12 (1.07-1.17) | 1.03 (0.89-1.19) |
| Other | N/A | N/A | 1.23 (1.17-1.28) | 1.15 (0.95-1.38) | 1.28 (1.18-1.40) | 1.33 (0.95-1.87) | 1.29 (1.21-1.39) | 1.19 (0.92-1.54) | 1.19 (1.11-1.27) | 1.09 (0.85-1.40) |
| Missing/Unknown | N/A | N/A | 1.16 (1.11-1.21) | 1.52 (1.35-1.72) | 1.26 (1.16-1.36) | 1.86 (1.50-2.31) | 1.31 (1.23-1.39) | 1.69 (1.47-1.95) | 1.21 (1.14-1.29) | 1.83 (1.59-2.12) |
| Myocardial infarction (MI) | N/A | N/A | 1.02 (0.99-1.04) | 0.93 (0.87-1.00) | 2.68 (2.56-2.81) | 2.57 (2.30-2.88) | 1.03 (0.97-1.09) | 1.07 (0.95-1.22) | 1.05 (1.00-1.10) | 1.07 (0.97-1.18) |
| Congestive heart failure (CHF) | N/A | N/A | 1.05 (1.03-1.07) | 1.07 (1.01-1.13) | 1.22 (1.16-1.28) | 1.17 (1.04-1.31) | 1.04 (0.99-1.09) | 0.98 (0.88-1.09) | 1.13 (1.09-1.17) | 1.11 (1.03-1.21) |
| Peripheral vascular disease (PVD) | N/A | N/A | 0.97 (0.94-0.99) | 1.01 (0.94-1.08) | 1.03 (0.98-1.09) | 1.12 (0.99-1.27) | 0.95 (0.90-1.01) | 0.95 (0.83-1.09) | 1.05 (1.01-1.10) | 0.95 (0.86-1.04) |
| Cerebrovascular disease (CVD) | N/A | N/A | 0.97 (0.94-1.00) | 0.93 (0.86-1.00) | 1.17 (1.10-1.23) | 1.18 (1.04-1.35) | 0.97 (0.91-1.03) | 0.96 (0.83-1.10) | 0.92 (0.88-0.97) | 0.97 (0.88-1.07) |
| Dementia | N/A | N/A | 0.81 (0.79-0.84) | 0.91 (0.83-1.00) | 0.70 (0.66-0.75) | 0.54 (0.44-0.66) | 0.58 (0.54-0.64) | 0.45 (0.35-0.57) | 1.16 (1.10-1.22) | 1.16 (1.04-1.30) |
| Chronic pulmonary disease (CPD) | N/A | N/A | 0.97 (0.95-1.00) | 0.95 (0.90-1.00) | 0.96 (0.92-1.00) | 0.90 (0.81-0.99) | 1.08 (1.04-1.13) | 1.08 (0.99-1.18) | 1.05 (1.01-1.08) | 1.05 (0.98-1.13) |
| Rheumatologic disease (RD) | N/A | N/A | 1.13 (1.10-1.16) | 1.14 (1.08-1.22) | 1.05 (0.99-1.11) | 0.94 (0.82-1.07) | 1.12 (1.06-1.18) | 1.02 (0.91-1.15) | 1.00 (0.96-1.05) | 1.07 (0.98-1.17) |
| Peptic ulcer disease (PUD) | N/A | N/A | 1.09 (1.04-1.14) | 0.94 (0.84-1.06) | 0.99 (0.90-1.09) | 0.72 (0.55-0.95) | 1.06 (0.96-1.16) | 0.92 (0.74-1.16) | 0.98 (0.91-1.06) | 0.85 (0.71-1.03) |
| Liver Disease |  |  |  |  |  |  |  |  |  |  |
| No History of Liver Disease | N/A | N/A | Reference | Reference | Reference | Reference | Reference | Reference | Reference | Reference |
| Mild Liver Disease | N/A | N/A | 1.03 (1.00-1.07) | 0.99 (0.91-1.07) | 0.95 (0.88-1.02) | 0.74 (0.61-0.90) | 1.10 (1.04-1.17) | 0.89 (0.77-1.03) | 1.02 (0.96-1.08) | 1.01 (0.88-1.14) |
| Moderate to Severe Liver Disease | N/A | N/A | 1.34 (1.28-1.40) | 1.24 (1.12-1.38) | 0.86 (0.77-0.96) | 0.73 (0.56-0.96) | 1.37 (1.26-1.48) | 1.32 (1.11-1.58) | 1.69 (1.57-1.82) | 1.66 (1.43-1.94) |
| Diabetes Mellitus |  |  |  |  |  |  |  |  |  |  |
| No History of Diabetes | N/A | N/A | Reference | Reference | Reference | Reference | Reference | Reference | Reference | Reference |
| Diabetes without Chronic Complications | N/A | N/A | 1.08 (1.05-1.11) | 0.99 (0.92-1.06) | 1.05 (0.98-1.11) | 0.96 (0.83-1.11) | 1.06 (1.01-1.12) | 0.96 (0.86-1.08) | 1.04 (0.99-1.09) | 1.01 (0.92-1.11) |
| Diabetes with Chronic Complications | N/A | N/A | 1.24 (1.22-1.27) | 1.20 (1.13-1.27) | 1.13 (1.07-1.19) | 1.19 (1.06-1.33) | 1.22 (1.17-1.29) | 1.10 (0.99-1.22) | 1.09 (1.05-1.14) | 1.11 (1.02-1.21) |
| Hemiplegia or paraplegia | N/A | N/A | 0.94 (0.90-0.99) | 0.93 (0.82-1.06) | 0.90 (0.82-0.99) | 0.92 (0.73-1.16) | 0.99 (0.90-1.09) | 0.91 (0.72-1.15) | 1.09 (1.01-1.18) | 1.07 (0.89-1.27) |
| Renal Disease | N/A | N/A | 2.28 (2.24-2.34) | 2.20 (2.09-2.32) | 1.02 (0.97-1.06) | 1.05 (0.94-1.17) | 1.02 (0.97-1.06) | 1.03 (0.94-1.13) | 1.19 (1.15-1.24) | 1.18 (1.09-1.27) |
| Cancer |  |  |  |  |  |  |  |  |  |  |
| No History of Malignant Cancer | N/A | N/A | Reference | Reference | Reference | Reference | Reference | Reference | Reference | Reference |
| Any Malignancy Except Neoplasm of skin | N/A | N/A | 0.92 (0.90-0.94) | 0.97 (0.91-1.03) | 0.81 (0.77-0.86) | 0.84 (0.74-0.95) | 0.85 (0.81-0.90) | 0.86 (0.77-0.96) | 1.09 (1.05-1.13) | 1.13 (1.04-1.22) |
| Metastatic Solid Tumor | N/A | N/A | 0.93 (0.89-0.98) | 0.98 (0.87-1.10) | 0.77 (0.69-0.86) | 0.77 (0.59-0.99) | 0.86 (0.78-0.95) | 0.75 (0.60-0.94) | 2.08 (1.96-2.21) | 1.87 (1.64-2.13) |
| HIV | N/A | N/A | 0.96 (0.88-1.05) | 0.91 (0.65-1.28) | 0.95 (0.78-1.17) | 0.68 (0.31-1.53) | 0.92 (0.77-1.08) | 0.44 (0.20-0.98) | 0.99 (0.83-1.19) | 1.20 (0.71-2.03) |
| Obesity | N/A | N/A | 1.12 (1.09-1.14) | 1.17 (1.12-1.23) | 1.00 (0.96-1.04) | 1.11 (1.01-1.22) | 1.12 (1.08-1.16) | 1.16 (1.08-1.25) | 1.00 (0.97-1.03) | 0.87 (0.82-0.93) |
| Hypertension | N/A | N/A | 1.14 (1.11-1.16) | 1.16 (1.10-1.22) | 1.07 (1.02-1.12) | 1.10 (0.99-1.22) | 0.89 (0.85-0.92) | 0.86 (0.79-0.94) | 0.84 (0.81-0.87) | 0.86 (0.80-0.93) |
| Former or Current Tobacco User | N/A | N/A | 1.10 (1.07-1.13) | 1.05 (0.99-1.12) | 1.26 (1.19-1.33) | 1.25 (1.11-1.41) | 0.97 (0.92-1.02) | 0.89 (0.80-0.98) | 0.95 (0.90-1.00) | 0.95 (0.86-1.04) |
| History of Substance Abuse Disorder | N/A | N/A | 0.96 (0.92-0.99) | 1.02 (0.94-1.11) | 0.90 (0.84-0.97) | 0.96 (0.81-1.15) | 1.08 (1.02-1.14) | 1.10 (0.97-1.25) | 0.98 (0.92-1.04) | 0.93 (0.81-1.07) |
| Vaccination Status Prior to SARS-CoV-2 Infection |  |  |  |  |  |  |  |  |  |  |
| No Documented COVID-19 Vaccination | N/A | N/A | Reference | Reference | Reference | Reference | Reference | Reference | Reference | Reference |
| Primary Vaccination Series | N/A | N/A | 0.79 (0.77-0.82) | 0.74 (0.69-0.79) | 0.74 (0.70-0.79) | 0.80 (0.71-0.92) | 0.62 (0.59-0.66) | 0.52 (0.46-0.60) | 0.70 (0.67-0.73) | 0.64 (0.59-0.70) |
| Primary+ Vaccination Series | N/A | N/A | 0.72 (0.69-0.74) | 0.69 (0.64-0.75) | 0.71 (0.66-0.76) | 0.75 (0.64-0.87) | 0.52 (0.48-0.56) | 0.49 (0.41-0.58) | 0.50 (0.47-0.53) | 0.45 (0.40-0.51) |
| Census Region |  |  |  |  |  |  |  |  |  |  |
| Midwest | N/A | N/A | Reference | Reference | Reference | Reference | Reference | Reference | Reference | Reference |
| Northeast | N/A | N/A | 1.35 (1.32-1.39) | 1.36 (1.26-1.48) | 1.71 (1.63-1.80) | 1.59 (1.38-1.83) | 1.06 (1.01-1.12) | 1.67 (1.48-1.89) | 0.82 (0.79-0.85) | 0.91 (0.82-1.02) |
| South | N/A | N/A | 1.65 (1.62-1.69) | 2.15 (2.06-2.25) | 2.00 (1.91-2.09) | 2.21 (2.02-2.42) | 2.15 (2.07-2.24) | 2.42 (2.26-2.59) | 1.03 (1.00-1.07) | 1.27 (1.20-1.35) |
| West | N/A | N/A | 1.60 (1.55-1.64) | 2.67 (2.45-2.92) | 1.50 (1.42-1.59) | 1.81 (1.49-2.20) | 2.32 (2.22-2.43) | 4.21 (3.78-4.68) | 1.00 (0.96-1.04) | 1.74 (1.56-1.95) |
| Remdesivir | N/A | N/A | 0.28 (0.27-0.29) | 0.35 (0.32-0.37) | 0.20 (0.19-0.22) | 0.25 (0.22-0.29) | 0.64 (0.62-0.66) | 0.57 (0.53-0.62) | 1.25 (1.21-1.28) | 1.15 (1.08-1.22) |

**E. Anticoagulants**

| **Characteristic** | **Anticoagulants, All Time Periods** | | | | | | | | | |
| --- | --- | --- | --- | --- | --- | --- | --- | --- | --- | --- |
|  | **Hospitalization** | | **AKI/Dialysis** | | **MACE** | | **IMV/ECMO** | | **Death** | |
|  | **Urban** | **Rural** | **Urban** | **Rural** | **Urban** | **Rural** | **Urban** | **Rural** | **Urban** | **Rural** |
|  | **Adjusted Odds Ratio  (95% CI)** | **Adjusted Odds Ratio  (95% CI)** | **Adjusted Hazard Ratio  (95% CI)** | **Adjusted Hazard Ratio  (95% CI)** | **Adjusted Hazard Ratio (95% CI)** | **Adjusted Hazard Ratio (95% CI)** | **Adjusted Hazard Ratio (95% CI)** | **Adjusted Hazard Ratio (95% CI)** | **Adjusted Hazard Ratio (95% CI)** | **Adjusted Hazard Ratio (95% CI)** |
| Sex |  |  |  |  |  |  |  |  |  |  |
| Female | N/A | N/A | Reference | Reference | Reference | Reference | Reference | Reference | Reference | Reference |
| Male | N/A | N/A | 1.49 (1.47-1.52) | 1.43 (1.37-1.49) | 1.45 (1.40-1.51) | 1.50 (1.39-1.63) | 1.63 (1.58-1.68) | 1.53 (1.44-1.62) | 1.39 (1.36-1.43) | 1.27 (1.21-1.34) |
| Age at COVID-19 Diagnosis | N/A | N/A | 1.02 (1.02-1.02) | 1.02 (1.01-1.02) | 1.03 (1.03-1.03) | 1.03 (1.02-1.03) | 1.01 (1.00-1.01) | 1.00 (1.00-1.00) | 1.04 (1.04-1.04) | 1.03 (1.03-1.04) |
| Race/Ethnicity |  |  |  |  |  |  |  |  |  |  |
| White Non-Hispanic | N/A | N/A | Reference | Reference | Reference | Reference | Reference | Reference | Reference | Reference |
| Black or African American Non-Hispanic | N/A | N/A | 1.64 (1.60-1.67) | 1.66 (1.56-1.76) | 1.29 (1.24-1.35) | 1.42 (1.25-1.61) | 1.06 (1.02-1.10) | 1.18 (1.07-1.31) | 0.93 (0.90-0.97) | 1.03 (0.93-1.15) |
| Hispanic or Latino Any Race | N/A | N/A | 1.12 (1.09-1.16) | 1.20 (1.08-1.33) | 1.02 (0.96-1.08) | 1.02 (0.81-1.29) | 1.16 (1.10-1.21) | 1.27 (1.12-1.44) | 1.13 (1.08-1.18) | 1.05 (0.90-1.21) |
| Other | N/A | N/A | 1.22 (1.17-1.27) | 1.13 (0.94-1.36) | 1.28 (1.17-1.39) | 1.28 (0.91-1.79) | 1.29 (1.20-1.38) | 1.15 (0.89-1.49) | 1.19 (1.11-1.27) | 1.08 (0.84-1.39) |
| Missing/Unknown | N/A | N/A | 1.21 (1.16-1.26) | 1.61 (1.42-1.82) | 1.32 (1.22-1.42) | 1.92 (1.55-2.39) | 1.33 (1.25-1.42) | 1.74 (1.51-2.01) | 1.22 (1.14-1.30) | 1.86 (1.61-2.15) |
| Myocardial infarction (MI) | N/A | N/A | 1.01 (0.98-1.03) | 0.91 (0.85-0.98) | 2.62 (2.50-2.75) | 2.49 (2.23-2.79) | 1.01 (0.96-1.08) | 1.04 (0.92-1.18) | 1.05 (1.00-1.10) | 1.06 (0.97-1.17) |
| Congestive heart failure (CHF) | N/A | N/A | 0.96 (0.94-0.99) | 1.01 (0.95-1.07) | 1.11 (1.06-1.17) | 1.10 (0.98-1.23) | 0.97 (0.93-1.02) | 0.94 (0.85-1.05) | 1.12 (1.07-1.16) | 1.11 (1.02-1.20) |
| Peripheral vascular disease (PVD) | N/A | N/A | 0.95 (0.93-0.98) | 0.97 (0.91-1.04) | 1.02 (0.96-1.07) | 1.07 (0.94-1.22) | 0.94 (0.89-1.00) | 0.93 (0.81-1.06) | 1.05 (1.00-1.10) | 0.95 (0.86-1.05) |
| Cerebrovascular disease (CVD) | N/A | N/A | 0.94 (0.91-0.97) | 0.93 (0.86-1.00) | 1.14 (1.08-1.20) | 1.16 (1.02-1.33) | 0.95 (0.89-1.01) | 0.95 (0.83-1.09) | 0.92 (0.88-0.97) | 0.98 (0.89-1.08) |
| Dementia | N/A | N/A | 0.81 (0.78-0.84) | 0.91 (0.83-1.00) | 0.69 (0.64-0.74) | 0.53 (0.43-0.65) | 0.57 (0.53-0.63) | 0.44 (0.34-0.56) | 1.15 (1.10-1.21) | 1.17 (1.04-1.31) |
| Chronic pulmonary disease (CPD) | N/A | N/A | 0.94 (0.92-0.96) | 0.93 (0.89-0.98) | 0.90 (0.87-0.95) | 0.85 (0.77-0.95) | 1.06 (1.02-1.11) | 1.07 (0.99-1.17) | 1.06 (1.03-1.10) | 1.06 (0.99-1.14) |
| Rheumatologic disease (RD) | N/A | N/A | 1.10 (1.07-1.13) | 1.12 (1.05-1.19) | 1.02 (0.96-1.08) | 0.90 (0.79-1.03) | 1.12 (1.06-1.18) | 1.02 (0.90-1.14) | 1.01 (0.97-1.06) | 1.07 (0.97-1.17) |
| Peptic ulcer disease (PUD) | N/A | N/A | 1.03 (0.99-1.08) | 0.93 (0.82-1.04) | 0.94 (0.85-1.04) | 0.71 (0.54-0.94) | 1.03 (0.94-1.13) | 0.91 (0.72-1.14) | 0.98 (0.91-1.06) | 0.85 (0.71-1.02) |
| Liver Disease |  |  |  |  |  |  |  |  |  |  |
| No History of Liver Disease | N/A | N/A | Reference | Reference | Reference | Reference | Reference | Reference | Reference | Reference |
| Mild Liver Disease | N/A | N/A | 1.03 (1.00-1.07) | 0.97 (0.89-1.06) | 0.95 (0.88-1.02) | 0.73 (0.60-0.89) | 1.11 (1.04-1.17) | 0.89 (0.76-1.03) | 1.02 (0.96-1.08) | 1.00 (0.88-1.14) |
| Moderate to Severe Liver Disease | N/A | N/A | 1.26 (1.21-1.32) | 1.18 (1.06-1.31) | 0.80 (0.72-0.90) | 0.67 (0.51-0.88) | 1.29 (1.18-1.40) | 1.23 (1.03-1.46) | 1.66 (1.54-1.78) | 1.63 (1.39-1.90) |
| Diabetes Mellitus |  |  |  |  |  |  |  |  |  |  |
| No History of Diabetes | N/A | N/A | Reference | Reference | Reference | Reference | Reference | Reference | Reference | Reference |
| Diabetes without Chronic Complications | N/A | N/A | 1.08 (1.05-1.11) | 1.00 (0.93-1.07) | 1.04 (0.98-1.11) | 0.96 (0.83-1.10) | 1.06 (1.00-1.12) | 0.96 (0.86-1.07) | 1.04 (0.99-1.09) | 1.01 (0.91-1.11) |
| Diabetes with Chronic Complications | N/A | N/A | 1.20 (1.18-1.23) | 1.19 (1.12-1.26) | 1.09 (1.03-1.15) | 1.18 (1.05-1.33) | 1.20 (1.14-1.26) | 1.09 (0.98-1.21) | 1.09 (1.04-1.13) | 1.10 (1.01-1.19) |
| Hemiplegia or paraplegia | N/A | N/A | 0.94 (0.89-0.98) | 0.89 (0.79-1.01) | 0.88 (0.80-0.97) | 0.89 (0.71-1.12) | 0.98 (0.89-1.08) | 0.88 (0.70-1.11) | 1.08 (1.00-1.17) | 1.05 (0.88-1.26) |
| Renal Disease | N/A | N/A | 1.97 (1.93-2.02) | 1.88 (1.78-1.98) | 0.86 (0.82-0.90) | 0.88 (0.79-0.98) | 0.91 (0.87-0.95) | 0.90 (0.82-0.99) | 1.17 (1.13-1.21) | 1.16 (1.08-1.25) |
| Cancer |  |  |  |  |  |  |  |  |  |  |
| No History of Malignant Cancer | N/A | N/A | Reference | Reference | Reference | Reference | Reference | Reference | Reference | Reference |
| Any Malignancy Except Neoplasm of skin | N/A | N/A | 0.89 (0.87-0.91) | 0.95 (0.89-1.01) | 0.78 (0.74-0.82) | 0.81 (0.71-0.92) | 0.83 (0.78-0.87) | 0.84 (0.75-0.94) | 1.09 (1.05-1.13) | 1.13 (1.05-1.23) |
| Metastatic Solid Tumor | N/A | N/A | 0.84 (0.80-0.89) | 0.92 (0.82-1.03) | 0.67 (0.60-0.75) | 0.71 (0.55-0.92) | 0.80 (0.73-0.88) | 0.69 (0.56-0.86) | 2.07 (1.95-2.20) | 1.86 (1.64-2.12) |
| HIV | N/A | N/A | 0.94 (0.86-1.03) | 0.86 (0.61-1.21) | 0.91 (0.75-1.12) | 0.76 (0.34-1.69) | 0.90 (0.76-1.06) | 0.45 (0.20-1.01) | 0.99 (0.83-1.18) | 1.18 (0.70-2.00) |
| Obesity | N/A | N/A | 1.19 (1.17-1.21) | 1.23 (1.17-1.29) | 1.08 (1.03-1.12) | 1.18 (1.07-1.29) | 1.20 (1.16-1.25) | 1.24 (1.15-1.33) | 1.04 (1.01-1.07) | 0.91 (0.85-0.97) |
| Hypertension | N/A | N/A | 1.09 (1.07-1.12) | 1.16 (1.10-1.23) | 1.02 (0.97-1.07) | 1.11 (0.99-1.23) | 0.86 (0.83-0.90) | 0.86 (0.79-0.93) | 0.84 (0.81-0.87) | 0.87 (0.81-0.93) |
| Former or Current Tobacco User | N/A | N/A | 1.11 (1.08-1.14) | 1.07 (1.00-1.15) | 1.26 (1.19-1.34) | 1.24 (1.10-1.40) | 0.99 (0.94-1.04) | 0.90 (0.81-1.00) | 0.96 (0.91-1.01) | 0.95 (0.86-1.04) |
| History of Substance Abuse Disorder | N/A | N/A | 0.98 (0.95-1.02) | 1.04 (0.96-1.14) | 0.93 (0.86-1.00) | 1.00 (0.83-1.19) | 1.06 (1.01-1.13) | 1.11 (0.98-1.26) | 0.97 (0.91-1.03) | 0.93 (0.81-1.07) |
| Vaccination Status Prior to SARS-CoV-2 Infection |  |  |  |  |  |  |  |  |  |  |
| No Documented COVID-19 Vaccination | N/A | N/A | Reference | Reference | Reference | Reference | Reference | Reference | Reference | Reference |
| Primary Vaccination Series | N/A | N/A | 0.76 (0.74-0.78) | 0.70 (0.66-0.75) | 0.70 (0.65-0.74) | 0.77 (0.68-0.88) | 0.60 (0.56-0.63) | 0.50 (0.43-0.56) | 0.69 (0.66-0.73) | 0.63 (0.58-0.69) |
| Primary+ Vaccination Series | N/A | N/A | 0.67 (0.65-0.70) | 0.66 (0.61-0.72) | 0.65 (0.60-0.70) | 0.73 (0.62-0.85) | 0.49 (0.46-0.53) | 0.47 (0.40-0.55) | 0.50 (0.47-0.53) | 0.44 (0.39-0.50) |
| Census Region |  |  |  |  |  |  |  |  |  |  |
| Midwest | N/A | N/A | Reference | Reference | Reference | Reference | Reference | Reference | Reference | Reference |
| Northeast | N/A | N/A | 1.70 (1.66-1.74) | 2.00 (1.84-2.16) | 2.27 (2.16-2.39) | 2.55 (2.20-2.94) | 1.32 (1.25-1.38) | 2.28 (2.02-2.58) | 0.87 (0.83-0.90) | 0.91 (0.82-1.02) |
| South | N/A | N/A | 1.74 (1.70-1.78) | 2.61 (2.49-2.74) | 2.14 (2.05-2.24) | 2.85 (2.60-3.12) | 2.38 (2.29-2.48) | 2.98 (2.78-3.20) | 1.11 (1.08-1.15) | 1.35 (1.27-1.43) |
| West | N/A | N/A | 1.61 (1.57-1.66) | 3.43 (3.14-3.74) | 1.52 (1.43-1.61) | 2.58 (2.12-3.13) | 2.42 (2.31-2.54) | 5.40 (4.85-6.01) | 1.03 (0.98-1.07) | 1.88 (1.68-2.10) |
| Anticoagulants | N/A | N/A | 0.17 (0.16-0.17) | 0.20 (0.19-0.21) | 0.14 (0.13-0.15) | 0.15 (0.13-0.17) | 0.34 (0.33-0.36) | 0.33 (0.30-0.35) | 0.86 (0.83-0.88) | 0.88 (0.83-0.93) |

**F. Tocilizumab**

| **Characteristic** | **Tocilizumab, Available after June 24, 2021** | | | | | | | | | |
| --- | --- | --- | --- | --- | --- | --- | --- | --- | --- | --- |
|  | **Hospitalization** | | **AKI/Dialysis** | | **MACE** | | **IMV/ECMO** | | **Death** | |
|  | **Urban** | **Rural** | **Urban** | **Rural** | **Urban** | **Rural** | **Urban** | **Rural** | **Urban** | **Rural** |
|  | **Adjusted Odds Ratio  (95% CI)** | **Adjusted Odds Ratio  (95% CI)** | **Adjusted Hazard Ratio  (95% CI)** | **Adjusted Hazard Ratio  (95% CI)** | **Adjusted Hazard Ratio (95% CI)** | **Adjusted Hazard Ratio (95% CI)** | **Adjusted Hazard Ratio (95% CI)** | **Adjusted Hazard Ratio (95% CI)** | **Adjusted Hazard Ratio (95% CI)** | **Adjusted Hazard Ratio (95% CI)** |
| Sex |  |  |  |  |  |  |  |  |  |  |
| Female | N/A | N/A | N/A | N/A | N/A | N/A | N/A | N/A | Reference | Reference |
| Male | N/A | N/A | N/A | N/A | N/A | N/A | N/A | N/A | 1.34 (1.30-1.39) | 1.27 (1.20-1.34) |
| Age at COVID-19 Diagnosis | N/A | N/A | N/A | N/A | N/A | N/A | N/A | N/A | 1.04 (1.03-1.04) | 1.03 (1.03-1.04) |
| Race/Ethnicity |  |  |  |  |  |  |  |  |  |  |
| White Non-Hispanic | N/A | N/A | N/A | N/A | N/A | N/A | N/A | N/A | Reference | Reference |
| Black or African American Non-Hispanic | N/A | N/A | N/A | N/A | N/A | N/A | N/A | N/A | 0.89 (0.85-0.93) | 1.06 (0.93-1.20) |
| Hispanic or Latino Any Race | N/A | N/A | N/A | N/A | N/A | N/A | N/A | N/A | 1.03 (0.98-1.10) | 0.98 (0.83-1.16) |
| Other | N/A | N/A | N/A | N/A | N/A | N/A | N/A | N/A | 1.09 (1.00-1.19) | 1.17 (0.90-1.52) |
| Missing/Unknown | N/A | N/A | N/A | N/A | N/A | N/A | N/A | N/A | 1.08 (1.0-1.17) | 1.79 (1.52-2.10) |
| Myocardial infarction (MI) | N/A | N/A | N/A | N/A | N/A | N/A | N/A | N/A | 1.08 (1.02-1.13) | 1.08 (0.97-1.20) |
| Congestive heart failure (CHF) | N/A | N/A | N/A | N/A | N/A | N/A | N/A | N/A | 1.13 (1.08-1.18) | 1.06 (0.97-1.16) |
| Peripheral vascular disease (PVD) | N/A | N/A | N/A | N/A | N/A | N/A | N/A | N/A | 1.04 (0.99-1.10) | 0.95 (0.85-1.06) |
| Cerebrovascular disease (CVD) | N/A | N/A | N/A | N/A | N/A | N/A | N/A | N/A | 0.93 (0.88-0.98) | 0.96 (0.86-1.08) |
| Dementia | N/A | N/A | N/A | N/A | N/A | N/A | N/A | N/A | 1.18 (1.12-1.25) | 1.14 (1.00-1.30) |
| Chronic pulmonary disease (CPD) | N/A | N/A | N/A | N/A | N/A | N/A | N/A | N/A | 1.06 (1.02-1.11) | 1.07 (0.99-1.16) |
| Rheumatologic disease (RD) | N/A | N/A | N/A | N/A | N/A | N/A | N/A | N/A | 1.04 (0.99-1.09) | 1.02 (0.92-1.13) |
| Peptic ulcer disease (PUD) | N/A | N/A | N/A | N/A | N/A | N/A | N/A | N/A | 0.99 (0.91-1.08) | 0.93 (0.77-1.13) |
| Liver Disease |  |  |  |  |  |  |  |  |  |  |
| No History of Liver Disease | N/A | N/A | N/A | N/A | N/A | N/A | N/A | N/A | Reference | Reference |
| Mild Liver Disease | N/A | N/A | N/A | N/A | N/A | N/A | N/A | N/A | 1.03 (0.97-1.10) | 1.01 (0.87-1.16) |
| Moderate to Severe Liver Disease | N/A | N/A | N/A | N/A | N/A | N/A | N/A | N/A | 1.65 (1.52-1.80) | 1.76 (1.49-2.08) |
| Diabetes Mellitus |  |  |  |  |  |  |  |  |  |  |
| No History of Diabetes | N/A | N/A | N/A | N/A | N/A | N/A | N/A | N/A | Reference | Reference |
| Diabetes without Chronic Complications | N/A | N/A | N/A | N/A | N/A | N/A | N/A | N/A | 1.03 (0.97-1.09) | 1.07 (0.96-1.19) |
| Diabetes with Chronic Complications | N/A | N/A | N/A | N/A | N/A | N/A | N/A | N/A | 1.06 (1.01-1.11) | 1.12 (1.02-1.23) |
| Hemiplegia or paraplegia | N/A | N/A | N/A | N/A | N/A | N/A | N/A | N/A | 1.07 (0.98-1.17) | 1.02 (0.84-1.25) |
| Renal Disease | N/A | N/A | N/A | N/A | N/A | N/A | N/A | N/A | 1.18 (1.13-1.23) | 1.18 (1.09-1.29) |
| Cancer |  |  |  |  |  |  |  |  |  |  |
| No History of Malignant Cancer | N/A | N/A | N/A | N/A | N/A | N/A | N/A | N/A | Reference | Reference |
| Any Malignancy Except Neoplasm of skin | N/A | N/A | N/A | N/A | N/A | N/A | N/A | N/A | 1.13 (1.08-1.19) | 1.16 (1.06-1.27) |
| Metastatic Solid Tumor | N/A | N/A | N/A | N/A | N/A | N/A | N/A | N/A | 2.25 (2.11-2.41) | 1.93 (1.68-2.22) |
| HIV | N/A | N/A | N/A | N/A | N/A | N/A | N/A | N/A | 0.92 (0.74-1.15) | 1.07 (0.58-2.00) |
| Obesity | N/A | N/A | N/A | N/A | N/A | N/A | N/A | N/A | 1.01 (0.97-1.04) | 0.89 (0.83-0.96) |
| Hypertension | N/A | N/A | N/A | N/A | N/A | N/A | N/A | N/A | 0.85 (0.82-0.89) | 0.89 (0.82-0.96) |
| Former or Current Tobacco User | N/A | N/A | N/A | N/A | N/A | N/A | N/A | N/A | 0.97 (0.91-1.02) | 0.93 (0.83-1.03) |
| History of Substance Abuse Disorder | N/A | N/A | N/A | N/A | N/A | N/A | N/A | N/A | 1.00 (0.93-1.07) | 0.98 (0.85-1.14) |
| Vaccination Status Prior to SARS-CoV-2 Infection |  |  |  |  |  |  |  |  |  |  |
| No Documented COVID-19 Vaccination | N/A | N/A | N/A | N/A | N/A | N/A | N/A | N/A | Reference | Reference |
| Primary Vaccination Series | N/A | N/A | N/A | N/A | N/A | N/A | N/A | N/A | 0.73 (0.69-0.76) | 0.64 (0.59-0.71) |
| Primary+ Vaccination Series | N/A | N/A | N/A | N/A | N/A | N/A | N/A | N/A | 0.53 (0.49-0.56) | 0.45 (0.40-0.51) |
| Census Region |  |  |  |  |  |  |  |  |  |  |
| Midwest | N/A | N/A | N/A | N/A | N/A | N/A | N/A | N/A | Reference | Reference |
| Northeast | N/A | N/A | N/A | N/A | N/A | N/A | N/A | N/A | 0.73 (0.70-0.77) | 0.87 (0.77-0.98) |
| South | N/A | N/A | N/A | N/A | N/A | N/A | N/A | N/A | 1.02 (0.98-1.06) | 1.18 (1.11-1.26) |
| West | N/A | N/A | N/A | N/A | N/A | N/A | N/A | N/A | 0.94 (0.90-0.99) | 1.59 (1.41-1.79) |
| Tocilizumab | N/A | N/A | N/A | N/A | N/A | N/A | N/A | N/A | 4.01 (3.77-4.27) | 3.69 (3.27-4.16) |

**G. Dexamethasone**

| **Characteristic** | **Dexamethasone, Available after June 16, 2020** | | | | | | | | | |
| --- | --- | --- | --- | --- | --- | --- | --- | --- | --- | --- |
|  | **Hospitalization** | | **AKI/Dialysis** | | **MACE** | | **IMV/ECMO** | | **Death** | |
|  | **Urban** | **Rural** | **Urban** | **Rural** | **Urban** | **Rural** | **Urban** | **Rural** | **Urban** | **Rural** |
|  | **Adjusted Odds Ratio  (95% CI)** | **Adjusted Odds Ratio  (95% CI)** | **Adjusted Hazard Ratio  (95% CI)** | **Adjusted Hazard Ratio  (95% CI)** | **Adjusted Hazard Ratio (95% CI)** | **Adjusted Hazard Ratio (95% CI)** | **Adjusted Hazard Ratio (95% CI)** | **Adjusted Hazard Ratio (95% CI)** | **Adjusted Hazard Ratio (95% CI)** | **Adjusted Hazard Ratio (95% CI)** |
| Sex |  |  |  |  |  |  |  |  |  |  |
| Female | N/A | N/A | N/A | N/A | N/A | N/A | N/A | N/A | Reference | Reference |
| Male | N/A | N/A | N/A | N/A | N/A | N/A | N/A | N/A | 1.36 (1.32-1.39) | 1.24 (1.18-1.31) |
| Age at COVID-19 Diagnosis | N/A | N/A | N/A | N/A | N/A | N/A | N/A | N/A | 1.04 (1.04-1.04) | 1.03 (1.03-1.04) |
| Race/Ethnicity |  |  |  |  |  |  |  |  |  |  |
| White Non-Hispanic | N/A | N/A | N/A | N/A | N/A | N/A | N/A | N/A | Reference | Reference |
| Black or African American Non-Hispanic | N/A | N/A | N/A | N/A | N/A | N/A | N/A | N/A | 0.94 (0.90-0.97) | 1.04 (0.94-1.16) |
| Hispanic or Latino Any Race | N/A | N/A | N/A | N/A | N/A | N/A | N/A | N/A | 1.11 (1.06-1.16) | 1.01 (0.87-1.17) |
| Other | N/A | N/A | N/A | N/A | N/A | N/A | N/A | N/A | 1.20 (1.12-1.28) | 1.12 (0.88-1.44) |
| Missing/Unknown | N/A | N/A | N/A | N/A | N/A | N/A | N/A | N/A | 1.21 (1.13-1.28) | 1.78 (1.54-2.06) |
| Myocardial infarction (MI) | N/A | N/A | N/A | N/A | N/A | N/A | N/A | N/A | 1.05 (1.00-1.10) | 1.08 (0.98-1.19) |
| Congestive heart failure (CHF) | N/A | N/A | N/A | N/A | N/A | N/A | N/A | N/A | 1.14 (1.10-1.18) | 1.12 (1.04-1.22) |
| Peripheral vascular disease (PVD) | N/A | N/A | N/A | N/A | N/A | N/A | N/A | N/A | 1.05 (1.01-1.10) | 0.94 (0.85-1.04) |
| Cerebrovascular disease (CVD) | N/A | N/A | N/A | N/A | N/A | N/A | N/A | N/A | 0.92 (0.88-0.97) | 0.97 (0.88-1.07) |
| Dementia | N/A | N/A | N/A | N/A | N/A | N/A | N/A | N/A | 1.20 (1.15-1.26) | 1.18 (1.05-1.32) |
| Chronic pulmonary disease (CPD) | N/A | N/A | N/A | N/A | N/A | N/A | N/A | N/A | 1.02 (0.98-1.05) | 1.03 (0.96-1.10) |
| Rheumatologic disease (RD) | N/A | N/A | N/A | N/A | N/A | N/A | N/A | N/A | 0.99 (0.95-1.04) | 1.06 (0.97-1.16) |
| Peptic ulcer disease (PUD) | N/A | N/A | N/A | N/A | N/A | N/A | N/A | N/A | 0.99 (0.91-1.07) | 0.85 (0.71-1.02) |
| Liver Disease |  |  |  |  |  |  |  |  |  |  |
| No History of Liver Disease | N/A | N/A | N/A | N/A | N/A | N/A | N/A | N/A | Reference | Reference |
| Mild Liver Disease | N/A | N/A | N/A | N/A | N/A | N/A | N/A | N/A | 1.02 (0.96-1.08) | 1.03 (0.90-1.17) |
| Moderate to Severe Liver Disease | N/A | N/A | N/A | N/A | N/A | N/A | N/A | N/A | 1.74 (1.62-1.87) | 1.74 (1.49-2.03) |
| Diabetes Mellitus |  |  |  |  |  |  |  |  |  |  |
| No History of Diabetes | N/A | N/A | N/A | N/A | N/A | N/A | N/A | N/A | Reference | Reference |
| Diabetes without Chronic Complications | N/A | N/A | N/A | N/A | N/A | N/A | N/A | N/A | 1.05 (1.00-1.10) | 1.02 (0.93-1.12) |
| Diabetes with Chronic Complications | N/A | N/A | N/A | N/A | N/A | N/A | N/A | N/A | 1.10 (1.06-1.15) | 1.13 (1.04-1.23) |
| Hemiplegia or paraplegia | N/A | N/A | N/A | N/A | N/A | N/A | N/A | N/A | 1.10 (1.01-1.18) | 1.10 (0.92-1.31) |
| Renal Disease | N/A | N/A | N/A | N/A | N/A | N/A | N/A | N/A | 1.21 (1.17-1.26) | 1.19 (1.10-1.28) |
| Cancer |  |  |  |  |  |  |  |  |  |  |
| No History of Malignant Cancer | N/A | N/A | N/A | N/A | N/A | N/A | N/A | N/A | Reference | Reference |
| Any Malignancy Except Neoplasm of skin | N/A | N/A | N/A | N/A | N/A | N/A | N/A | N/A | 1.08 (1.04-1.12) | 1.10 (1.02-1.19) |
| Metastatic Solid Tumor | N/A | N/A | N/A | N/A | N/A | N/A | N/A | N/A | 1.99 (1.87-2.11) | 1.78 (1.56-2.03) |
| HIV | N/A | N/A | N/A | N/A | N/A | N/A | N/A | N/A | 1.05 (0.87-1.25) | 1.23 (0.73-2.09) |
| Obesity | N/A | N/A | N/A | N/A | N/A | N/A | N/A | N/A | 0.93 (0.90-0.96) | 0.81 (0.76-0.86) |
| Hypertension | N/A | N/A | N/A | N/A | N/A | N/A | N/A | N/A | 0.85 (0.82-0.88) | 0.85 (0.80-0.92) |
| Former or Current Tobacco User | N/A | N/A | N/A | N/A | N/A | N/A | N/A | N/A | 0.94 (0.89-0.99) | 0.93 (0.85-1.03) |
| History of Substance Abuse Disorder | N/A | N/A | N/A | N/A | N/A | N/A | N/A | N/A | 1.03 (0.97-1.09) | 0.96 (0.84-1.10) |
| Vaccination Status Prior to SARS-CoV-2 Infection |  |  |  |  |  |  |  |  |  |  |
| No Documented COVID-19 Vaccination | N/A | N/A | N/A | N/A | N/A | N/A | N/A | N/A | Reference | Reference |
| Primary Vaccination Series | N/A | N/A | N/A | N/A | N/A | N/A | N/A | N/A | 0.75 (0.72-0.79) | 0.68 (0.62-0.75) |
| Primary+ Vaccination Series | N/A | N/A | N/A | N/A | N/A | N/A | N/A | N/A | 0.56 (0.52-0.59) | 0.49 (0.43-0.55) |
| Census Region |  |  |  |  |  |  |  |  |  |  |
| Midwest | N/A | N/A | N/A | N/A | N/A | N/A | N/A | N/A | Reference | Reference |
| Northeast | N/A | N/A | N/A | N/A | N/A | N/A | N/A | N/A | 0.77 (0.74-0.80) | 0.77 (0.69-0.87) |
| South | N/A | N/A | N/A | N/A | N/A | N/A | N/A | N/A | 0.94 (0.91-0.97) | 1.08 (1.02-1.15) |
| West | N/A | N/A | N/A | N/A | N/A | N/A | N/A | N/A | 0.88 (0.84-0.91) | 1.41 (1.26-1.58) |
| Dexamethasone | N/A | N/A | N/A | N/A | N/A | N/A | N/A | N/A | 1.94 (1.89-1.99) | 1.87 (1.76-1.97) |

**Supplemental References**

1. Benchimol EI, Smeeth L, Guttmann A, et al. The REporting of studies Conducted using Observational Routinely-collected health Data (RECORD) statement. *PLoS Med*. Oct 2015;12(10):e1001885. doi:10.1371/journal.pmed.1001885

2. R Core Team R. R: A language and environment for statistical computing. 2013;

3. Therneau TM, Lumley T. Package ‘survival’. *R Top Doc*. 2015;128(10):28-33.

4. Stuart EA, King G, Imai K, Ho D. MatchIt: nonparametric preprocessing for parametric causal inference. *Journal of statistical software*. 2011;

5. Greifer N. Covariate balance tables and plots: a guide to the cobalt package. *Accessed March*. 2020;10:2020.

6. Daniel dS, Whiting K, Curry M, Lavery JA, Larmarange J. Reproducible summary tables with the gtsummary package. *The R Journal*. 2021;13(1):570-580.

7. Wickham H. ggplot2. *Wiley interdisciplinary reviews: computational statistics*. 2011;3(2):180-185.

8. Larmarange J. ggstats: Extension to'ggplot2'for Plotting Stats. 2023;

9. Di Lorenzo P. usmap: US maps including Alaska and Hawaii. 2021.

10. OHDSI. *The Book of OHDSI: Observational Health Data Sciences and Informatics*. OHDSI; 2019.

11. Anzalone AJ, Horswell R, Hendricks BM, et al. Higher hospitalization and mortality rates among SARS-CoV-2-infected persons in rural America. *J Rural Health*. Jun 27 2022;doi:10.1111/jrh.12689

12. Anzalone AJ, Sun J, Vinson AJ, et al. Community risks for SARS-CoV-2 infection among fully vaccinated US adults by rurality: A retrospective cohort study from the National COVID Cohort Collaborative. *PLoS One*. 2023;18(1):e0279968. doi:10.1371/journal.pone.0279968

13. OMOP CDM v5.3. Observational Health Data Sciences and Informatics (OHDSI). <https://ohdsi.github.io/CommonDataModel/cdm53.html>

14. Dong E, Du H, Gardner L. An interactive web-based dashboard to track COVID-19 in real time. *Lancet Infect Dis*. May 2020;20(5):533-534. doi:10.1016/s1473-3099(20)30120-1

15. Data from: Rural-Urban Continuum Codes 2013.

16. Data from: 2020 Census Results. 2021. *Washington, DC*.

17. Horby P, Lim WS, Emberson JR, et al. Dexamethasone in Hospitalized Patients with Covid-19. *N Engl J Med*. Feb 25 2021;384(8):693-704. doi:10.1056/NEJMoa2021436

18. FDA Approves First Treatment for COVID-19. U.S. Food and Drug Administration (FDA); 10/10/2020, 2020. Accessed 03/15/2023. <https://www.fda.gov/news-events/press-announcements/fda-approves-first-treatment-covid-19>
